# Cycloadditions of 4-Alkenyl-2-aminothiazoles with Nitroalkenes in the Formal Synthesis of Pramipexole: An Experimental and Computational Study

**DOI:** 10.1021/acs.joc.4c00843

**Published:** 2024-08-19

**Authors:** Mateo Alajarin, Jose Cabrera, Delia Bautista, Pilar Sanchez-Andrada, Aurelia Pastor

**Affiliations:** † Department of Organic Chemistry, Faculty of Chemistry, Regional Campus of International Excellence “Campus Mare Nostrum”, University of Murcia, Murcia 30100, Spain; ‡ ACTI, University of Murcia, Murcia 30100, Spain

## Abstract

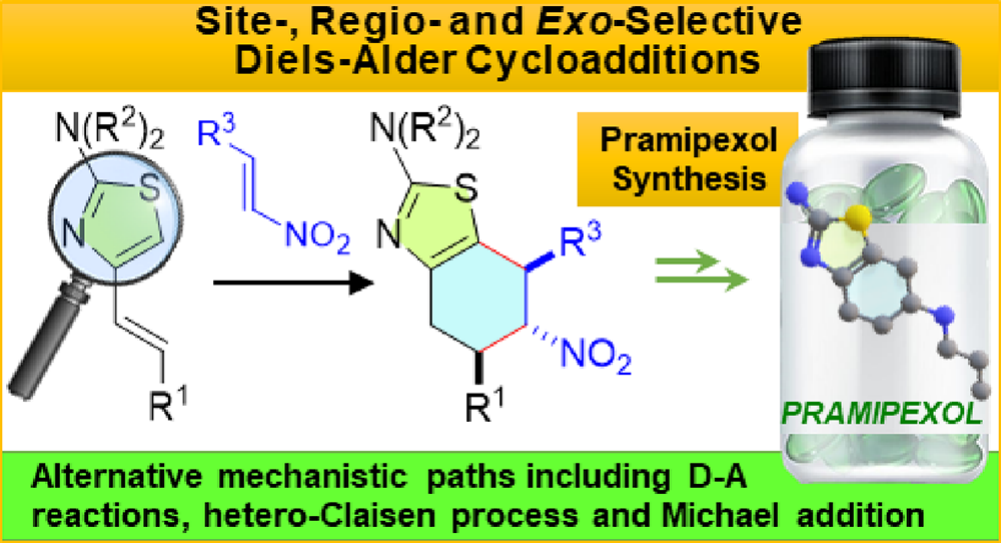

4-Alkenyl-2-dialkylaminothiazoles act as in–out dienes in [4 + 2] cycloaddition reactions with nitroalkenes, furnishing 2-amino-6-nitro-4,5,6,7-tetrahydrobenzothiazoles in moderate to good yields, accompanied by a subsequent 1,3-*H* migration. These transformations proceed with exquisite site-, regio-, and diastereoselectivity. This strategy is further enriched by revealing a novel route for pramipexole synthesis. The examination of the potential energy surfaces associated with the four possible reaction pathways for the Diels–Alder cycloaddition (relative approach of the diene–dienophile and *endo*/*exo* approach of the nitro group) not only aligns with experimental observations but also unveils key mechanistic insights. Specifically, computational analyses uncover the favored pathway yielding 6-nitro-4,5,6,7-tetrahydrobenzothiazoles, with some instances proceeding through a two-step mechanism involving a tandem sequence of chemical processes, and the influence of various factors such as dienophile structure and the approach mode of the nitro group. Additionally, the stabilization of the *exo*-transition states, particularly facilitated by phenyl substitution in the dienophile, is highlighted. Asynchronicity, dipole moment, and other parameters indicative of polar character further characterize these Diels–Alder reactions. Conceptual DFT calculations underscore the pivotal role of the 1,3-thiazole ring in enhancing dienic activation and dictating regioselectivity, emphasizing interactions between the C5 of the thiazole nucleus and the Cβ atom of the nitroalkenes.

## Introduction

**Scheme 1:**
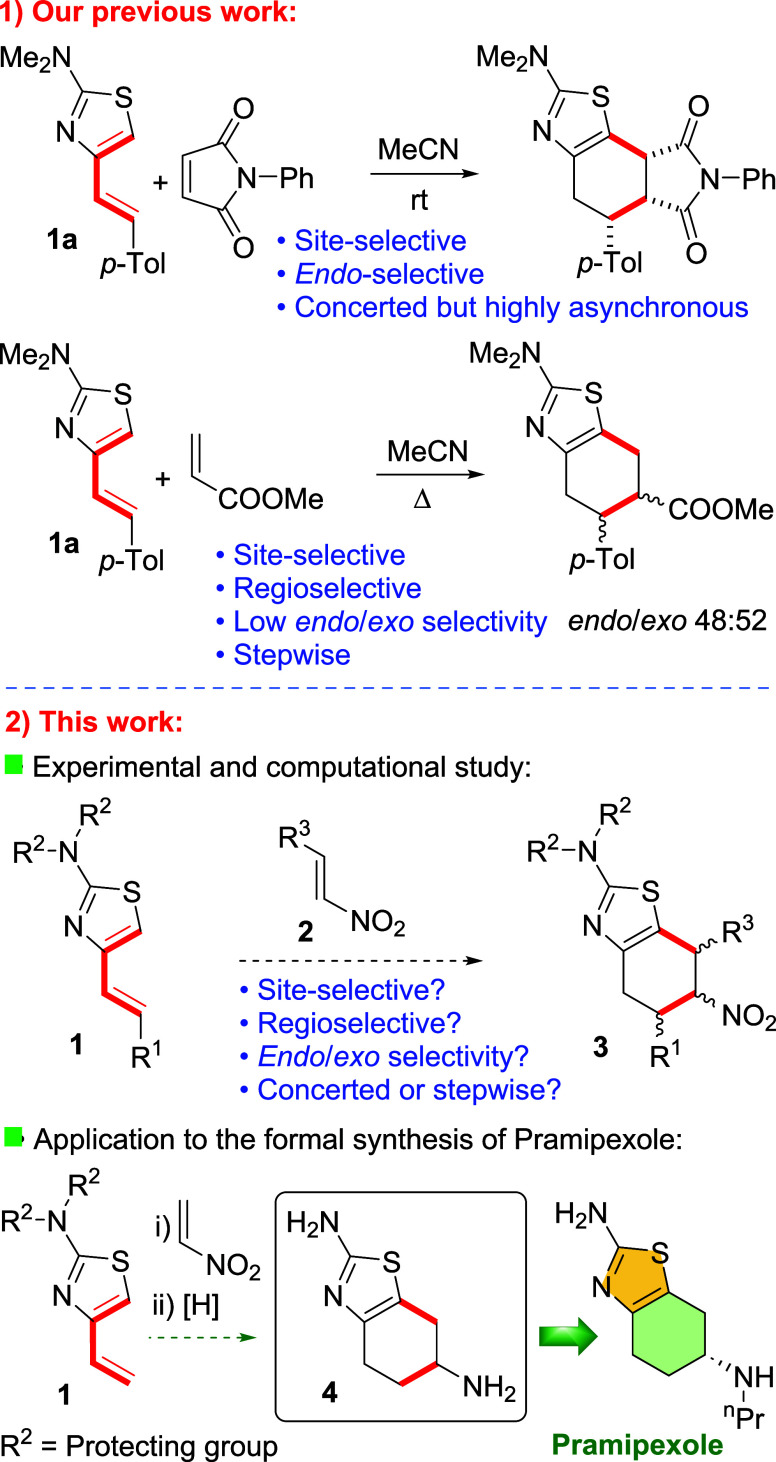
(1) 2-Amino-4-alkenylthiazoles Are Excellent In–Out Dienes in [4 + 2] Cycloadditions, although They Are Not *endo/exo* Selective with Methyl Acrylate; (2) Herein, We Describe [4 + 2] Cycloaddition Reactions of Substrates **1** with Nitroalkenes and Their Application to the Formal Synthesis of Pramipexole

The use of heteroaromatic compounds as either diene or dienophile has gained prominence in Diels–Alder chemistry.^1^ In this context, vinyl heterocycles as the dienic component show a notable preference for the in–out cycloaddition, which includes the side-chain double bond (*site selectivity*). Thus, diverse polycyclic structures have been synthesized in this way from vinylfurans,^2^ vinylindoles,^3^ vinylpyrroles,^4^ and vinylimidazoles.^5^ Although it was generally accepted that thiazoles showed unfavorable Diels–Alder reactions due to their considerable aromatic stabilization,^6^ we have demonstrated in a preceding work that 4-alkenylthiazoles also behave as excellent dienes, especially when an amino group is present at the 2-position of the thiazole ring.^7^ Thus, 2-amino-4-alkenylthiazoles react with dienophiles bearing electron-withdrawing groups under extremely mild conditions with excellent yields and high *endo* selectivity. However, the isolated products are not the initial [4 + 2]-cycloadducts but those resulting from a subsequent 1,3-hydrogen migration. These processes are exemplified in Scheme 1 by the reaction of **1a** with *N*-phenylmaleimide.^7c^ Curiously, a dramatic drop in the yield and *endo*/*exo* selectivity was observed when the same substrate (**1a**) was reacted with methyl acrylate, although this reaction takes place with high levels of regioselectivity (Scheme 1, part 1). These results were explained in terms of a concerted but highly asynchronous mechanism when using cyclic dienophiles, which turns out to be stepwise with acyclic ones.^7c^ Subsequently, we became interested in the [4 + 2] cycloaddition reactions of 2-amino-4-alkenylthiazoles (Scheme 1, part 2) with nitroalkenes (**2**), which could presumably lead to cycloadducts **3**. The use of nitroalkenes is a highly efficient way to control the regiochemistry of cycloaddition reactions.^8^ Moreover, the corresponding adduct of nitroethylene with the proper 2-amino-4-vinylthiazole could be easily transformed into 2,6-diamino-4,5,6,7-tetrahydrobenzothiazole (**4**) after deprotection and reduction of the nitro group (Scheme 1, part 2).

Compound **4** constitutes a key precursor of pramipexole,^9^ a potent drug used in the treatment of Parkinson’s disease.^10^ To address the synthesis of **4**, the amino group at the C-2 position of the thiazole ring needs to be protected since a free or partially substituted amino group would compete with the dienic system in its reaction with nitroethylene. Nevertheless, the nature of this protecting group is critical since the reactivity of substrates **1** is determined by the availability of the lone pair at this nitrogen atom to a great extent.^7c^ For that reason, we selected *N*,*N*-dibenzylamino- and *N*,*N*-bis(4-methoxybenzyl)amino-4-vinylthiazoles as substrates. In principle, these compounds would maintain their reactivity toward dienophiles and the amino protecting groups would be easy to remove.

Here, we disclose the results of a study of the [4 + 2] cycloaddition processes of 2-amino-4-alkenylthiazoles with (*E*)-β-nitrostyrene and nitroethylene from experimental and computational perspectives and include a new formal synthesis of pramipexole.

## Results and Discussion

### [4 + 2] Cycloaddition of **1a**–**d** with Nitroalkenes **2a**,**b**: Experimental Study

#### Synthesis of 4-Alkenyl-2-dialkylaminothiazoles **1b**–**d**

**Scheme 2:**
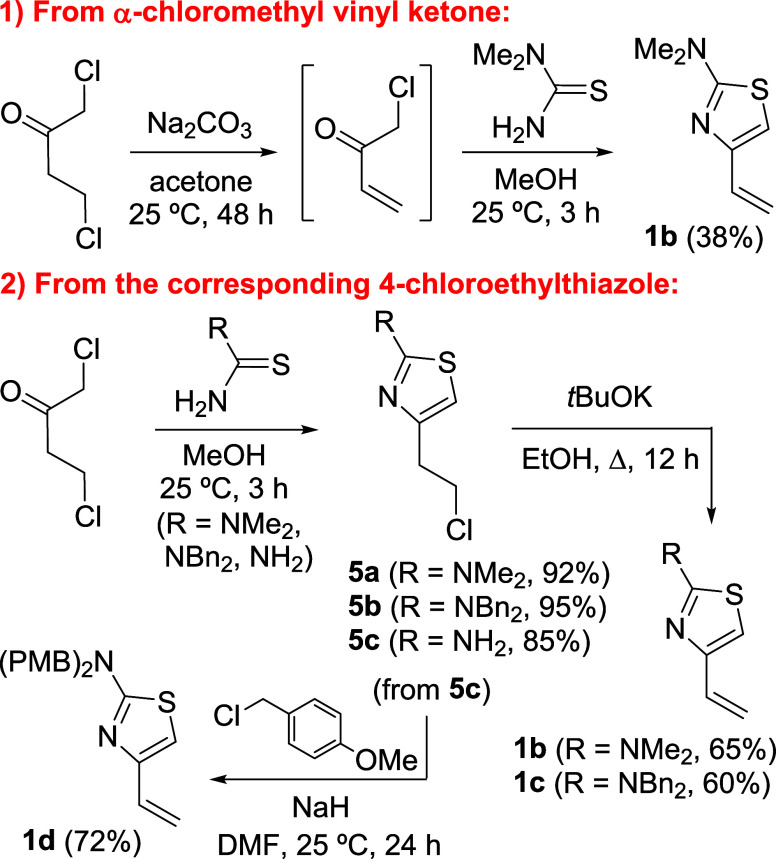
Synthesis of 4-Alkenyl-2-dialkylaminothiazoles **1b**–**d**

1-Chlorobut-3-en-2-one was obtained from 1,4-dichlorobutan-2-one after basic treatment (Scheme 2, part 1).^11^ α-Chloromethyl vinyl ketone was not isolated but reacted *in situ* with *N*,*N*-dimethylthiourea to give 2-dimethylamino-4-vinylthiazole **1b**.^12^ In view of the low yield obtained (38%), the synthetic route was slightly modified. Thus, 1,4-dichlorobutan-2-one was first reacted with *N*,*N*-dimethylthiourea in MeOH at 25 °C (Scheme 2, part 2). Under these conditions, 4-(2-chloroethyl)thiazole **5a** was isolated in an excellent yield (92%). Dehydrohalogenation of **5a** with potassium *tert*-butoxide led to **1b** in 65% of yield. An analogous sequence starting from *N*,*N*-dibenzylthiourea led to **5b** and subsequently to 2-dibenzylamino-4-vinylthiazole (**1c**) in good overall yield (Scheme 2, part 2). Compound **5c** was prepared, in turn, from 1,4-dichlorobutan-2-one and thiourea and isolated in 85% yield, whereas the synthesis of 2-[bis(4-methoxybenzyl)amino]-4-vinylthiazole (**1d**) was carried out from **5c**, by reaction with 4-methoxybenzyl chloride in the presence of a high excess of sodium hydride.

### [4 + 2] Cycloaddition Reactions of **1a**–**d** with Nitroalkenes **2a**,**b**

First, we tested the reaction of **1a**(13) with commercially available (*E*)-β-nitrostyrene (**2a**) in acetonitrile at 25 °C (Table 1, entry 1). Under these reaction conditions, tetrahydrobenzothiazole **3aa** was isolated in excellent yield (82%) as a single diastereomer. Compound **3aa** is the result of a [4 + 2] cycloaddition and a further 1,3-hydrogen migration leading to rearomatization of the thiazole ring. Conversely, the reaction of 2-dimethylamino-4-vinylthiazole **1b** with the same dienophile needs to be conducted under refluxing acetonitrile to give the cycloadduct **3ba** in lower yield (60%) in addition to small amounts of the Michael adduct **6ba** (14%, Table 1, entry 2). The higher reactivity of **1a** can be explained by considering a rise in the HOMO energy value^7c^ and will be discussed in more depth below. The reaction of **1b** with nitroethylene (**2b**)^14^ in acetonitrile at 25 °C led to a mixture of tetrahydrobenzothiazoles **3bb** and **7bb**, in poor yields (Table 1, entry 3).

**Table 1:**
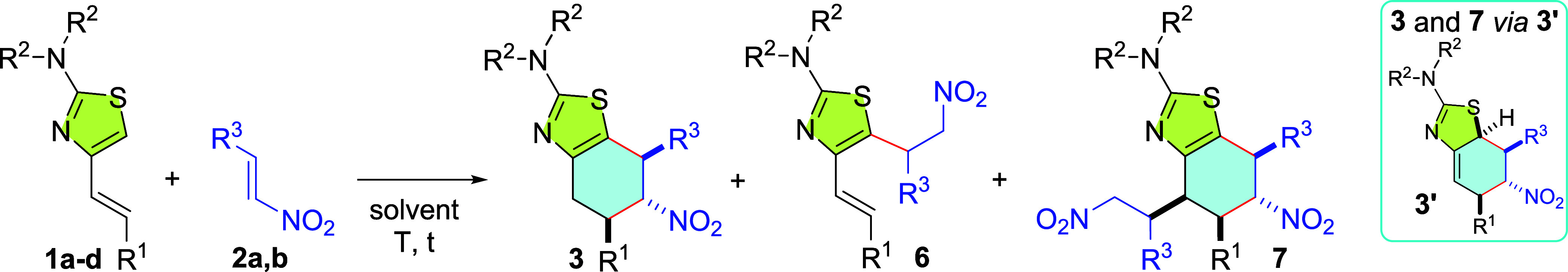

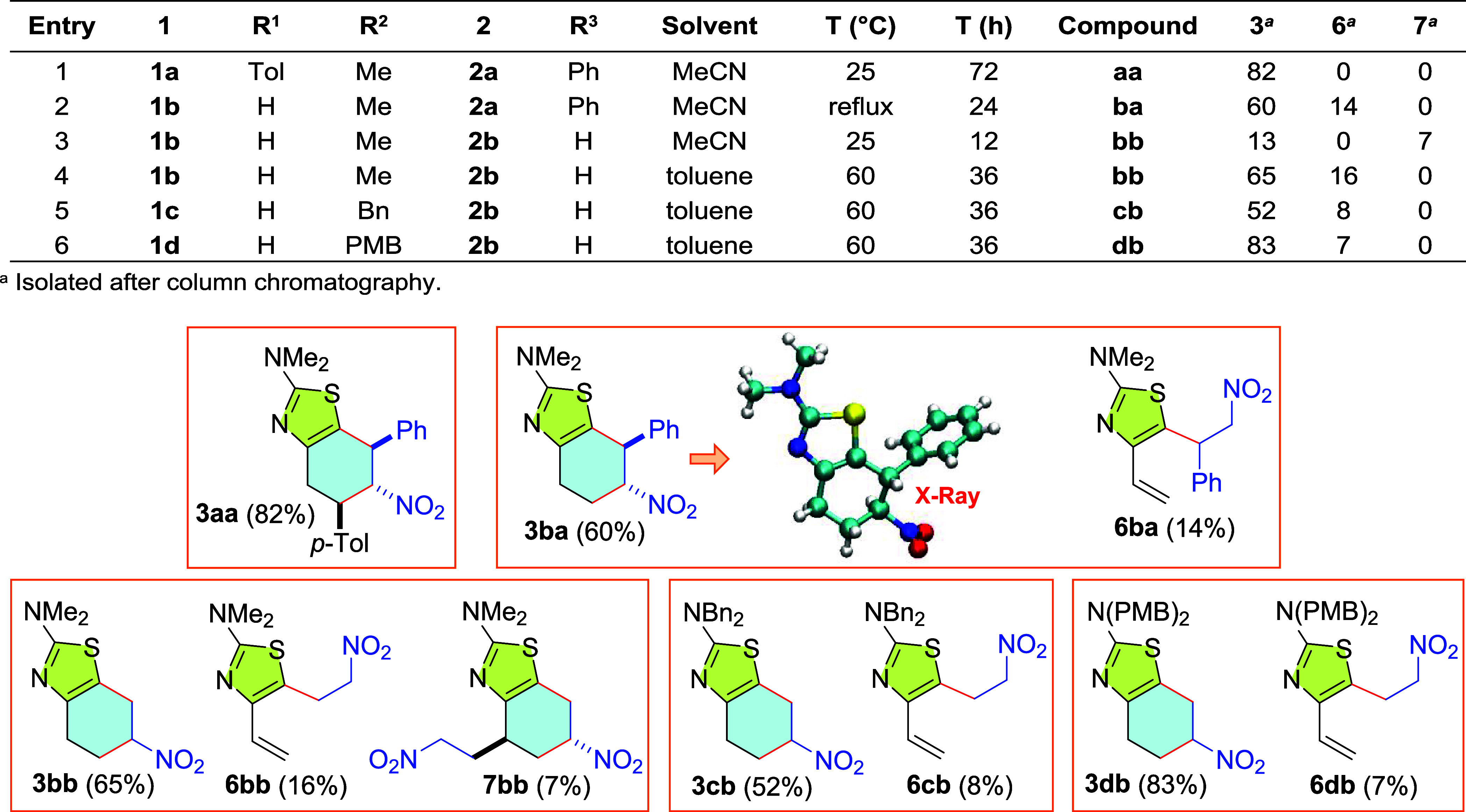
[4 + 2] Cycloaddition Reactions of 4-Alkenyl-2-dialkylaminothiazoles **1a**–**d** with Nitroalkenes **2a,b** Led to Tetrahydrobenzothiazoles **3** as Main Products^a^

a Michael adducts **6** or compounds **7**, resulting from the reaction with two molecules of nitroalkene, were isolated as secondary products. Framed box: Primary Diels–Alder adduct **3′**

b Isolated after column chromatography.

Compound **7bb** results from the primary Diels–Alder adduct **3**′ (at the top of the Table 1) by a further ene reaction with a second molecule of **2b**. This process is driven by the rearomatization of the thiazole ring. Under these conditions, a high amount of starting thiazole **1b** was recovered. The low conversion of **1b** by reaction with **2b** was probably caused by polymerization of nitroethylene within the reaction mixture, favored by the presence of a polar solvent such as acetonitrile. Accordingly, we conducted the reactions of **1b**–**d** with nitroethylene in toluene at 60 °C. Under these reaction conditions, cycloadducts **3bb**, **3cb**, and **3db** were isolated in good yields although with small amounts of the corresponding Michael adducts **6** (Table 1, entries 4–6). The relative stereochemistry of substituents on the six-membered ring in **3aa**, **3ba**, and **7bb** was established based on the values of the coupling constants between the protons placed at that ring and of significant cross-peaks in the ^1^H,^1^H-NOESY spectra (Supporting Information). The single-crystal X-ray structure determination of **3ba** confirmed the *trans* relative position of both the phenyl and nitro groups (Table 1).

From the above results, we can conclude that [4 + 2] cycloadditions of 4-alkenyl-2-dialkylaminothiazoles **1a**–**d** with nitroalkenes **2a**,**b** leading to **3** are not only site-selective (compounds **1** act as in–out dienes) but also completely regio- and diastereoselective.^15^ Our next objective was to use this cycloaddition between 4-alkenyl-2-aminothiazoles and nitroalkenes for the synthesis of a key precursor of pramipexole.

### New Formal Synthesis of Pramipexole

**Scheme 3:**
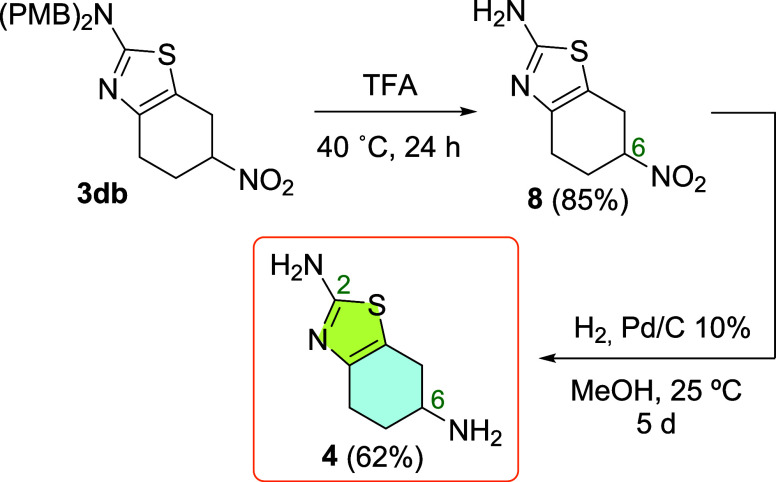
Synthesis of Pramipexole Precursor **4** from **3db**

Cycloadducts **3cb** and **3db** were selected as potential intermediates in the synthesis of 2,6-diamino-4,5,6,7-tetrahydrobenzothiazole **4** (Scheme 1, part 2). Our first attempt was the simultaneous deprotection of the amino functionality and the reduction of the nitro group by using **3cb** as a starting material. However, the deprotection did not take place after reacting **3cb** under a H_2_ atmosphere in the presence of Pd/C (10%) at room temperature or heating at 80 °C.^16^ We were unable to isolate **4** by treating **3cb** with ammonium formate either at room temperature or in refluxing methanol. The decomposition of **3cb** was observed when it was subjected to more severe reaction conditions, Pd/C (10%) under a H_2_-atmosphere (3 bar). Fortunately, the treatment of **3db** with trifluoroacetic acid at 40 °C led to 2-amino-6-nitro-4,5,6,7-tetrahydrobenzothiazole (**8**) in excellent yield (Scheme 3). Subsequent reduction of the nitro group in **8** with H_2_ in the presence of Pd/C gave **4** in a fair yield (Scheme 3). Optical resolution of racemic **4** and further derivatization steps were shown to convert **4** into enantiomerically enriched pramipexole.^9^ Interestingly, it has been recently reported that 4,5,6,7-tetrahydrobenzo[*d*]thiazole-2,6-diamine derivatives act as inhibitors of bacterial DNA gyrase B, an attractive target for antibacterial drug discovery.^17^

### Mechanistic Proposal for the [4 + 2] Cycloaddition of **1a**–**d** with (*E*)-β-Nitrostyrene (**2a**) and Nitroethylene (**2b**)

**Scheme 4:**
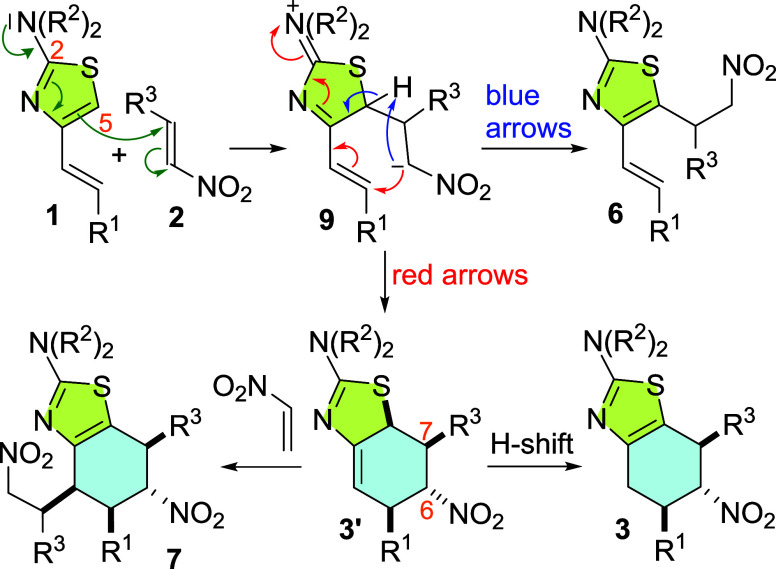
Mechanistic Proposal to Rationalize the Formation of Compounds **3**, **6**, and **7** from the Reaction of 4-Alkenyl-2-dialkylaminothiazoles **1a**–**d** with Nitroalkenes **2a**,**b**

A proposed explanation for the outcome of these processes is summarized in Scheme 4, which outlines a stepwise mechanism. Nucleophilic attack of the thiazole via C-5 to the most electron-deficient position of the nitroalkene would give zwitterionic intermediate **9**. This process would be assisted by the lone pair of the amino group at C-2 and by the stabilization of the negative charge by the nitro group, thus explaining the observed regioselectivity.^7c^ Zwitterion **9** might evolve in two alternative ways. An intermolecular 1,3-hydrogen migration would lead to the Michael adducts **6** (blue arrows) isolated in only low yields when the reactions are conducted at higher temperatures. Alternatively, the cyclization of **9** might lead to a formal [4 + 2]-cycloadduct **3′** (red arrows) with further evolution by an intermolecular 1,3-hydrogen migration, favored by the rearomatization of the thiazole ring. Finally, **3′** may experience an ene reaction with another molecule of nitroalkene leading to the tetrahydrobenzothiazole **7**.^7a^ While this proposal outlines the origin of products **3**, **6**, and **7**, there remains uncertainty regarding whether the Diels–Alder cycloadducts **3** can be formed with the high degrees of stereoselectivity experimentally observed when originating from intermediate **9**.^14,18^ This question will be addressed in the computational study.

In summary, our experimental work shows that the Diels–Alder cycloaddition between 4-alkenyl-2-aminothiazoles **1** and nitroalkenes **2** regioselectively produces the 6-NO_2_-substituted cycloadducts **3** due to the preferential orientation between both reactants. Furthermore, the configuration of the substituents within **3aa** indicates that only the NO_2_-*exo* diastereomer is obtained, *i.e.*, the reaction takes place with complete regio- and diastereoselectivity. Moreover, these processes are probably stereospecific because the *trans* orientation of both nitro and phenyl groups is retained from the (*E*)-β-nitrostyrene to the corresponding cycloadducts **3aa** and **3ba**.

### Computational Study

We next carried out an extensive computational study with the aim of gaining a more comprehensive understanding of the mechanism underlying this type of process as well as explaining the observed regio- and stereoselectivities.

**Scheme 5:**
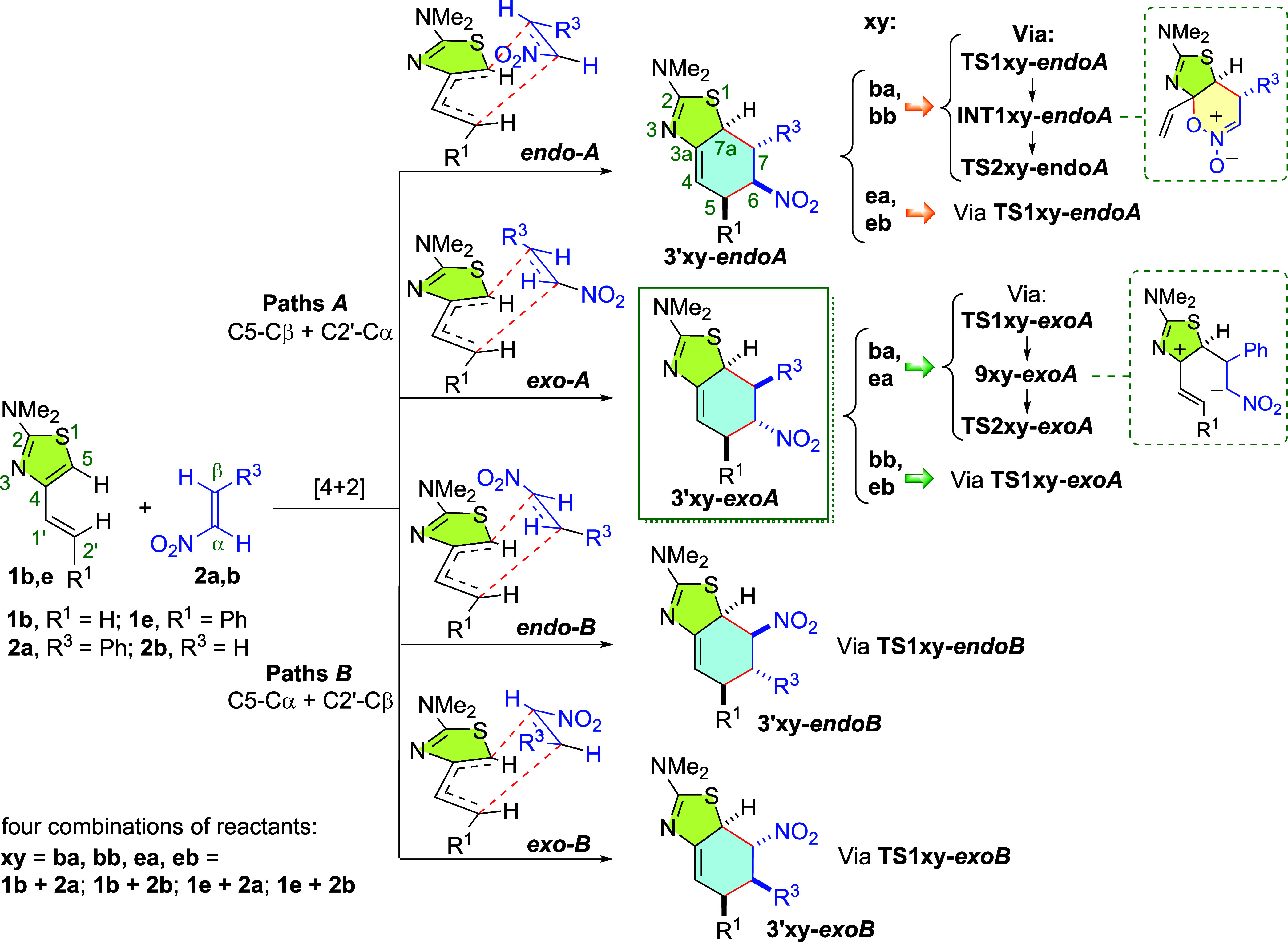
Alternative Mechanistic Pathways, Paths *A* (*endo-A* and *exo-A*) and Paths *B* (*endo-B* and *exo-B*), for the Diels–Alder Reaction of 4-Alkenyl-2-dimethylaminothiazoles **1b**,**e** with Nitroalkenes **2a**,**b**

We explored the potential energy surface at the ωB97X-D/6-31+G** theoretical level in toluene associated with the Diels–Alder cycloadditions of 4-alkenyl-2-dimethylaminothiazoles **1b**,**e** with nitroalkenes **2a**,**b**. There are four competing mechanistic pathways depending on how the diene and dienophile approach each other: ***endo-A***; ***exo-A***, ***endo-B***; and ***exo-B*** (Scheme 5). Paths *A* and *B* are regioisomeric with each other and lead to the respective 6- or 7-NO_2_-substituted cycloadducts. In paths *A*, the newly formed sigma bonds are C5(diene)-Cβ(nitroalkene) and C2′(diene)-Cα(nitroalkene), giving rise to **3′-*endoA*** and **3′-*exoA*** diastereoisomers. Paths *B* involve formation of C5–Cα and C2′-Cβ σ bonds and, in the same way, giving rise to **3′-*endoB*** and **3′-*exoB*** products. Indeed, these four alternative approaches could also be applied in the reaction of 2-dimethylamino-4-vinylthiazole with nitroethylene (the simplest diene–dienophile combination), but only two final regioisomers, not diastereoisomers, could then form. In a later step, cycloadducts **3′** experience an H-shift from C7a to C4, favored by the recovery of the aromaticity of the thiazole ring, leading to the final compounds **3**.^19^

As a result of our computational scrutiny, we discovered complex potential energy surfaces, including processes we had not anticipated, thus adding fascinating challenges to our study.

While for paths *B*, we found that the Diels–Alder cycloaddition takes place in a single kinetic step through the transition structures **TS1-*endoB*** and **TS1-*exoB***, for both possible pairing of dienes **1b**,**e** with dienophiles **2a**,**b**, for paths *A* we detected some cases with two-step mechanisms.

**Scheme 6:**
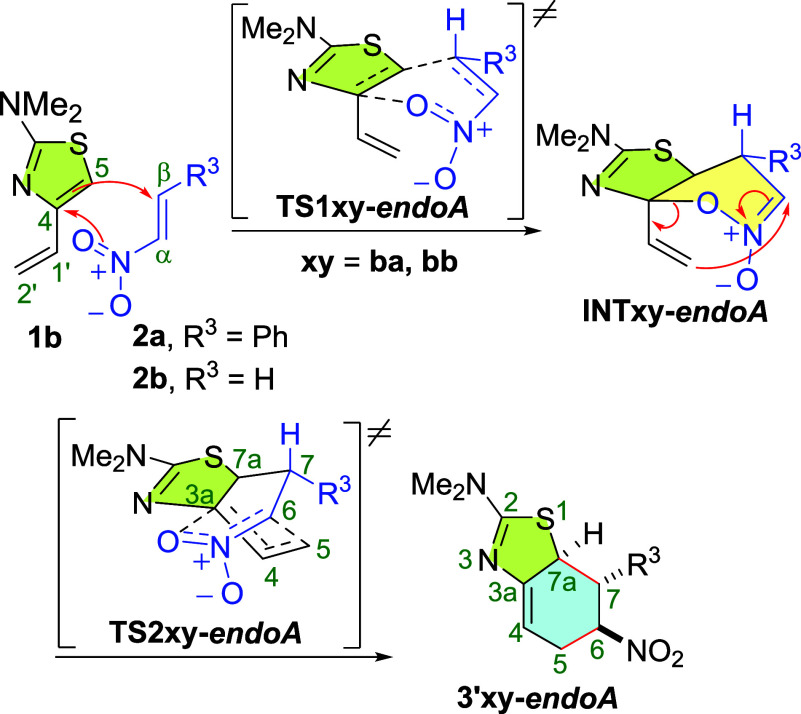
Tandem Hetero-Diels–Alder/Hetero-Claisen Processes Found in the Reaction of 2-Dimethylamino-4-vinylthiazole **1b** with Nitroalkenes **2a**,**b** via the *endo-A* Path

Curiously, the reaction of vinylthiazole **1b** with nitroalkenes **2a** or **2b** via the *endo-A* channel involves tandem Diels–Alder/hetero-Claisen processes. The first step leads to the formation of the nitronate ester cycloadducts **INTba-*endoA*** and **INTbb-*endoA*** through transition structures **TS1xy-*endoA*** (**xy** = **ba**, **bb**). These intermediates are the result of a Diels–Alder reaction in which the nitroalkene acts as a diene by involving the alkenyl C=C and one of its N–O bonds as a 4π component, while the C4=C5 bond of the thiazole ring acts as a dienophile (Scheme 6). These intermediates are thermodynamically much less stable than the **3′xy-*endoA*** cycloadducts and, in a fast second step, undergo a peculiar hetero-Claisen rearrangement through the transition structures **TS2xy-*endoA***, in which cleavage of the C–O bond and formation of the C5–C6 bond takes place simultaneously converting the 1,2-oxazinium ring into a nitro-substituted-cyclohexene one. This behavior is not observed in the reactions of 2-dimethylamino-(*E*)-4-styrylthiazole **1e** with the same nitroalkenes, but the IRC path toward the products shows a flat region with structures quite similar to **INTxy-** and **TS2xy-*endoA*** (**xy** = **ba**, **bb**) (see the Supporting Information for IRC plots).

The significance of nitroalkenes as heterodienes has gained substantial attention in documented studies.^20^ In some instances, the resulting nitronate ester cycloadducts display a propensity for diverse subsequent reactions.^20a,20d,20g,20h^

An intriguing sequence encompasses a tandem [4 + 2] cycloaddition/[3,3] sigmatropic rearrangement, ultimately leading to a cycloadduct that appears to be the result of a formal [4 + 2] cycloaddition where the nitroalkene has apparently acted as a dienophile.^20g^ This peculiar series of processes characterizes the reaction of vinylthiazole **1b** with nitroalkenes **2a** or **2b** by the endo-*A* pathway (Scheme 6).

**Scheme 7:**
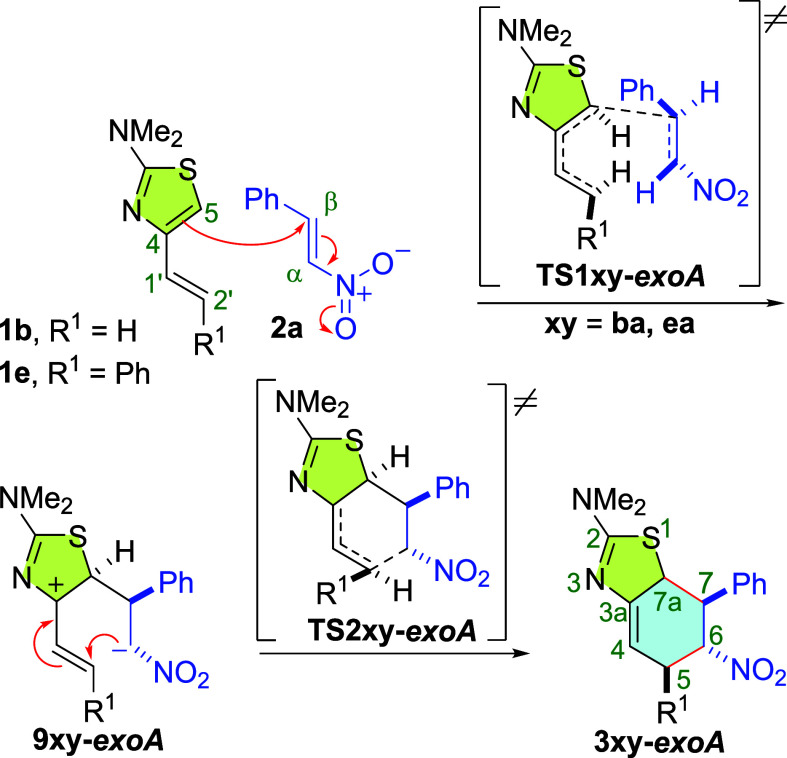
Two-Step Mechanism Initiated by Michael Addition Found for the Reaction of 4-Alkenyl-2-dimethylaminothiazoles **1b**,**e** with (*E*)-β-Nitrostyrene **2a** via the *exo-A* Path

Another peculiarity is observed in the *exo-A* path for the reactions of the thiazoles **1b**,**e** with the (*E*)-β-nitrostyrene dienophile **2a**. They also occur by a two-step mechanism, but the first step leads to the zwitterions **9xy-*exoA*** via **TS1xy-*****exoA*** (**xy** = **ba**, **ea**), as a result of a Michael addition of the nucleophilic C5 atom of the thiazole nucleus to the nitrostyrene. The formation of the second σ bond, C2′-Cα (or C5–C6 in the cycloadduct), takes place through **TS2xy-*****exoA*** in the following kinetic step (Scheme 7).

**Figure 1:**
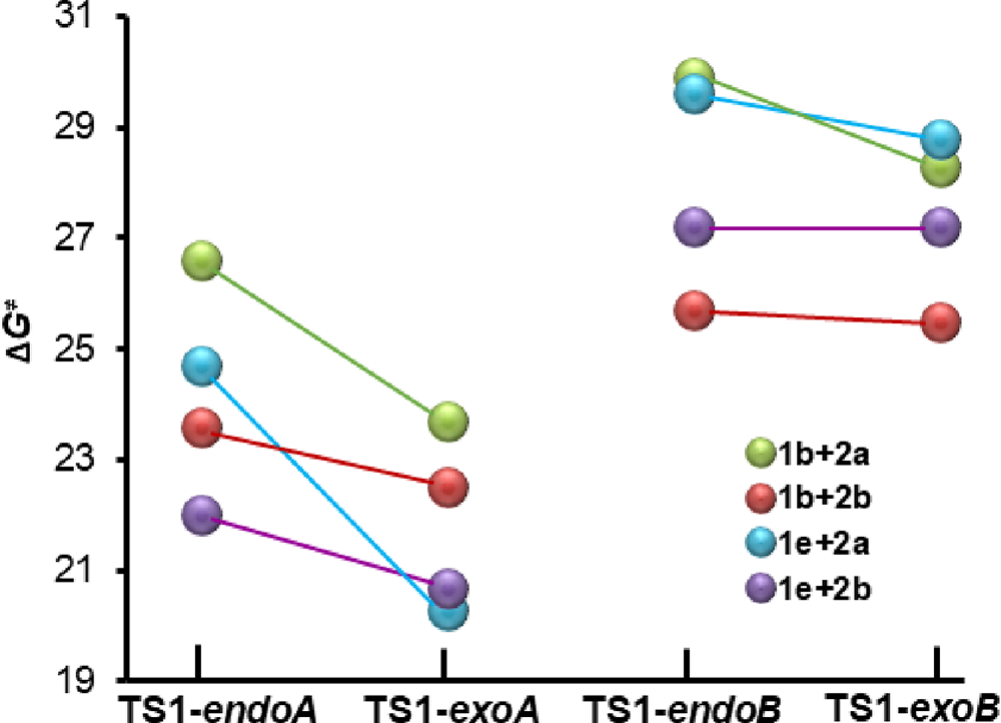
Plot of the ωB97X-D/6-31+G** Gibbs free energy barriers (in kcal·mol^–1^) computed at 298 K in toluene for the Diels–Alder reaction of **1b**,**e** with **2a**,**b** through the transition structures **TS1xy** corresponding to the *endo-A*, *exo-A*, *endo-B*, and *exo-B* paths.

The relative Gibbs free energies of each stationary point of the Diels–Alder reactions of **1b**,**e** with **2a**,**b** are collected in Table 2, whereas Figure 1 shows the energy barriers through transition structures **TS1xy** of each alternative mechanistic path and each diene–dienophile combination. As shown in Figure 1, the energy barriers calculated for pathways *A* leading to **3′xy-*endoA*** and **3′xy-*****exoA*** are always lower than those corresponding to paths *B* (Δ*G*^⧧^ ranges for paths *A* and *B*: 20.3–26.6 *vs* 25.5–29.9 kcal·mol^–1^), with the reaction of 2-dimethylamino-4-vinylthiazole (**1b**) with nitroethylene (**2b**) presenting a minor difference, and therefore a smaller preference for pathway *A*.

**Table 2:** ωB97X-D/6-31+G** Relative Gibbs Free Energies in kcal·mol^–1^, Computed at 298 K in Toluene, of the Stationary Points Involved in the Diels–Alder Reaction of 4-Alkenyl-2-dimethylaminothiazoles **1b**,**e** with Nitroethylenes **2a**,**b**

path		**1b**+**2a**	**1b**+**2b**	**1e**+**2a**	**1e**+**2b**
endo-A	**TS1xy-*endoA***	26.6	23.6	24.7	22.0
	**INTxy-*endoA***	10.8	5.4		
	**TS2xy-*endoA***	22.5	17.5		
	**3′xy-*endoA***	–18.1	–22.6	–11.4	–16.1
	**3xy-*endoA***	–30.8	–34.7	–24.1	–28.6
exo-A	**TS1xy-*exoA***	23.7	22.5	20.3	20.7
	**9xy-*exoA***	21.1		16.4	
	**TS2xy-*exoA***	21.8		a	
	**3′xy-*exoA***	–18.3	–24.8	–13.1	–19.8
	**3xy-*exoA***	–31.0	–36.2	–26.8	–31.2
endo-B	**TS1xy-*endoB***	29.9	25.7	29.6	27.2
	**3′xy-*endoB***	–14.9	–20.0	–11.4	–13.8
	**3xy-*endoB***	–25.1	–34.5	–25.4	–30.0
exo-B	**TS1xy-*exoB***	28.3	25.5	28.8	27.2
	**3′xy-*exoB***	–17.0	–22.9	–12.6	–18.1
	**3xy-*exoB***	–29.1	–35.7	–25.5	–31.5

a We have not been able to optimize this transition state.

When comparing the *endovsexo* approaches of the nitro group, it is remarkable that *exo*-transition states are always lower in energy than their alternative *endo* counterparts. In general, the preference for the *exo* orientation is higher for paths *A* (ΔΔ*G*^⧧^_*exo-endo*_ ranges from 1.1 to 4.4 kcal·mol^–1^) than for paths *B* (ΔΔ*G*^⧧^_*exo-endo*_ oscillates between 0 and 1.6 kcal·mol^–1^).

Interestingly, the most pronounced preference for the *exo* approach is more evident in pathway *A* when the dienophile is (*E*)-β-nitrostyrene (**2a**). Therefore, the incorporation of a phenyl ring into the dienophile has a highly advantageous impact on the *exoA* transition structures.

Our theoretical study predicts that the conversion of reactants into Diels–Alder cycloadducts via transition structures **TS1xy-*endoB*** will be the slowest for all combinations of diene–dienophile (except for **1e** with **2b** since **TS1eb-*endoB*** and **-*exoB*** have the same energy). On the contrary, the *exoA* paths will always be the fastest. Moreover, among the four combinations, the reaction of **1e** with **2a** is the fastest one. This combination of reactants also shows the largest energy difference between **TS1ea-*endoA*** and **TS1ea-*exoA*** (ΔΔ*G*^⧧^_*exo-endo*_ = 4.4 kcal·mol^–1^).

In cases in which the reaction takes place by a two-step mechanism (Schemes 6 and 7), the energy barrier corresponding to the second kinetic step is small and much lower than that of the previous one, as shown by the values of the energy barriers involving **TS2ba-** and **TS2bb-*endoA***, 11.7 and 12.1 kcal·mol^–1^, respectively (see the Supporting Information, Figure S1). Even smaller are those involving **TS2ba-** and **TS2ea-*exoA***, which can be considered nonexistent in practice.

Even though the preferred path, *exo-A*, is predicted to occur in a stepwise manner when the dienophile is **2a**, the half-life of intermediates **9** should be so small that no loss of stereoselectivity is observed in the final cycloadducts. The existence of these intermediates also provides a rationale for the experimentally observed formation of Michael adduct **6** in some cases. On the other hand, if we consider the final cycloadducts **3xy**, these processes are highly exergonic and the ***exo-A*** cycloadducts are invariably predicted to be the thermodynamically controlled products.

In sum, paths *exo-A* are anticipated to be the preferred in all cases under consideration, aligning with the findings of the experimental study. After the analysis of the mechanistic trajectories and the energy profiles, we next present the results obtained from various analytical tools employed to elucidate the structural and electronic characteristics of these cycloadditions. These insights, in turn, aid in understanding the reactivity of the diene and dienophile and the stereoselectivity of the reaction steps.

### Geometry of Transition Structures

**Figure 2:**
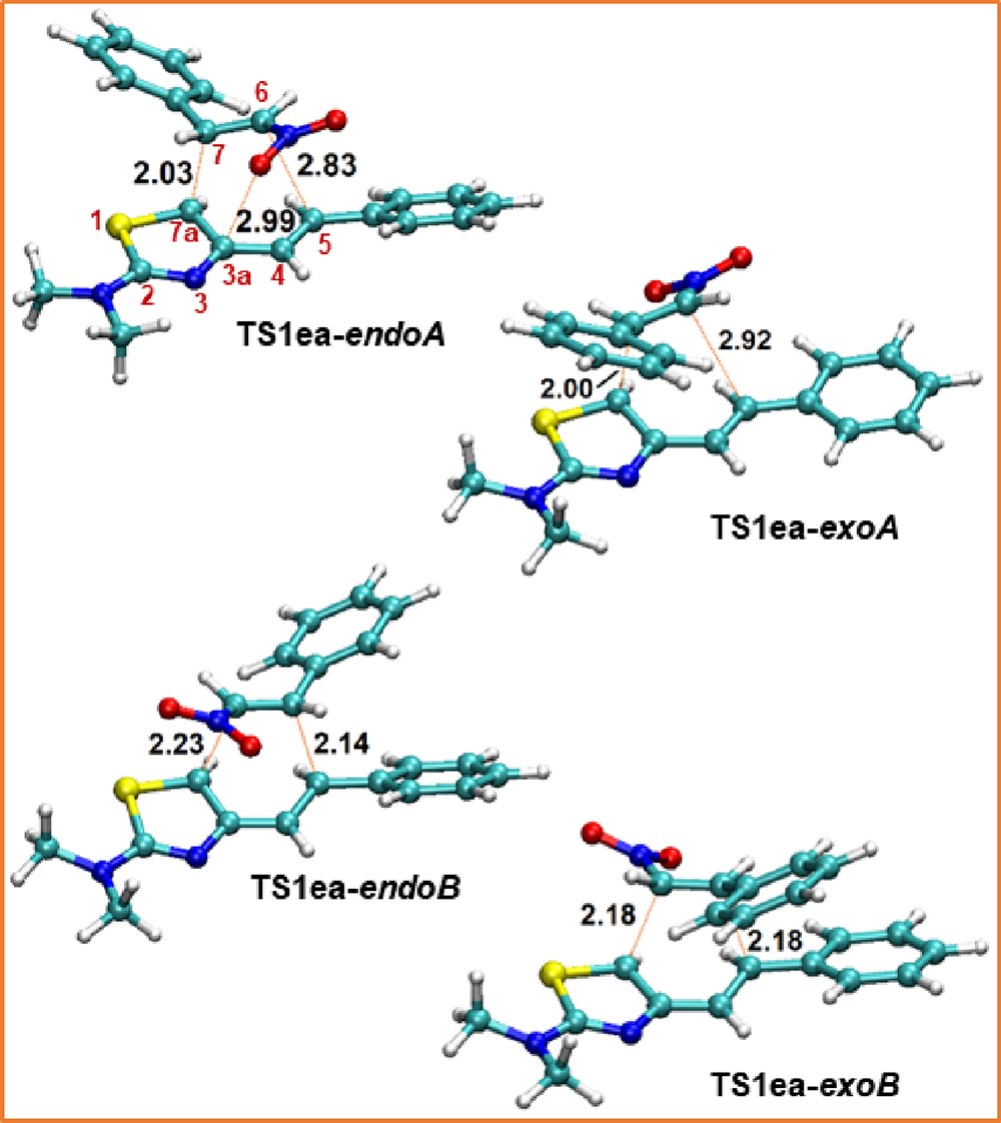
ωB97X-D/6-31+G** optimized transition structures corresponding to the Diels–Alder reaction of 2-dimethylamino-(*E*)-4-styrylthiazole (**1e**) with (*E*)-β-nitrostyrene **2a** through alternative paths.

The geometries of transition structures corresponding to each path for the reaction of 2-dimethylamino-(*E*)-4-styrylthiazole **1e** with (*E*)-β-nitrostyrene (**2a**) are disclosed in Figure 2.

The transition structures **TS1xy-*endoA*** exhibit very similar geometries, regardless of whether the reaction occurs in one or two steps. Likewise, all **TS1xy-*exoA*** species are geometrically comparable. In addition, if the transformation takes place in a single step, as is the case of **1e** + **2a** → **3′ea-*****endoA*** and **1e** + **2b** → **3′eb**-***endoA***, a shoulder is clearly distinguished on the IRC paths downhill from **TS1ea**- and **TS1eb-*endoA*** toward the respective Diels–Alder cycloadducts, whose highest point in energy is geometrically analogous to the second transition state when the reaction occurs in two mechanistic steps. In the same way, the IRC pathways downhill from **TS1bb-** and **TS1eb-*****exoA*** to the cycloadducts also cross a small hill, and the structure of their highest energy point is structurally like the second transition structure when the reaction involves two kinetic steps. For these reasons, and for the sake of simplicity, we have used the same notation **TS1xy** for all reactions concerted or not.

The lengths of the σ bonds that are being formed allow us to establish that the TSs of paths *A* are very asynchronous (see Figure 2 and Table 3), with the forming σ bond between the C7a atom of the thiazole ring and the σ carbon of the nitroalkene, C7a-C7 in Figure 2, being the most advanced. As depicted in Table 3, the length of this bond ranges from 1.95 to 2.06 Å, notably shorter than that measured for the C5–C6 forming a σ bond, which varies between 2.83 and 2.96 Å. On the contrary, the C5–C6 bond is the most advanced in the TSs of *B* pathways.

**Table 3:** Lengths of Forming σ-Bonds (*d*, in Å) and Difference between Bond Forming Distances (Δ*d*, in Å) at Transition States **TS1xy** Found for the Diels–Alder Cycloaddition of **1b**,**e** with the **2a**,**b** Optimized at the ωB97X-D/6-31+G** Theoretical Level in Toluene

	*d*C7a-C7/*d*C5–C6, Δ*d*		*d*C7a-C7/*d*C5–C6, Δ*d*	
**TS1xy**	***endoA***	***exoA***	***endoB***	***exoB***
**1b**+**2a**	2.00/2.91, 0.91	1.95/2.93, 0.97	2.64/1.96, 0.67	2.58/1.96, 0.62
**1b**+**2b**	2.02/2.96, 0.94	2.01/2.94, 0.92	2.77/1.97, 0.80	2.71/1.97, 0.74
**1e**+**2a**	2.02/2.83, 0.80	2.00/2.92, 0.92	2.23/2.14, 0.09	2.18/2.18, 0.00
**1e**+**2b**	2.06/2.91, 0.86	2.05/2.94, 0.88	2.60/1.96, 0.64	2.56/1.96, 0.60

Another difference between the TSs of paths *A* and *B* is that the latter shows much less asynchronicity. The higher asynchrony of the transition states of paths *A* can be easily seen by the difference between the lengths of the two σ-bonds being formed, Δ*d* (Table 3).

For the former, the Δ*d* range is 0.80–0.97, and the most asynchronous TSs belong to the cycloadditions that take place via a two-step mechanism, except **TS1bb-*ex******oA***, which, despite its large Δ*d* (0.92), corresponds to a one-step process, as revealed by the IRC calculations. In contrast, for the TSs of paths *B*, the Δ*d* values vary between 0.00 and 0.80 Å.

**Figure 3:**
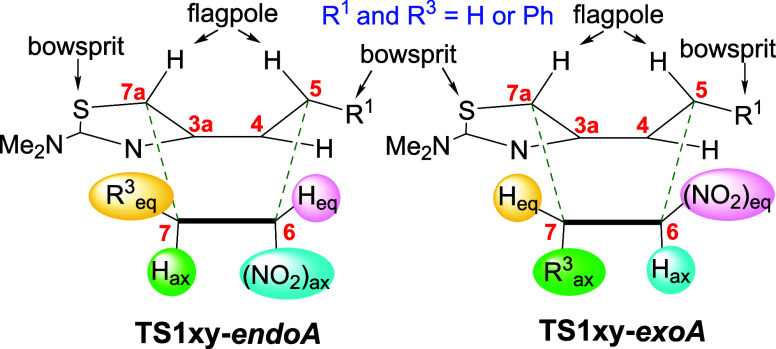
Two views of the mean boat-like geometry of transition states **TS1-*endoA*** and **-*exoA*** showing the flagpole and bowsprit substituents and the boat-axial (B_ax_) and -equatorial (B_eq_) positions.

Since paths *B* cannot energetically compete with pathways *A*, and with the aim to simplify the discussion, in the following paragraphs, we will only compare transition structures **TS1xy** belonging to paths *A*. In these transition structures, the six-membered forming ring shows a boat-like conformation (see Figure 3). Given the structure and stereochemistry of the diene, in all TSs, the flagpole positions are those of hydrogen atoms. Undoubtedly, this fact presents an advantage, as there are no significant repulsions arising from flagpole steric interactions. One of the two bowsprit locations, at C7a, is invariably occupied by the sulfur atom of the thiazole ring, whereas the other one, at C5, can be H or a phenyl ring depending on whether the diene is **1b** or **1e**, respectively (R^1^ in Figure 3). Some of the TSs exhibit a deformation of this mean conformation due to the twist of the C6–C7 bond (originally the C=C double bond of the dienophile). This distortion probably relieves a significant portion of the van der Waals strain, and it is particularly evident in **TS1xy-*exoA***.

Concerning the position of the other substituents in the forming ring, the nitro group is placed at C6 at the boat-axial position if the approach is *endo* or at the boat-equatorial position if it is *exo*. When the dienophile is **2a**, its phenyl ring is attached to C7. In the *endo* transition states, it is located at the equatorial position, whereas the *exo* transition states place it at the axial position.

Of note, in the TSs *exoA*, the phenyl ring is oriented toward the 2-dimethylamino-1,3-thiazole fragment, which could allow noncovalent interactions between both moieties.^21^ The inclusion of a phenyl ring at C7 increases the energy of the TSs of path *endo-A*, by approximately 3 kcal·mol^–1^, whereas this destabilization is lower or nonexistent in path *exo-A*. On the other hand, the attachment of a Ph ring at the bowsprit position of C5 (*i.e.*, when the diene is **1e**) has in all cases a stabilizing effect, which is larger in the *exoA* transition structures.

The stabilizing effect of positioning *exo* the nitro group is evident by comparing the energy of **TS1bb-*endoA*** with **TS1bb-*exoA*** (23.6 and 22.5 kcal·mol^–1^, respectively), which correspond to the simplest diene–dienophile cycloaddition, **1b** + **2b**. The preference for *exo* transition states is observed in all cases, and this effect is higher when the dienophile is **2a** (R^3^ = Ph) as shown by the decrease in energy from **TS1ba-*endoA*** to **TS1ba-*exoA*** and from **TS1ea-*endoA*** to **TS1ea-*exoA*** (differences of 2.9 and 4.4 kcal·mol^–1^, respectively). Note that in these *exoA* TSs, the phenyl ring at C7 is *endo*.^22,23^

### Noncovalent Interactions

To gain insight into the marked preference for the *exo-A* path, we have also performed a topological analysis of noncovalent interactions^23b,24^ (NCIs) in all transition structures **TS1xy** (Supporting Information). Here, we comment on the significant observations for the transition states of paths A.

**Figure 4:**
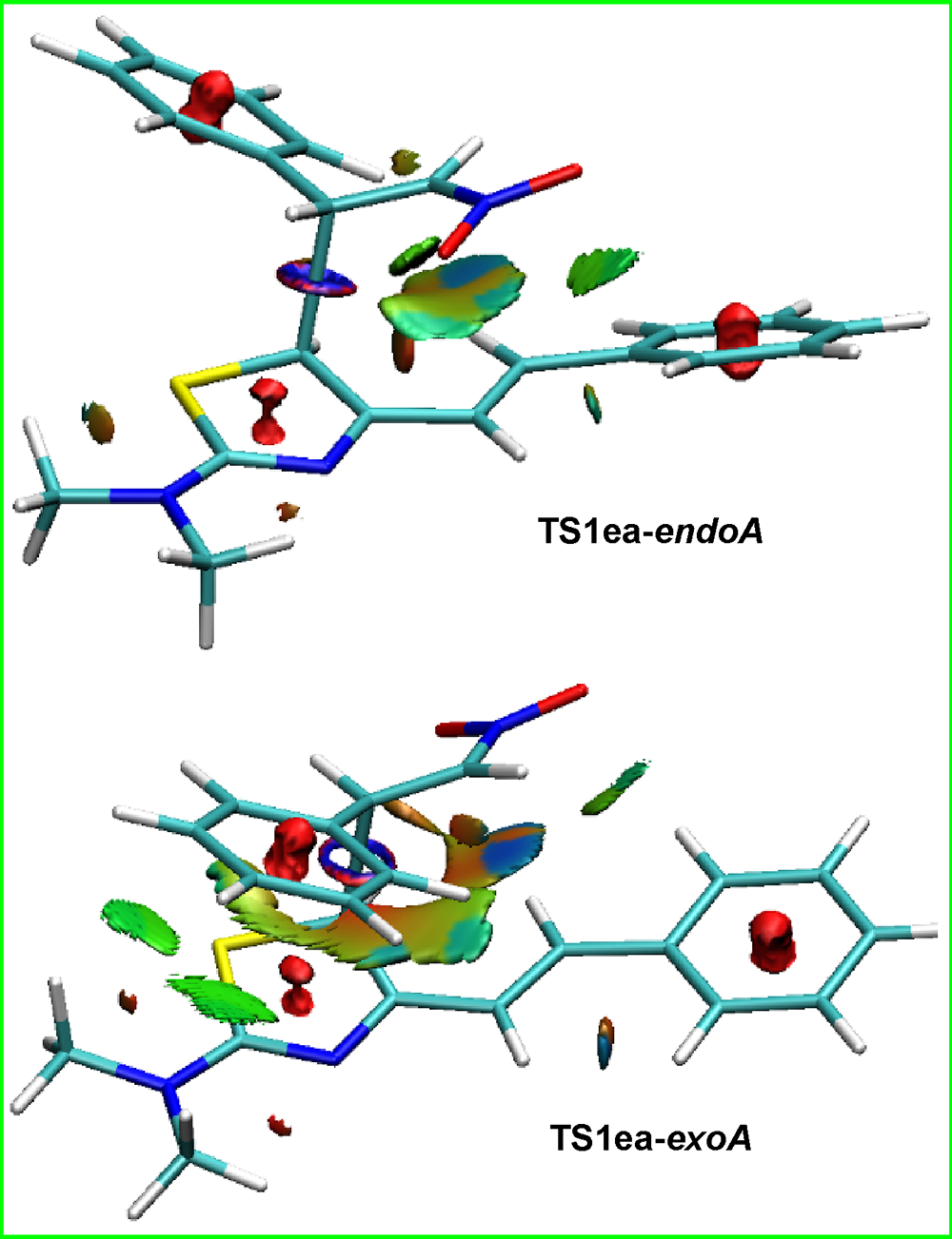
NCI isosurfaces of 0.3 associated with the density overlap at **TS1ea**-***endoA*** (top) and **TS1ea-***exoA* (bottom) and blue (hydrogen bonds), green (van der Waals interactions), and red (steric crowding) color scales.

A relevant finding refers to the larger stabilization of TSs *exoA* with respect to their *endoA* counterparts when the dienophile is **2a**. This fact can be explained in part by the presence of an extended green surface between the phenyl ring at C7, with both the dimethylamino group and the thiazole ring in the species **TS1ba**- and **TS1ea-*exoA*** (see Figure 4), whereas these weak favorable interactions are not present in **TS1ba**- and **TS1ea-*endoA***.

Furthermore, a significant amount of the stabilizing effect observed when a phenyl ring is attached at the bowsprit position of C5 (**1e**) could be attributed to the favorable interaction between the phenyl ring and the nitro group, which can be visualized in both *endoA* and *exoA* transition structures of Figure 4. Besides, within the *exo* transition structures, one can observe a subtle green–blue surface situated between an oxygen atom of the nitro group and a hydrogen at C7a. This may be attributed to the formation of a weak hydrogen bond between these two atoms.

Recapping, the analysis of geometries and NCI interactions aligns with the tendency to position the nitro group *exo*. This orientation is primarily attributed to more favorable electronic and thermodynamic factors in this pathway, while also being influenced by the balance between steric and electrostatic noncovalent interactions.

### Analysis of the Conceptual DFT (CDFT) Indexes

#### Reactivity Indexes at the Ground States of Reagents

We have also computed conceptual DFT indexes based on changes in the electron density.^25^ Thanks to the work of Domingo^26^ and Miranda-Qintana et al.,^27^ these reactivity descriptors have become powerful tools of quantum chemistry for understanding and predicting chemical reactivity and they have been successfully used to envisage the polar character of cycloaddition reactions as well as their feasibility.^27,28^

First, we computed a series of reactivity indexes such as HOMO and LUMO energies; electronic chemical potential, μ; chemical hardness, η; global electrophilicity, ω; and global nucleophilicity, *N*, at the ground state of the reactants 4-alkenyl-2-dimethylaminothiazoles **1b**,**e** and nitroalkenes **2a**,**b**. The results are collected in Table 4.

**Table 4:** HOMO and LUMO Energies (Hartrees), Electronic Chemical Potential, μ; Chemical Hardness, η, Global Electrophilicity, ω; and Global Nucleophilicity, *N*, of **1b**,**e** and **2a**,**b** at B3LYP/6-31G* Computed on the Optimized Geometries at the Same Theoretical Level^a^

	*E*_HOMO_ (au)	*E*_LUMO_ (au)	μ (eV)	η (eV)	ω (eV)	*N* (eV)
**1b**	–0.1899	–0.0226	–2.89	4.56	0.92	3.95
**1e**	–0.1843	–0.0439	–3.10	3.82	1.26	4.11
**2a**	–0.2553	–0.0967	–4.79	4.31	2.66	2.17
**2b**	–0.2958	–0.0957	–5.33	5.45	2.61	1.07

a We have computed the CDFT reactivity indexes at the B3LYP/6-31G* theoretical level since the electrophilicity and nucleophilicity scales were calculated and reported at this level of theory.^26b^ During the writing of this manuscript, a study on these scales at different theoretical methods was published.^29^

The electron chemical potential, μ, is associated with the propensity of a chemical species in its ground state to exchange electron density with its environment. The comparison of μ values allows for a prediction of the direction of electron density flux from the less electronegative reactant toward the more electronegative one. Since the values computed for the 4-akenylthiazoles **1b**,**e** (μ = −2.89 and −3.10 eV, respectively) are greater than those of nitroalkenes **2a**,**b** (μ = −4.79 and −5.33 eV, respectively), we establish that throughout these Diels–Alder reactions, the electron density will flow from the thiazoles to the nitroalkenes. Furthermore, the magnitude of the difference in the chemical potentials between dienes **1b**,**e** and dienophiles **2a**,**b** (Δμ), ensures that these transformations will have a marked polar character: Δμ_**1b**-**2a**_ = 1.90, Δμ_**1b**-**2b**_ = 2.44, and Δμ_**1e**-**2a**_ = 1.68, Δμ_**1e**-**2b**_ = 2.22, with the combination **1b** + **2b** presenting the greatest difference.

By comparing the chemical hardness, it can be inferred that 4-styrylthiazole **1e** (η = 3.82 eV) is softer and more sensitive to perturbations than **1b** (η = 4.56 eV) and also than nitro compounds **2a**,**b** (η = 4.31 and 5.45 eV, respectively).

In relation to the electrophilicity (ω) and nucleophilicity indexes (*N*), both nitroalkenes are classified as strong electrophiles within the electrophilicity scale,^26b,30^ presenting similar ω values (2.66 and 2.61 eV for **2a** and **2b**, respectively).

On the other hand, both thiazoles, **1b** and **1d**, show high *N* values (*N* = 4.11 and 3.95 eV for **1b** and **1d**, respectively), which allow us to classify them as strong nucleophiles.

Furthermore, to better understand the relative reactivity of these in–out dienes, along with the influence exerted by the dialkylamino group on its C2 atom, we have also computed the HOMO values and the nucleophilicity index for 4-alkenylthiazoles **10b**,**e**, for the Danishefsky–Kitahara (**11a**) and Rawal (**11b**) dienes, which are known for their great ability to behave as activated dienes in Diels–Alder reactions, and for (*E*)-1-dimethylamino-1,3-butadiene (**12**). These results are given in Table 5.

**Table 5:**
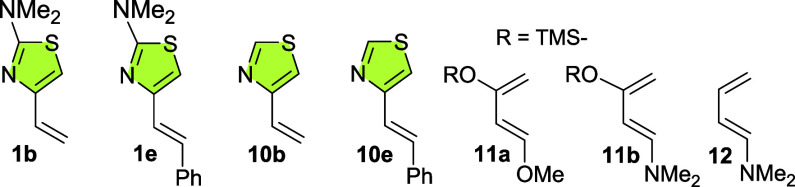
HOMO Energy (Hartrees), Electronic Chemical Potential, μ (eV), and Global Nucleophilicity, *N* (eV), of 4-Alkenylthiazoles **1b**,**e** and **10b**,**e**, Danishefsky–Kitahara Diene (**11a**), Rawal Diene (**11b**), and (*E*)-1-Dimethylamino-1,3-butadiene **12** at B3LYP/6-31G* Computed on the Optimized Geometries at the Same Theoretical Level

	**1b**	**1e**	**10b**	**10e**	**11a**	**11b**	**12**
***E***_**HO**_	–0.19	–0.18	–0.23	–0.21	–0.20	–0.18	–0.18
**μ**	–2.89	–3.10	–3.64	–3.53	–2.66	–2.27	–2.36
***N***	3.95	4.11	2.97	3.53	3.72	4.33	4.31

The values of nucleophilicity of **1b**,**e** and **10e** are comparable to those computed for dienes **11a**,**b** and **12**, demonstrating that they are strong nucleophiles accordingly to the nucleophilicity scale. 4-Vinylthiazole **10b** should be classified as a moderate nucleophile, as it shows a very similar *N* value to 1,3-butadiene.^26b^ The decreasing order of the nucleophilicity indexes of the considered species, *N*, is **11b** > **12** > **1e** > **1b** > **11a** > **10e** > **10b**.

Undoubtedly, in view of these theoretical and experimental studies, the 1,3-thiazole ring has a very beneficial effect on the activation of these species as dienes and, which makes them even more valuable, on the observed regioselectivity, as we will comment below. The inclusion of the dimethylamino group at its 2-position clearly increases the nucleophilicity of the diene, as evidenced by the higher *N* values of **1b** and **1e** compared with those of their nonsubstituted analogues (3.95 and 4.11 for **1b** and **1e**, respectively, *vs* 2.97 and 3.53 for **10b** and **10e** in this order). Attachment of a phenyl ring at the C2′ carbon of the exocyclic double bond also enhances nucleophilicity (compare *N* values for **1b** vs **1e**, as well as for **10b** vs **10e**).

**Figure 5:**
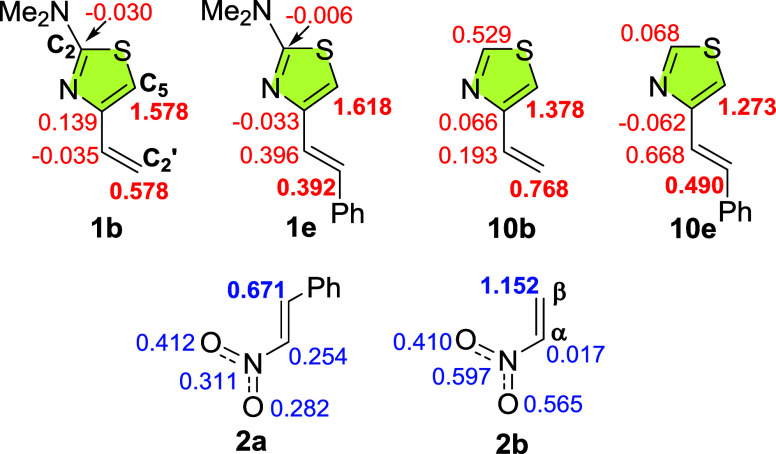
Local nucleophilicity indexes, *N*_*k*_ (in red), computed for thiazoles **1b**,**e** and **10b**,**e** and local electrophilicity indexex, ω_*k*_ (in blue), computed for nitroalkenes **2a**,**b**.

To further investigate the regioselectivity of these cycloadditions, we have also computed the nucleophilic (*P*_k_^–^) and electrophilic (*P*_k_^+^) Parr functions and calculated the local nucleophilicity and electrophilicity indexes, *N*_*k*_ and ω_*k*,_ respectively (Figure 5). These parameters characterize the most nucleophilic and electrophilic centers of the reagents and thus the nature of the best two-center interaction. Thus, the preferred reaction path will be that which involves the bond between the most electrophilic atom of the dienophile and the most nucleophilic atom of the diene.

Analysis of *N*_*k*_ values of the 2-dimethylamino-4-alkenylthiazoles **1b**,**e** indicates that the C5 atom of the 1,3-thiazole nucleus is by far the most nucleophilic center, much more than the C2′ atom, thus guiding the regioselectivity of the Diels–Alder cycloaddition. The attachment of a phenyl ring at the C2′ atom slightly increases the *N*_*k*_ value at C5 (1.578 for **1b** vs 1.618 for **1e**). This pattern is not observed in thiazoles **10b**,**e** (1.378 for **10b** vs 1.273 for **10e**). The dimethylamino group at C2 clearly amplifies the local nucleophilicity index at C5, as shown by comparing the *N*_*k*_ (C5) values of **1b**,**e** with those of **10b**,**e**. Furthermore, the difference between the *N*_*k*_ values at both ends of the diene, C5 and C2′, is greater in thiazoles **1b**,**e** than in **10b**,**e**, suggesting that the former will produce a greater degree of regioselectivity.

With respect to nitroalkenes **2a**,**b**, the largest local electrophilic value ω_*k*_ is located at the Cβ carbon atom of the alkenyl system. It is noteworthy that the attachment of a phenyl ring at this atom diminishes its ω_*k*_ value from 1.152 (**2b**) to 0.671 (**2a**).

Therefore, with all these data in hand, it is established that in the Diels–Alder reaction of thiazoles **1b**,**e** with nitroalkenes **2a**,**b**, the best electrophilic/nucleophilic interaction will occur between C5 of the thiazole nucleus and the Cβ carbon atom of the nitroalkenes, thus accounting for the regioselectivity observed in the experimental study.

#### Analysis of the Global Electron Density Transfer

Finally, our study on the electronic nature of these cycloadditions was extended to the analysis of Global Electron Density Transfer (GEDT) within molecular electron density theory (MEDT) as an additional research tool.

The GEDT from the nucleophile to electrophile frameworks could be one of the key factors controlling the activation energies of these Diels–Alder cycloadditions. For several reactions, it has been recognized that the higher the GEDT at the TS, the faster the reaction.^31^

The values of GEDT at transition structures **TS1xy** have been calculated as the sum of the natural atomic charges, obtained through NPA, of all atoms initially belonging to the diene and those belonging to the dienophile. The results are compiled in Table 6, where we have also collected the dipole moment values, which show the high polar character of transition structures corresponding to paths *A*.

**Table 6:** GEDT Values (e) at Transition States **TS1xy** Optimized at the ωB97X-D/6-31+G** Theoretical Level in Toluene Calculated as the Sum of Natural Atomic Charges of All Atoms Belonging to the Diene Interacting Fragment and Dipole Moments (m, in Debyes) Computed at the Same Level

	GEDT/m			
**TS1xy**	***endoA***	***exoA***	***endoB***	***exoB***
**1b** + **2a**	0.38/10.7	0.43/13.7	0.30/4.4	0.29/7.6
**1b** + **2b**	0.33/10.6	0.34/12.6	0.28/4.4	0.28/7.5
**1e** + **2a**	0.36/8.9	0.41/11.8	0.21/3.6	0.19/6.2
**1e** + **2b**	0.31/9.1	0.32/10.8	0.26/4.3	0.26/7.1

The TSs corresponding to channels *A* exhibit much larger GEDT values (0.31–0.43e) than those of the *B* channels (0.19–0.30e), in agreement with the lower energy barriers found for the former ones. The fact that the *A* routes have the maximum GEDT values is consistent with the result of the analysis of the local nucleophilicity and electrophilicity indexes, which predicts C5–Cβ as the best-matching nucleophile–electrophile centers, precisely the one that occurs through the *A* paths, another piece of information that supports the regioselectivity of these processes.

Furthermore, within *A* paths, the GEDT values for *exo* approaches are larger than those for *endo* orientations, particularly at **TS1ba-*exoA*** and **TS1ea-*****exoA***, which is also consistent with the *exoA* path being the most favorable in all the cases considered and accounting for the observed diastereoselectivity.

The TS that shows the highest value of GEDT is **TS1ba-*****exoA*** (0.43e), although **TS1ea-*****exoA*** is that corresponding to the fastest cycloaddition despite having a slightly lower GEDT (0.41e). These small discrepancies between this result with the above computations must have their origin in the existence of different attractive and repulsive noncovalent interactions in the transition states caused by the distinct pattern of substituents in the six-membered ring that is being formed.

**Figure 6:**
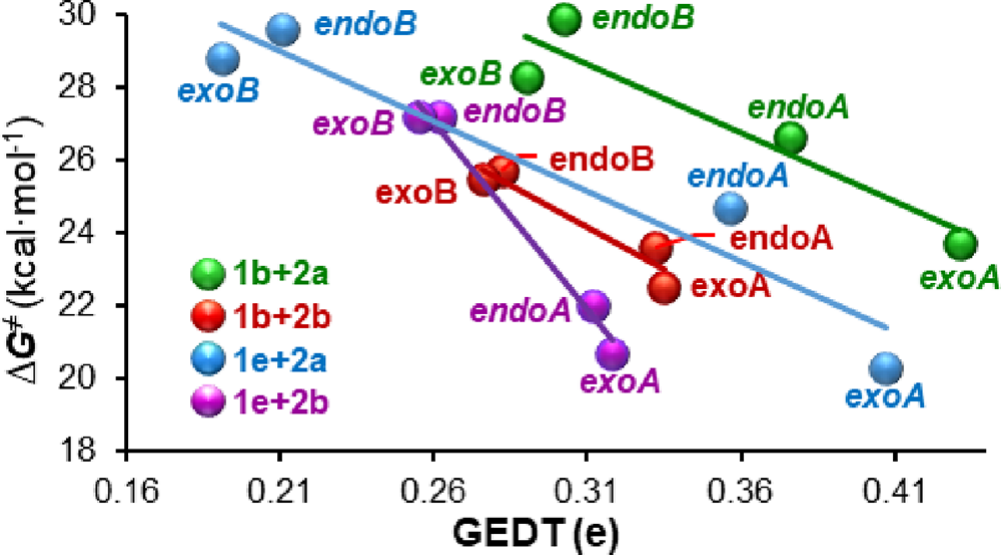
Plot of the GEDT values vs the energy barriers associated with **TS1xy** for each combination diene-dienophile.

The plot of the GEDT values vs the energy barriers for each combination diene–dienophile shows a good correlation between both parameters (Figure 6).^32^

## Conclusions

The [4 + 2] cycloaddition reactions between 4-alkenyl-2-dialkylaminothiazoles and nitroalkenes lead to 2-dialkylamino-6-nitro-4,5,6,7-tetrahydrobenzothiazoles after 1,3-*H* migration in moderate to good yields. Under certain reaction conditions and depending on the substituents at the side double bond of the thiazole ring, products resulting from either a Michael addition between the starting thiazole and nitroalkene or a Michael addition coming from the Diels–Alder-adduct intermediate and a further ene reaction are additionally obtained in minor amounts.

The position of the nitro group as well as the relative configuration of the substituents within the 2-dialkylamino-6-nitro-4,5,6,7-tetrahydrobenzothiazoles indicates, first, that the cycloaddition takes place regioselectively, and second, that the arrangement of dienophile with respect to the diene is *exo*.

This approach allows the formal synthesis of pramipexole and access to other derivatives by appropriate substitution of the 4-alkenyl-2-dimethylamino-1,3-thiazole and the nitroalkene, which could allow modulation of its biological activity.

We have scrutinized the PES associated with the four possible reaction paths for the D–A cycloaddition of 4-alkenyl-2-dimethylamino-1,3-thiazoles with nitroalkenes, depending on the relative approach of diene–dienophile and on the *exo* or *endo* orientation of the nitro group. This study predicts that in all cases, the most favorable is the path *exo*-*A*, in agreement with the experimental results. For all combinations of diene–dienophile via paths *B*, the cycloaddition takes place in a single kinetic step; however, for some cases of paths *A*, it occurs by a two-step mechanism, involving an interesting tandem of chemical processes. The degree of asynchronicity of the TSs, its dipolar moment value, and other parameters demonstrate the marked polar character of these Diels–Alder cycloadditions. We have used conceptual DFT and MEDT calculations to understand the favored mechanism by which these transformations occur. The analysis of noncovalent interactions, electronic chemical potential, chemical hardness, global and local electrophilicity and nucleophilicity, and GEDT values allows to rationalize the regio- and diastereoselectivity of our experimental results. The electronic features of our dienes, with one of its double bonds properly contained in the 4-alkenylthiazole, makes them excellent dienes as well as allow the high degrees of diastereo- and regioselectivity obtained.

## Data Availability

The data underlying this study are available in the published article and its Supporting Information.

## Supporting Information Available

Synthetic procedures; experimental data; configurational assignment of **3aa**, **3ba**, and **7bb**; crystal data and structure refinement for **3ba** (CCDC2339709); ^1^H NMR and ^13^C NMR spectra for all new compounds; bidimensional spectra for selected derivatives; computational methods; reaction profiles; electronic and Gibbs free energies; first frequency; Cartesian coordinates; ball-and-stick figures for all stationary points; and IRC plots (PDF)
(Movie S1) irc forward from TS1ba_endoA (MP4)
(Movie S2) irc forward from TS1ba_endoB (MP4)
(Movie S3) irc forward fromTS1ba_exoA (MP4)
(Movie S4) irc forward from TS1ba_exoB (MP4)
(Movie S5) irc forward from TS1bb_exoA (MP4)
(Movie S6) irc forward from TS1bb_exoB (MP4)
(Movie S7) irc forward fromTS1ea_endoA (MP4)
(Movie S8) irc forward from TS1ea_exoA (MP4)
(Movie S9) forward from TS1eb_endoA (MP4)
(Movie S10) irc forward from TS1eb_exoA (MP4)
(Movie S11) irc forward from TS2bb_endoA (MP4)
(Movie S12) forward from TS1bb_endoA (MP4)
