# Supplementary material for: Cycloadditions of 4-Alkenyl-2-aminothiazoles with Nitroalkenes in the Formal Synthesis of Pramipexole: An Experimental and Computational Study
Source: J Org Chem. 2024 Aug 19;89(17):12049–61. doi: 10.1021/acs.joc.4c00843 (PMC11382162; doi:10.1021/acs.joc.4c00843)

The Cycloadditions of 4-Alkenyl-2-aminothiazoles with Nitroalkenes in the Formal Synthesis of Pramipexole: An Experimental and Computational Study

Mateo Alajarin,[a] Jose Cabrera,[a] Delia Bautista,[b] Pilar Sanchez-Andrada*[a] and Aurelia Pastor*[a]

[a] *Department of Organic Chemistry, Faculty of Chemistry. University of Murcia. Regional Campus of International Excellence "Campus Mare Nostrum", 30100 Murcia, Spain.*
[b] *ACTI, University of Murcia, 30100 Murcia, Spain.*
E-mail: andrada@um.es, aureliap@um.es

SUPPORTING INFORMATION

Table of Contents

1. EXPERIMENTAL PART S2
  1.1 General S2
  1.2. Synthetic procedures and experimental data S3
  1.3. Configurational assignment of **3aa**, **3ba** and **7bb** S13
  1.4. Crystal data and structure refinement for **3ba** S17
  1.5. Copies of the ^{1}H NMR and $^{13}C\{^{1}H\}$ NMR spectra of all new compounds S19
  1.6. References S119
2. Computational Part S120
  2.1. Computational Methods S120
  2.2. Table S1. ..... S122
  2.3. Figure S1. ..... S124
  2.4. Figure S2. ..... S125
  2.5. Table S2. ..... S126
  2.6. Table S3. ..... S128
    Series **1b** + **2b** S128
    Series **1b** + **2a** S134
    Series **1e** + **2b** S143
    Series **1e** + **2a** S149
    Reactants S157
  2.7. IRC Energy Plots Corresponding to the Movies S159

1. EXPERIMENTAL PART

1.1 General

All melting points were determined on a Reichert hot-plate melting point apparatus and are uncorrected. IR spectra were recorded as neat or nujol emulsions on a Nicolet Impact 400 apparatus. NMR spectra were recorded on Bruker Avance 300 MHz, 400 MHz and 600 MHz instruments. ^{1}H-NMR chemical shifts are reported relative to Me_4Si and were referenced via residual proton resonances of the corresponding deuterated solvent, whereas ^{13}C-NMR spectra are reported relative to Me_4Si using the carbon signals of the deuterated solvent. Signals in the ^{1}H- and ^{13}C-NMR spectra were assigned with the aid of DEPT-135 or two-dimensional NMR experiments (COSY, NOESY and HSQC). Abbreviations of coupling patterns are as follows: br, broad; s, singlet; d, doublet; t, triplet; q, quadruplet; m, multiplet. Mass spectra were recorded on the EI (70 eV) mode and high-resolution mass spectra (HRMS) were recorded on an Agilent HPLC 1200/MS TOF 6220 instrument. Single crystal X-ray diffraction data were obtained at 100 K using a Bruker D8 Quest diffractometer equipped with Mo Kα radiation and a Photon 100 detector.

1.2. Synthetic procedures and experimental data

Synthesis of the 1-chlorobut-3-en-2-one, 1,4-dichlorobutan-2-one, *N*,*N*'-dimethylthiourea, (*E*)-*N*,*N*-dimethyl-4-[2-(*p*-tolyl)ethenyl]thiazol-2-amine 1a and nitroethylene. The synthesis of 1-chlorobut-3-en-2-one has been previously described in the literature through a two-step synthesis, being the first step the preparation of 1,4-dichlorobutan-2-one.[1] The synthesis of *N*,*N*'-dimethylthiourea and **1a** have been previously reported by the authors.[2] Nitroethylene was prepared following the experimental procedure described by Ranganathan and coworkers.[3]

Synthesis of *N*,*N*'-dibenzylthiourea: *N*,*N*'-dibenzylthiourea was prepared from ethoxycarbonyl isothiocyanate following a slightly modified method from that previously described:[4] To a solution of ethoxycarbonyl isothiocyanate (0.50 g, 3.81 mmol) in acetone (50 mL), a solution of *N*,*N*'-dibenzylamine (0.94 g, 4.77 mmol) in acetone (5 mL) was slowly added at 25 ºC. The reaction mixture was stirred at this temperature for 45 min and then, the solvent was evaporated to dryness. After addition of concentrated HCl (50 mL) the reaction mixture was heated until gas evolution was observed. After that, the reaction mixture was neutralized with 30% aqueous NH_3, the aqueous phase was saturated with NaCl and extracted with CH_2Cl_2 (3 × 50 mL). The organic extracts were dried over anhydrous $MgSO_4$, and the solvent evaporated to dryness. *N*,*N*-Dibenzylthiourea was purified by silica-gel chromatography eluting with 1:5→1:2 AcOEt/*n*-hexane (R_f = 0.20 in 1:2 AcOEt/*n*-hexane) (0.52 g, 53%). The spectroscopical data agree well with those described previously in the literature.[5] ^{1}H NMR ($CDCl_3$, 400 MHz, 298 K) δ 7.38-7.27 (m, 10H), 5.71 (br s, 2H), 4.93 (s, 4H); ^{13}C{^{1}H} NMR ($CDCl_3$, 100 MHz, 298 K) δ 184.6 (s), 135.6 (s), 129.2 (d), 128.2 (d), 127.3 (d), 54.8 (t).

General procedure for the synthesis of the 4-(2-chloroethyl)thiazol-2-amines 5a-c: To a solution of 1,4-dichlorobutan-2-one (2.0 g, 14.18 mmol, 1 equiv) in methanol (50 ml, 0.1 *M*) the corresponding thiourea (5.45 g, 21.27 mmol, 1.5 equiv) was added. The reaction mixture was stirred at 25 ºC for 3 h. After removal of the solvent, an aqueous solution of 5% $NaHCO_3$ (50 ml) was added, and the resulting mixture was extracted with CH_2Cl_2 (3×50 ml). The organic extracts were dried under anhydrous $MgSO_4$. After removal of the solvent the residue was purified by silica-gel chromatography.

4-(2-Chloroethyl)-*N*,*N*-dimethylthiazol-2-amine (5a). Compound **5a** was obtained as a colorless oil in 92% yield (0.21 g) from 1,4-dichlorobutan-2-one (0.2 g, 1.17 mmol), after purification by silica-gel chromatography using 1:5 AcOEt/*n*-hexane as eluent (R_f = 0.30); IR (neat) 1560, 1532, 1423, 1408, 1344, 1288, 1120, 1064, 914, 710 cm^{-1}; ^{1}H NMR ($CDCl_3$, 400 MHz, 298 K) δ 6.20 (t, ^4J = 0.8 Hz, 1H, H_5), 3.80 (t, ^3J = 7.2 Hz, 2H, Cl<u>CH_2</u>CH_2), 3.07 (s, 6H, NMe_2), 3.00 (td, ^3J = 7.2 Hz, ^4J = 0.8 Hz, 2H, ClCH_2<u>CH_2</u>); $^{13}C\{^1H\}$ NMR ($CDCl_3$, 100 MHz, 298 K) δ 171.2 (s, C_2), 149.9 (s, C_4), 102.3 (d, C_5), 43.3 (t), 40.2 (2×q, NMe_2), 35.4 (t); MS (EI, 70 eV) *m/z* (rel int) 192 (M^+ + 2, 45), 190 (M^+, 100), 175 (49), 163 (37), 161 (88), 155 (66), 149 (67), 141 (38), 71 (73), 57 (60). HRMS (ESI) m/z: $[M + H]^+$ calcd. for $C_7H_{12}ClN_2S$, 191.0404; Found, 191.0408.

4-(2-Chloroethyl)-*N*,*N*-dibenzylthiazol-2-amine (5b). Compound **5b** was obtained as a colorless oil in 95% yield (4.62 g) from 1,4-dichlorobutan-2-one (2.0 g, 14.18 mmol), after purification by silica-gel chromatography using 1:10 AcOEt/*n*-hexane as eluent (R_f = 0.36); IR (neat) 1527, 1494, 1453, 1439, 1331, 1289, 1214, 754, 697 cm^{-1}; ^{1}H NMR ($CDCl_3$, 400 MHz, 298 K) δ 7.35-7.23 (m, 10H), 6.21 (t, ^4J = 0.7 Hz, 1H, H_5), 4.62 (s, 4H, 2×<u>CH_2</u>Ph), 3.84 (t, ^3J = 7.2 Hz, 2H, Cl<u>CH_2</u>CH_2), 3.02 (td, ^3J = 7.2 Hz, ^4J = 0.6 Hz, 2H, ClCH_2<u>CH_2</u>); $^{13}C\{^1H\}$ NMR ($CDCl_3$, 100 MHz, 298 K) δ 171.3 (s, C_2), 149.8 (s, C_4), 136.9 (2×s), 128.8 (4×d), 128.0 (4×d), 127.7 (2×d), 102.3 (d, C_5), 53.7 (2×t, 2×<u>CH_2</u>Ph), 43.5 (t), 35.6 (t); MS (EI, 70 eV) *m/z* (rel int) 344 (M^+ + 2, 5), 342 (M^+, 13), 307 (26), 306 (52), 251 (33), 216 (31), 215 (91), 92 (27), 91 (100), 65 (33). HRMS (ESI) m/z: $[M + H]^+$ calcd. for $C_{19}H_{20}ClN_2S$, 343.1030; found, 343.1025.

4-(2-Chloroethyl)thiazol-2-amine (5c).[6] Compound **5c** was obtained in 85% yield (0.49 g) from 1,4-dichlorobutan-2-one (0.5 g, 3.55 mmol), after purification by silica-gel chromatography using 1:1 AcOEt/*n*-hexane as eluent (R_f = 0.23); mp 68-69 ºC (colorless prims from $CHCl_3/Et_2O$); IR (nujol) 3446, 3257, 1618, 1515, 1319, 1224, 1101, 976, 756, 710, 638 cm^{-1}; ^{1}H NMR ($CDCl_3$, 400 MHz, 298 K) δ, 6.22 (br s, 1H, H_5), 5.28 (br s, 2H, NH_2), 3.75 (t, ^3J = 7.1 Hz, 2H, Cl<u>CH_2</u>CH_2), 2.96 (t, ^3J = 7.1 Hz, 2H, ClCH_2<u>CH_2</u>); $^{13}C\{^1H\}$ NMR ($CDCl_3$, 100 MHz, 298 K) δ 167.9 (s, C_2), 148.8 (s, C_4), 104.5 (d, C_5), 43.2 (t), 35.1 (t); MS (EI, 70 eV) *m/z* (rel int) 164 (M^+ + 2, 37), 162 (M^+, 100), 126 (94), 113 (59), 86 (34), 85 (36), 71 (61), 69 (43).

Synthesis of the 4-vinylthiazol-2-amines 1b,c: Potassium *terc*-butoxide (0.18 g, 1.58 mmol, 1.5 equiv) was added to a solution of the corresponding 4-(2-chloroethyl)thiazol-2-amine **5a** or **5b** (1.05 mmol, 1 equiv) in EtOH (20 mL, 0.05 *M*). The reaction mixture was stirred under reflux in an oil bath for 12 h. The solvent was removed under reduced pressure and H_2O (15 ml) was added. The aqueous solution was extracted with CH_2Cl_2 (3 × 15 ml) and the organic extracts were dried over anhydrous $MgSO_4$. After filtration of the inorganic salts, the solvent was removed under reduced pressure and the residue purified by silica-gel chromatography.

***N,N*-Dimethyl-4-vinylthiazol-2-amine (1b).** Compound **1b** was obtained as a colorless oil in 65% yield (0.105 g) after purification by silica-gel chromatography using 1:5 AcOEt/*n*-hexane as eluent (R_f = 0.39); IR (neat) 1625, 1561, 1494, 1425, 1344, 1117, 1040, 985, 966, 909, 751, 727 cm^{-1}; ^1H NMR ($CDCl_3$, 400 MHz, 298 K) δ 6.50 (dd, ^3J = 17.1 Hz, ^3J = 10.7 Hz, 1H, CH_2=<u>CH</u>), 6.33 (s, 1H, H_5), 5.93 (dd, ^3J = 17.1 Hz, ^2J = 1.9 Hz, 1H, (*E*)-<u>CH_2</u>=CH), 5.25 (dd, ^3J = 10.7 Hz, ^2J = 2.0 Hz, 1H, (*Z*)-<u>CH_2</u>=CH), 3.09 (s, 6H, NMe_2); $^{13}C\{^1H\}$ NMR ($CDCl_3$, 100 MHz, 298 K) δ 170.8 (s, C_2), 151.4 (s, C_4), 130.6 (d, CH_2=<u>CH</u>), 115.3 (t, <u>CH_2</u>=CH), 104.8 (d, C_5), 40.2 (2×q, NMe_2); MS (EI, 70 eV) *m/z* (rel int) 154 (M^+, 100), 139 (53), 125 (73), 112 (15). HRMS (ESI) m/z: $[M + H]^+$ calcd. for $C_7H_{11}N_2S$, 155.0638; found, 155.0645.

***N,N*-Dibenzyl-4-vinylthiazol-2-amine (1c).** Compound **1c** was obtained in 60% yield (0.79 g) starting from **5b** (1.5 g, 4.37 mmol), after purification by silica-gel chromatography using 1:10 AcOEt/*n*-hexane as eluent (R_f = 0.42); mp 51-52 ºC (colorless prims, $CHCl_3/Et_2O$); IR (nujol) 1548, 1493, 1334, 1290, 1217, 1204, 1079, 944, 924, 765, 719, 700, 622 cm^{-1}; ^1H NMR ($CDCl_3$, 400 MHz, 298 K) δ 7.34-7.24 (m, 10H), 6.52 (dd, ^3J = 17.1 Hz, ^3J = 10.6 Hz, 1H, CH_2=<u>CH</u>), 6.33 (s, 1H, H_5), 5.97 (dd, ^3J = 17.1 Hz, ^2J = 2.0 Hz, 1H, (*E*)-<u>CH_2</u>=CH), 5.27 (dd, ^3J = 10.6 Hz, ^2J = 2.2 Hz, 1H, (*Z*)-<u>CH_2</u>=CH), 4.66 (s, 4H, 2×<u>CH_2</u>Ph); $^{13}C\{^1H\}$ NMR ($CDCl_3$, 100 MHz, 298 K) δ 170.8 (s, C_2), 151.2 (s, C_4), 136.9 (2×s), 130.5 (d, CH_2=<u>CH</u>), 128.7 (4×d), 128.1 (4×d), 127.7 (2×d), 115.6 (t, <u>CH_2</u>=CH), 104.7 (d, C_5), 53.5 (2×t, 2×<u>CH_2</u>Ph); MS (EI, 70 eV) *m/z* (rel int) 306 (M^+, 28), 215 (100), 91 (99), 65 (33). HRMS (ESI) m/z: $[M + H]^+$ calcd. for $C_{19}H_{19}N_2S$, 307.1264; found, 307.1263.

Synthesis of the *N,N*-bis(4-methoxybenzyl)-4-vinylthiazol-2-amine 1d: A suspension of sodium hydride 60% in mineral oil (0.98 g, 24.59 mmol) was added to a solution of 4-(2-chloroethyl)thiazol-2-amine (**5c**) (1.00 g, 6.15 mmol) and methoxybenzyl chloride (1.93 g, 12.30 mmol) in anhydrous DMF (100 mL) cooled to 0 °C. The reaction mixture was stirred under 25 ºC for 24 h. After addition of MeOH (20 mL), the solvent was removed under reduced pressure and H_2O (100 mL) was added. The aqueous solution was extracted with CH_2Cl_2 (3 × 75 ml) and the organic extracts were dried over anhydrous $MgSO_4$. After filtration of the inorganic salts, the solvent was removed under reduced pressure and the residue purified by silica-gel chromatography eluting with 1:5 AcOEt/*n*-hexane (R_f = 0.28) to give **1d** as a colorless oil in 72% yield (1.63 g); IR (neat) 1611, 1544, 1511, 1441, 1338, 1303, 1248, 1175, 1036, 820, 752 cm^{-1}; ^1H NMR ($CDCl_3$, 400 MHz, 298 K) δ 7.22-7.19 (m, 4H), 6.88-6.84 (m, 4H), 6.53 (dd, ^3J = 17.1 Hz, ^3J = 10.7 Hz, 1H, CH_2=<u>CH</u>), 6.33 (s, 1H, H_5), 5.99 (dd, ^3J = 17.1 Hz, ^2J = 2.0 Hz, 1H, (*E*)-<u>CH_2</u>=CH), 5.28 (dd, ^3J = 10.6 Hz, ^2J = 2.0 Hz, 1H, (*Z*)-<u>CH_2</u>=CH), 4.58 (s, 4H, 2×<u>CH_2</u>Ar), 3.81 (s, 6H, 2×OMe); $^{13}C\{^1H\}$ NMR ($CDCl_3$, 100 MHz, 298 K) δ 170.8 (s, C_2), 159.2 (2×s), 151.1 (s, C_4), 130.6 (d, CH_2=<u>CH</u>), 129.5 (4×d), 128.9 (2×s), 115.5 (t, <u>CH_2</u>=CH), 114.1 (4×d), 104.6 (d, C_5), 55.4 (2×q, 2×OMe), 52.8 (2×t, 2×<u>CH_2</u>Ar); MS (EI, 70 eV) *m/z* (rel int) 366 (M^+, 24), 245 (45), 135 (29), 121 (100), 91 (42), 78 (38), 77 (46). HRMS (ESI) m/z: $[M + H]^+$ calcd. for $C_{21}H_{23}N_2O_2S$, 367.1475; found, 367.1459.

General procedure for the reactions of 4-alkenylthiazol-2-amines 1a,b with *trans*-β-nitrostyrene: A solution of the corresponding 4-alkenylthiazol-2-amines **1a** or **1b** (1 equiv) and *trans*-β-nitrostyrene (3 equiv) in acetonitrile (0.05 *M*) was stirred at 25 ºC or under reflux in an oil bath. Temperature, reaction time, isolation and purification methodologies are described for every example.

(5*R,6*R**,7*S**)-*N,N*-Dimethyl-6-nitro-7-phenyl-5-(*p*-tolyl)-4,5,6,7-tetrahydrobenzo[*d*]thiazol-2-amine (3aa).** The reaction mixture was stirred at 25 ºC for 72 h. The product precipitated in the reaction mixture, it was filtered and dried under vacuum. Compound **3aa** was obtained in 82% yield (0.132 g) starting from **1a** (0.1 g, 0.41 mmol); mp 248-250 ºC (colorless needles, $CHCl_3/Et_2O$); IR (nujol) 1576, 1556, 1514, 1422, 1339, 1261, 820, 702 cm^{-1}; ^1H NMR ($CDCl_3$, 400 MHz, 298 K) δ 7.35-7.29 (m, 3H, H_m-Ph + H_p-Ph), 7.25-7.23 (m, 2H, H_o-Ph), 7.18-7.16 (m,

2H, H_o-*p*Tol), 7.14-7.12 (m, 2H, H_m-*p*Tol), 5.03 (dd, ^3J = 11.6 Hz, ^3J = 9.8 Hz, 1H, H_6), 4.79 (ddd, ^3J = 9.8 Hz, ^5J = 3.1 Hz, ^5J = 1.3 Hz, 1H, H_7), 3.73 (td, ^3J = 11.6 Hz, ^3J = 5.8 Hz, 1H, H_5), 3.15 (ddd, ^2J = 17.2 Hz, ^3J = 5.8 Hz, ^5J = 1.5 Hz, 1H, H_{4b}), 3.08 (ddd, ^2J = 17.2 Hz, ^3J = 11.6 Hz, ^5J = 3.2 Hz, 1H, H_{4a}), 3.04 (s, 6H, NMe_2), 2.32 (s, 3H, $C_6H_4\underline{Me}$); $^{13}C\{^1H\}$ NMR ($CDCl_3$, 100 MHz, 298 K) δ 171.0 (s, C_2), 146.3 (s, C_{3a}), 139.8 (s), 137.8 (s), 135.7 (s), 129.8 (2×d), 129.1 (2×d), 128.6 (d), 128.1 (2×d), 127.5 (2×d), 117.0 (s, C_{7a}), 96.4 (d, C_6), 48.0 (d, C_7), 45.9 (d, C_5), 40.3 (2×q, NMe_2), 34.5 (t, C_4), 21.2 (q, $C_6H_4\underline{Me}$); MS (EI, 70 eV) *m/z* (rel int) 393 (M^+, 11), 347 (25), 346 (100), 345 (29), 255 (33), 241 (56), 115 (21), 88 (24). HRMS (ESI) m/z: $[M + H]^+$ calcd. for $C_{22}H_{24}N_3O_2S$, 394.1584; found, 394.1586.

(6*R,7*S**)-*N*,*N*-Dimethyl-6-nitro-7-phenyl-4,5,6,7-tetrahydrobenzo[*d*]thiazol-2-amine (3ba).** The reaction mixture was stirred under reflux for 24 h in an oil bath. The solvent was evaporated to dryness and the residue purified by silica-gel chromatography eluting with 1:3→1:1 AcOEt/*n*-hexane (R_f = 0.23 in 1:1 AcOEt/*n*-hexane) to give **3ba** in 60% yield (0.173 g) starting from **1b** (0.15 g, 0.97 mmol); mp 178-179 ºC (colorless needles, $CHCl_3/Et_2O$); IR (nujol) 1549, 1401, 1339, 1289, 1257, 1126, 810, 700 cm^{-1}; ^1H NMR ($CDCl_3$, 600 MHz, 298 K) δ 7.35-7.28 (m, 3H, H_m-Ph + H_p-Ph), 7.22-7.21 (m, 2H, H_o-Ph), 4.80 (ddd, ^3J = 10.4 Hz, ^3J = 7.7 Hz, ^3J = 3.0 Hz, 1H, H_6), 4.71 (dt, ^3J = 8.0 Hz, ^5J = 1.8 Hz, 1H, H_7), 3.03 (s, 6H, NMe_2), 2.90-2.87 (m, 2H, H_4), 2.55-2.48 (m, 1H, H_{5b}), 2.44 (dtd, ^2J = 13.2 Hz, ^3J = 5.1 Hz, ^3J = 3.2 Hz, 1H, H_{5a}); $^{13}C\{^1H\}$ NMR ($CDCl_3$, 150 MHz, 298 K) δ 170.8 (s, C_2), 146.4 (s, C_{3a}), 140.3 (s), 129.0 (2×d), 128.3 (d), 128.2 (2×d), 116.5 (s, C_{7a}), 89.8 (d, C_6), 45.6 (d, C_7), 40.3 (2×q, NMe_2), 27.4 (t, C_5), 25.0 (t, C_4); MS (EI, 70 eV) *m/z* (rel int) 303 (M^+, 41), 256 (100), 185 (30), 179 (37), 152 (33), 128 (36), 115 (34), 88 (34). HRMS (ESI) m/z: $[M + H]^+$ calcd. for $C_{15}H_{18}N_3O_2S$, 304.1114; found, 304.1108.

***N*,*N*-Dimethyl-5-(2-nitro-1-phenylethyl)-4-vinythiazol-2-amine (6ba).** The reaction mixture was stirred under reflux for 24 h in an oil bath. The solvent was evaporated to dryness and the residue purified by silica-gel chromatography eluting with 1:3→1:1 AcOEt/*n*-hexane (R_f = 0.31 in 1:3 AcOEt/*n*-hexane) to give **6ba** in 14% yield (40 mg) as a yellow oil starting from **1b** (0.15 g, 0.97 mmol); IR (neat) 1556, 1496, 1454, 1424, 1376, 1328, 1265, 1137, 1043, 980, 918, 736, 700 cm^{-1}; ^1H NMR ($CDCl_3$, 600 MHz, 298 K) δ 7.36-7.33 (m, 2H), 7.29-7.26 (m, 3H), 6.72 (dd, ^3J = 16.8 Hz, ^3J = 10.7 Hz, 1H, CH_2=$\underline{CH}$), 6.06 (dd, ^3J = 16.8 Hz, ^2J = 2.2 Hz, 1H, (*E*)-$\underline{CH_2}$=CH), 5.40

(dd, ^3J = 10.7 Hz, ^2J = 2.2 Hz, 1H, (*Z*)-$\underline{CH_2}$=CH), 5.21 (t, ^3J = 8.0 Hz, 1H, $\underline{CH}PhCH_2NO_2$), 4.96 (dd, ^2J = 12.8 Hz, ^3J = 8.2 Hz, 1H, $\underline{CH_2}NO_2$), 4.81 (dd, ^2J = 12.8 Hz, ^3J = 7.9 Hz, 1H, $\underline{CH_2}NO_2$), 3.04 (s, 6H, NMe_2); $^{13}C\{^1H\}$ NMR ($CDCl_3$, 150 MHz, 298 K) δ 168.5 (s, C_2), 147.2 (s, C_4), 138.8 (s), 129.2 (2×d), 128.0 (d), 127.2 (2×d), 127.0 (d, CH_2=$\underline{CH}$), 120.8 (s, C_5), 117.8 (t, $\underline{CH_2}$=CH), 79.4 (t, $CHPh\underline{CH_2}NO_2$), 41.4 (d, $\underline{CH}PhCH_2NO_2$), 40.1 (2×q, NMe_2); MS (EI, 70 eV) *m/z* (rel int) 303 (M^+, 71), 255 (21), 243 (100), 169 (52), 88 (21). HRMS (ESI) m/z: $[M + H]^+$ calcd. for $C_{15}H_{18}N_3O_2S$, 304.1114; found, 304.1105.

General procedure for the reaction of 4-alkenylthiazol-2-amines 1b-d with nitroethylene: Nitroethylene[3] (2 equiv) was added to a solution of the corresponding 4-alkenylthiazol-2-amine **1b-d** (1 equiv) in anhydrous toluene (0.02 *M*). The reaction mixture was stirred at 60 °C in an oil bath for 36 h. The residue was purified by silica-gel chromatography.

***N*,*N*-Dimethyl-6-nitro-4,5,6,7-tetrahydrobenzo[*d*]thiazol-2-amine (3bb).** The residue was purified by silica-gel chromatography eluting with 1:2→1:1 AcOEt/*n*-hexane (R_f = 0.21 in 1:1 AcOEt/*n*-hexane) to give **3bb** in 65% yield (48 mg) starting from **1b** (0.05 g, 0.32 mmol); mp 133-134 ºC (colorless needles, $CHCl_3/Et_2O$); IR (nujol) 1539, 1414, 1403, 1341, 1289, 1255, 1125, 706 cm^{-1}; ^1H NMR ($CDCl_3$, 400 MHz, 298 K) δ 4.80-4.73 (m, 1H, H_6), 3.29 (ddt, ^2J = 15.7 Hz, ^3J = 8.1 Hz, ^5J = 2.1 Hz, 1H, H_{7b}), 3.20-3.15 (m, 1H, H_{7a}), 3.03 (s, 6H, NMe_2), 2.82-2.66 (m, 2H, H_4), 2.46-2.34 (m, 2H, H_5); $^{13}C\{^1H\}$ NMR ($CDCl_3$, 100 MHz, 298 K) δ 169.9 (s, C_2), 145.4 (s, C_{3a}), 110.9 (s, C_{7a}), 81.3 (d, C_6), 40.3 (2×q, NMe_2), 27.64 (t), 27.62 (t), 24.7 (t, C_4); MS (EI, 70 eV) *m/z* (rel int) 227 (M^+, 21), 181 (27), 180 (100), 88 (25), 71 (22). HRMS (ESI) m/z: $[M + H]^+$ calcd. for $C_9H_{14}N_3O_2S$, 228.0801; found, 228.0796.

***N*,*N*-Dimethyl-5-(2-nitroethyl)-4-vinythiazol-2-amine (6bb).** The residue purified by silica-gel chromatography eluting with 1:2→1:1 AcOEt/*n*-hexane (R_f = 0.45 in 1:1 AcOEt/*n*-hexane) to give **6bb** in 16% yield (12 mg) as a yellow oil starting from **1b** (0.05 g, 0.32 mmol); IR (neat) 1554, 1426, 1408, 1377, 1329, 1259, 1136, 1066, 1038, 982, 916, 747, 734 cm^{-1}; ^1H NMR ($CDCl_3$, 400 MHz, 298 K) δ 6.54 (dd, ^3J = 16.8 Hz, ^3J = 10.7 Hz, 1H, CH_2=$\underline{CH}$), 6.00 (dd, ^3J = 16.8 Hz, ^2J = 2.2 Hz, 1H, (*E*)-$\underline{CH_2}$=CH), 5.36 (dd, ^3J = 10.7 Hz, ^2J = 2.2 Hz, 1H, (*Z*)-$\underline{CH_2}$=CH), 4.51 (t, ^3J = 7.2 Hz, 2H, $CH_2\underline{CH_2}NO_2$), 3.41 (t, ^3J = 7.2 Hz, 2H, $\underline{CH_2}CH_2NO_2$), 3.07 (s, 6H, NMe_2); $^{13}C\{^1H\}$ NMR

($CDCl_3$, 100 MHz, 298 K) δ 168.4 (s, C_2), 147.9 (s, C_4), 126.8 (d, $CH_2=\underline{CH}$), 117.2 (t, $\underline{CH_2}=CH$), 115.6 (s, C_5), 75.7 (t, $CH_2\underline{CH_2}NO_2$), 40.1 (2×q, NMe_2), 24.1 (t, $\underline{CH_2}CH_2NO_2$); MS (EI, 70 eV) *m/z* (rel int) 227 (M^+, 81), 180 (100), 169 (53), 167 (62), 165 (46), 151 (41), 97 (43), 88 (46), 71 (59). HRMS (ESI) m/z: $[M + H]^+$ calcd. for $C_9H_{14}N_3O_2S$, 228.0801; found, 228.0796.

(4*R, 6*R**)-*N*,*N*-Dimethyl-4-(2-nitroethyl)-6-nitro-4,5,6,7-tetrahydrobenzo[*d*]thiazol-2-amine (7bb).** Compound **7bb** was isolated when the reaction of **1b** (0.21 g, 1.37 mmol) and nitroethylene (0.10 g, 1.37 mmol) was conducted in acetonitrile (15 mL) at 25 ºC for 12 h. After removal of the solvent the residue was purified by silica-gel chromatography eluting with 1:5→1:2→1:1 AcOEt/*n*-hexane (R_f = 0.48 in 1:2 AcOEt/*n*-hexane); yield 7% (30 mg); the starting 4-vinylthiazol-2-amine **1b** and **3bb** were also isolated in 36% (76 mg) and 13% (40 mg), respectively; mp 113-114 ºC (colorless needles, $CHCl_3/Et_2O$) ; IR (nujol) 1585, 1545, 1423, 1346, 1291, 1137, 716 cm^{-1}; ^1H NMR ($CDCl_3$, 400 MHz, 298 K) δ 4.88-4.81 (m, 2H, $CH_2\underline{CH_2}NO_2$ + H_6), 4.73 (dt, ^2J = 13.9 Hz, ^3J = 7.6 Hz, 1H, $CH_2\underline{CH_2}NO_2$), 3.35 (dd, ^2J = 16.3 Hz, ^3J = 6.6 Hz, 1H, H_{7b}), 3.19 (dd, ^2J = 16.4 Hz, ^3J = 5.4 Hz, 1H, H_{7a}), 3.03 (s, 6H, NMe_2), 2.99-2.92 (m, 1H, H_4), 2.62 (ddd, ^2J = 13.9 Hz, ^3J = 8.5 Hz, ^3J = 5.7 Hz, 1H, H_{5b}), 2.34-2.28 (m, 2H, $\underline{CH_2}CH_2NO_2$), 2.13 (ddd, ^2J = 13.7 Hz, ^3J = 6.0 Hz, ^3J = 3.1 Hz, 1H, H_{5a}); $^{13}C\{^1H\}$ NMR ($CDCl_3$, 100 MHz, 298 K) δ 169.7 (s, C_2), 147.2 (s, C_{3a}), 111.8 (s, C_{7a}), 79.2 (d, C_6), 74.1 (t, $CH_2\underline{CH_2}NO_2$), 40.1 (2×q, NMe_2), 33.1 (t, C_5), 32.7 (t, $\underline{CH_2}CH_2NO_2$), 31.9 (d, C_4), 27.4 (t, C_7); MS (EI, 70 eV) *m/z* (rel int) 300 (M^+, 33), 253 (37), 205 (60), 180 (40), 179 (100), 88 (43). HRMS (ESI) m/z: $[M + H]^+$ calcd. for $C_{11}H_{17}N_4O_4S$, 301.0965; found, 301.0969.

***N*,*N*-Dibenzyl-6-nitro-4,5,6,7-tetrahydrobenzo[*d*]thiazol-2-amine (3cb).** The residue was purified by silica-gel chromatography eluting with 1:5 AcOEt/*n*-hexane (R_f = 0.20) to give **3cb** in 52% yield (0.129 g) starting from **1c** (0.20 g, 0.65 mmol); mp 90-92 ºC (orange prims, $CHCl_3/Et_2O$); IR (nujol) 1536, 1493, 1314, 1289, 1195, 763, 714, 699 cm^{-1}; ^1H NMR ($CDCl_3$, 400 MHz, 298 K) δ 7.35-7.28 (m, 6H), 7.24-7.22 (m, 4H), 4.84-4.77 (m, 1H, H_6), 4.60 (s, 4H, 2×$\underline{CH_2}Ph$), 3.29 (ddt, ^2J = 15.7 Hz, ^3J = 8.2 Hz, ^5J = 2.1 Hz, 1H, H_{7b}), 3.19 (dd, ^2J = 15.7 Hz, ^3J = 5.4 Hz, 1H, H_{7a}), 2.89-2.70 (m, 2H, H_4), 2.50-2.38 (m, 2H, H_5); $^{13}C\{^1H\}$ NMR ($CDCl_3$, 75 MHz, 298 K) δ 170.0 (s, C_2), 145.2 (s, C_{3a}), 136.6 (2×s), 128.8 (4×d), 127.9 (4×d), 127.7 (2×d), 110.9 (s, C_{7a}), 81.4 (d, C_6), 53.5 (2×t, 2×$\underline{CH_2}Ph$), 27.72 (t), 27.69 (t), 24.8 (t, C_4); MS (EI, 70 eV) *m/z*

(rel int) 379 (M^+, 42), 333 (23), 332 (66), 288 (58), 242 (40), 241 (80), 92 (24), 91 (100), 65 (45). HRMS (ESI) m/z: $[M + H]^+$ calcd. for $C_{21}H_{22}N_3O_2S$, 380.1427; found, 380.1432.

***N*,*N*-Dibenzyl-5-(2-nitroethyl)-4-vinythiazol-2-amine (6cb).** The residue was purified by silica-gel chromatography eluting with 1:5 AcOEt/*n*-hexane (R_f = 0.33) to give **6cb** in 8% yield (20 mg) as a yellow oil starting from **1c** (0.20 g, 0.65 mmol); IR (neat) 1603, 1552, 1495, 1454, 1378, 1329, 1266, 1206, 1115, 1079, 1029, 736, 700 cm^{-1}; ^{1}H NMR ($CDCl_3$, 400 MHz, 298 K) δ 7.36-7.26 (m, 10H, 2×Ph), 6.58 (dd, ^3J = 16.8 Hz, ^3J = 10.7 Hz, 1H, $CH_2=\underline{CH}$), 6.07 (dd, ^3J = 16.8 Hz, ^2J = 2.3 Hz, 1H, (*E*)-$\underline{CH_2}$=CH), 5.38 (dd, ^3J = 10.7 Hz, ^2J = 2.3 Hz, 1H, (*Z*)-$\underline{CH_2}$=CH), 4.63 (s, 4H, 2×$\underline{CH_2}$Ph), 4.51 (t, ^3J = 7.3 Hz, 2H, $CH_2\underline{CH_2}NO_2$), 3.41 (t, ^3J = 7.3 Hz, 2H, $\underline{CH_2}CH_2NO_2$); $^{13}C\{^1H\}$ NMR ($CDCl_3$, 100 MHz, 298 K) δ 168.3 (s, C_2), 147.6 (s, C_4), 136.7 (2×s), 128.8 (4×d), 128.1 (4×d), 127.8 (2×d), 126.7 (d, $CH_2=\underline{CH}$), 117.5 (t, $\underline{CH_2}$=CH), 115.4 (s, C_5), 75.6 (t, $CH_2\underline{CH_2}NO_2$), 53.5 (2×t, 2×$\underline{CH_2}$Ph), 24.0 (t, $\underline{CH_2}CH_2NO_2$); MS (EI, 70 eV) *m/z* (rel int) 379 (M^+, 96), 332 (97), 321 (61), 288 (91), 258 (62), 241 (100), 106 (59), 91 (99), 65 (63). HRMS (ESI) m/z: $[M + H]^+$ calcd. for $C_{21}H_{22}N_3O_2S$, 380.1427; found, 380.1437.

***N*,*N*-Bis(4-methoxybenzyl)-6-nitro-4,5,6,7-tetrahydrobenzo[*d*]thiazol-2-amine (3db).** The residue was purified by silica-gel chromatography eluting with 1:3 AcOEt/*n*-hexane (R_f = 0.15) to give **3db** in 83% yield (0.50 g) starting from **1d** (0.50 g, 1.36 mmol); mp 108-109 ºC (orange prims, $CHCl_3/Et_2O$); IR (nujol) 1609, 1585, 1548, 1528, 1512, 1302, 1245, 1031, 816 cm^{-1}; ^{1}H NMR ($CDCl_3$, 600 MHz, 298 K) δ 7.15 (d, ^3J = 8.7 Hz, 4H, H_o-Ar), 6.85 (d, ^3J = 8.7 Hz, 4H, H_m-Ar), 4.82-4.78 (m, 1H, H_6), 4.51 (s, 4H, 2×$\underline{CH_2}$Ar), 3.80 (s, 6H, 2×OMe), 3.28 (ddt, ^2J = 15.7 Hz, ^3J = 8.3 Hz, ^5J = 1.9 Hz, 1H, H_{7b}), 3.18 (dd, ^2J = 15.7 Hz, ^3J = 5.3 Hz, 1H, H_{7a}), 2.86-2.82 (m, 1H, H_{4b}), 2.78-2.73 (m, 1H, H_{4a}), 2.49-2.40 (m, 2H, H_5); $^{13}C\{^1H\}$ NMR ($CDCl_3$, 100 MHz, 298 K) δ 169.9 (s, C_2), 159.2 (2×s), 145.1 (s, C_{3a}), 129.3 (4×d), 128.6 (2×s), 114.1 (4×d), 110.6 (s, C_{7a}), 81.4 (d, C_6), 55.4 (2×q, 2×OMe), 52.7 (2×t, 2×$\underline{CH_2}$Ar), 27.72 (t, C_7), 27.70 (t, C_5), 24.8 (t, C_4); MS (EI, 70 eV) *m/z* (rel int) 439 (M^+, 17), 318 (43), 123 (27), 121 (100), 91 (32), 78 (36), 77 (47). HRMS (ESI) m/z: $[M + H]^+$ calcd. for $C_{23}H_{26}N_3O_4S$, 440.1639; found, 440.1656.

***N*,*N*-Bis(4-methoxybenzyl)-5-(2-nitroethyl)-4-vinythiazol-2-amine (6db).** The residue purified by silica-gel chromatography eluting with 1:3 AcOEt/*n*-hexane (R_f = 0.25) to give **6db** in 7% yield

(40 mg) as a yellow oil starting from **1d** (0.50 g, 1.36 mmol); IR (neat) 1611, 1552, 1512, 1441, 1332, 1303, 1248, 1175, 1109, 1034, 912, 819, 735 cm^{-1}; ^1H NMR ($CDCl_3$, 400 MHz, 298 K) δ 7.19-7.17 (m, 4H), 6.86-6.85 (m, 4H), 6.57 (dd, ^3J = 16.8 Hz, ^3J = 10.7 Hz, 1H, CH_2=<u>CH</u>), 6.07 (dd, ^3J = 16.8 Hz, ^2J = 2.2 Hz, 1H, (*E*)-<u>CH_2</u>=CH), 5.38 (dd, ^3J = 10.7 Hz, ^2J = 2.2 Hz, 1H, (*Z*)-<u>CH_2</u>=CH), 4.53-4.49 (m, 6H, CH_2<u>CH_2</u>NO_2 + 2×<u>CH_2</u>Ar), 3.80 (s, 6H, 2×OMe), 3.40 (t, ^3J = 7.3 Hz, 2H, <u>CH_2</u>CH_2NO_2); $^{13}C\{^1H\}$ NMR ($CDCl_3$, 100 MHz, 298 K) δ 168.3 (s, C_2), 159.2 (2×s), 147.5 (s, C_4), 129.4 (4×d), 128.7 (2×s), 126.7 (d, CH_2=<u>CH</u>), 117.3 (t, <u>CH_2</u>=CH), 115.2 (s, C_5), 114.1 (4×d), 75.6 (t, CH_2<u>CH_2</u>NO_2), 55.4 (2×q, 2×OMe), 52.7 (2×t, 2×<u>CH_2</u>Ar), 24.0 (t, <u>CH_2</u>CH_2NO_2); MS (EI, 70 eV) *m/z* (rel int) 439 (M^+, 100), 391 (31), 318 (100), 314 (37), 290 (43), 272 (74), 269 (40), 178 (45), 136 (65), 134 (57), 119 (99), 106 (55), 91 (91), 89 (40), 77 (100), 65 (49). HRMS (ESI) m/z: $[M + H]^+$ calcd. for $C_{23}H_{26}N_3O_4S$, 440.1639; found, 440.1632.

Preparation of 6-nitro-4,5,6,7-tetrahydrobenzo[*d*]thiazol-2-amine (8): A solution of the tetrahydrobenzothiazole **3db** (0.20 g, 0.46 mmol) in trifluoroacetic acid (5 ml) was stirred at 40 °C in an oil bath for 24 h. After cooling, CH_2Cl_2 (20 ml) was added, and the resulting solution was neutralized with a saturated aqueous solution of $NaHCO_3$. The aqueous phase was extracted with CH_2Cl_2 (3 × 20 ml) and the organic extracts were dried over anhydrous $MgSO_4$. After filtration of the inorganic salts, the solvent was removed under reduced pressure and the residue purified by silica-gel chromatography eluting with AcOEt (R_f = 0.25) to give **8** in 85% yield (77 mg); mp 191-192 ºC (colorless prims, $CHCl_3/Et_2O$); IR (nujol) 3446, 3260, 3088, 1620, 1594, 1545, 1526, 1104 cm^{-1}; ^1H NMR ($CDCl_3$, 600 MHz, 298 K) δ 4.88 (br s, 2H, NH_2), 4.80 (td, ^3J = 8.6 Hz, ^3J = 4.5 Hz, 1H, H_6), 3.29 (dddd, ^2J = 15.9 Hz, ^3J = 8.1 Hz, ^5J = 2.3 Hz, ^5J = 1.9 Hz, 1H, H_{7b}), 3.19 (dd, ^2J = 15.9 Hz, ^3J = 5.6 Hz, 1H, H_{7a}), 2.79-2.75 (m, 1H, H_{4b}), 2.70 (dddt, ^2J = 14.9 Hz, ^3J = 10.6 Hz, ^3J = 6.8 Hz, ^5J = 2.1 Hz, 1H, H_{4a}), 2.45-2.39 (m, 2H, H_5); $^{13}C\{^1H\}$ NMR ($CDCl_3$, 150 MHz, 298 K) δ 166.2 (s, C_2), 144.5 (s, C_{3a}), 113.4 (s, C_{7a}), 81.2 (d, C_6), 27.6 (t, C_7), 27.5 (t, C_5), 24.3 (t, C_4); MS (EI, 70 eV) *m/z* (rel int) 199 (M^+, 98), 152 (99), 126 (52), 119 (65), 110 (100), 93 (97), 82 (50), 77 (62), 70 (100), 66 (99), 60 (58). HRMS (ESI) m/z: $[M + H]^+$ calcd. for $C_7H_{10}N_3O_2S$, 200.0488; found, 200.0481.

Preparation of 4,5,6,7-tetrahydrobenzo[*d*]thiazol-2,6-diamine (4):[7] Pd/C 10% (0.05 g) was added to a solution of the tetrahydrobenzothiazole **8** (0.10 g, 0.50 mmol) in MeOH (15 ml). The

reaction mixture was stirred at 25 ºC for 5 d under a hydrogen atmosphere. The suspension was filtered, and the solvent removed under reduced pressure. The residue was purified by chromatography by using deactivated silica gel and eluting with AcOEt (R_f = 0.15). Silica gel was deactivated by flushing with 10% triethylamine in *n*-hexane and then, washing with *n*-hexane prior to use; yield 62% (53 mg); mp > 300 ºC (colorless prims, MeOH); IR (nujol) 3383, 1602, 1072, 1035, 874, 721 cm^{-1}; ^{1}H NMR (CD_3OD, 400 MHz, 298 K) δ 3.56-3.51 (m, 1H), 2.98 (dd, ^2J = 15.0 Hz, ^3J = 4.0 Hz, 1H), 2.63-2.59 (m, 3H), 2.16-2.14 (m, 1H), 1.95-1.85 (m, 1H); $^{13}C\{^1H\}$ NMR (DMSO-d_6, 100 MHz, 298 K) δ 165.8 (s, C_2), 144.2 (s, C_{3a}), 113.0 (s, C_{7a}), 47.6 (d, C_6), 32.04 (t), 32.00 (t), 24.8 (t); MS (EI, 70 eV) *m/z* (rel int) 169 (M^+, 30), 152 (57), 127 (44), 126 (100).

1.3. Configurational assignment of 3aa, 3ba and 7bb

The X-ray structure of **3ba** confirmed the *trans* relative position of the nitro and phenyl groups at the six-membered ring of the tetrahydrobenzothiazole. The relative stereochemistry of the stereocenters in **3aa** was established based on either the values of the coupling constants between the protons at the six-membered ring and on significant cross-peaks found in its ^{1}H,^{1}H-NOESY spectrum.

The most valuable information to stablish the relative configuration at 5-, 6- and 7-positions of the tetrahydrobenzothiazole ring within **3aa** rely on the coupling constant values of the protons placed at these positions. Thus, the coupling constant between H_5/H_6 and H_6/H_7 are 11.6 and 9.8 Hz, respectively, indicating that these three protons adopt a 1,2-transdiaxial arrangement (Figure S1).[8, 9] Notably, the coupling constant between H_{4a}/H_5 is 11.6 Hz showing that these two protons are either in a 1,2-transdiaxial position. On the contrary, the coupling constant between H_{4b}/H_5 is only 5.8 Hz. The most significant contacts in the NOESY spectrum of **3aa** are symbolized as red arrows in Figure S1 (Figures S2 and S3 display the corresponding regions of the NOESY spectrum where these contacts appear). The cross peak observed between H_5 and H_7 joined to the closed contact between H_{4a} and H_6 agree well with the assignment based on the coupling constants.

As was to be expected based on the X-ray structure of **3ba**, both couple of protons H_{5b}/H_6 (^3J = 10.4 Hz) and H_6/H_7 (^3J = ≈ 7.7-8.0 Hz) are typical of protons in a relative 1,2-transdiaxial arrangement. Moreover, NOESY contacts were found between the couple of protons H_{5b}/H_7 and H_{4a}/H_6 (Figures S4-6).

The determination of the relative stereochemistry of **7bb** was a challenge. However, the values of the coupling constants between H_4/H_{5a} (^3J = 6.0 Hz), H_4/H_{5b} (^3J = 5.5 Hz) and H_{5b}/H_6 (^3J = 8.5 Hz) were the most useful information to **tentatively assign** the relative *trans* position of the two substituents at the six-membered ring (Figure S7). In agreement with the *trans*-relative arrangement of the substituent at the 4- and 6-positions of the tetrahydrobenzothiazole ring, NOE contacts were found relating protons $H_{4'}/H_{5a}$, H_4/H_{5b} and H_6/H_{7a}. Significantly, no cross peak was found relating H_4 and H_6 indicating that these two protons are **probably** at opposite sides of the six-membered ring (Figures S7-9).

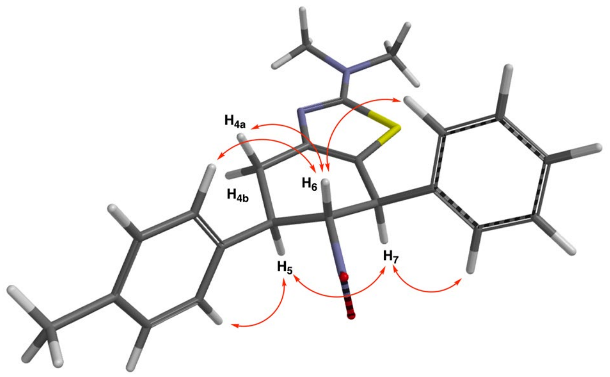

Figure S1. Most relevant NOE contacts on the geometry-optimized structure of **3aa**.[10]

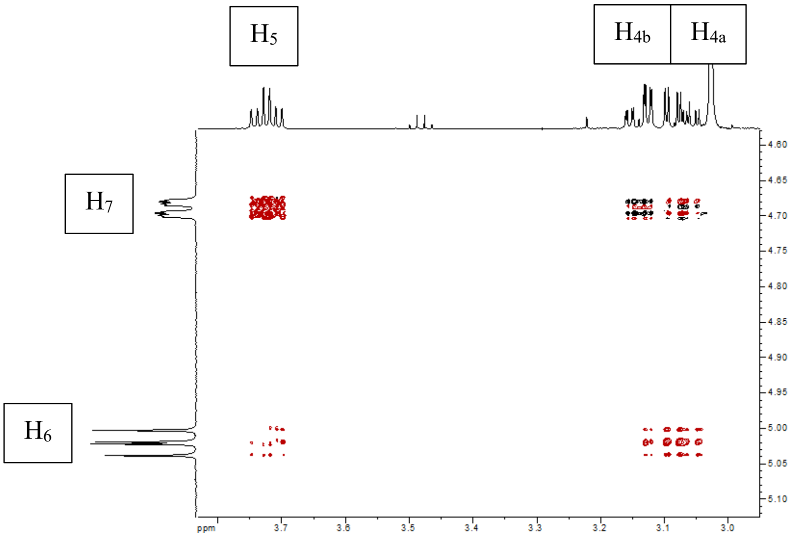

Figure S2. Selected region of the NOESY spectrum (600 MHz, $CDCl_3$) of **3aa**.

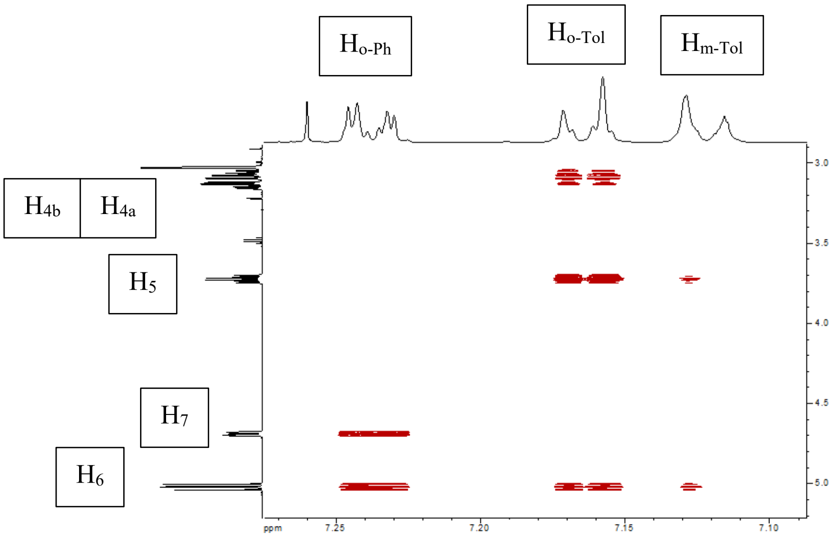

Figure S3. Selected region of the NOESY spectrum (600 MHz, $CDCl_3$) of **3aa**.

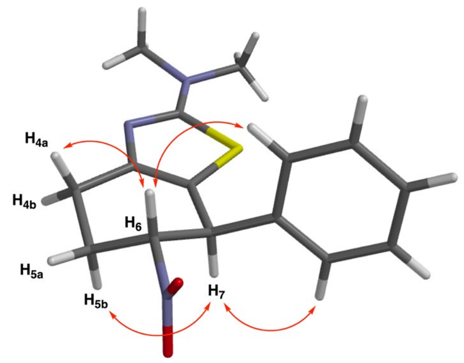

Figure S4. Most relevant NOE contacts on the X-ray structure of **3ba**.

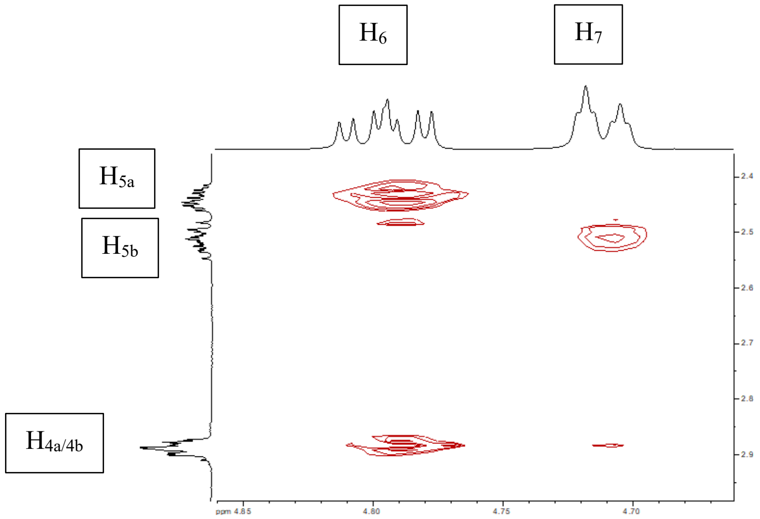

Figure S5. Selected region of the NOESY spectrum (600 MHz, $CDCl_3$) of **3ba**.

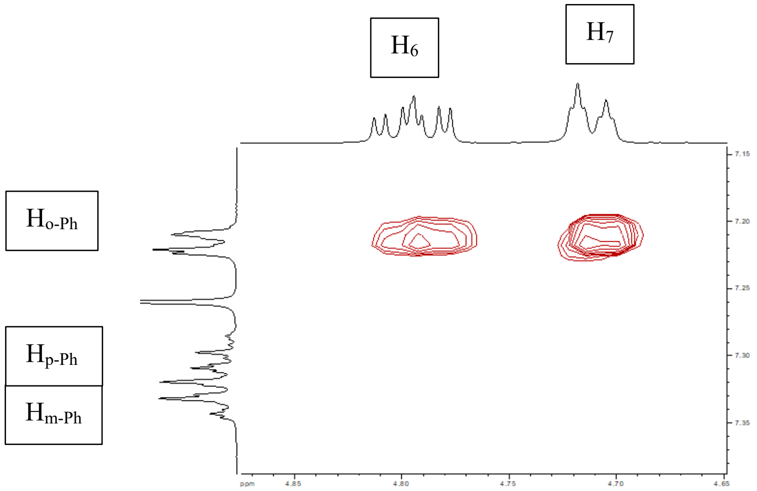

Figure S6. Selected region of the NOESY spectrum (600 MHz, $CDCl_3$) of **3ba**.

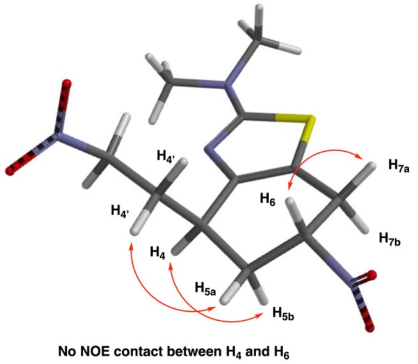

Figure S7. Most relevant NOE contacts on the X-ray structure of **7bb**.[10]

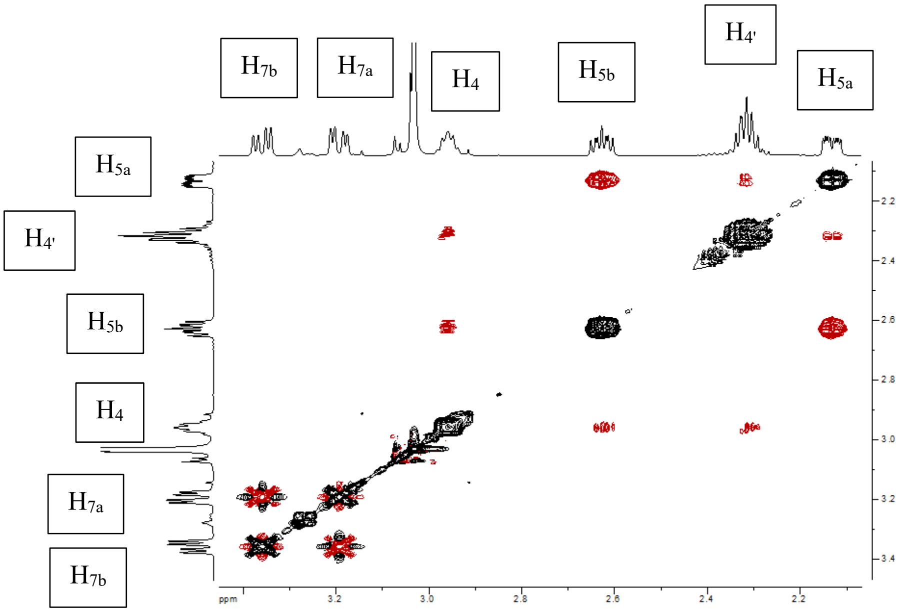

Figure S8. Selected region of the NOESY spectrum (600 MHz, $CDCl_3$) of **7bb**.

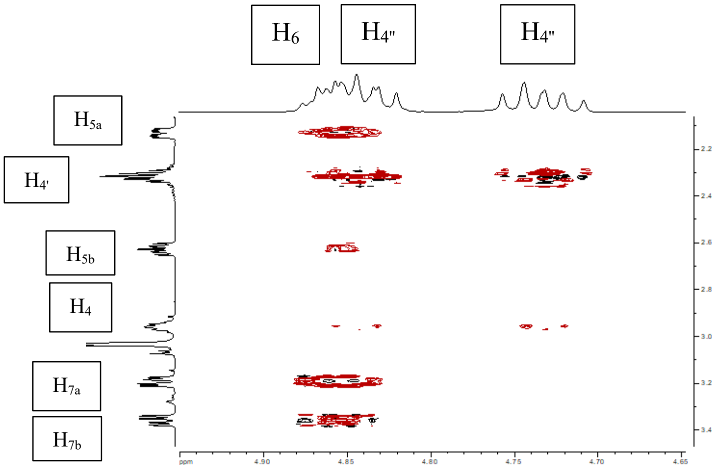

Figure S9. Selected region of the NOESY spectrum (600 MHz, $CDCl_3$) of **7bb**.

1.4. Crystal data and structure refinement for 3ba

Table S1. Crystal data and structure refinement for **3ba**.

Identification code	III-APV-30-F4_sp_a	
Empirical formula	C15 H17 N3 O2 S	
Formula weight	303.38	
Temperature	100(2) K	
Wavelength	0.71073 Å	
Crystal system	Monoclinic	
Space group	$P2_1$	
Unit cell dimensions	a = 5.6533(3) Å	α= 90°.
	b = 18.7332(10) Å	β= 97.801(2)°.
	c = 14.1142(8) Å	γ = 90°.
Volume	1480.92(14) $Å^3$	
Z	4	
Density (calculated)	1.361 Mg/m^3	
Absorption coefficient	0.227 mm^{-1}	
F(000)	640	
Crystal size	0.270 x 0.220 x 0.130 mm^3	
Theta range for data collection	2.174 to 30.614°.	
Index ranges	-8<=h<=8, -26<=k<=26, -20<=l<=20	
Reflections collected	115749	
Independent reflections	9051 [R(int) = 0.0520]	
Completeness to theta = 25.242°	99.9 %	
Absorption correction	Semi-empirical from equivalents	
Max. and min. transmission	0.7461 and 0.7187	
Refinement method	Full-matrix least-squares on F^2	
Data / restraints / parameters	9051 / 1 / 383	
Goodness-of-fit on F^2	1.046	
Final R indices [I>2sigma(I)]	R1 = 0.0349, wR2 = 0.0719	
R indices (all data)	R1 = 0.0431, wR2 = 0.0748	
Absolute structure parameter	0.017(11)	
Largest diff. peak and hole	0.241 and -0.295 e.$Å^{-3}$	

Table S2. Hydrogen bonds for **3ba** [Å and °].

D-H...A	d(D-H)	d(H...A)	d(D...A)	<(DHA)
C(6)-H(6)...O(2)#1	1.00	2.51	3.401(3)	148.1
C(9)-H(9B)...N(23)#2	0.98	2.47	3.436(3)	168.1
C(25)-H(25B)...O(1)#3	0.99	2.40	3.223(3)	140.7
C(26)-H(26)...O(21)#1	1.00	2.52	3.423(3)	149.7

Symmetry transformations used to generate equivalent atoms:

#1 x+1,y,z   #2 x,y,z+1   #3 -x,y-1/2,-z

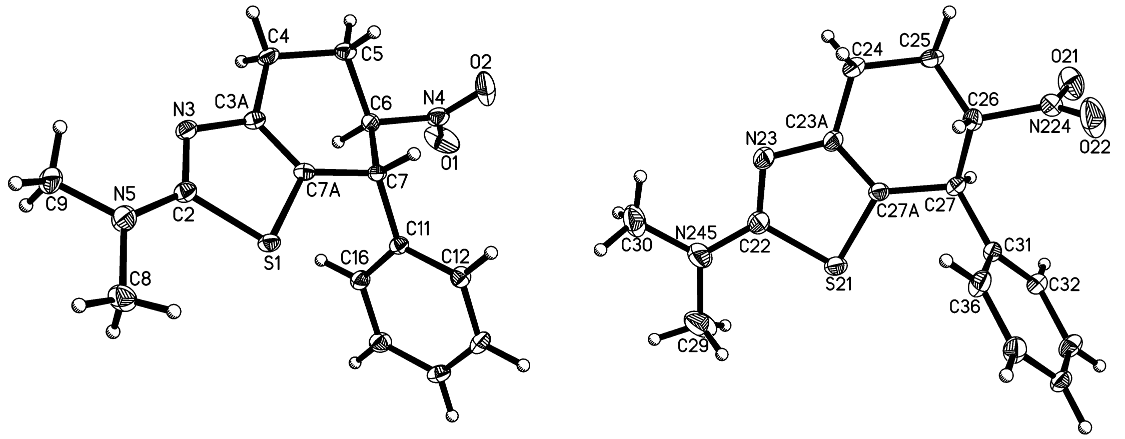

C6 is *R*, C7 is *S*.   C26 is *S*, C27 is *R*.

Figure S10. Structures of the two molecules of **3ba** present in the crystal with thermal ellipsoids drawn at 50% probability.

1.5. Copies of the ^1H NMR and $^{13}C\{^1H\}$ NMR spectra of all new compounds

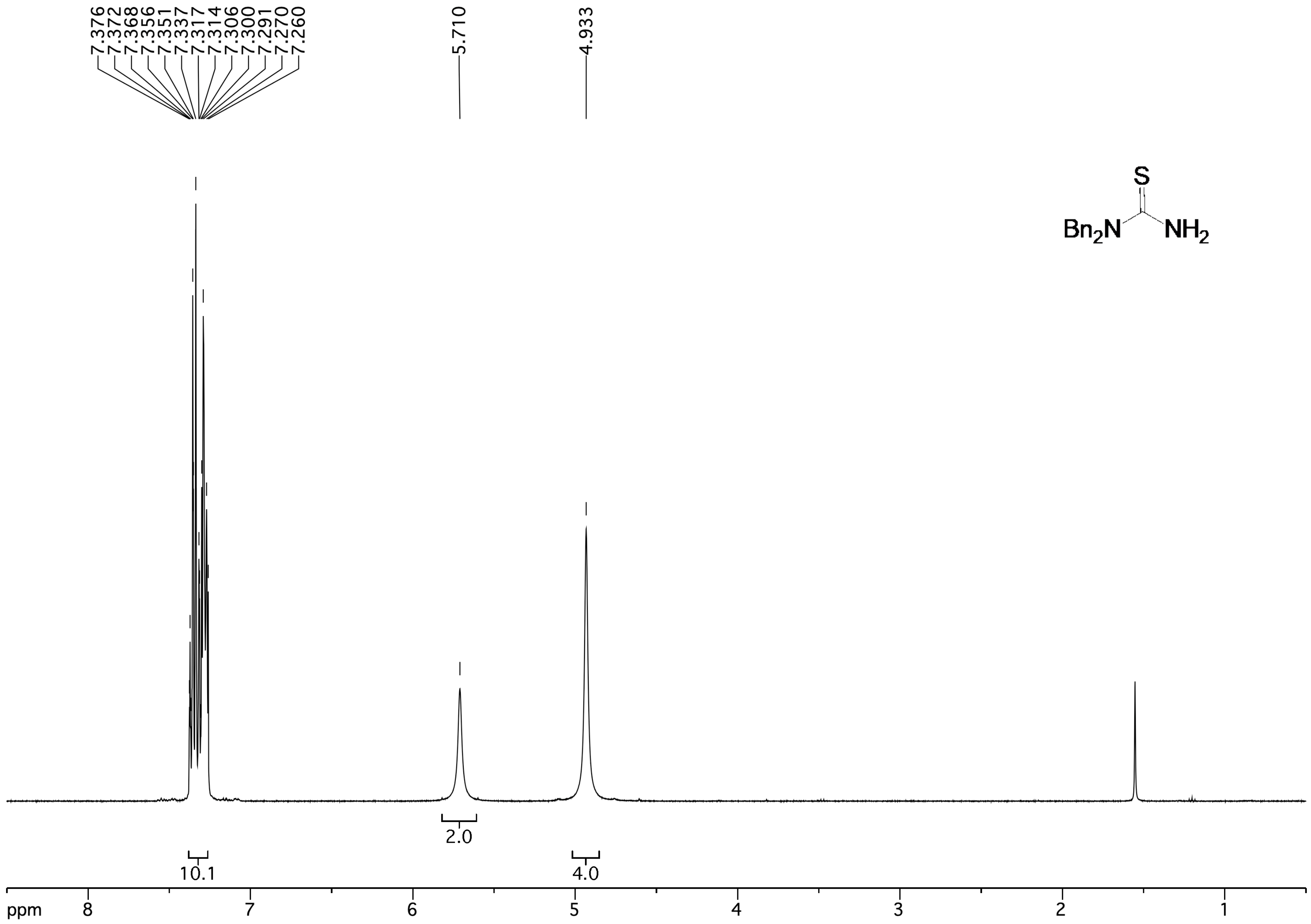

^{1}H NMR ($CDCl_3$, 400 MHz) of *N*,*N*'-dibenzylthiourea.

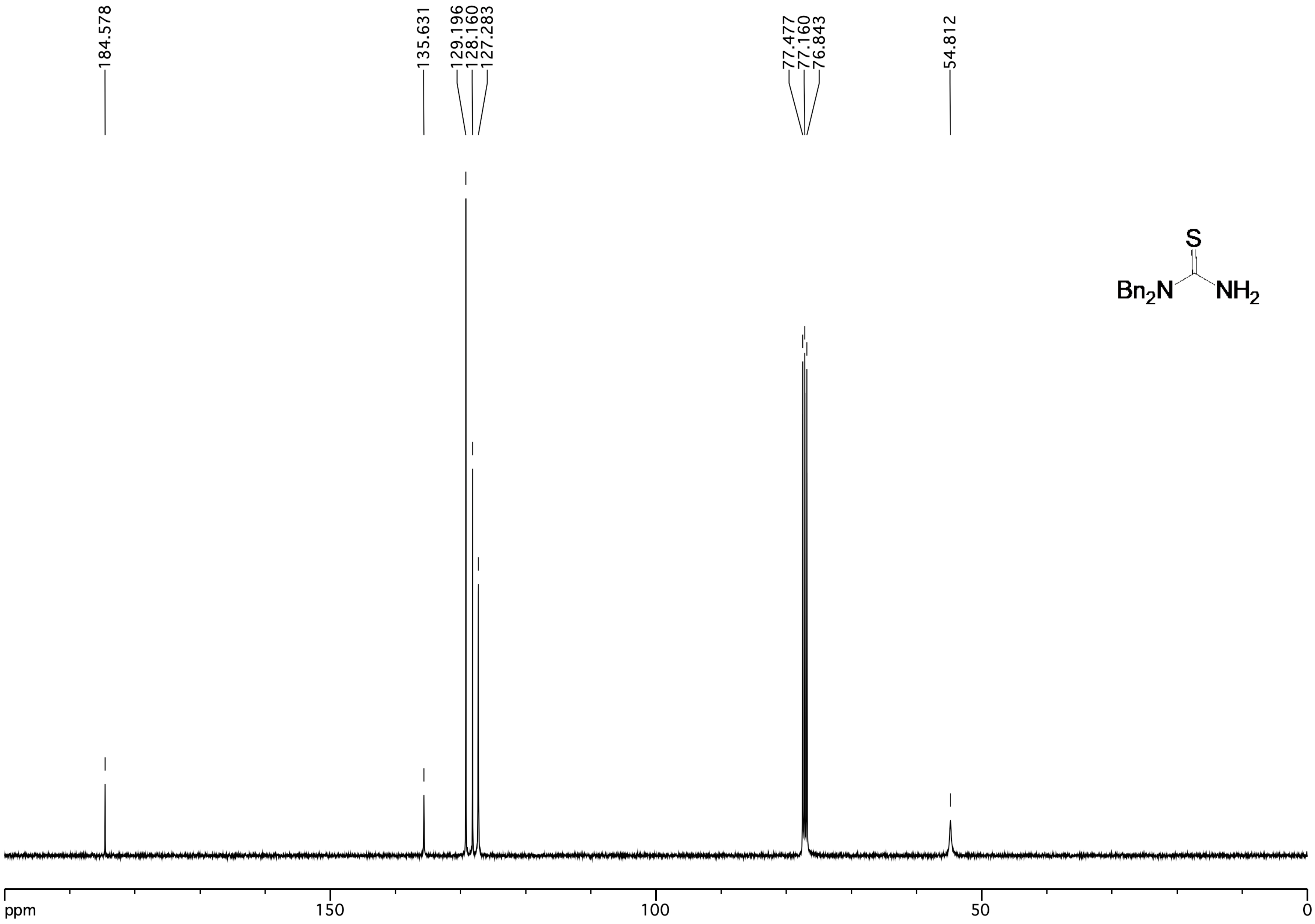

$^{13}C\{^1H\}$ NMR ($CDCl_3$, 400 MHz) of *N*,*N*'-dibenzylthiourea.

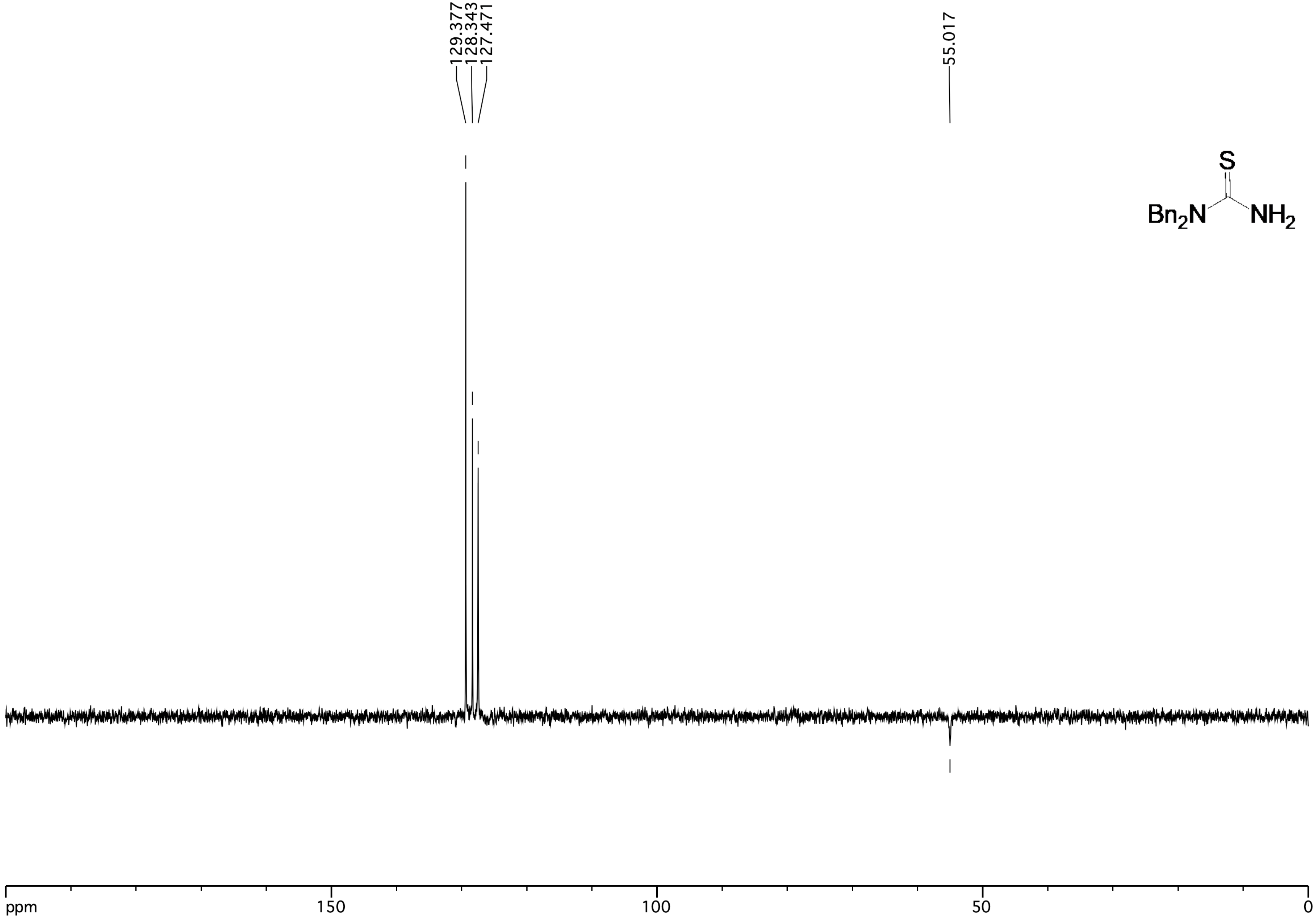

DEPT-135 ($CDCl_3$, 100 MHz) of *N*,*N*'-dibenzylthiourea.

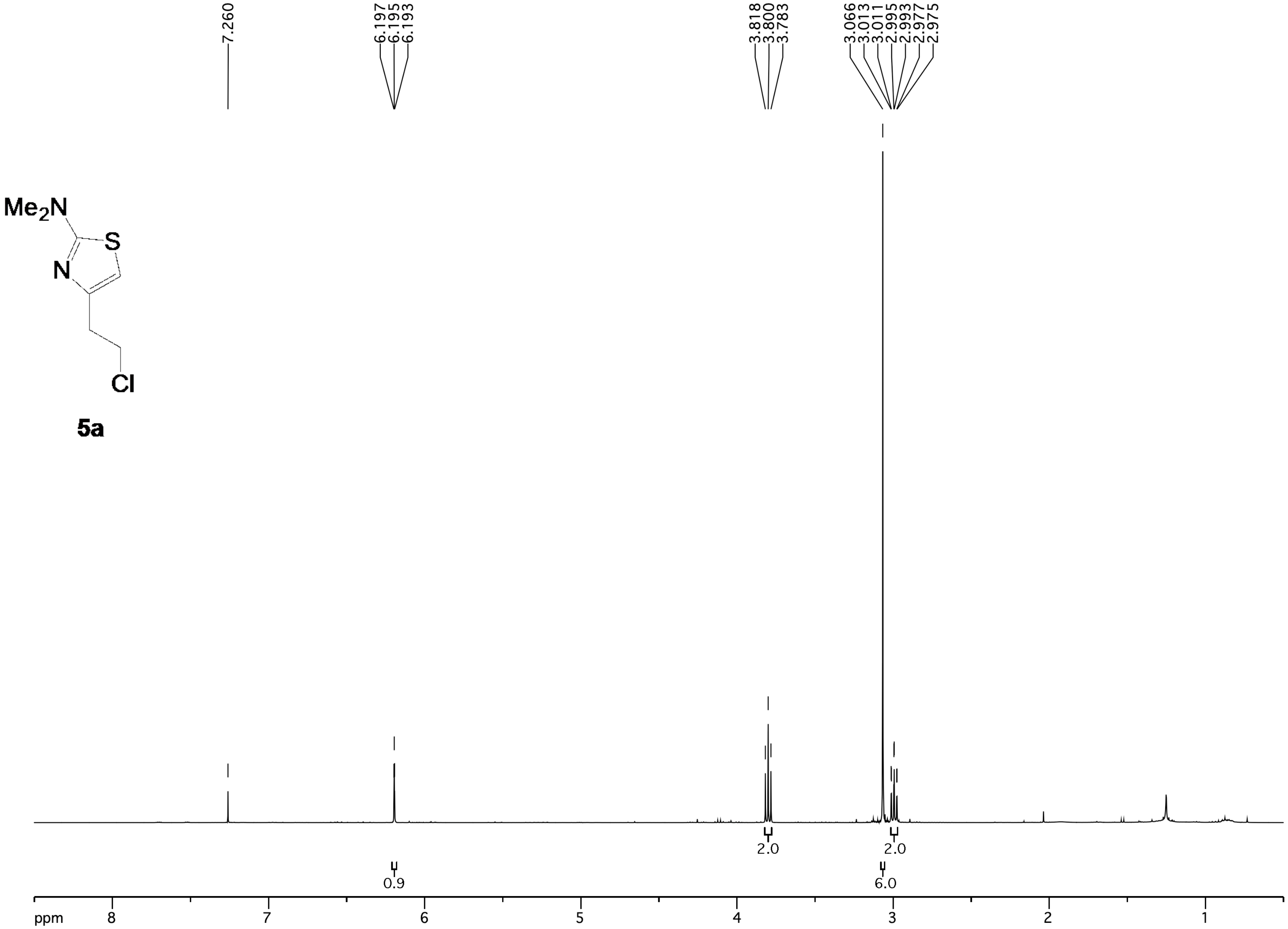
5a
7.260
6.197
6.195
6.193
3.818
3.800
3.783
3.066
3.013
3.011
2.995
2.993
2.977
2.975
2.0
2.0
0.9
6.0
ppm
8
7
6
5
4
3
2
1

^{1}H NMR ($CDCl_3$, 400 MHz) of compound **5a**.

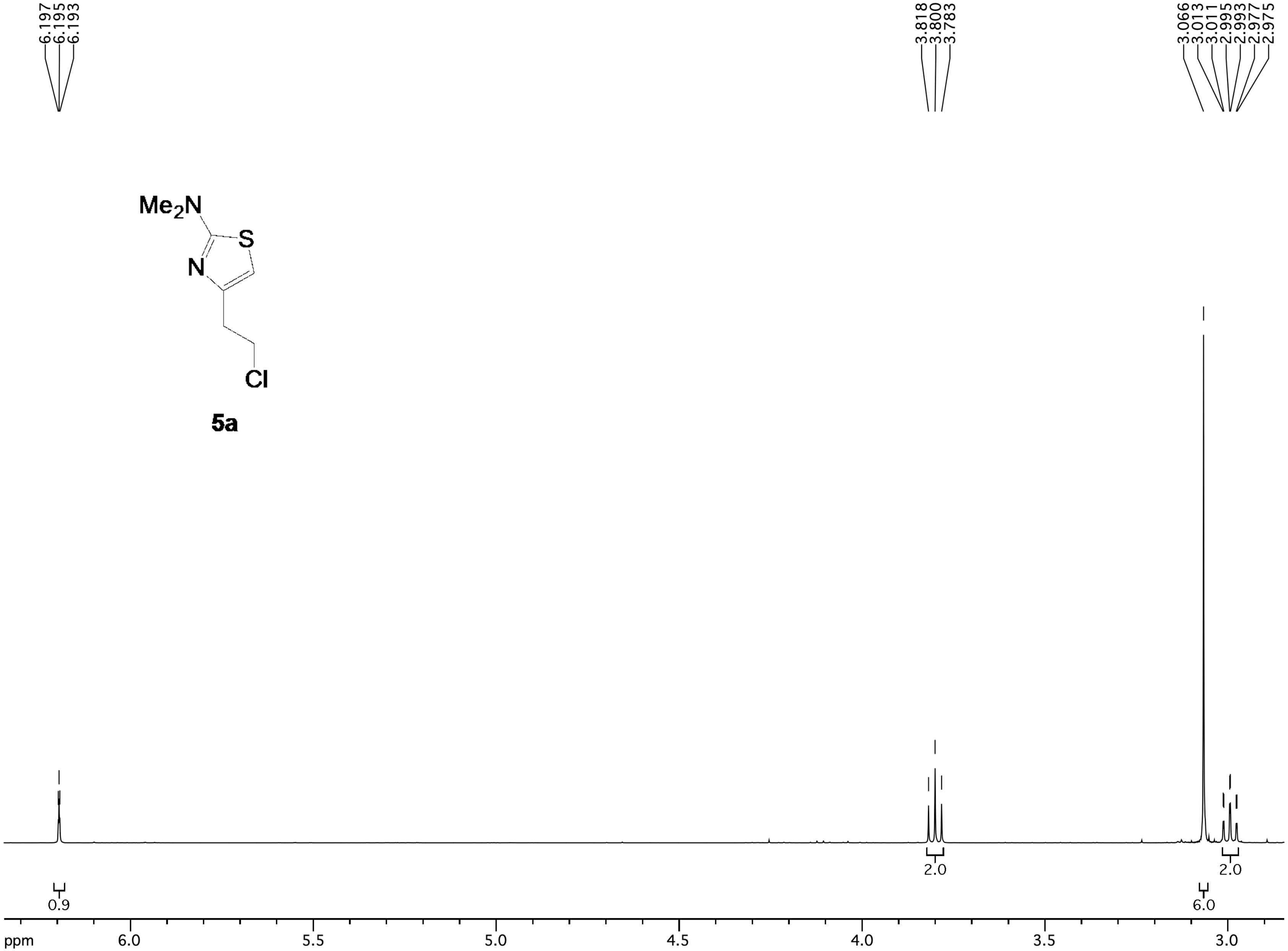

^{1}H NMR (CDCl3, 400 MHz) of compound **5a** (expansion).

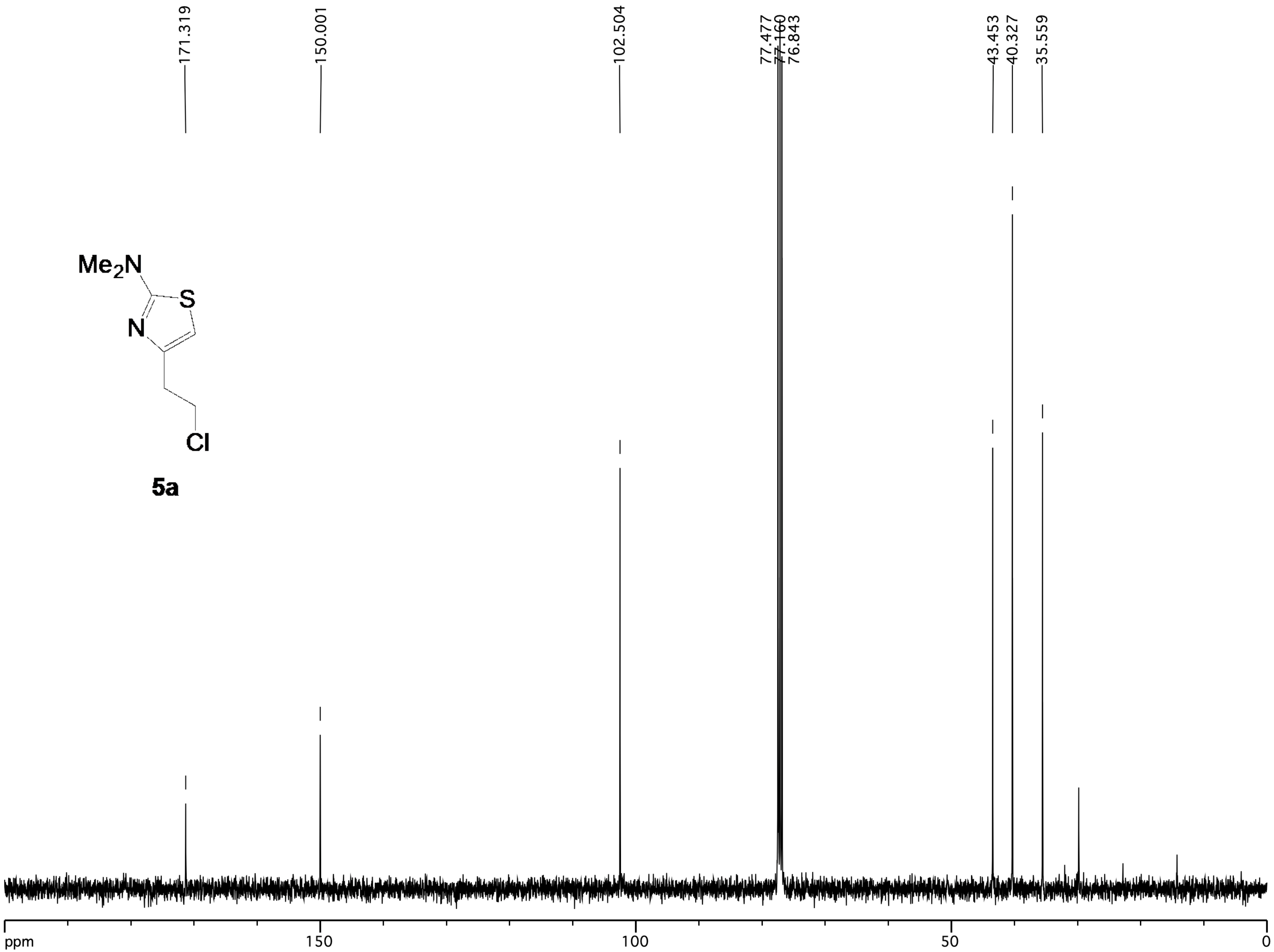

$^{13}C\{^{1}H\}$ NMR (CDCl3, 100 MHz) of compound **5a**.

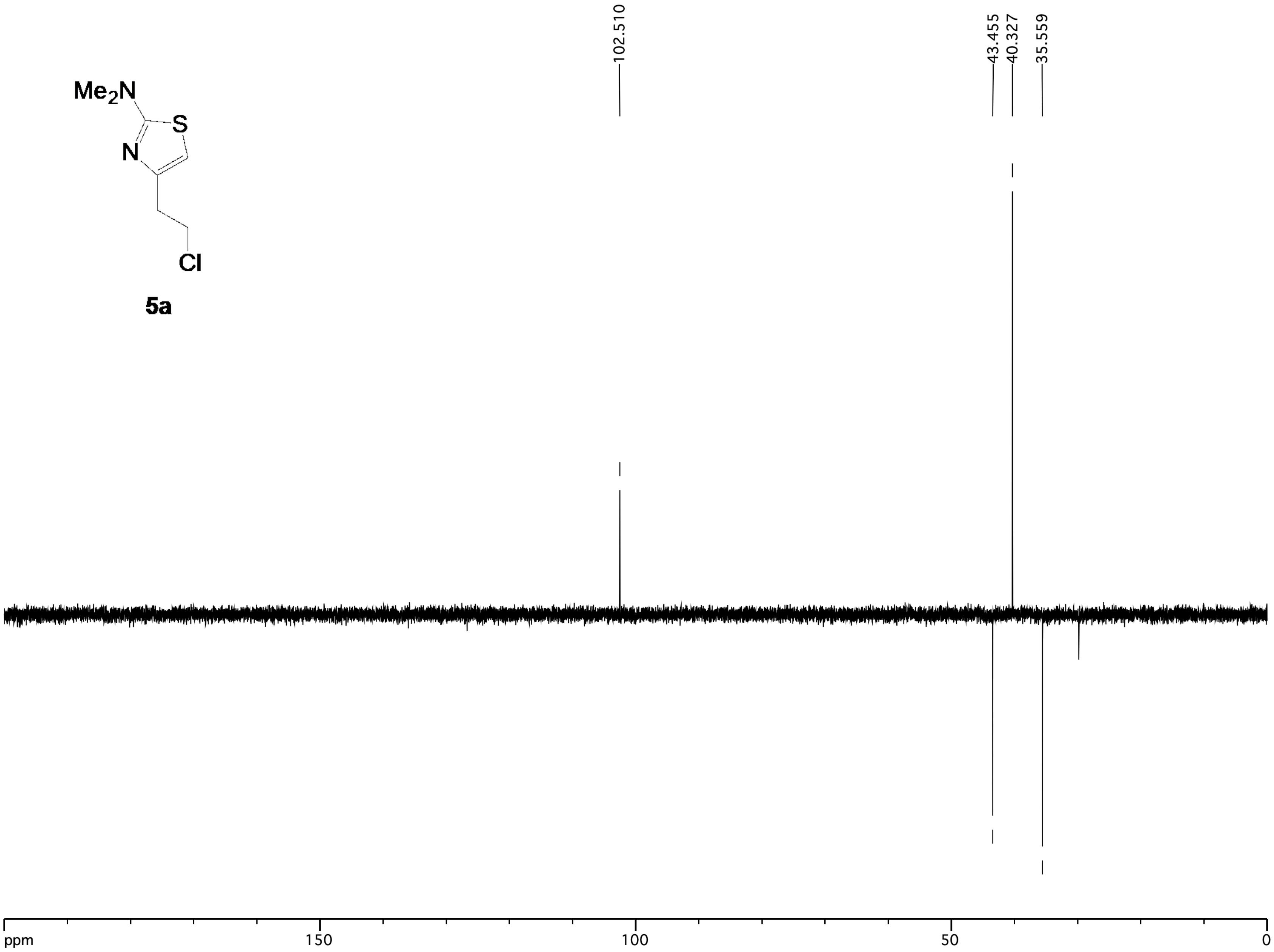

DEPT-135 ($CDCl_3$, 100 MHz) of compound **5a**.

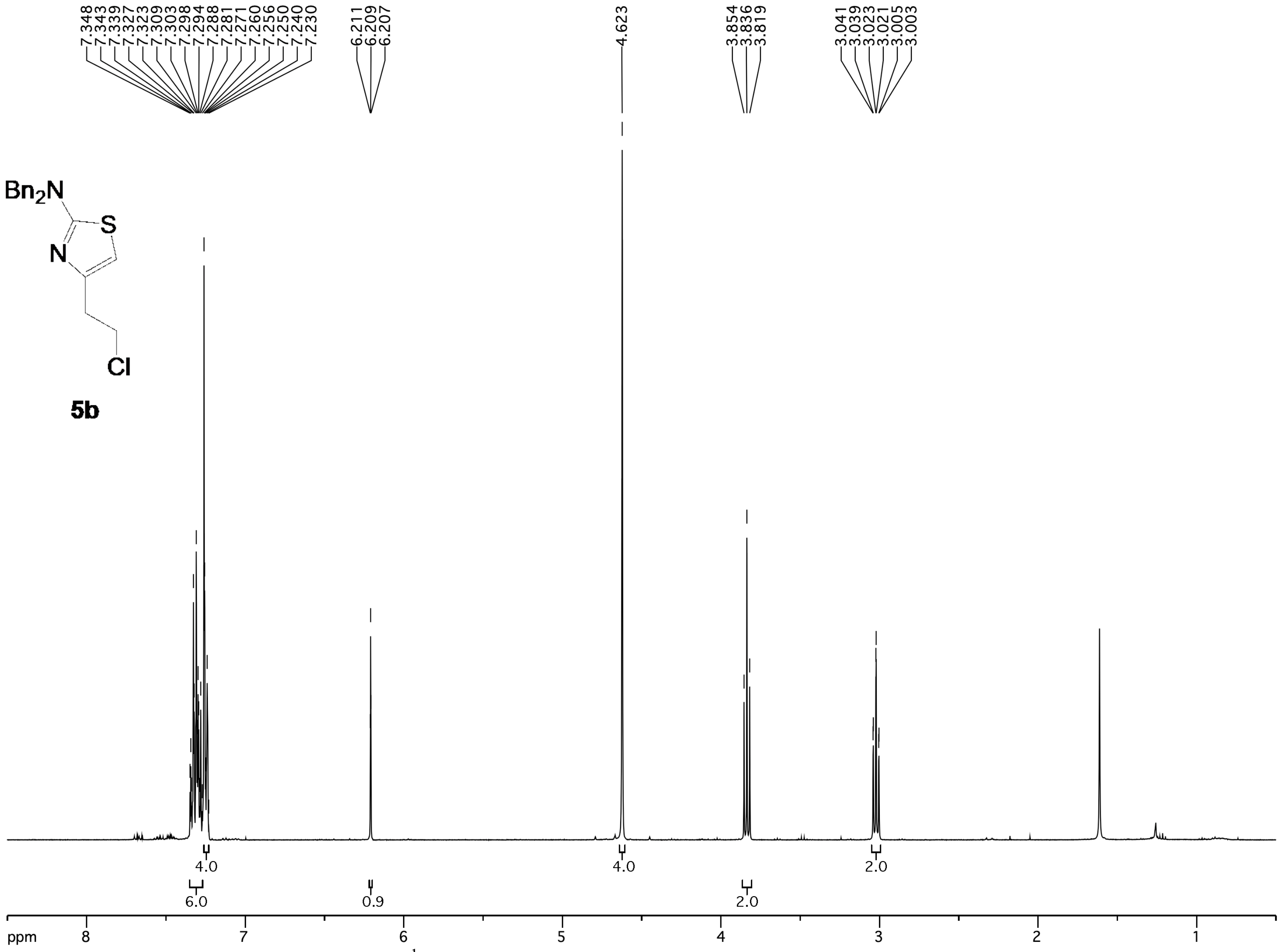

^{1}H NMR (CDCl$_3$, 400 MHz) of compound **5b**.

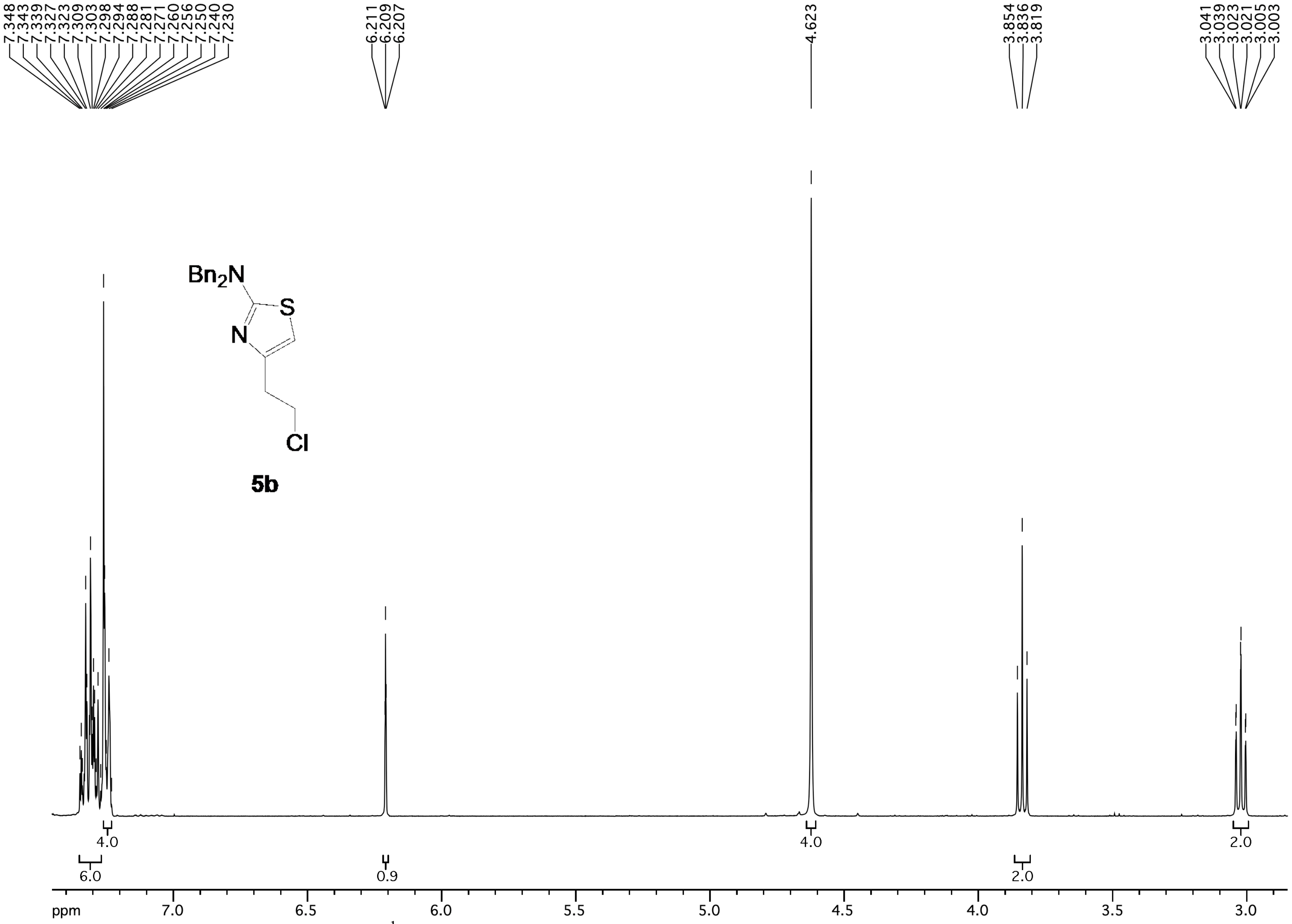

^{1}H NMR ($CDCl_3$, 400 MHz) of compound **5b** (expansion).

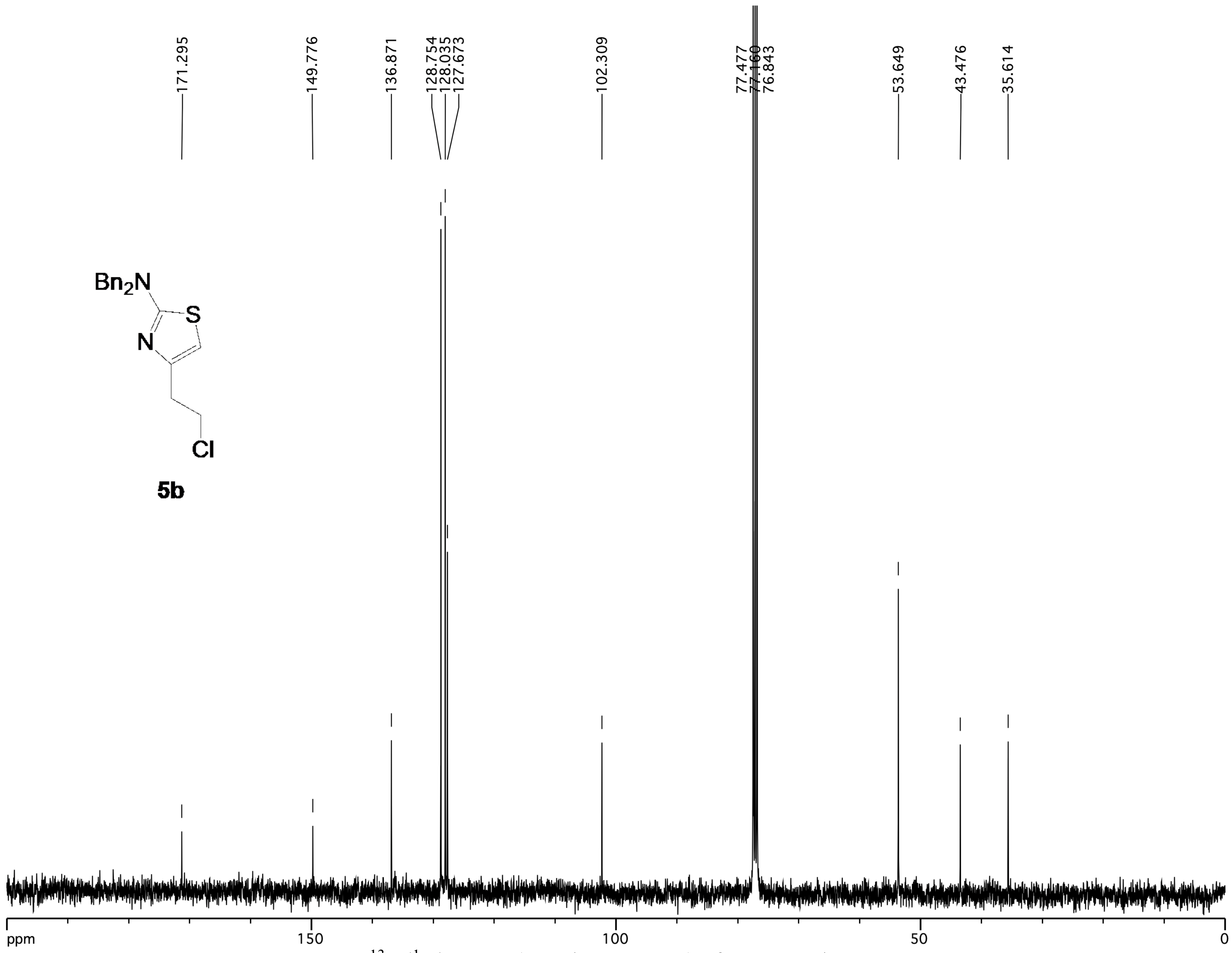

$^{13}C\{^{1}H\}$ NMR ($CDCl_3$, 100 MHz) of compound **5b**.

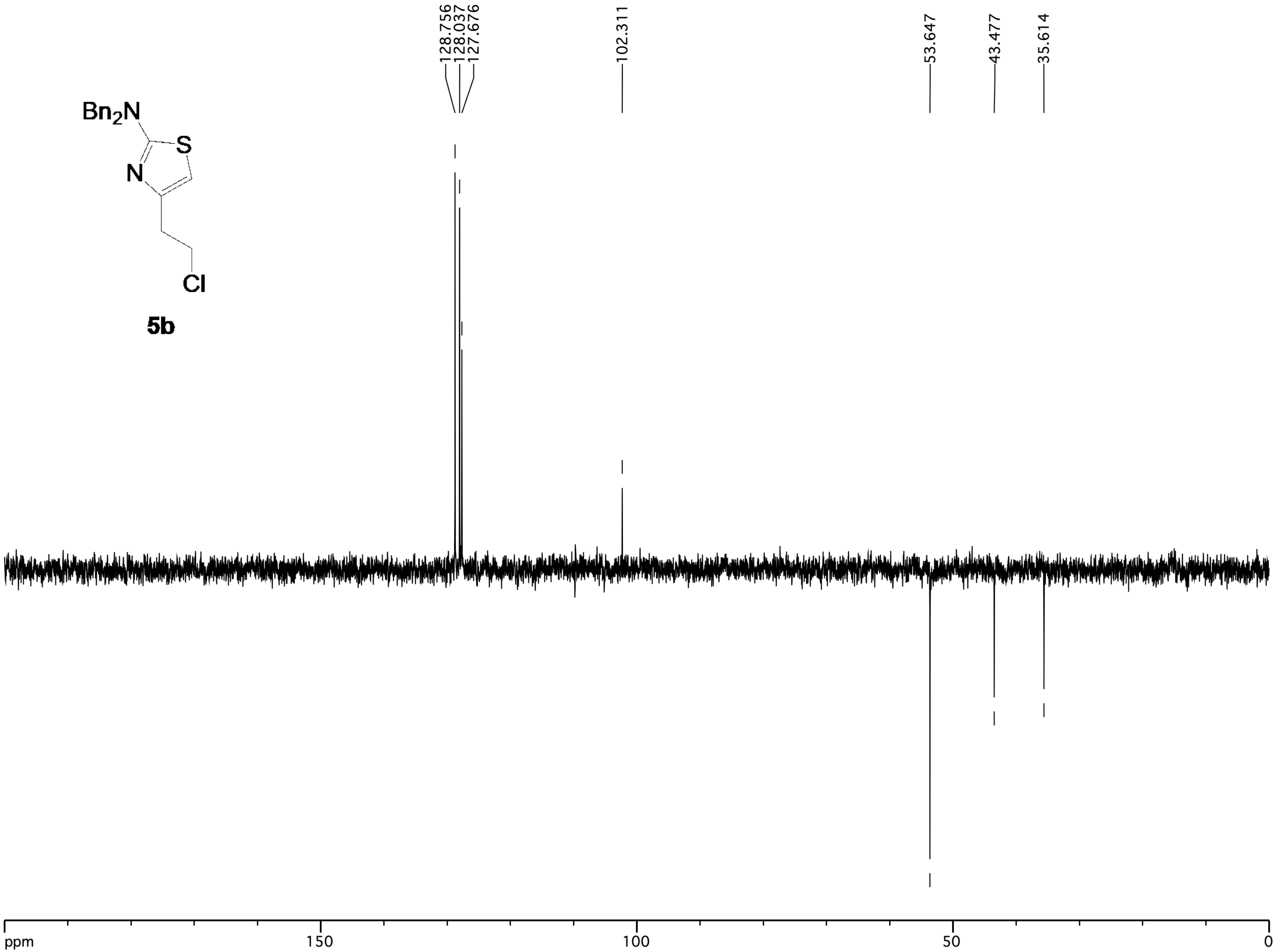

DEPT-135 ($CDCl_3$, 100 MHz) of compound **5b**.

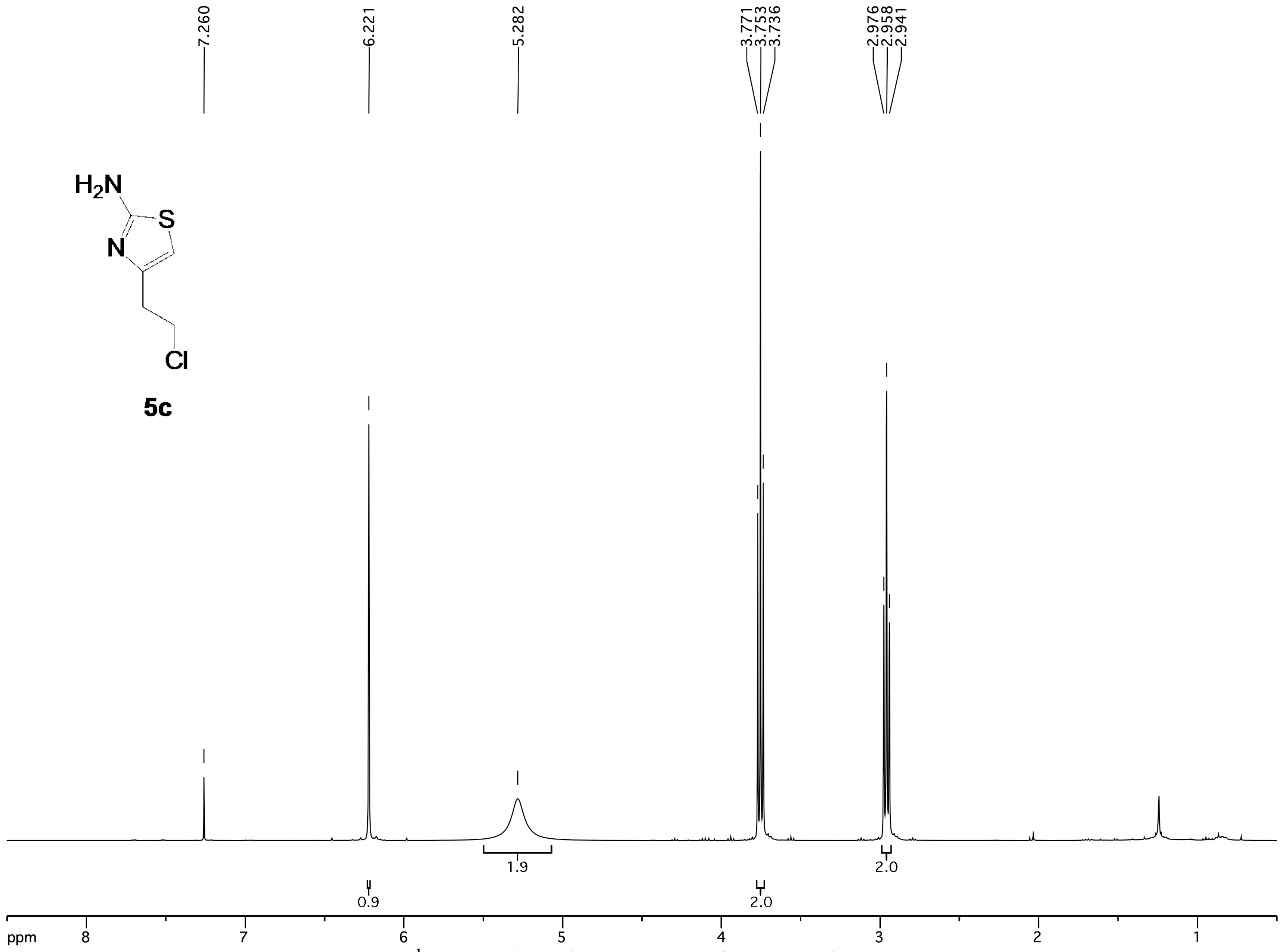

^{1}H NMR ($CDCl_3$, 400 MHz) of compound **5c**.

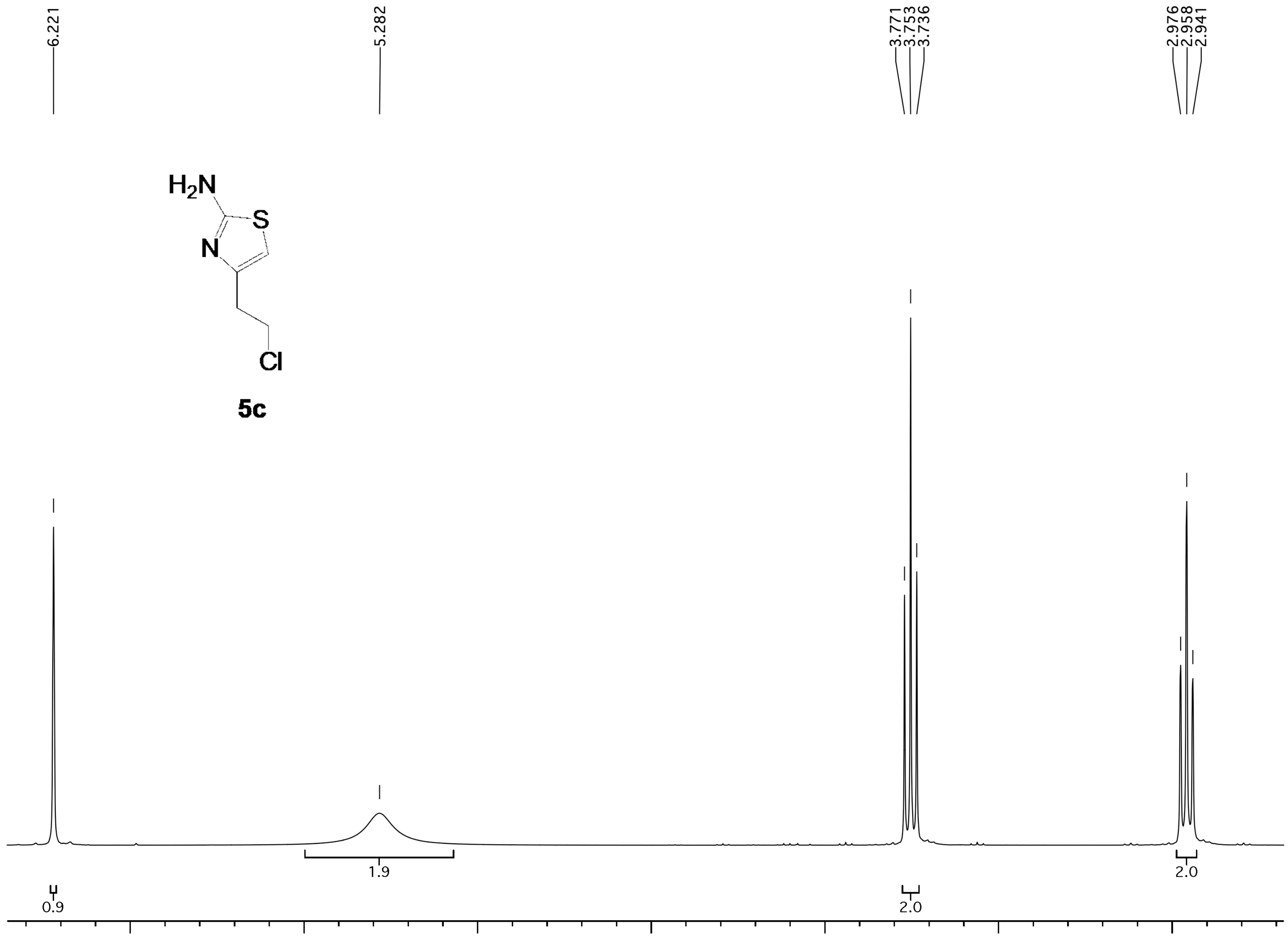

^{1}H NMR ($CDCl_3$, 400 MHz) of compound **5c** (expansion).

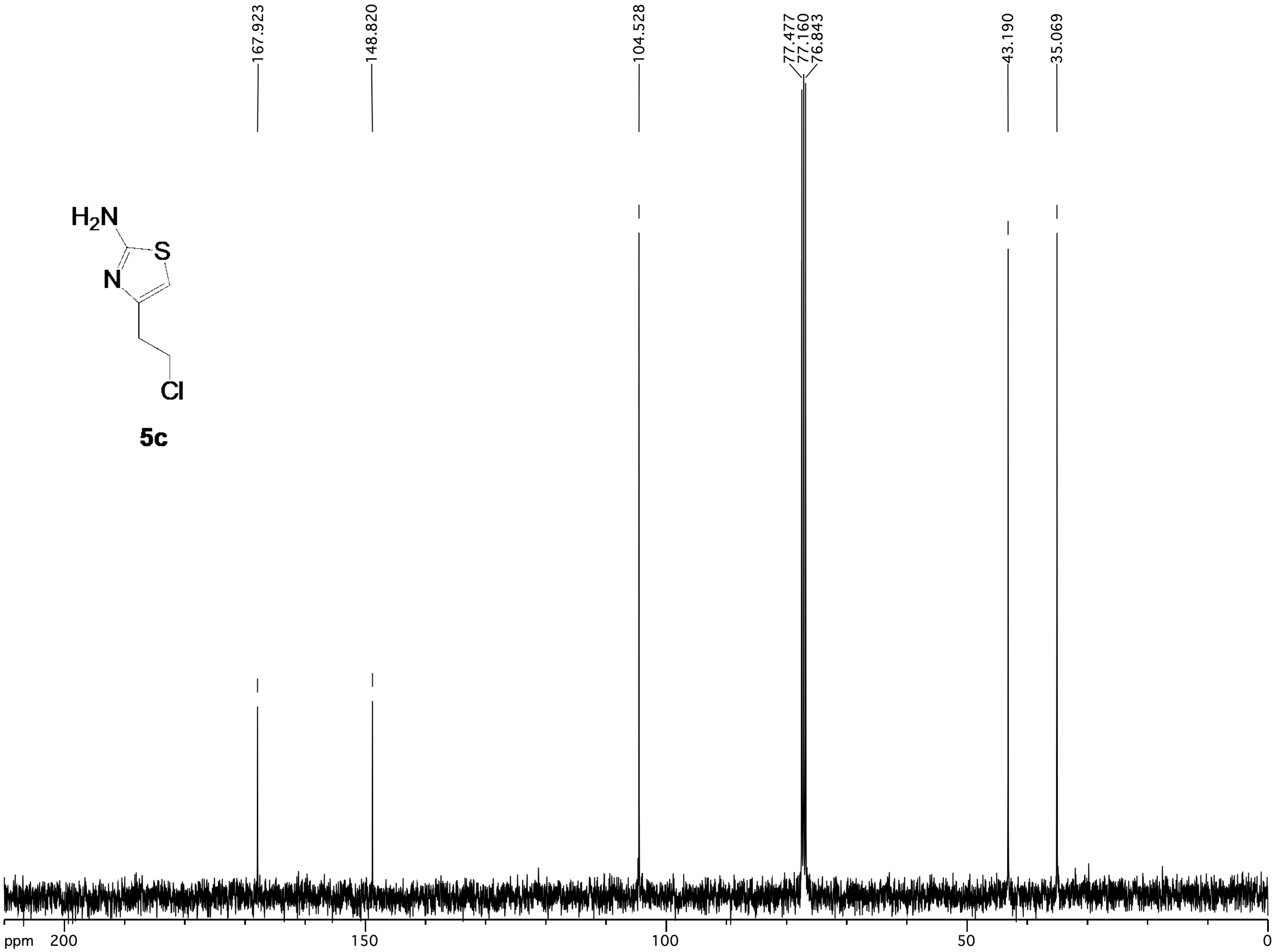

$^{13}C\{^1H\}$ NMR ($CDCl_3$, 100 MHz) of compound **5c**.

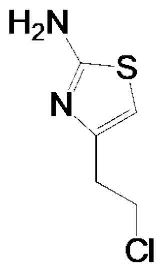

5c

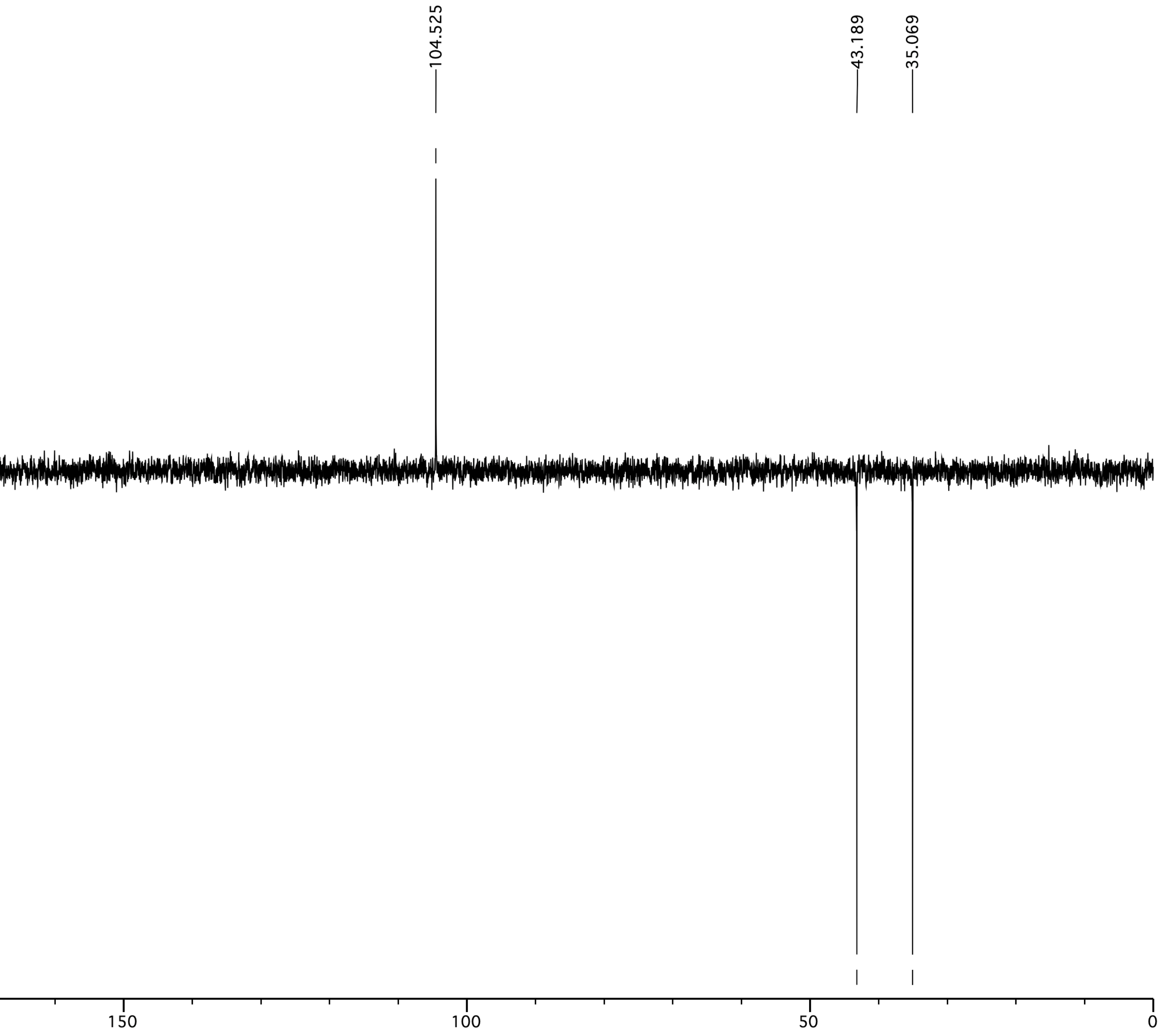

DEPT-135 ($CDCl_3$, 100 MHz) of compound **5c**.

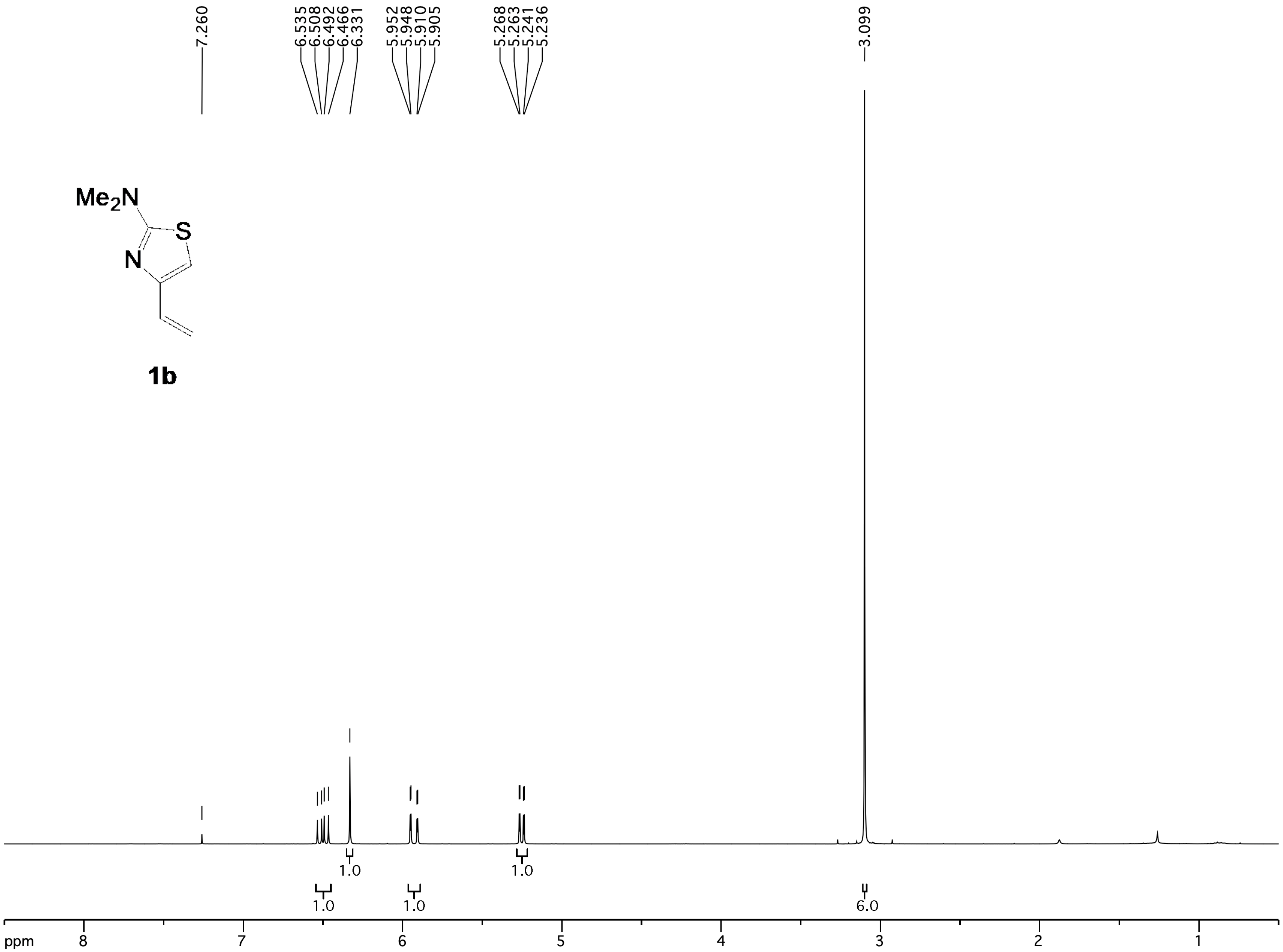

^{1}H NMR (CDCl3, 400 MHz) of compound **1b**.

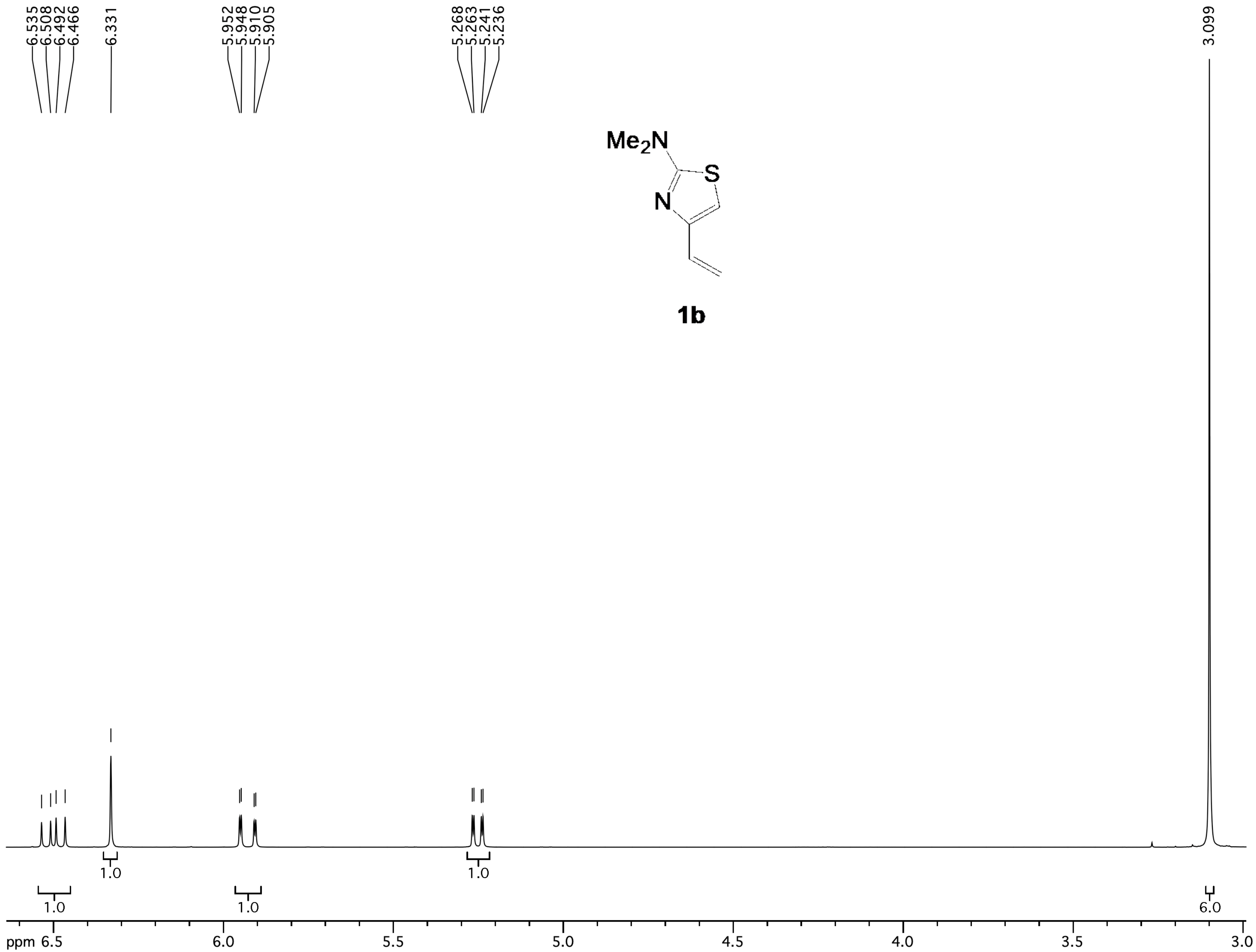

^{1}H NMR ($CDCl_3$, 400 MHz) of compound **1b** (expansion).

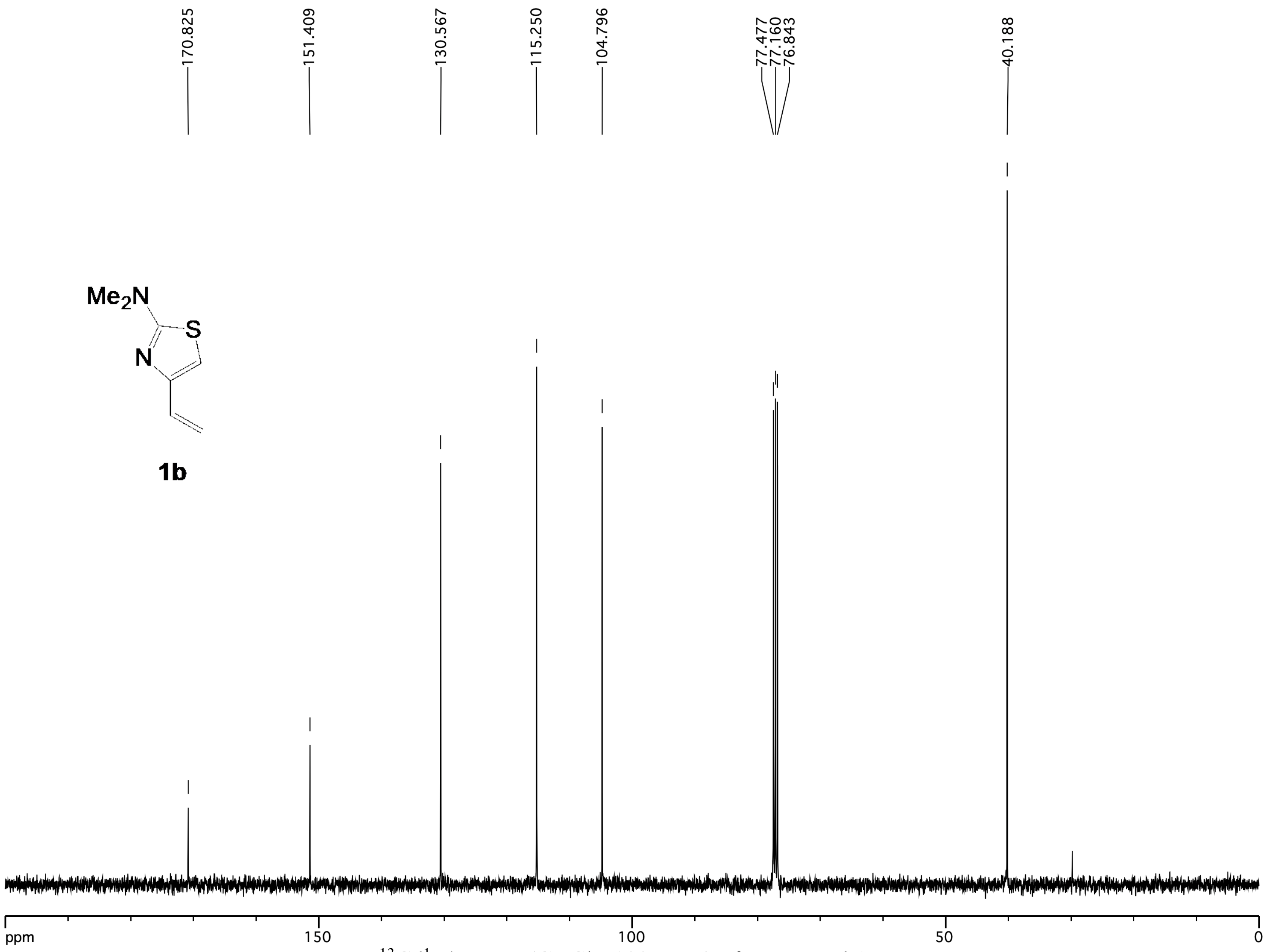

$^{13}C\{^1H\}$ NMR ($CDCl_3$, 100 MHz) of compound **1b**.

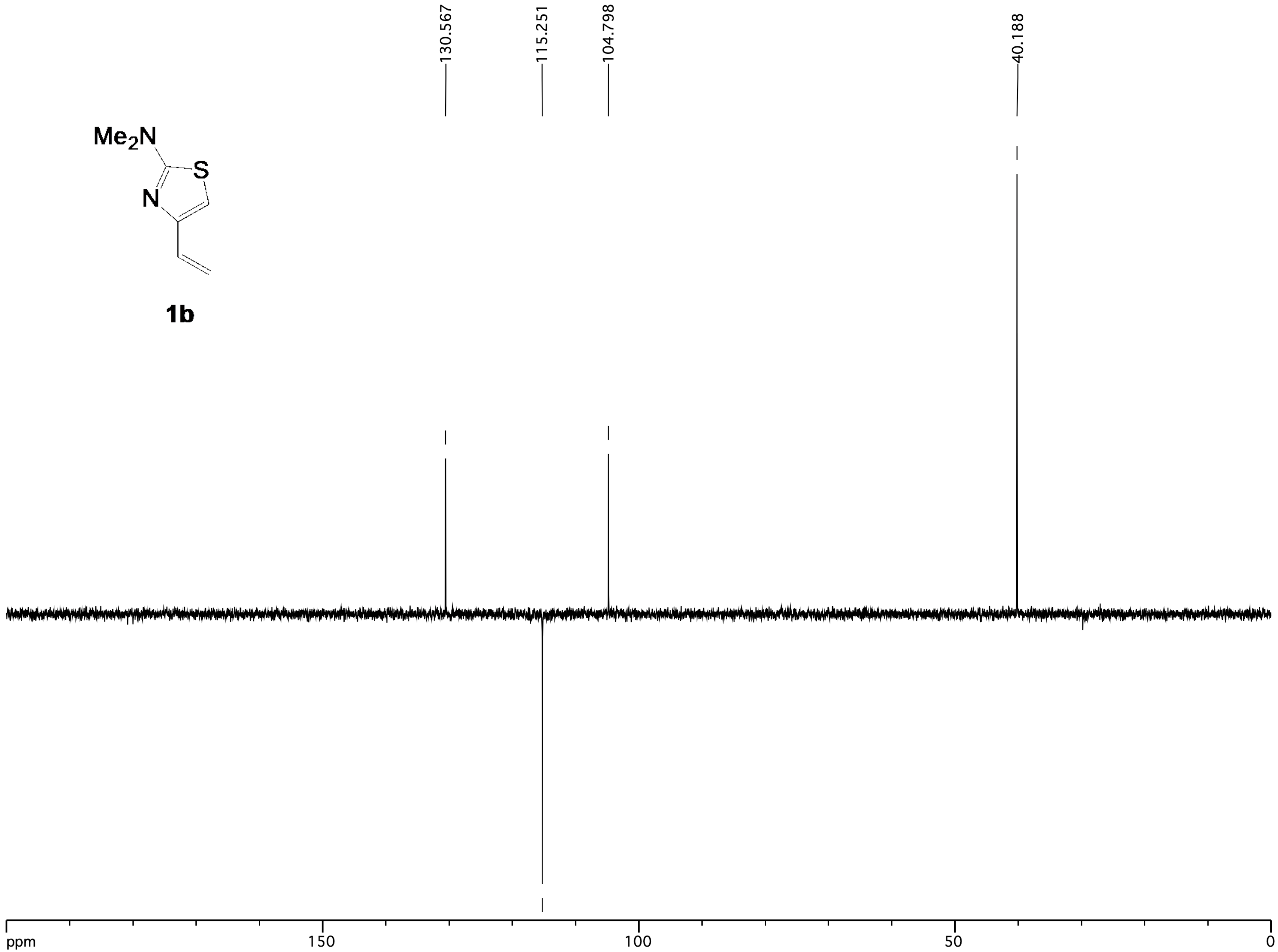

DEPT-135 ($CDCl_3$, 100 MHz) of compound **1b**.

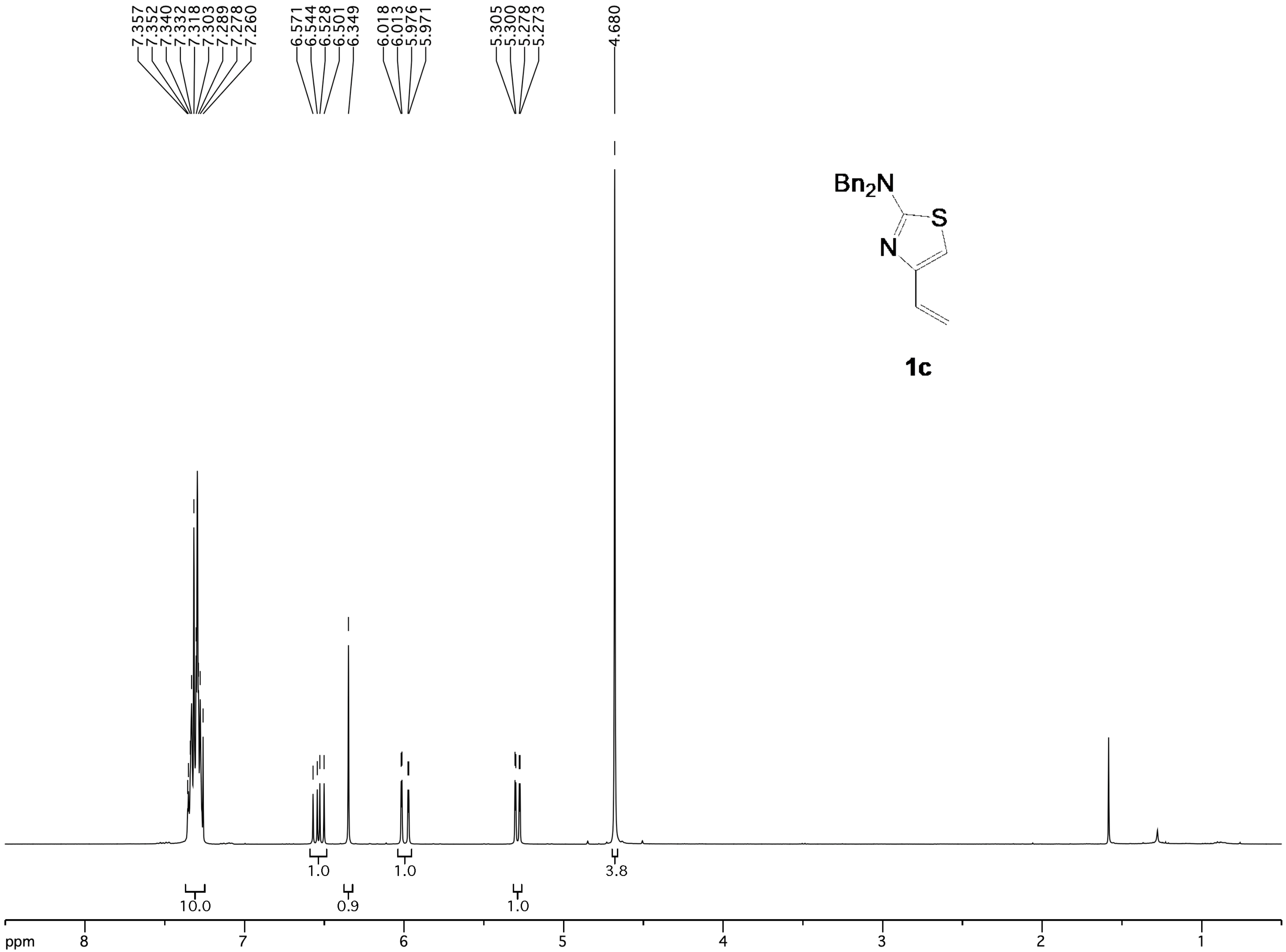

1c

^{1}H NMR ($CDCl_3$, 400 MHz) of compound **1c**.

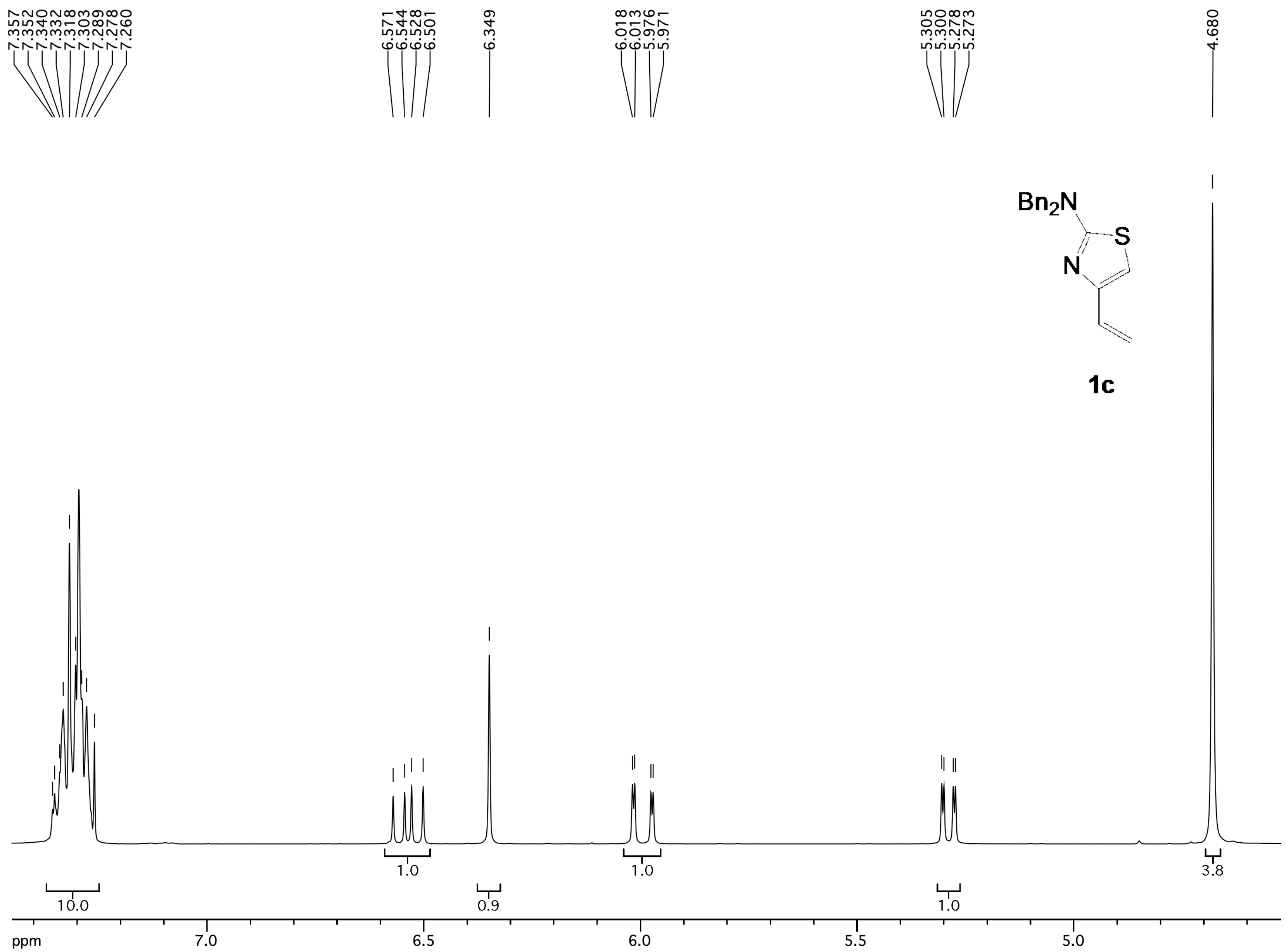

^{1}H NMR (CDCl$_3$, 400 MHz) of compound **1c** (expansion).

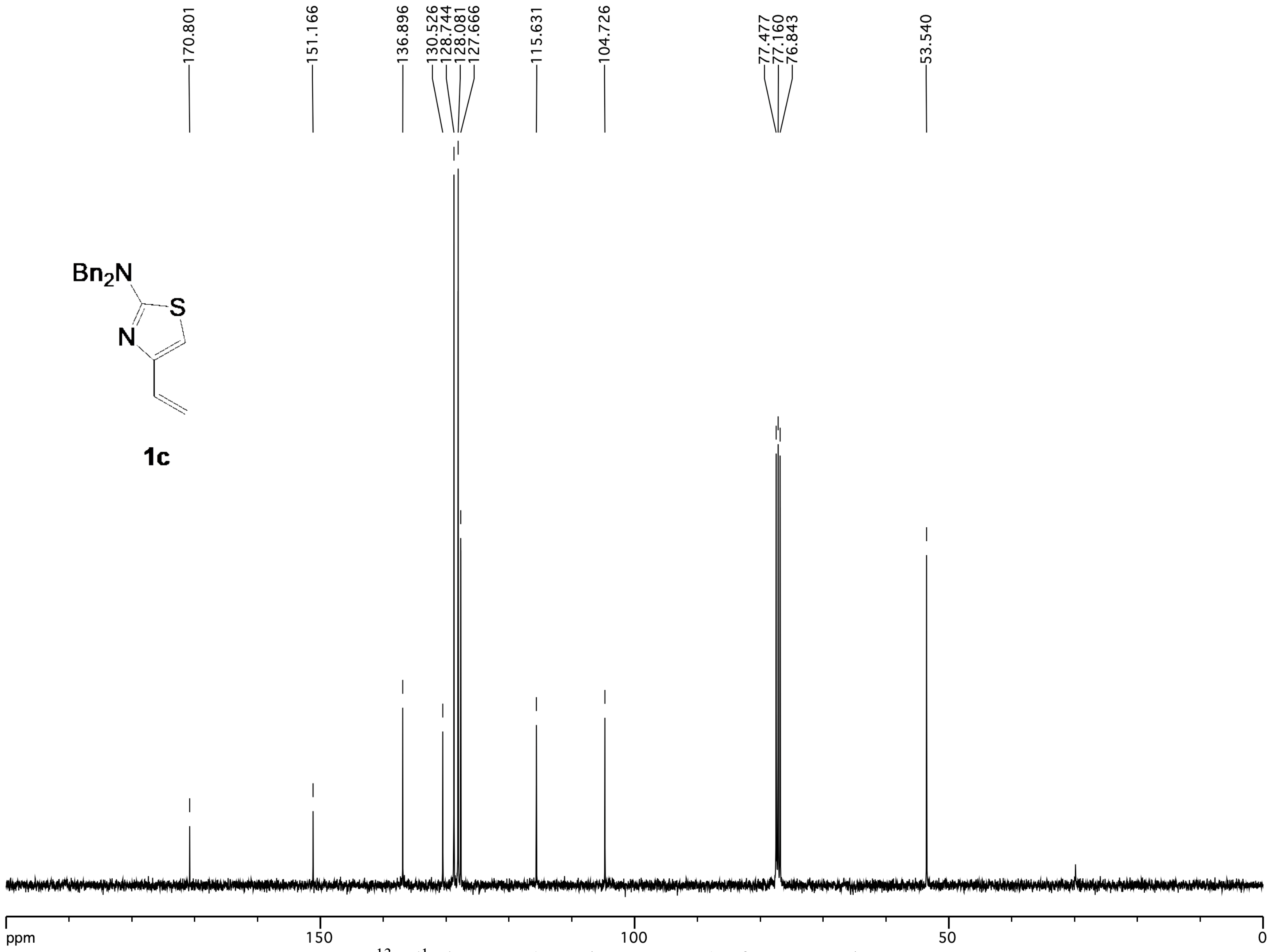

1c

$^{13}C\{^{1}H\}$ NMR ($CDCl_3$, 100 MHz) of compound **1c**.

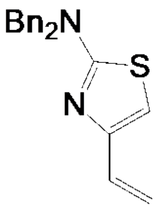

1c

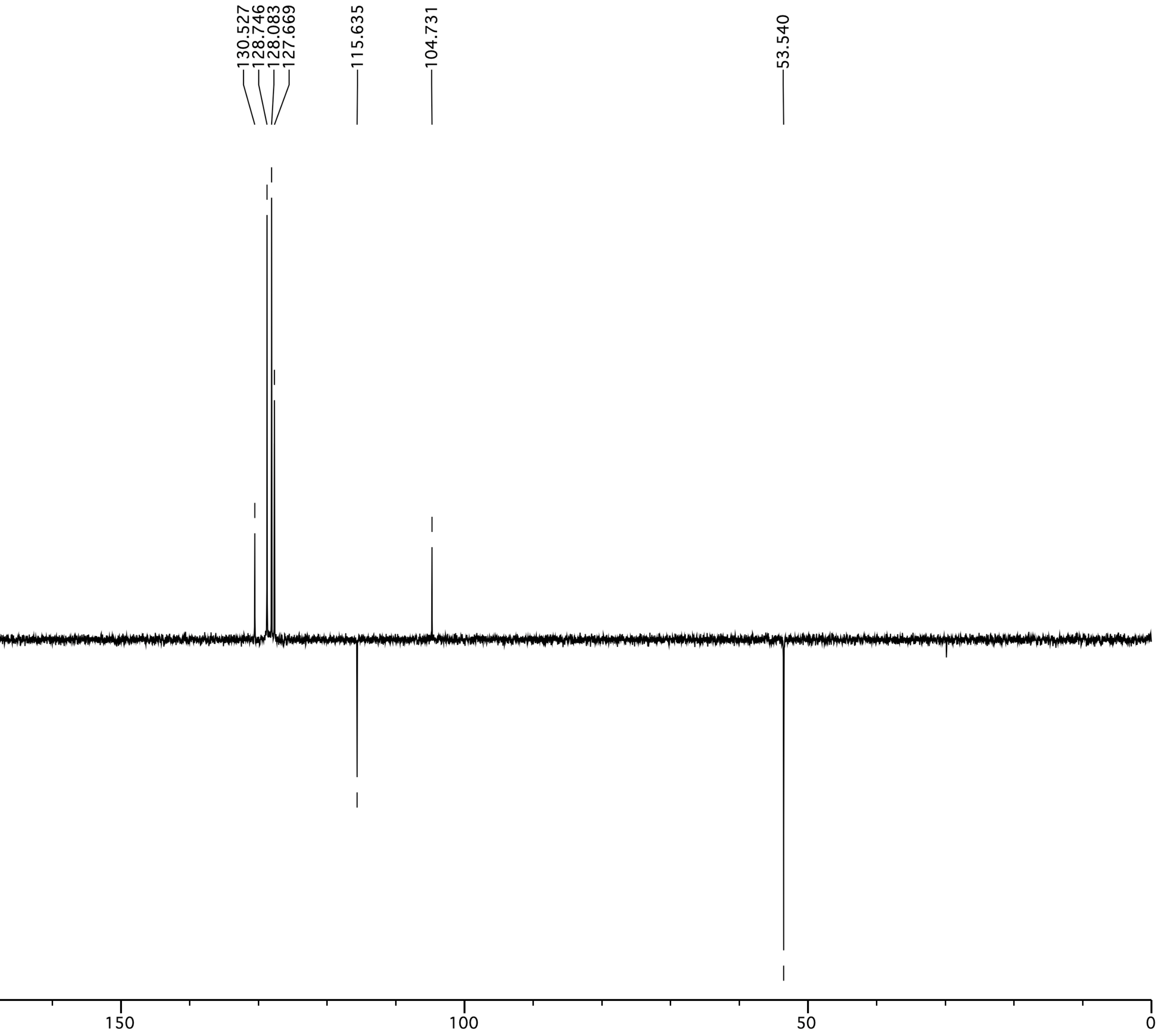

DEPT-135 ($CDCl_3$, 100 MHz) of compound **1c**.

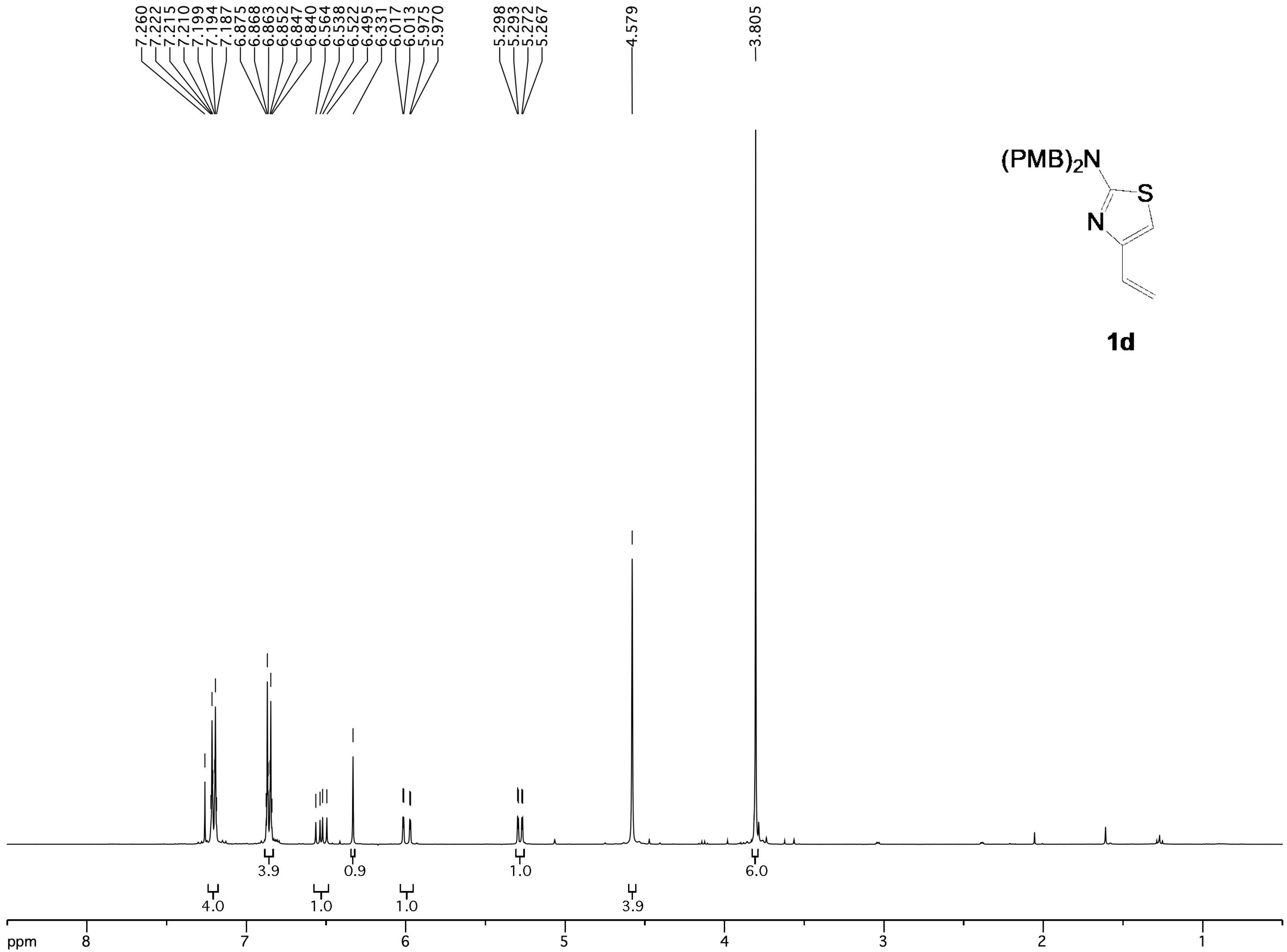

^{1}H NMR (CDCl$_3$, 400 MHz) of compound **1d**.

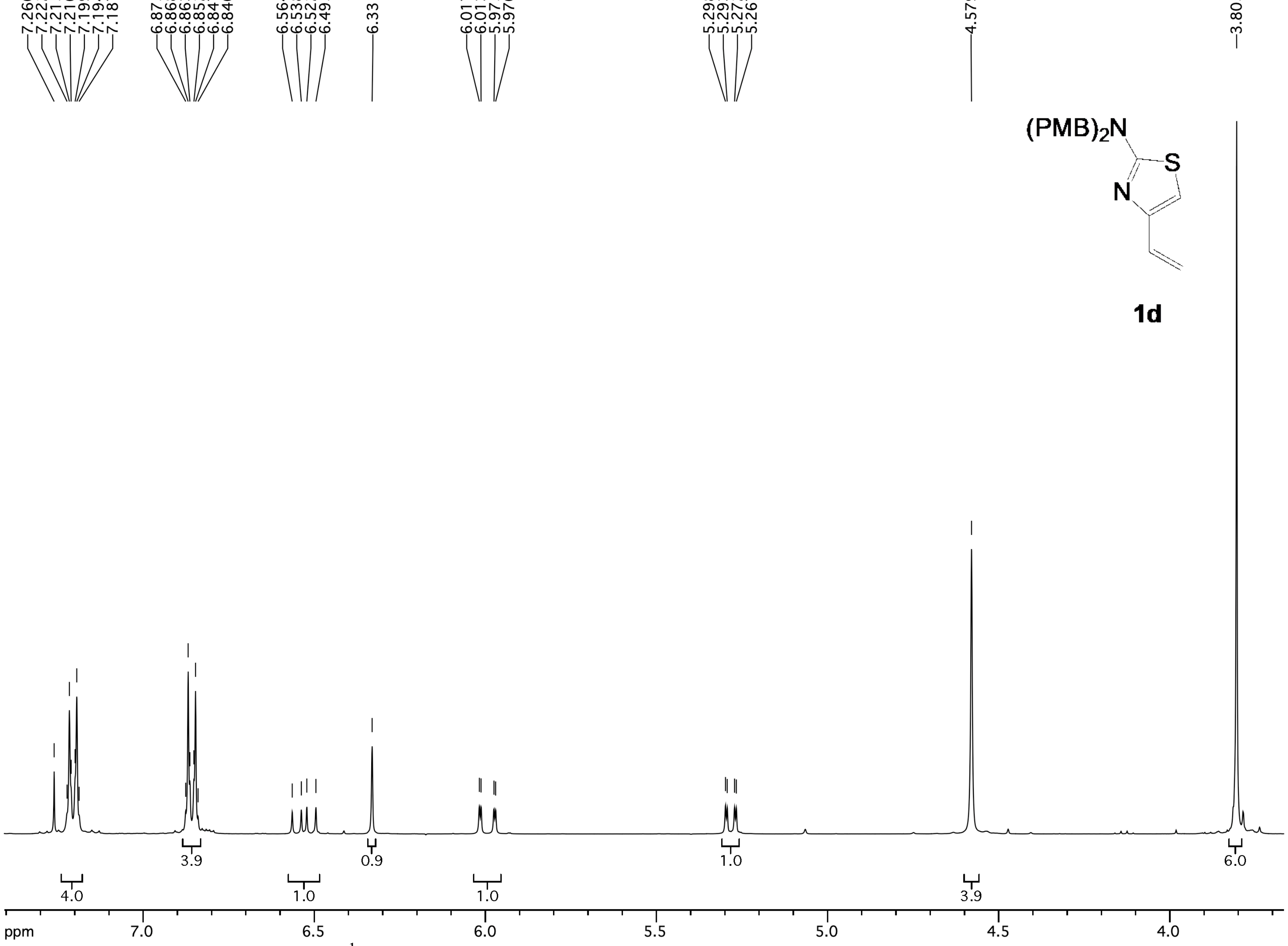

^{1}H NMR ($CDCl_3$, 400 MHz) of compound **1d** (expansion).

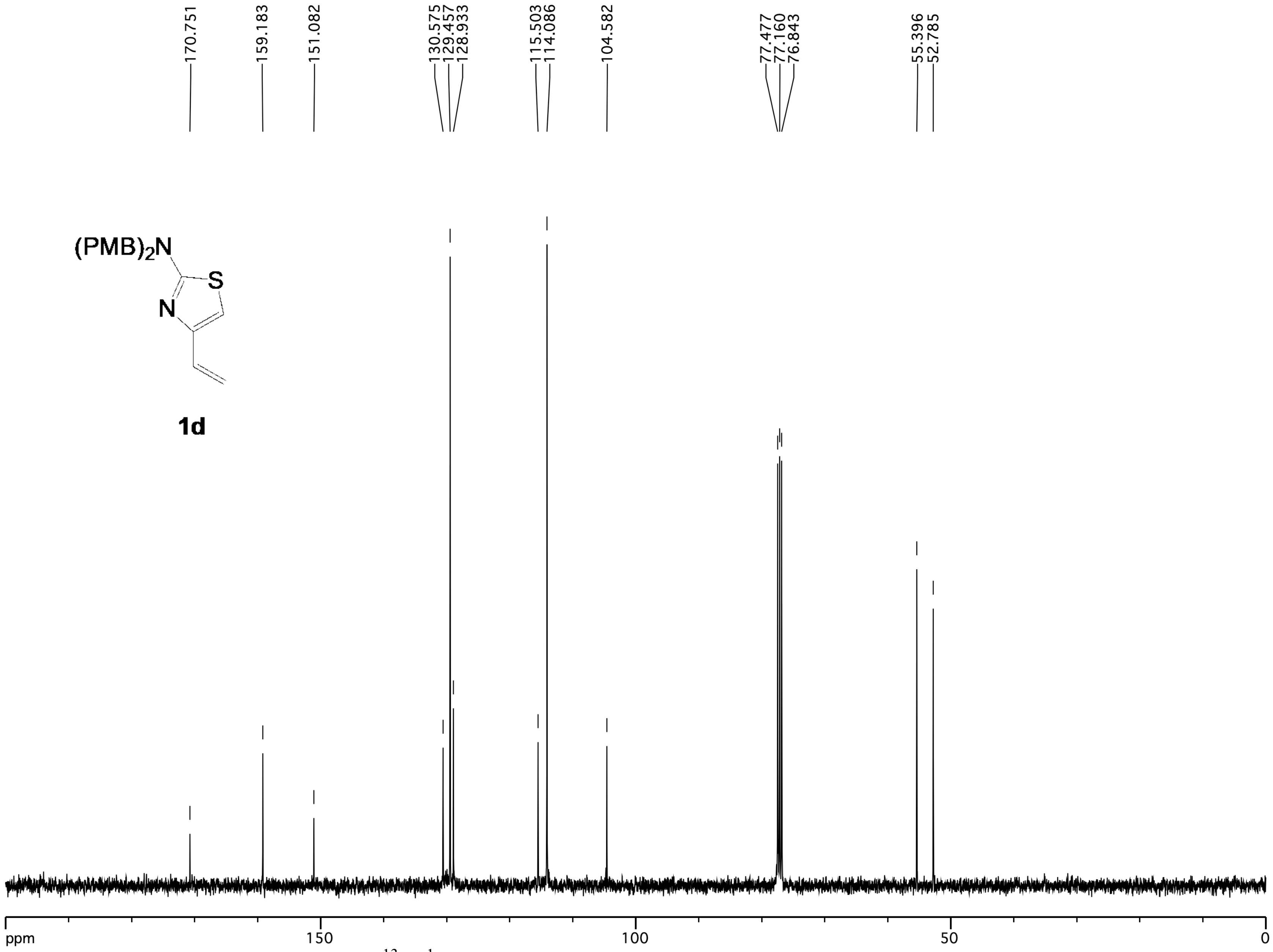

$^{13}C\{^{1}H\}$ NMR ($CDCl_3$, 100 MHz) of compound **1d**.

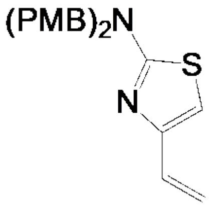

1d

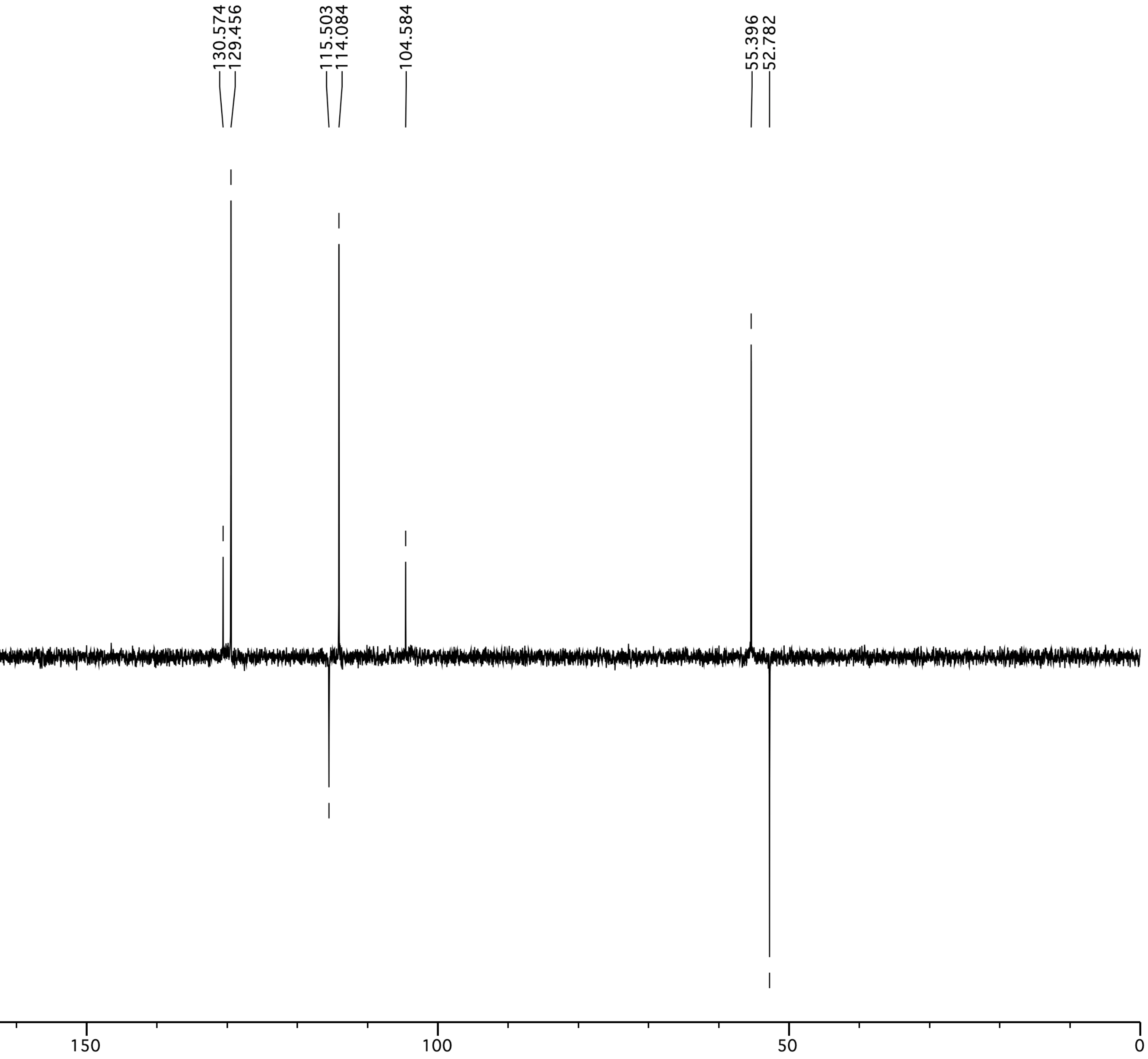

DEPT-135 ($CDCl_3$, 100 MHz) of compound **1d**.

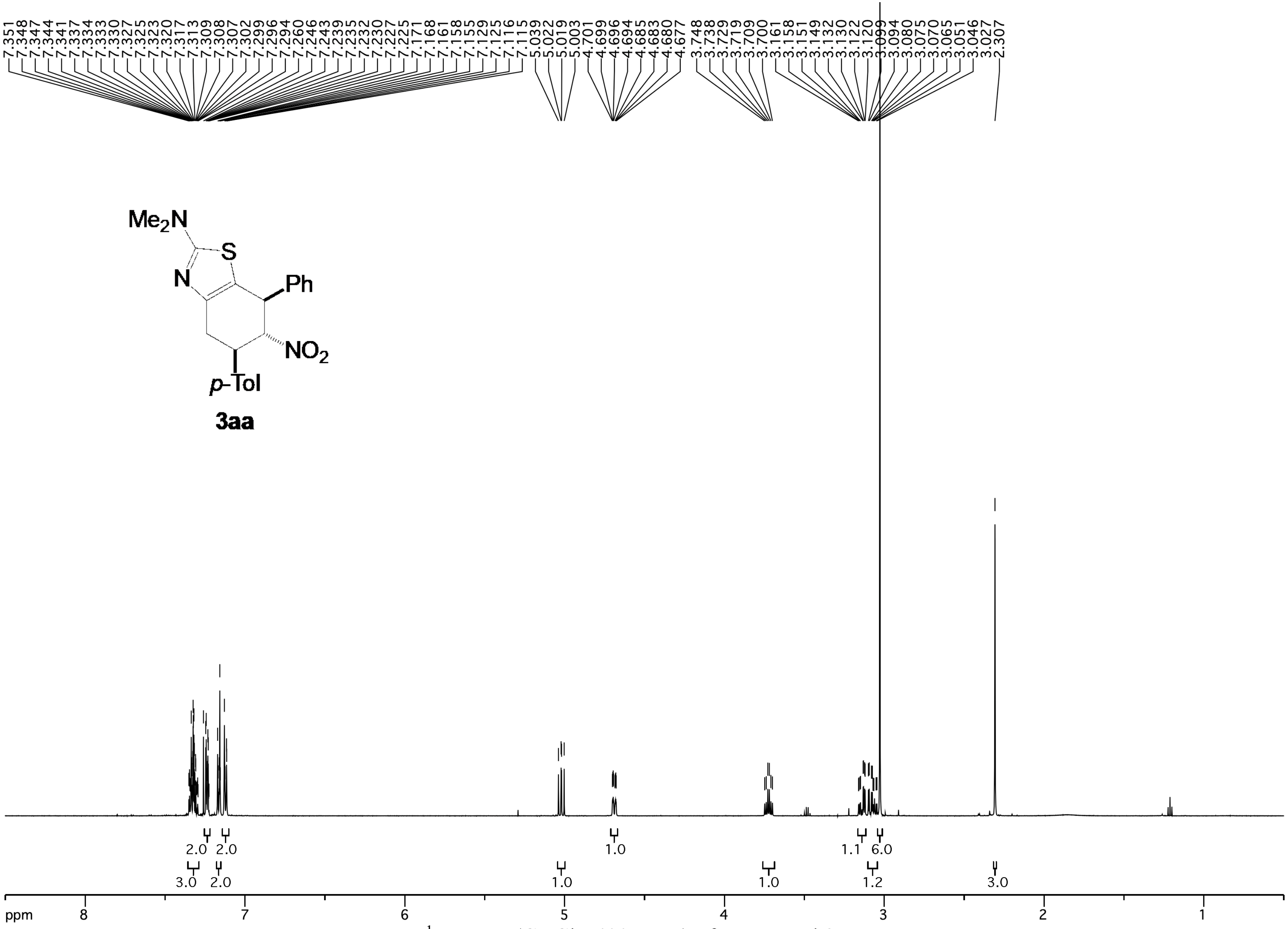

^{1}H NMR ($CDCl_3$, 400 MHz) of compound **3aa**.

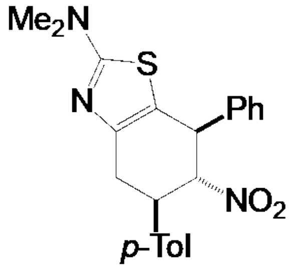

3aa

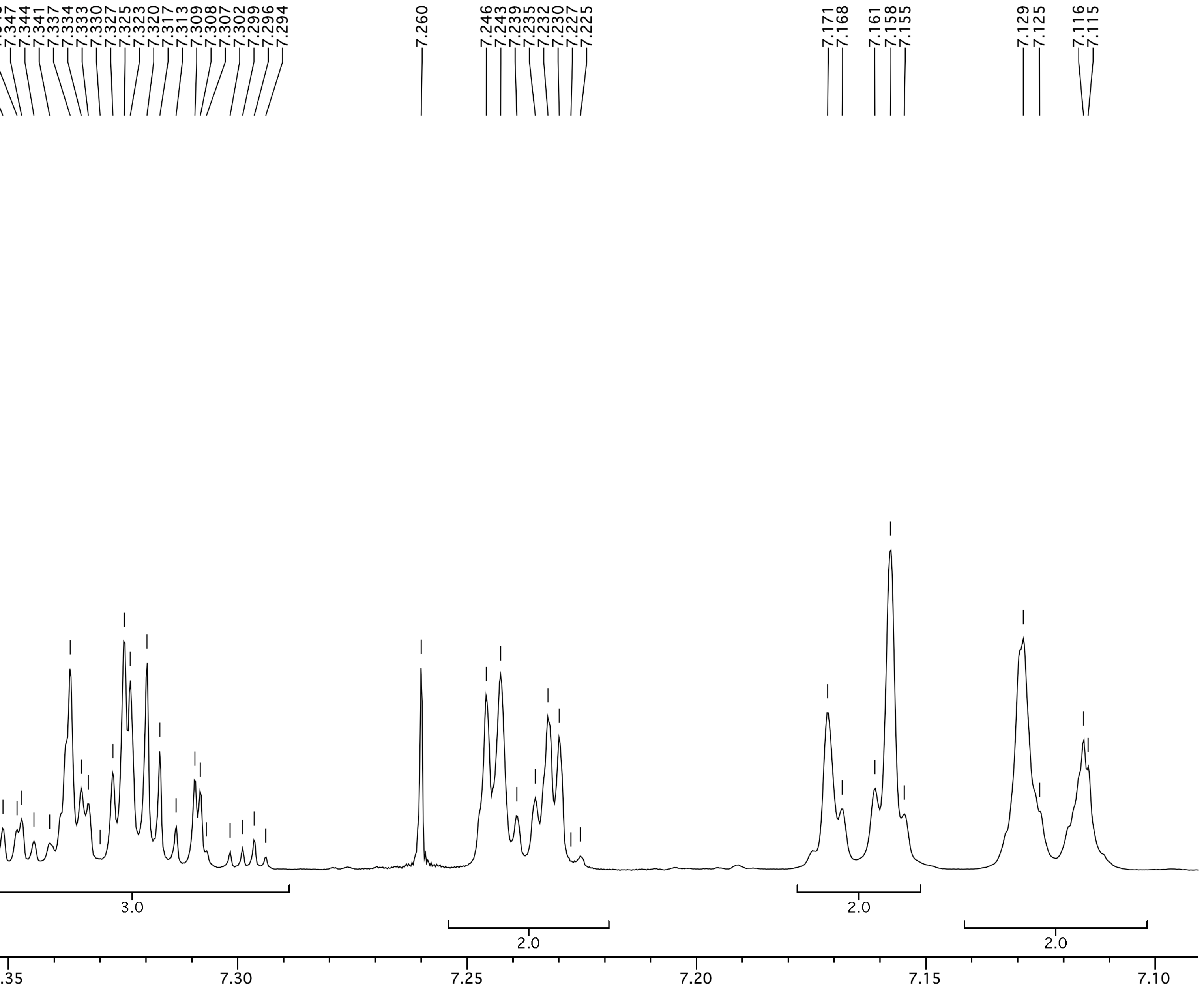

^{1}H NMR (CDCl$_3$, 400 MHz) of compound **3aa** (expansion).

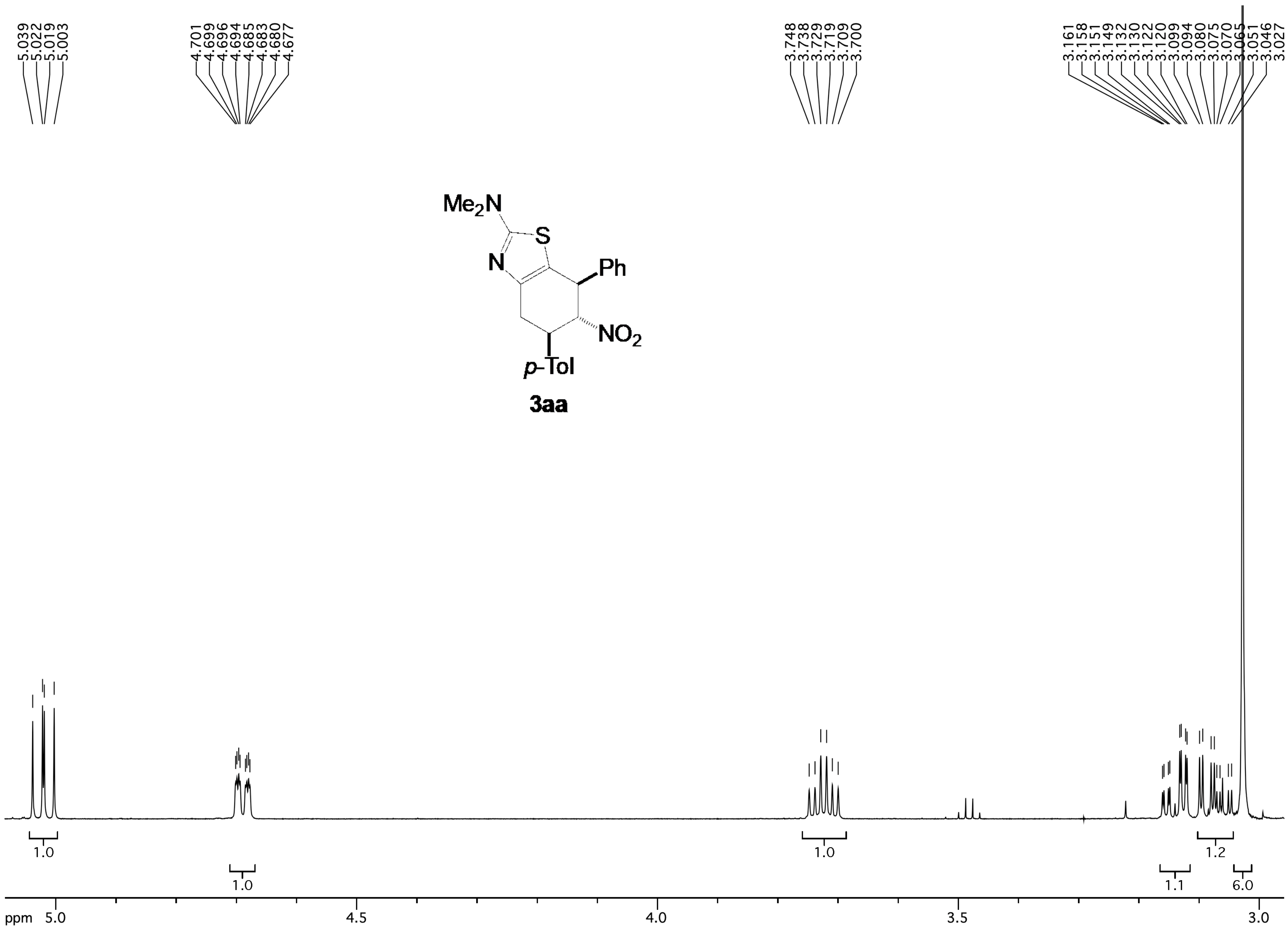

^{1}H NMR ($CDCl_3$, 400 MHz) of compound **3aa** (expansion).

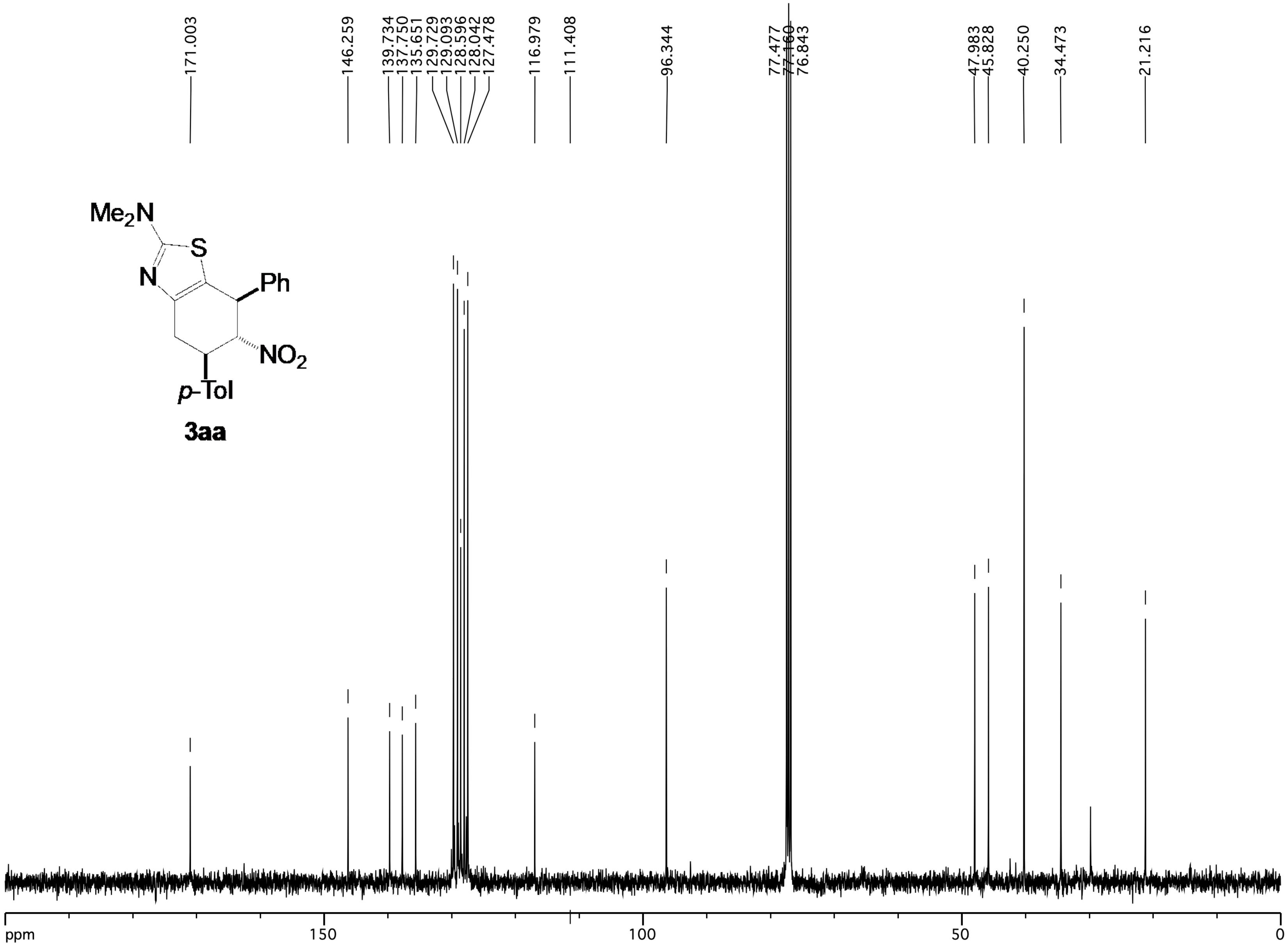

$^{13}C\{^{1}H\}$ NMR (CDCl3, 100 MHz) of compound **3aa**.

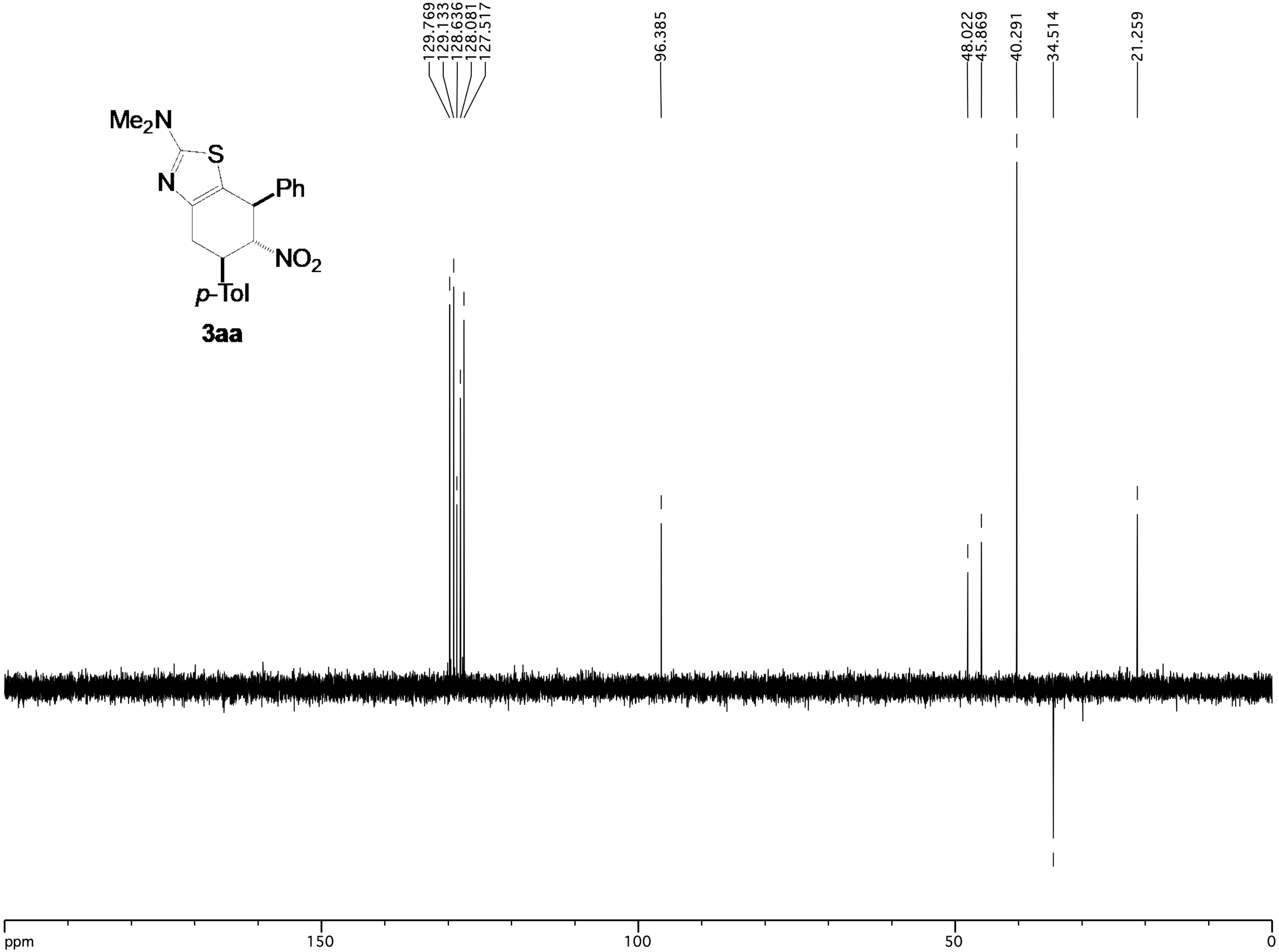

DEPT-135 ($CDCl_3$, 100 MHz) of compound **3aa**.

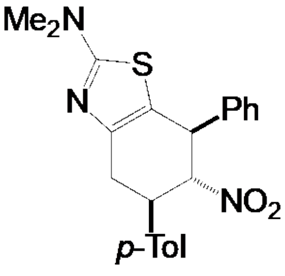

3aa

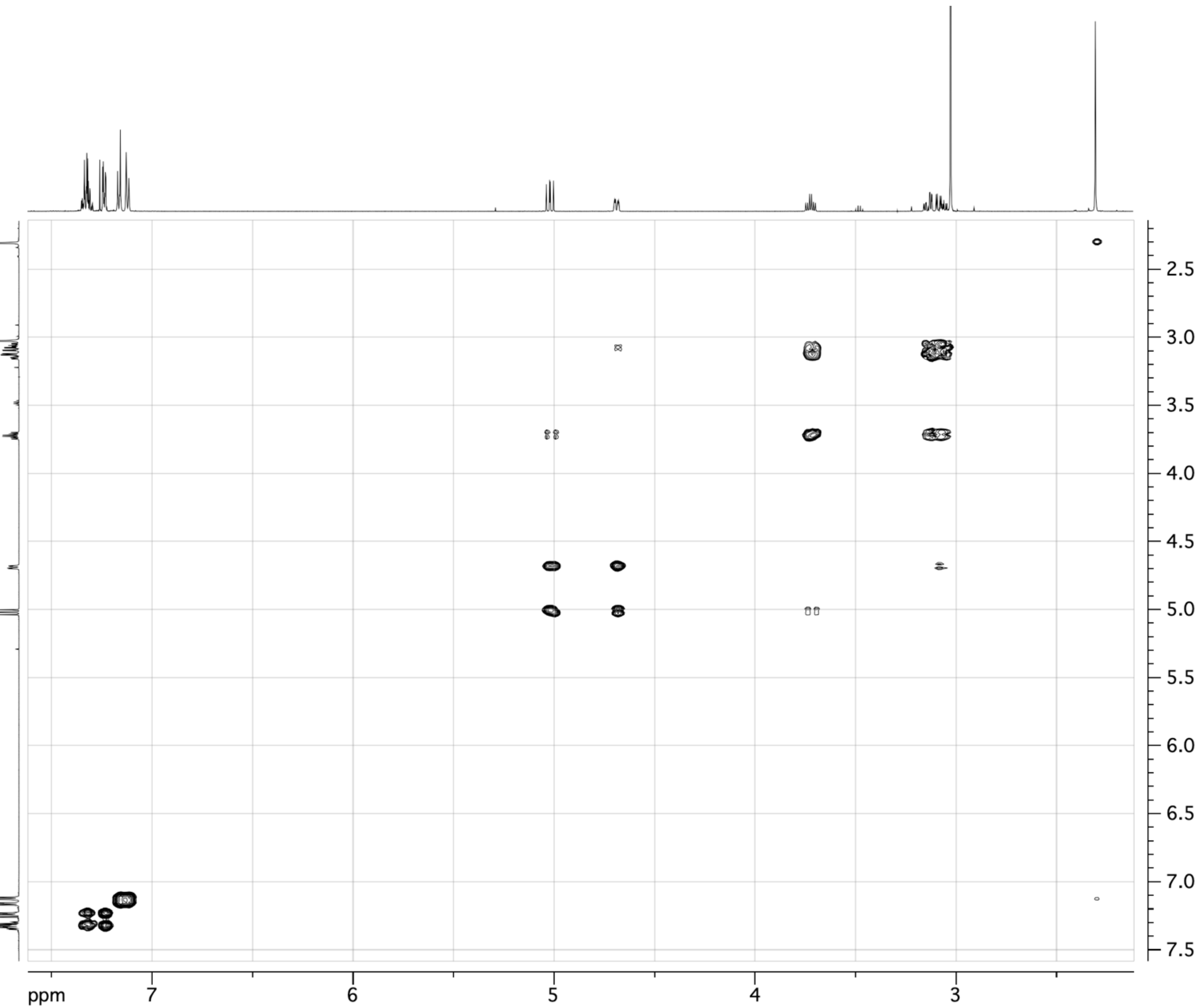

COSY ($CDCl_3$, 600 MHz) of compound **3aa**.

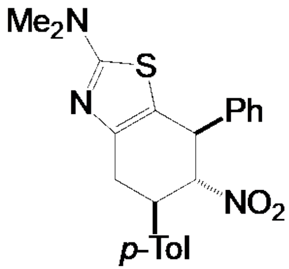

3aa

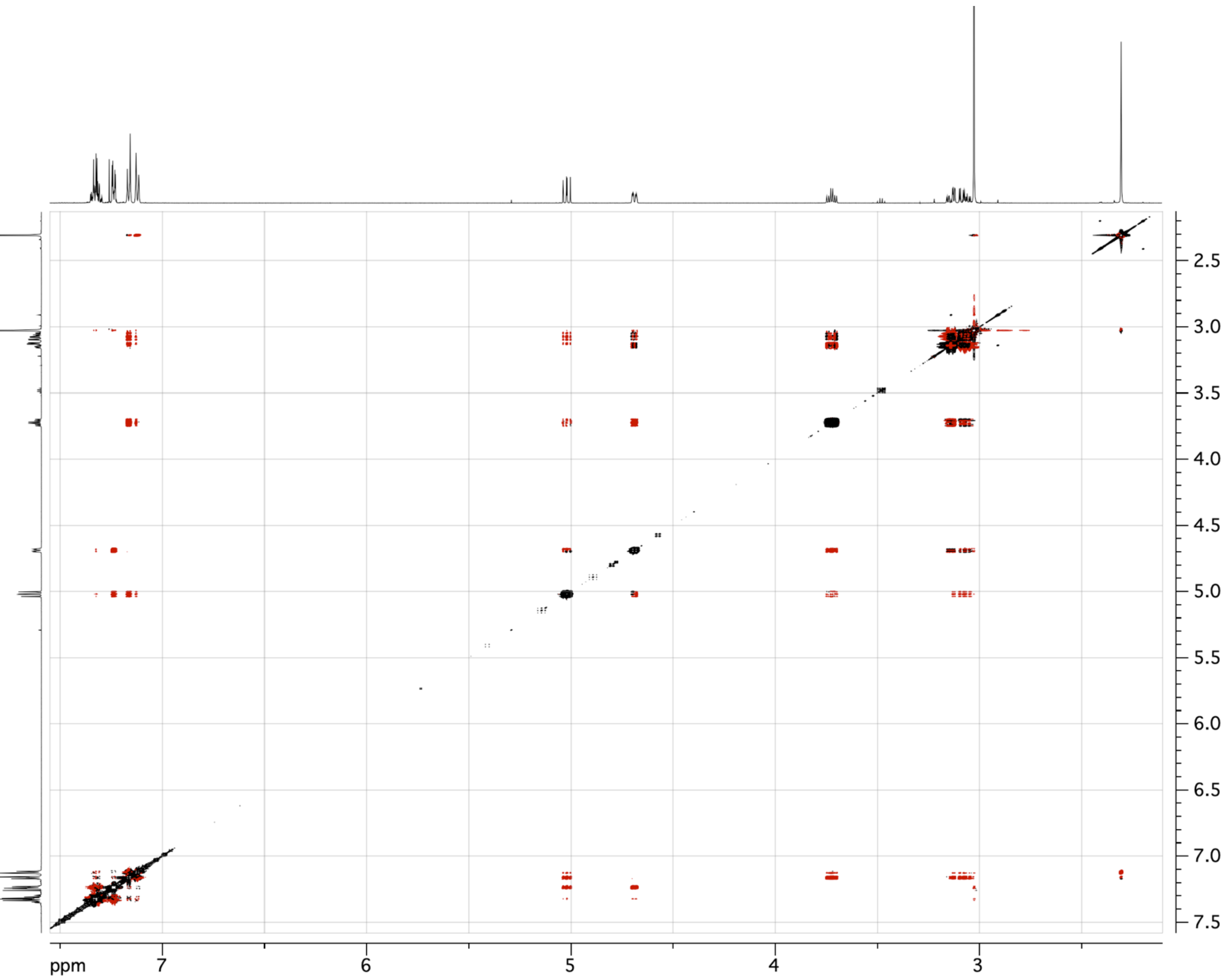

NOESY ($CDCl_3$, 600 MHz) of compound **3aa**.

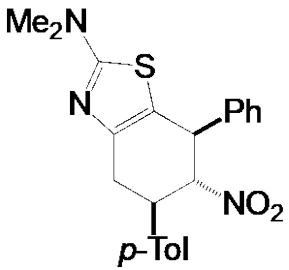

3aa

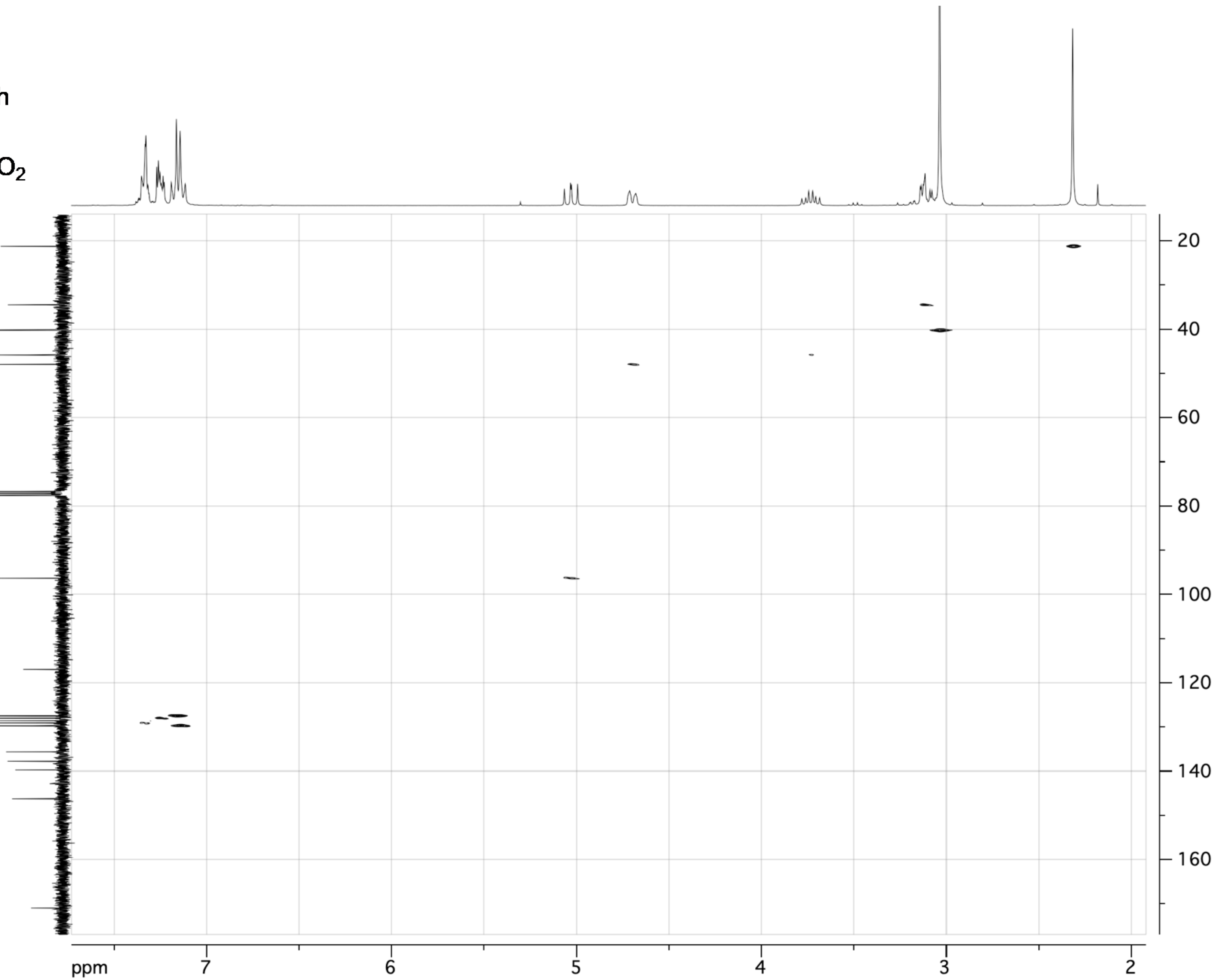

HSQC (CDCl3, 300 MHz) of compound **3aa**.

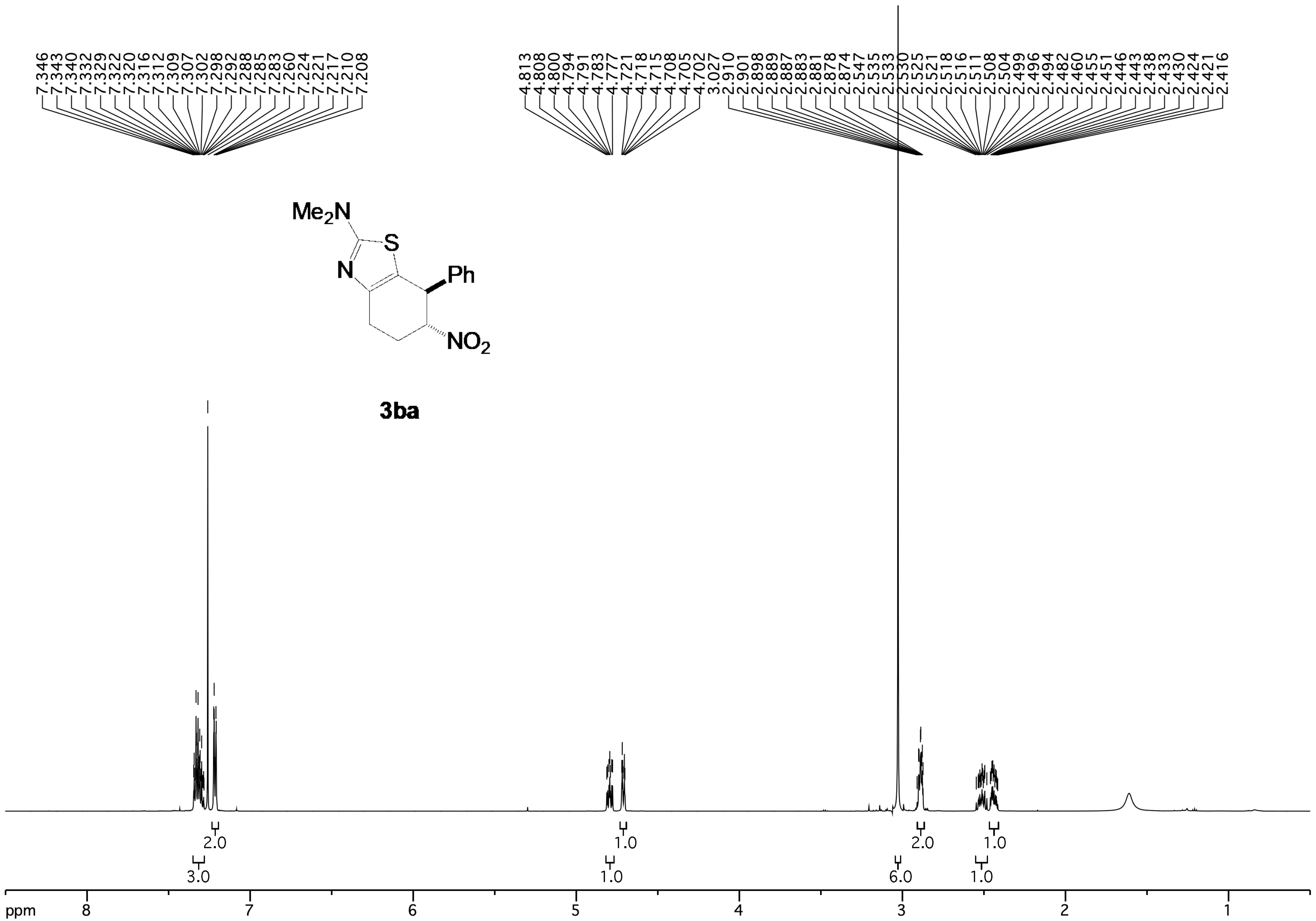

3ba

^{1}H NMR ($CDCl_3$, 600 MHz) of compound **3ba**.

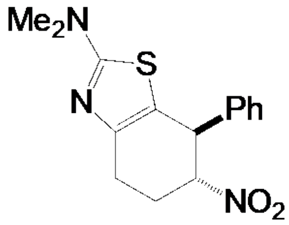

3ba

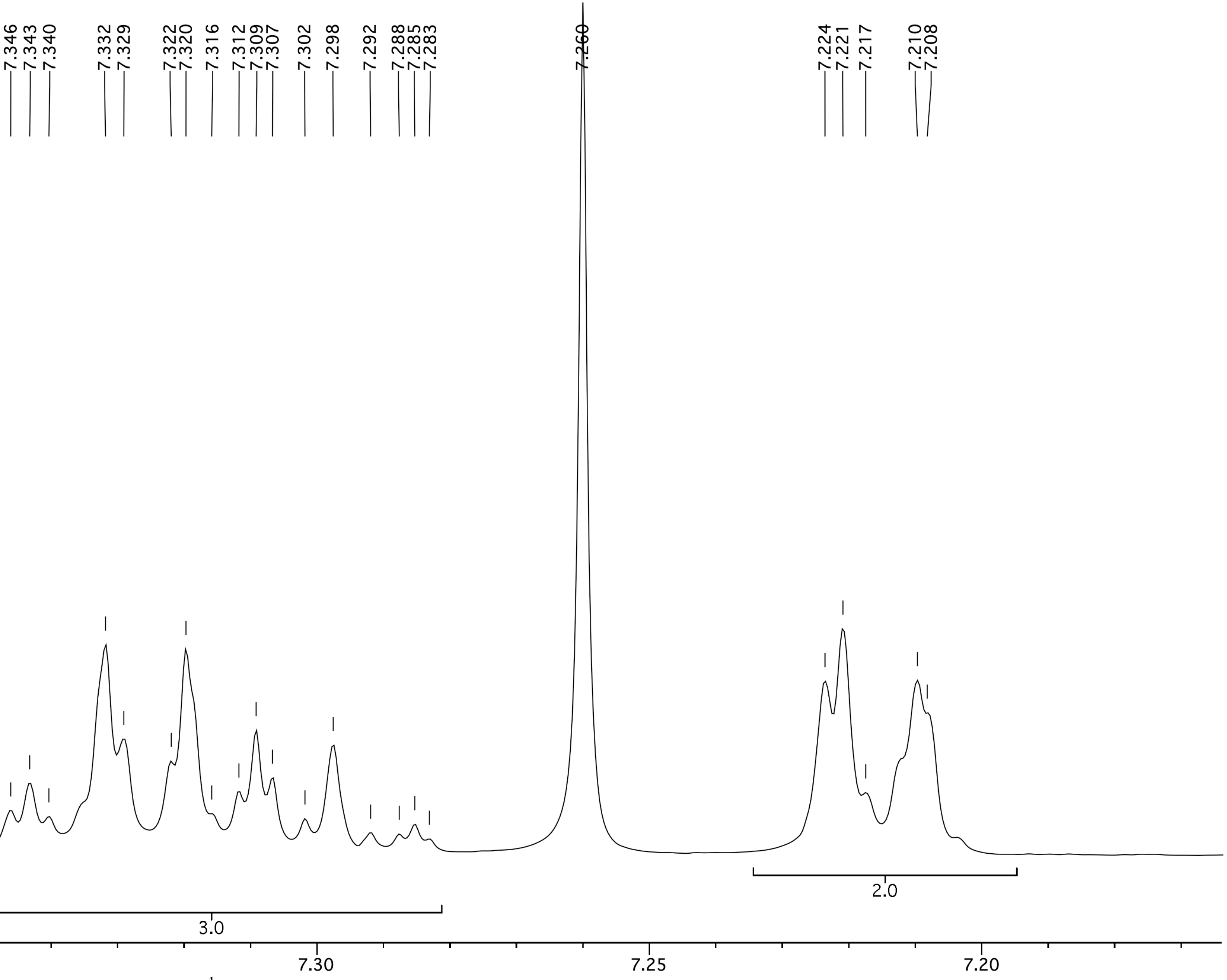

^{1}H NMR (CDCl$_3$, 600 MHz) of compound **3ba** (expansion).

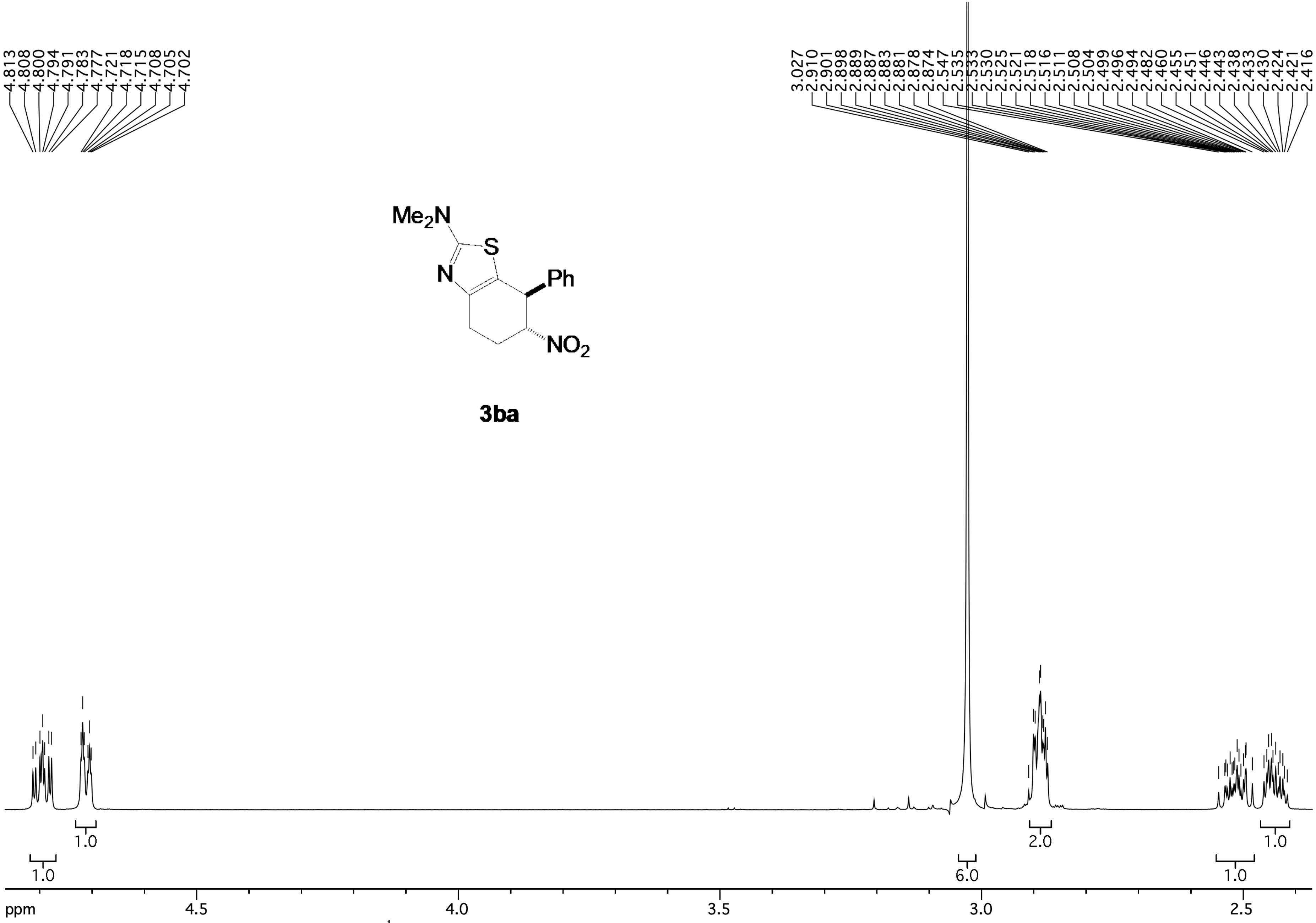

^{1}H NMR ($CDCl_3$, 600 MHz) of compound **3ba** (expansion).

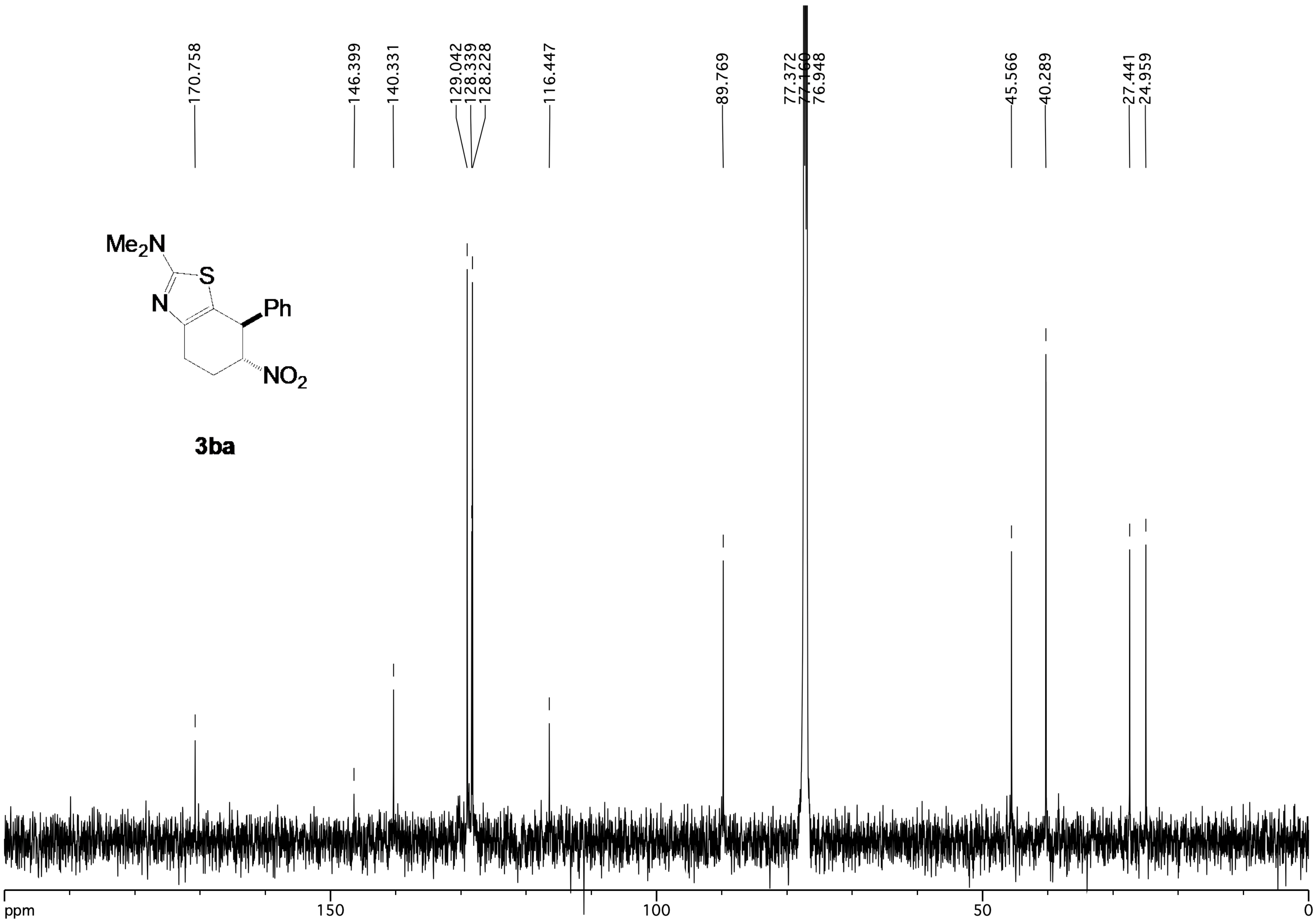

$^{13}C\{^{1}H\}$ NMR ($CDCl_3$, 150 MHz) of compound **3ba**.

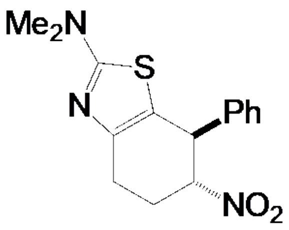

3ba

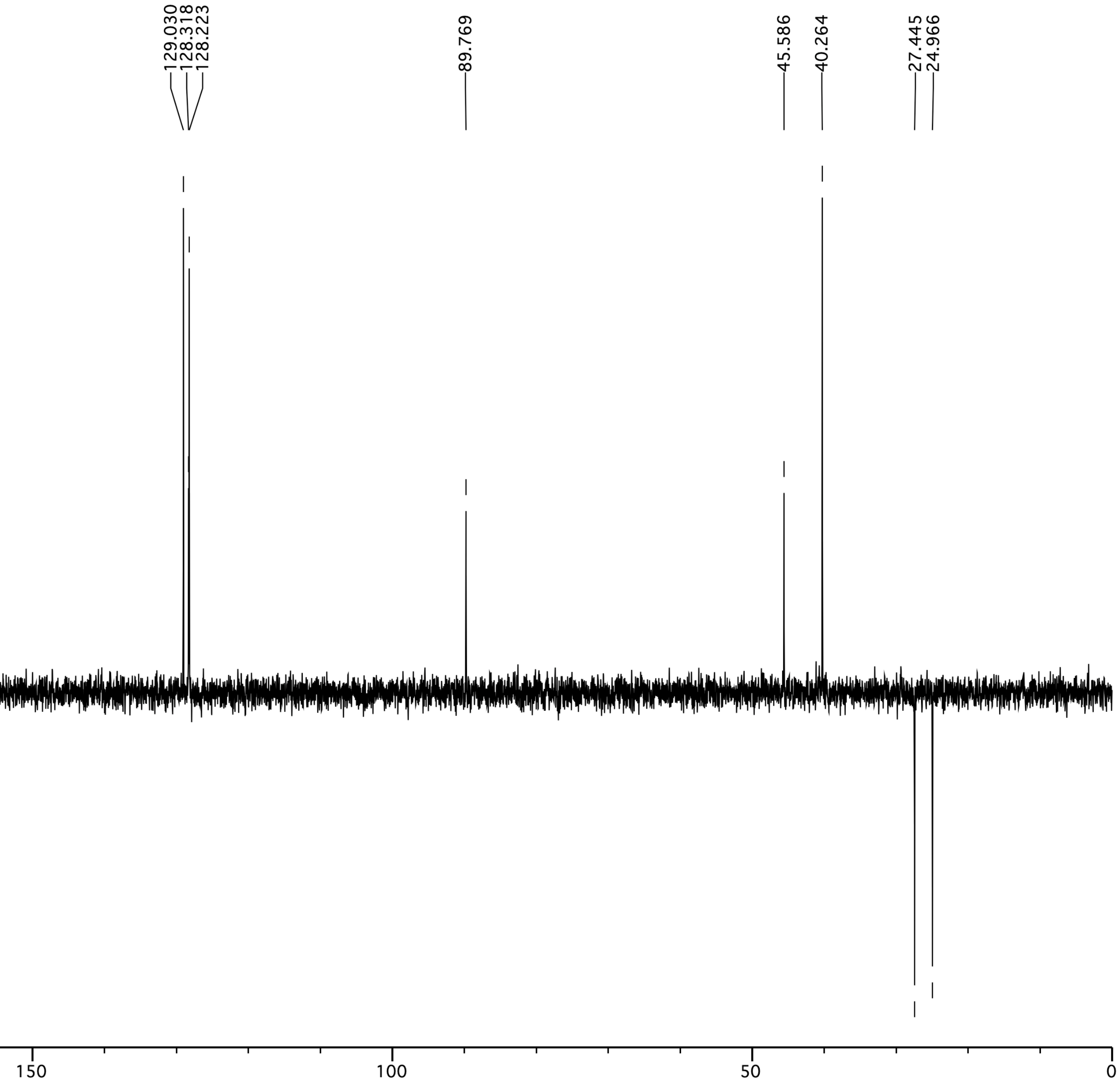

DEPT-135 ($CDCl_3$, 100 MHz) of compound **3ba**.

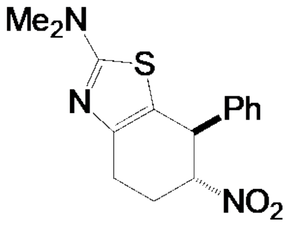

3ba

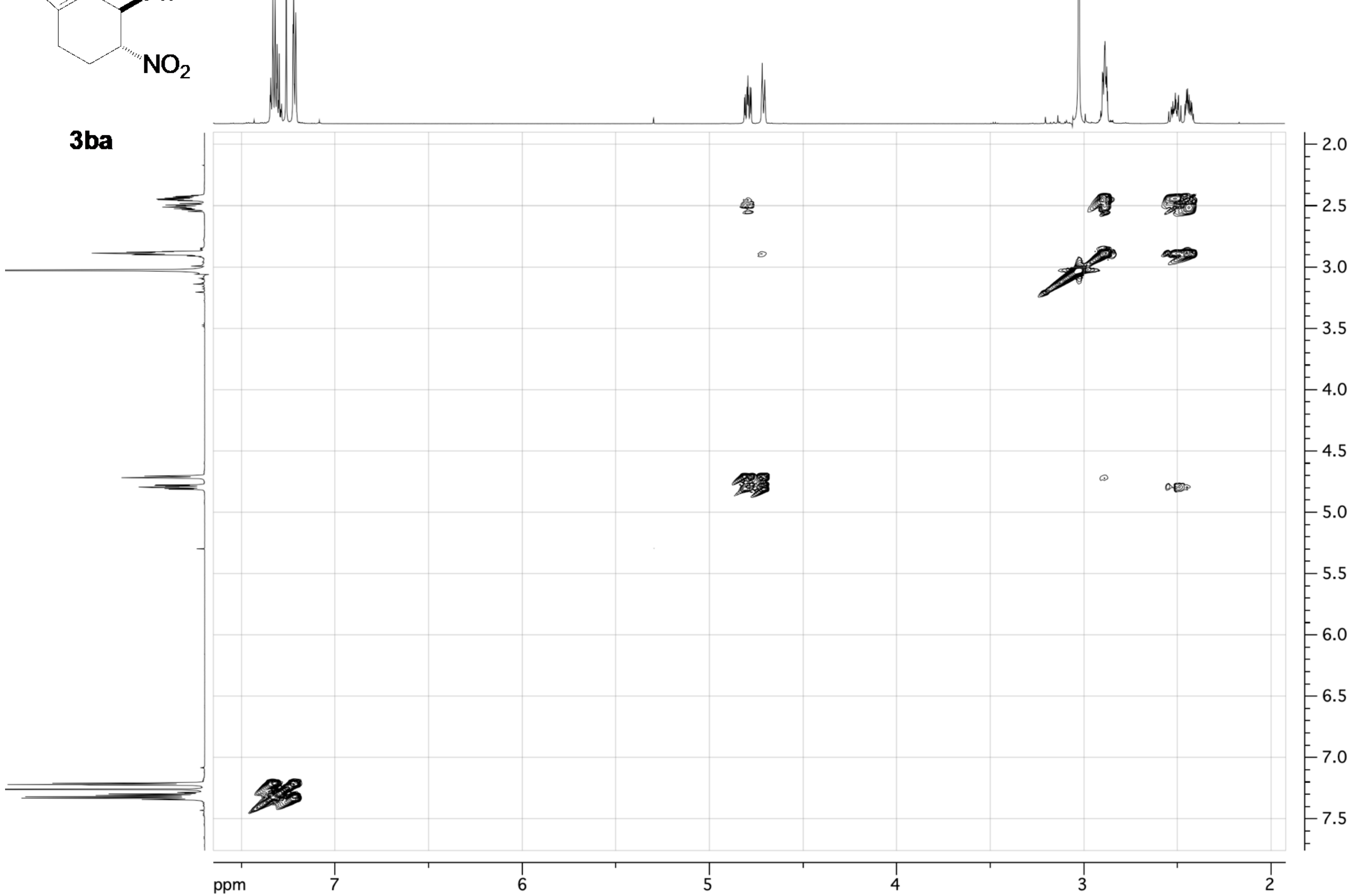

COSY ($CDCl_3$, 600 MHz) of compound **3ba**.

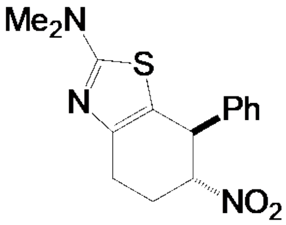

3ba

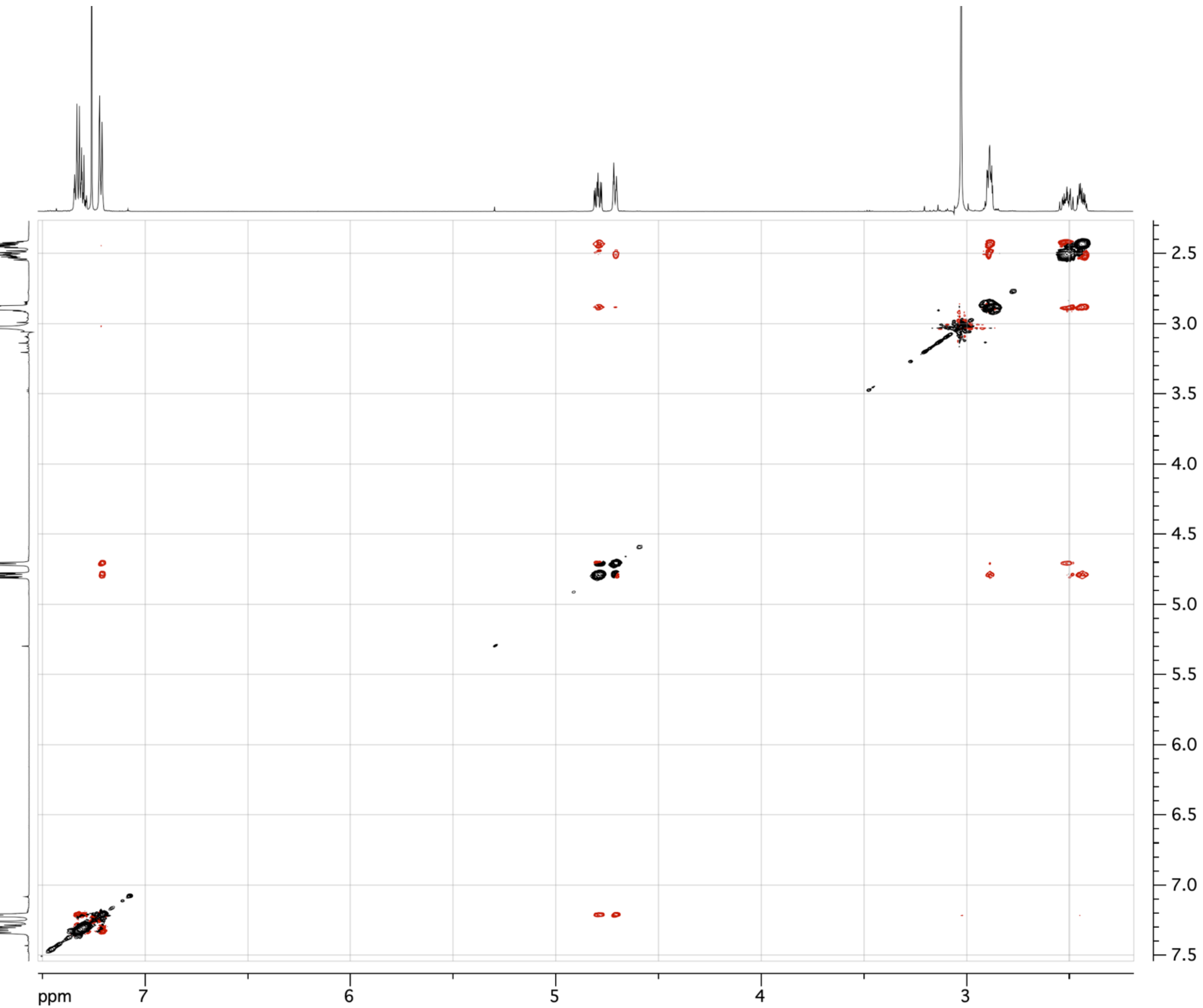

NOESY (CDCl₃, 600 MHz) of compound **3ba**.

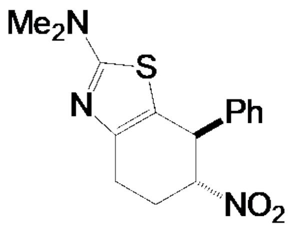

3ba

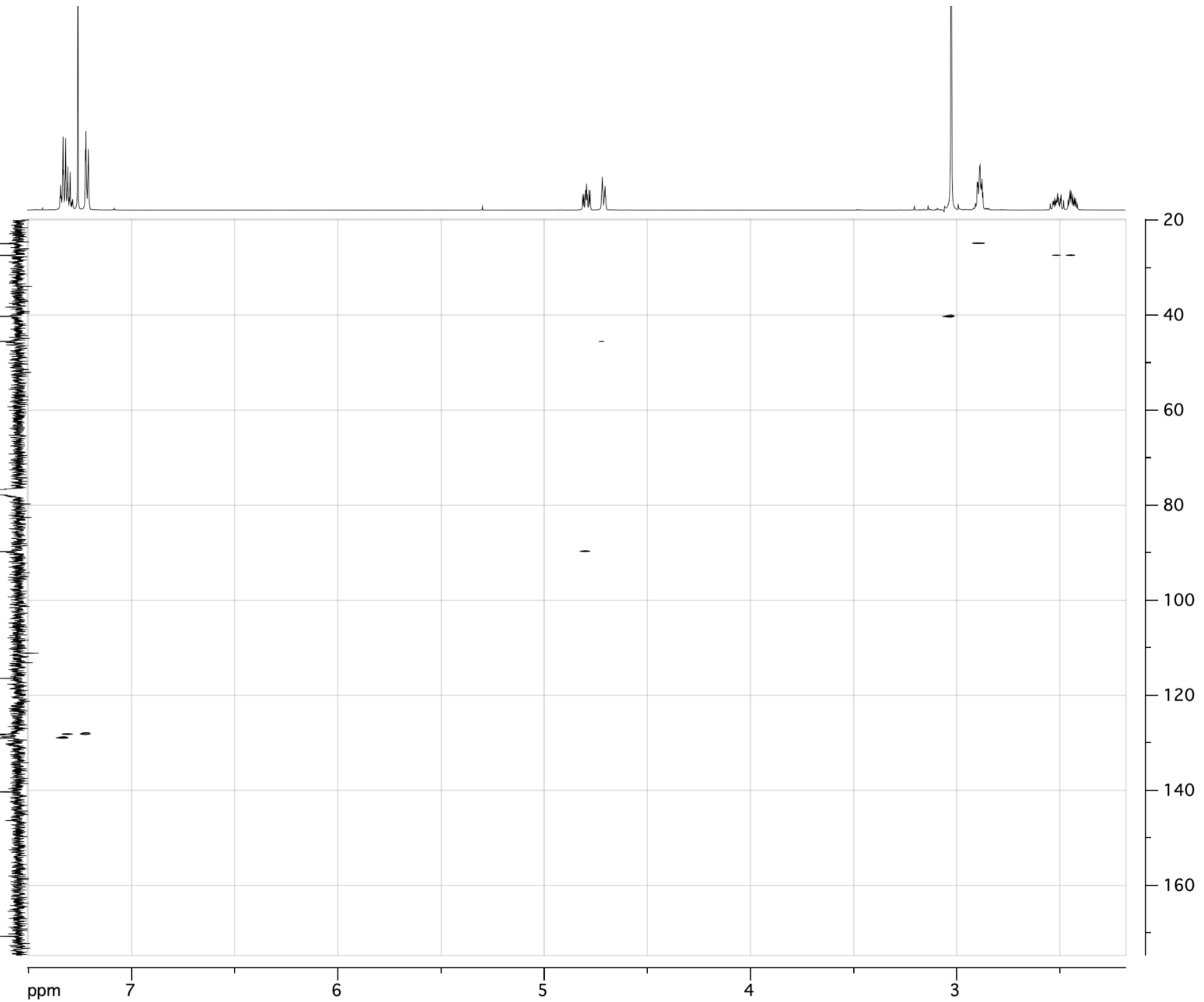

HSQC ($CDCl_3$, 600 MHz) of compound **3ba**.

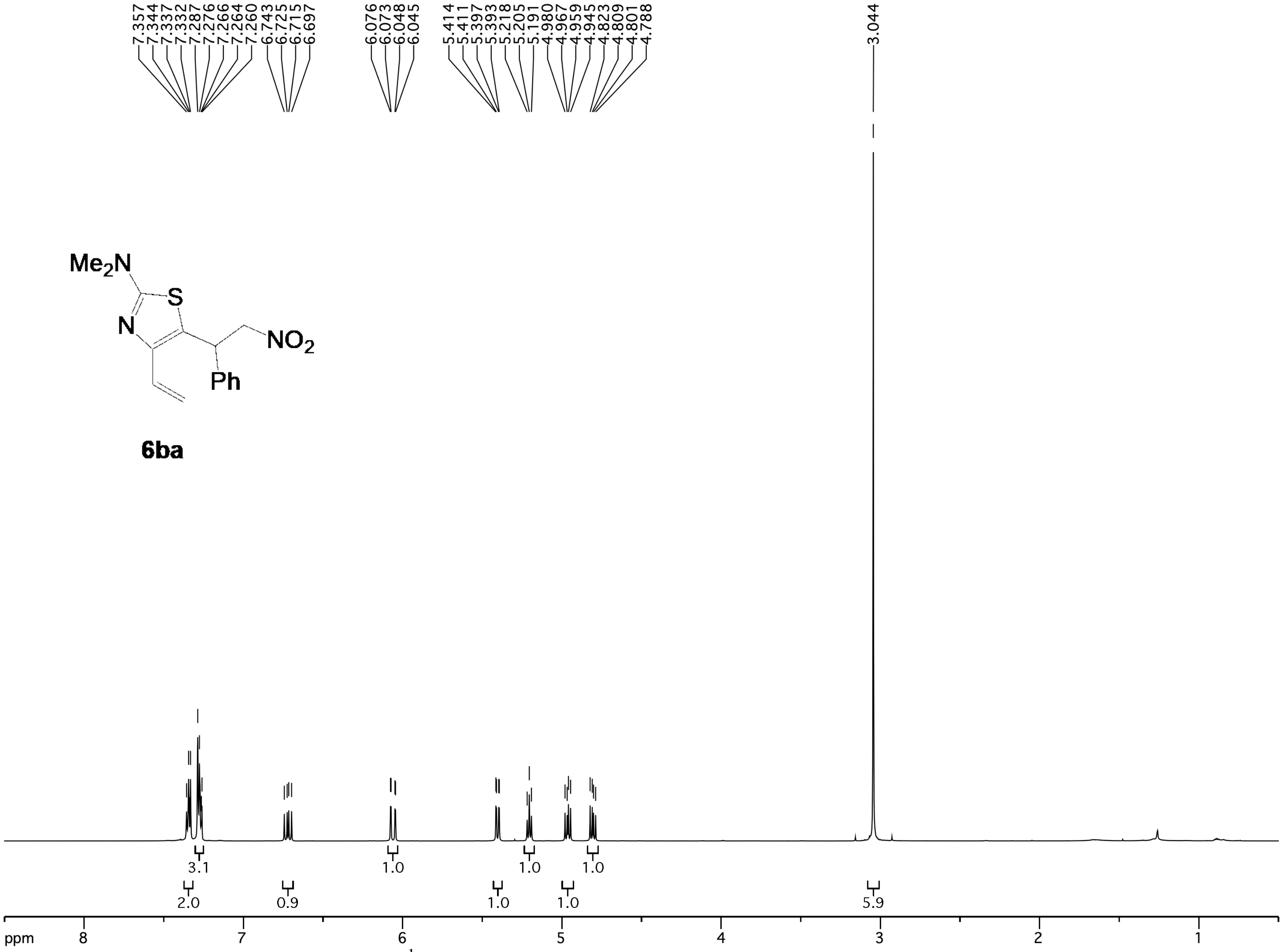

^{1}H NMR ($CDCl_3$, 600 MHz) of compound **6ba**.

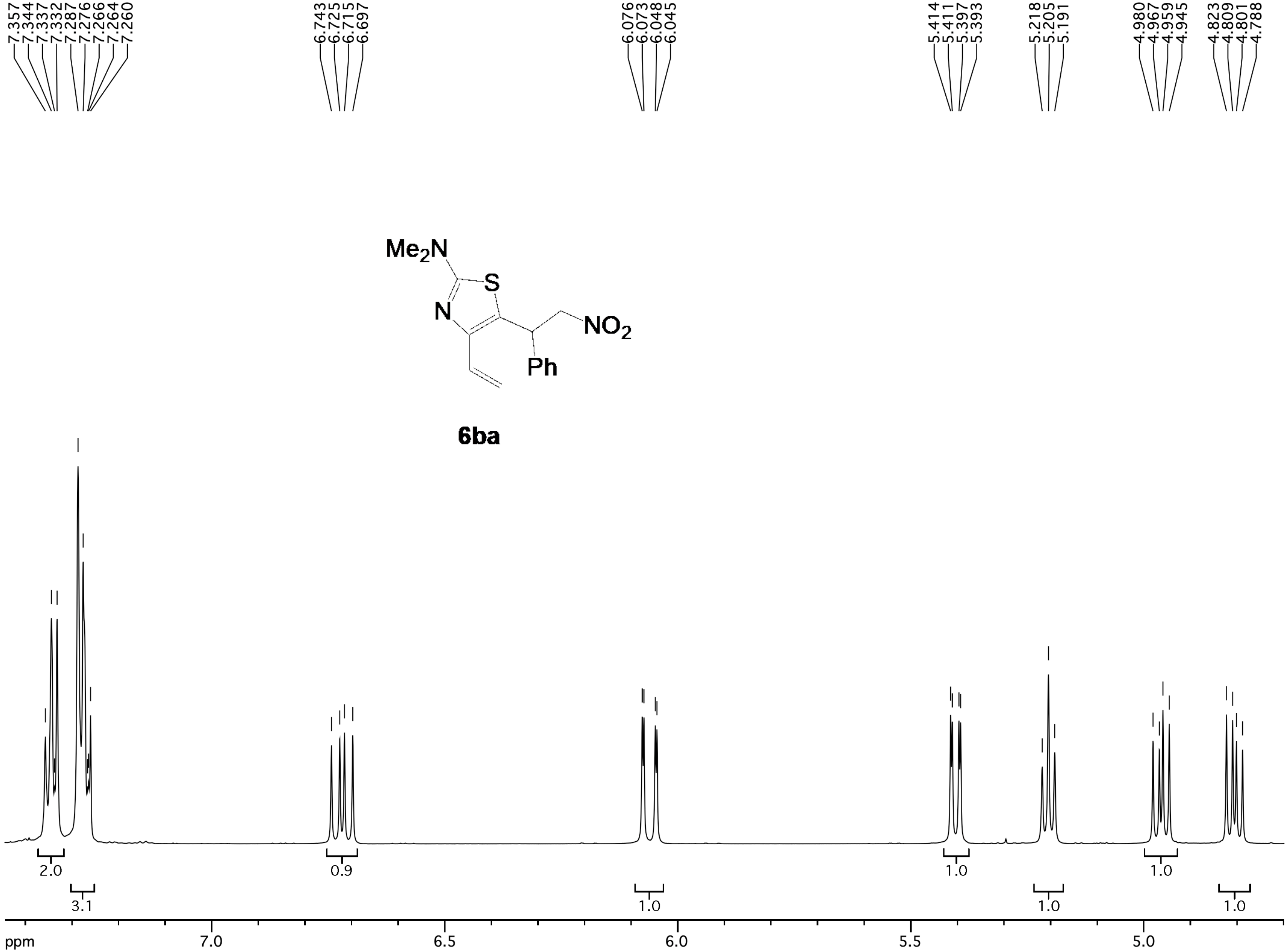

^{1}H NMR (CDCl$_3$, 600 MHz) of compound **6ba** (ampliado).

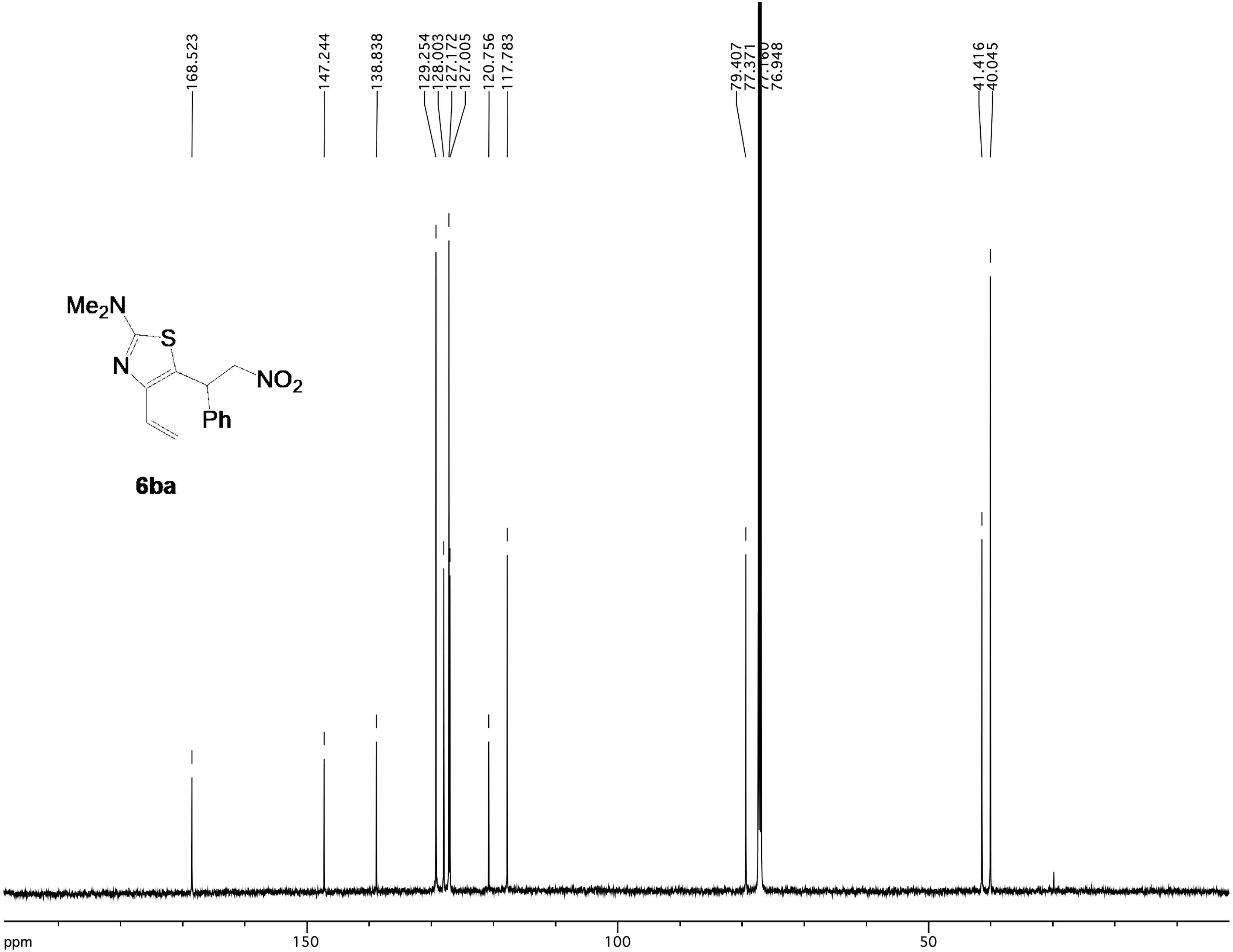

$^{13}C\{^{1}H\}$ NMR ($CDCl_3$, 150 MHz) of compound **6ba**.

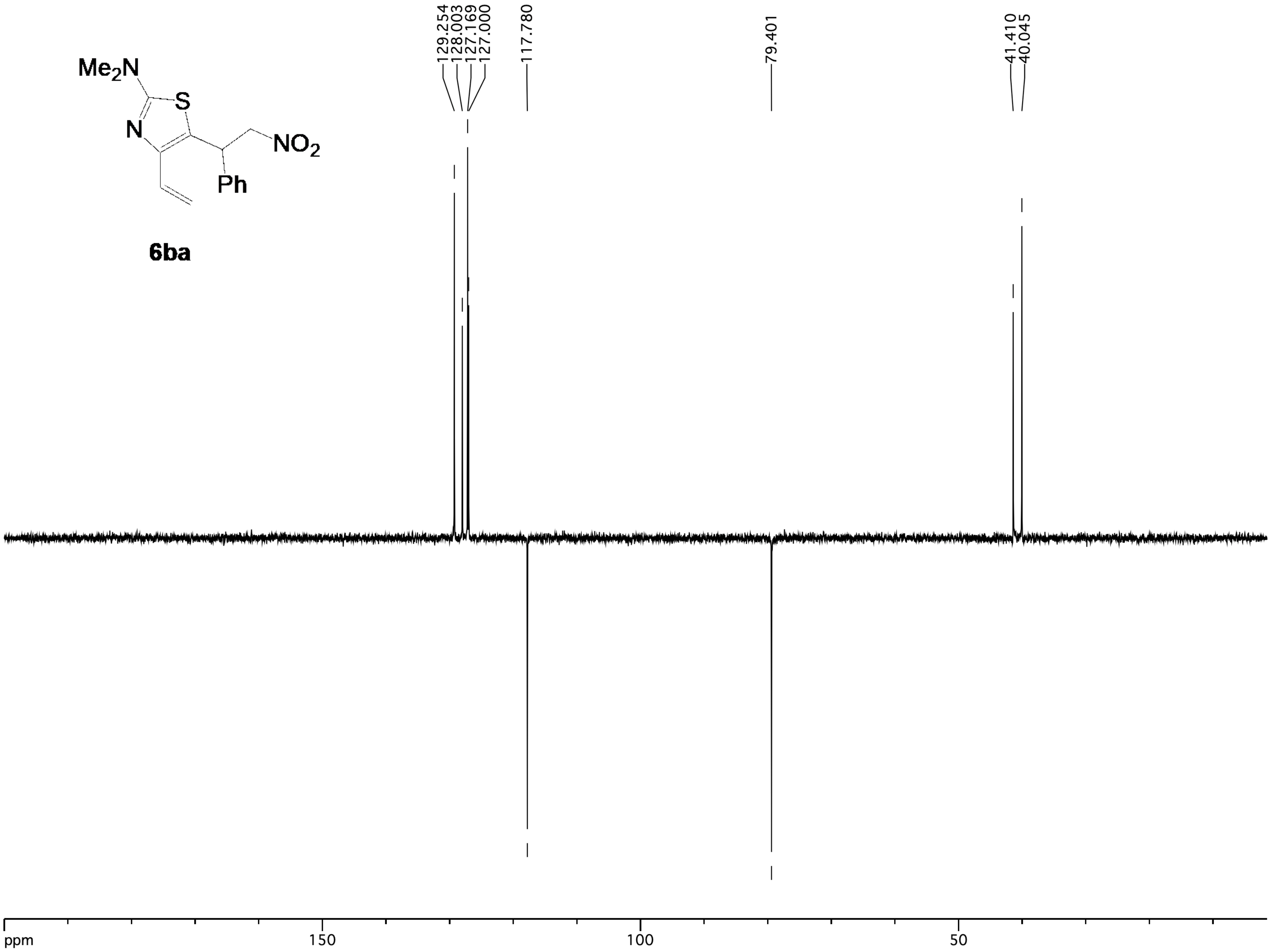

DEPT-135 ($CDCl_3$, 150 MHz) of compound **6ba**.

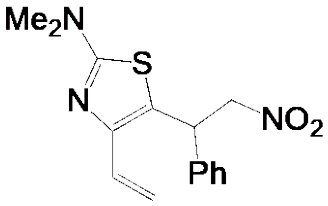

6ba

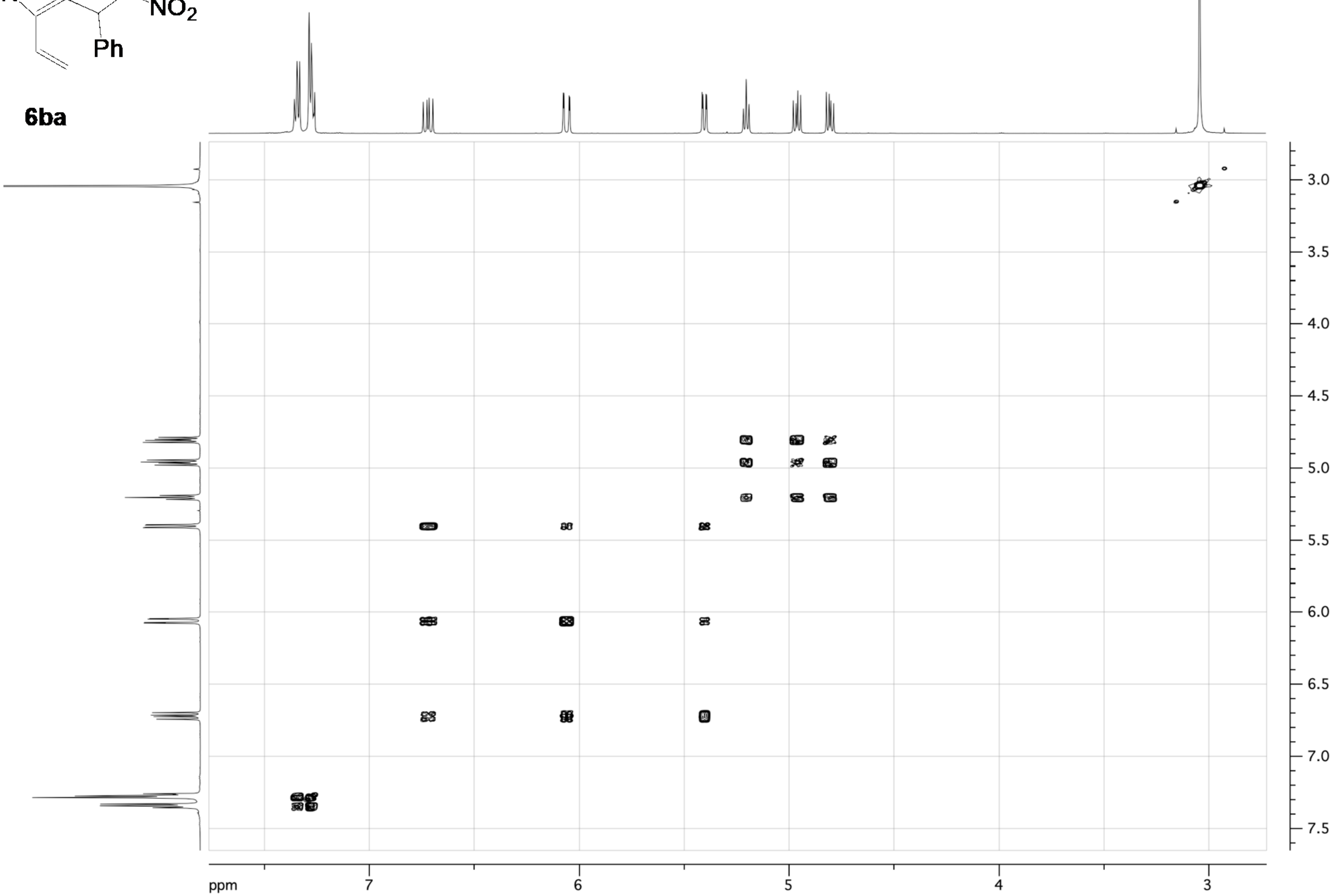

COSY ($CDCl_3$, 600 MHz) of compound **6ba**.

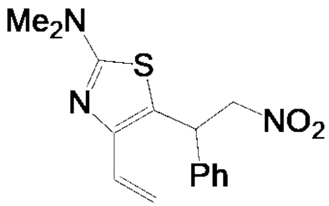

6ba

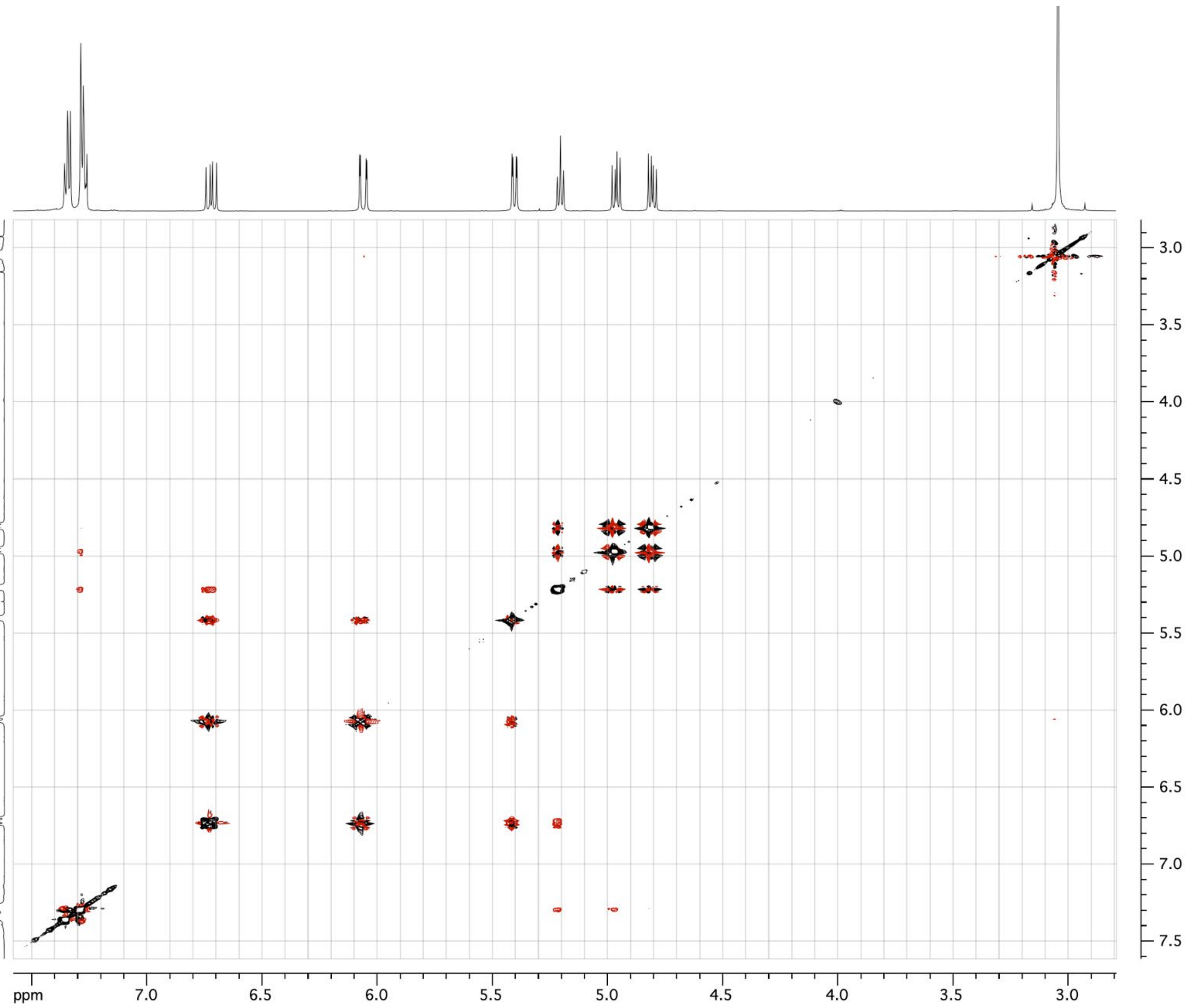

NOESY ($CDCl_3$, 600 MHz) of compound **6ba**.

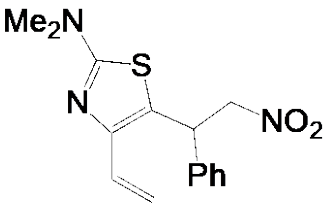

6ba

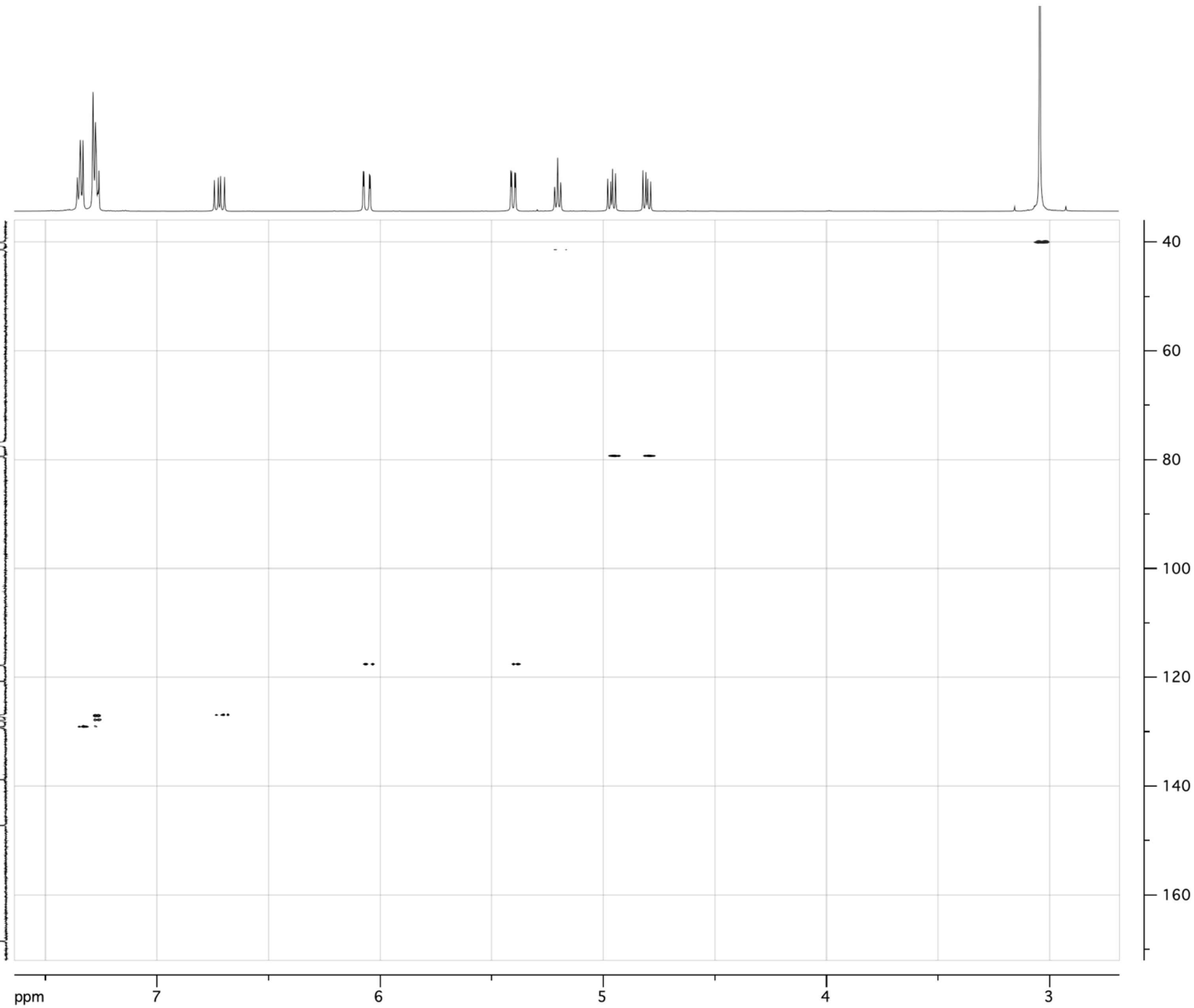

HSQC (CDCl₃, 600 MHz) of compound **6ba**.

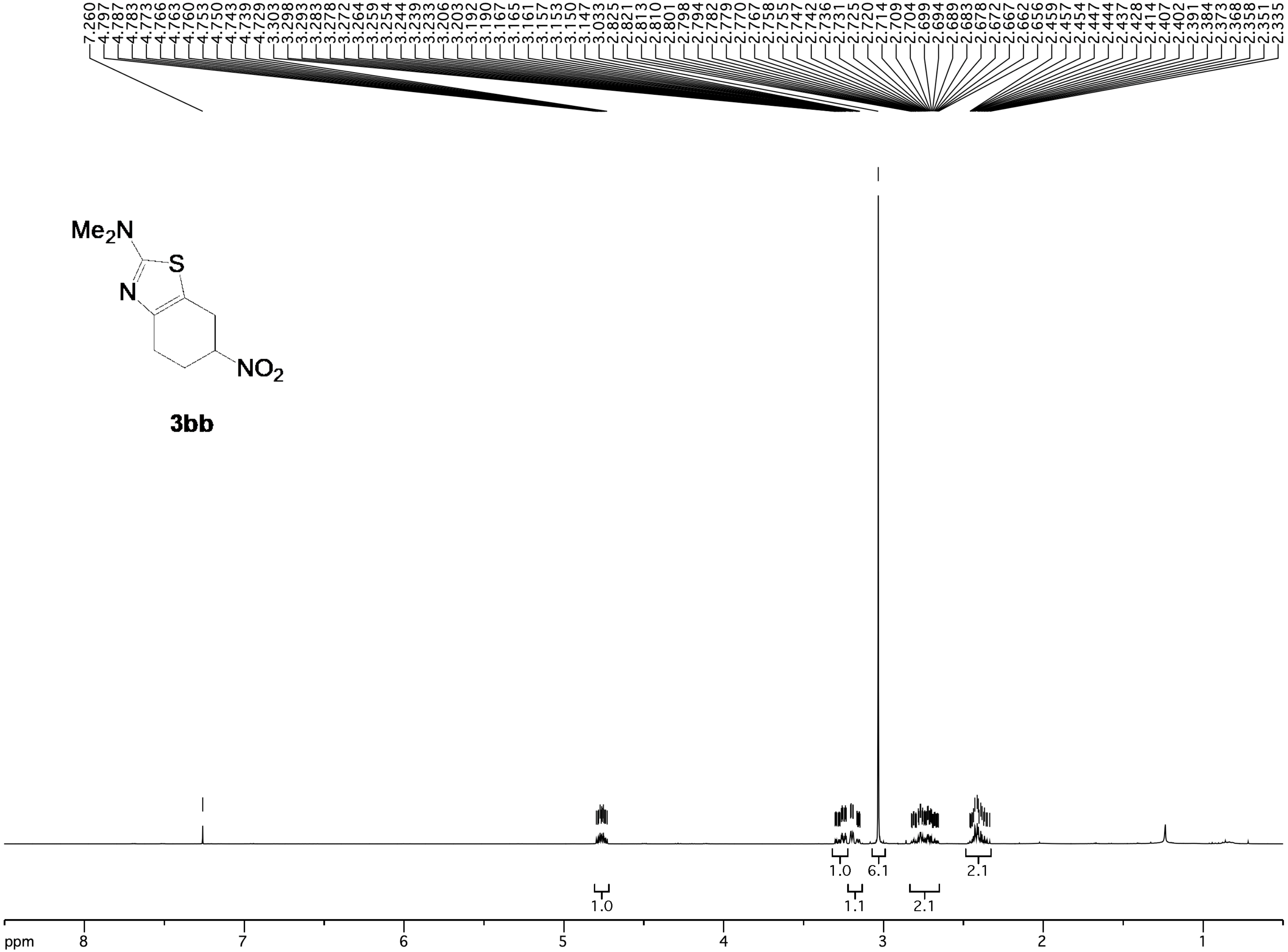

^{1}H NMR ($CDCl_3$, 400 MHz) of compound **3bb**.

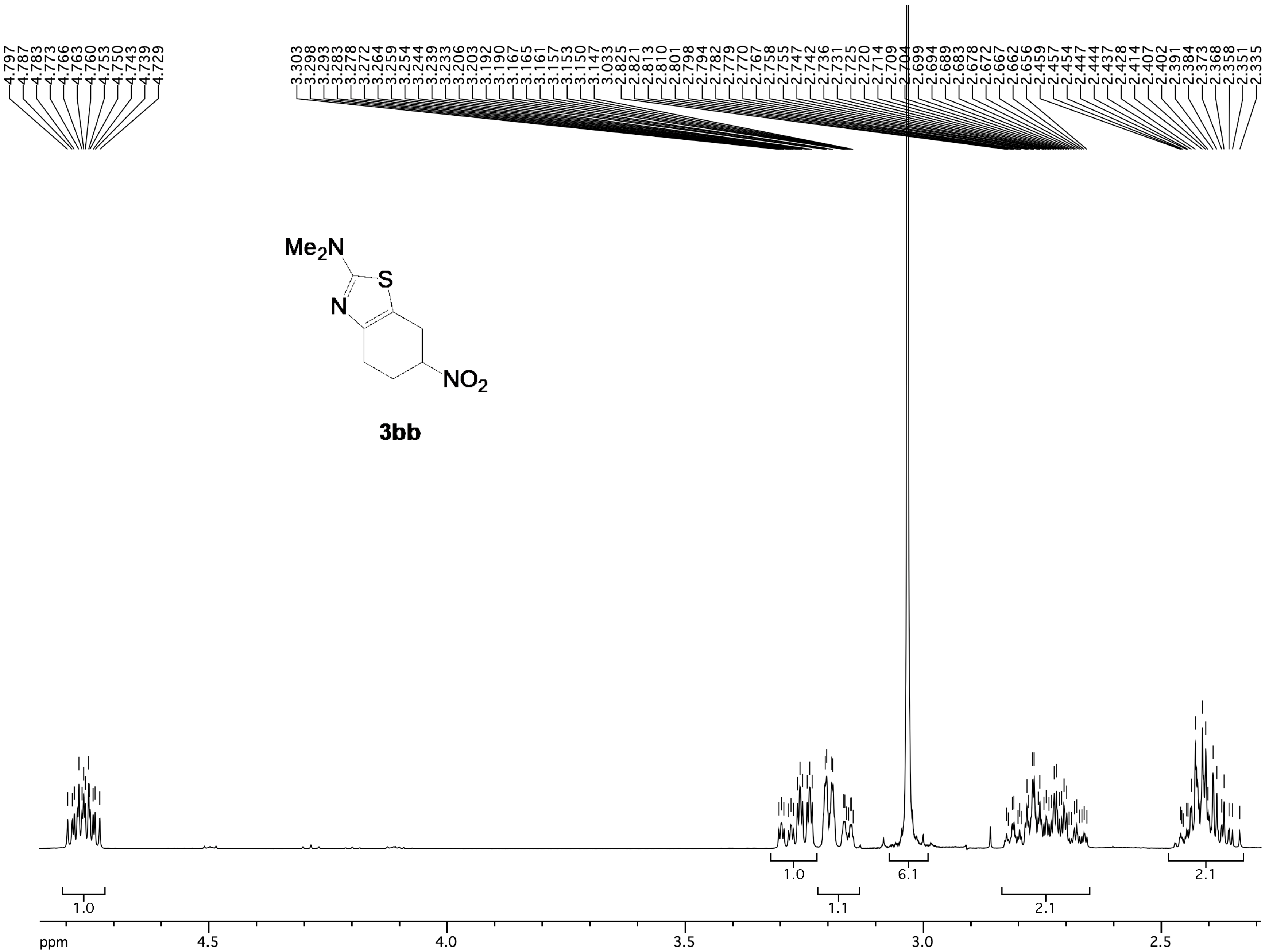

^{1}H NMR ($CDCl_3$, 400 MHz) of compound **3bb** (expansion).

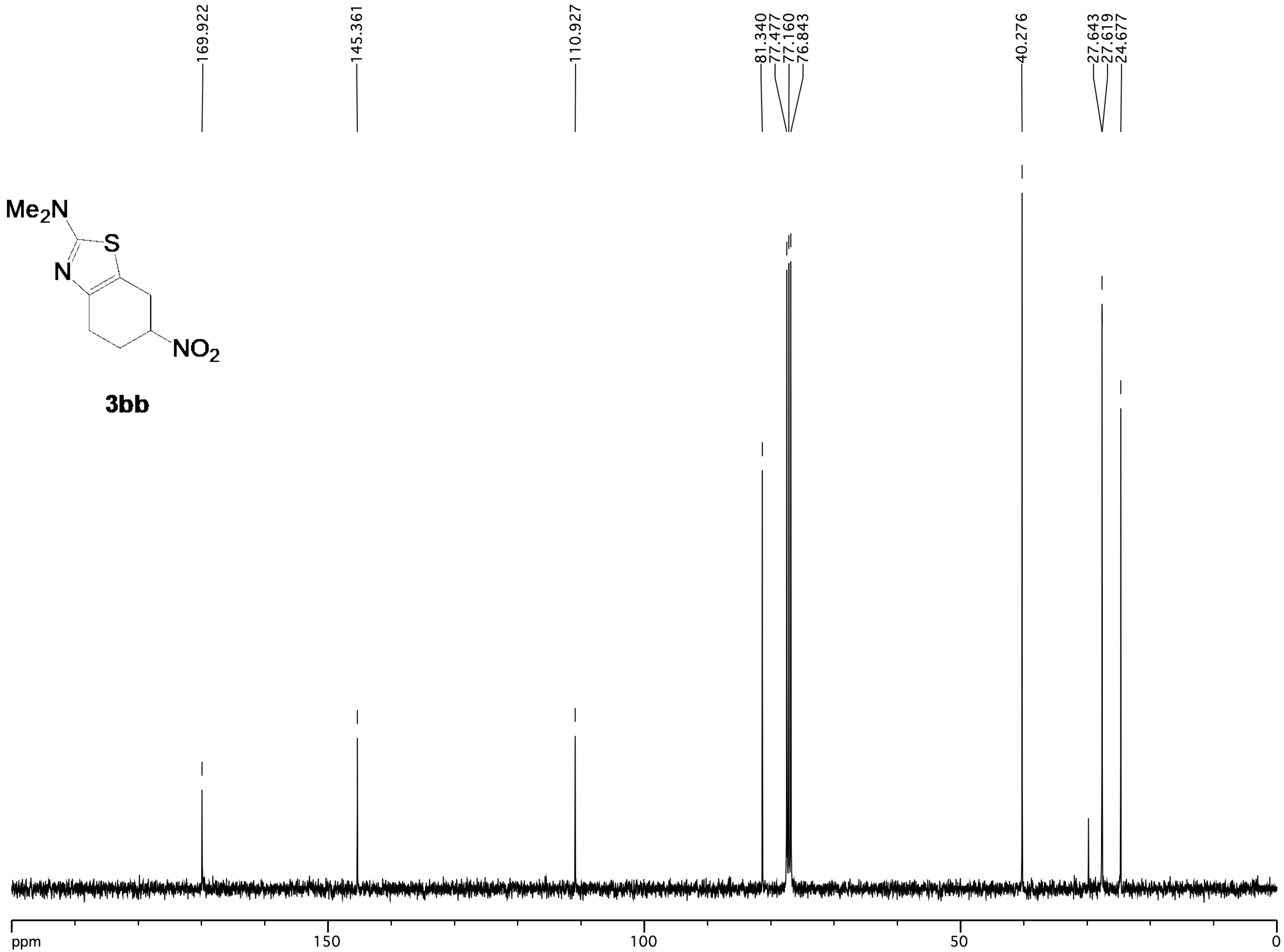

$^{13}C\{^{1}H\}$ NMR ($CDCl_3$, 100 MHz) of compound **3bb**.

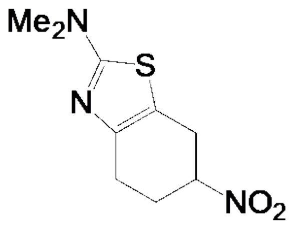

3bb

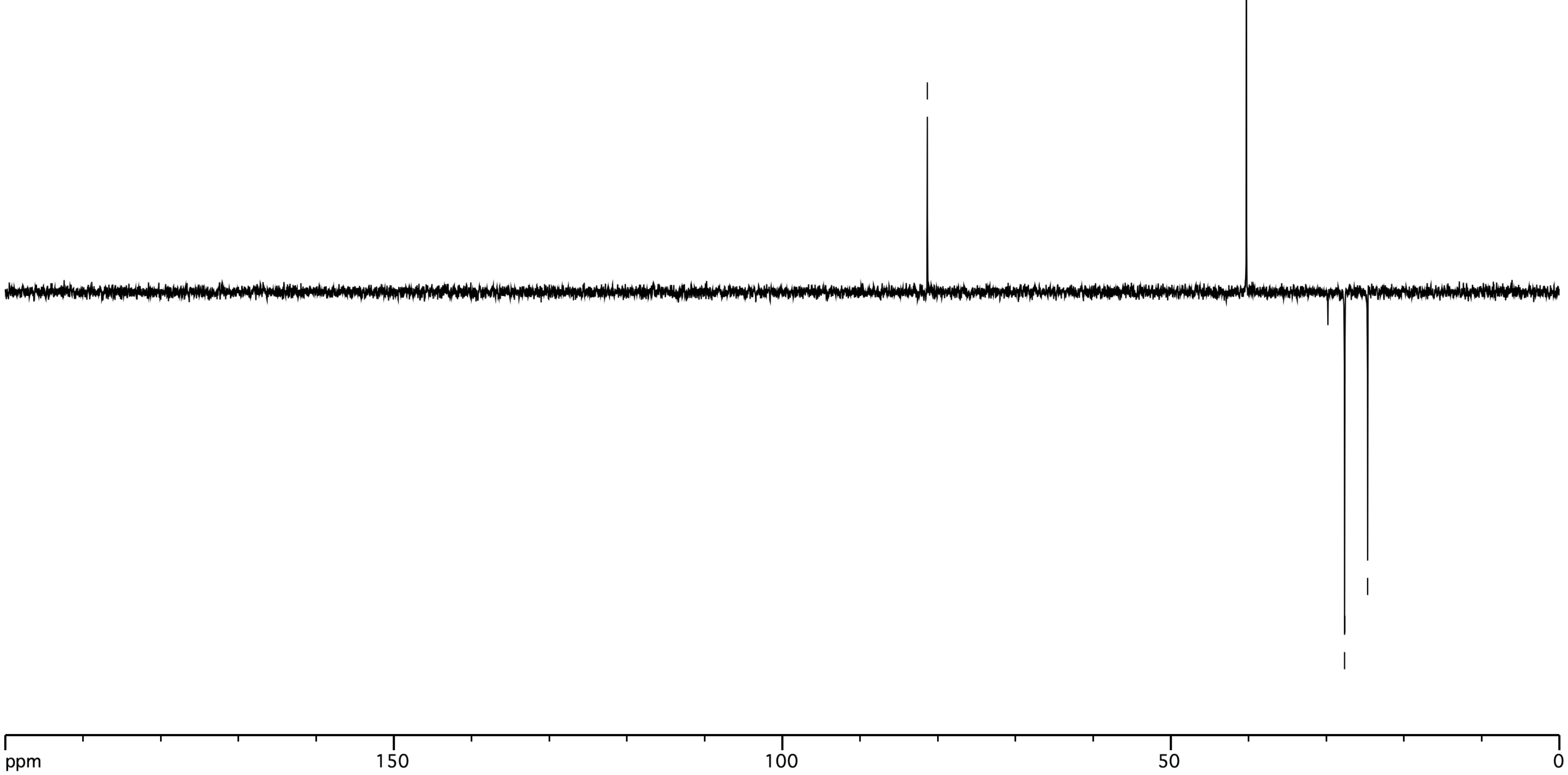

DEPT-135 ($CDCl_3$, 100 MHz) of compound **3bb**.

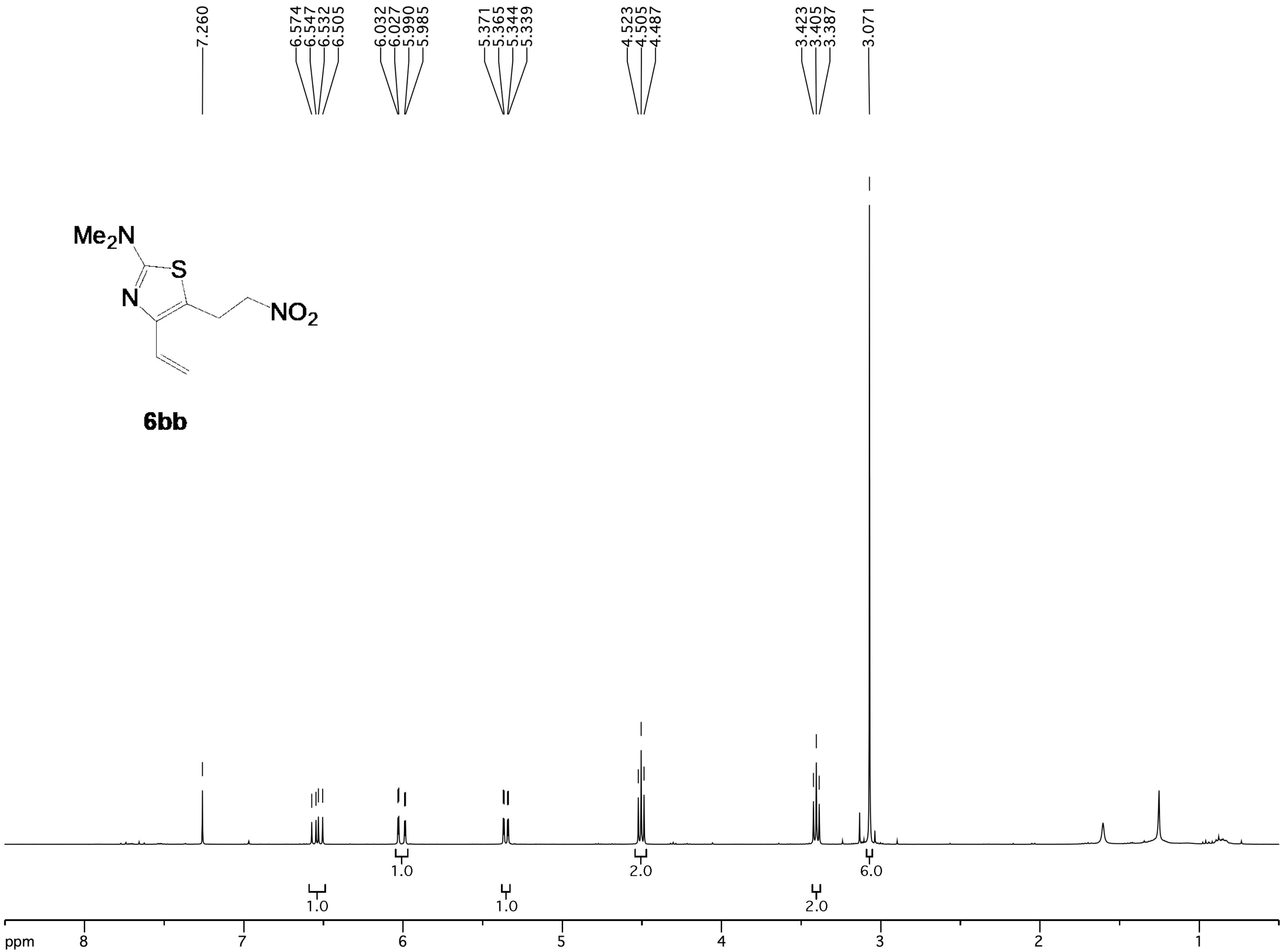

^{1}H NMR ($CDCl_3$, 400 MHz) of compound **6bb**.

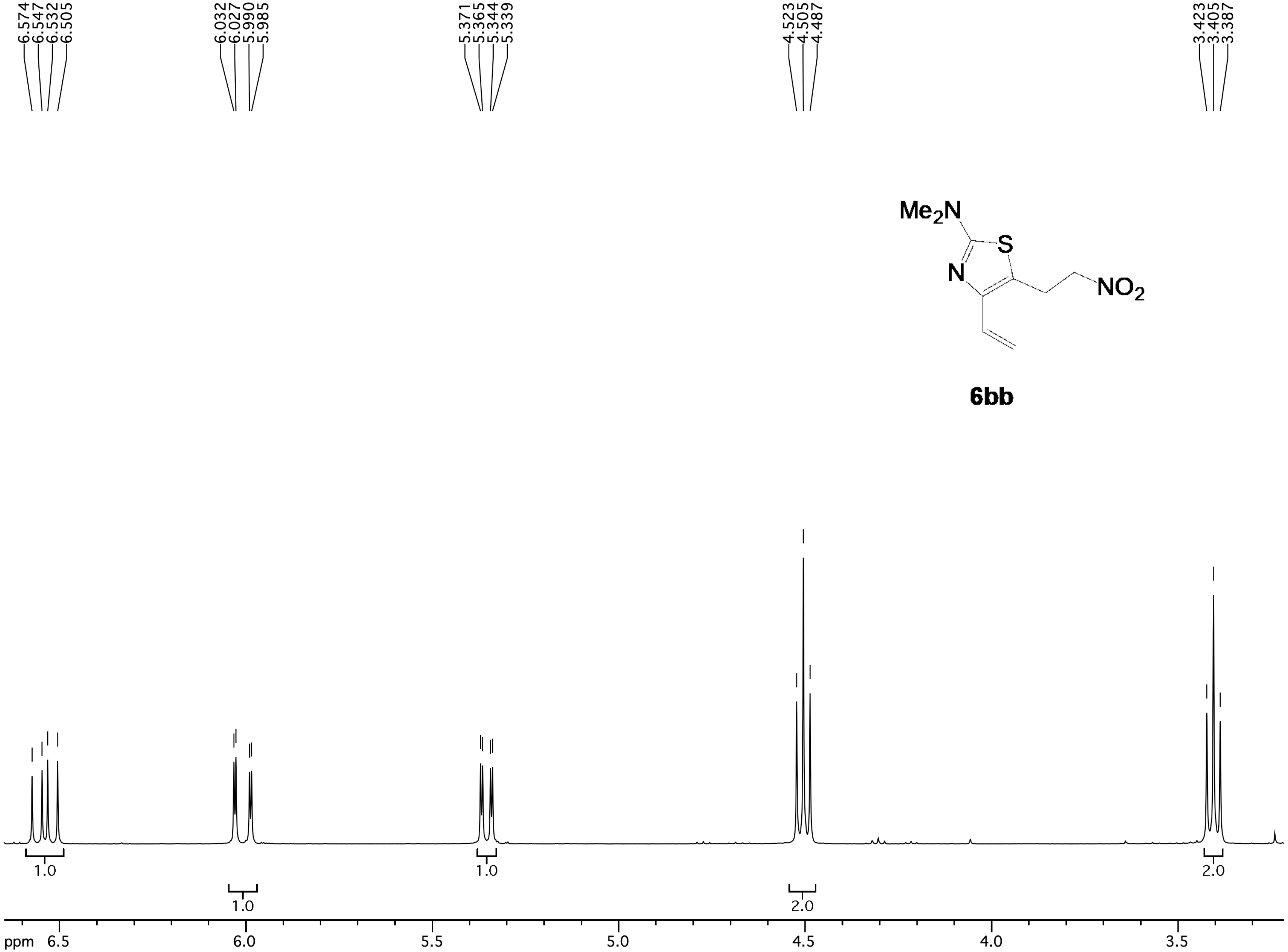

^{1}H NMR ($CDCl_3$, 400 MHz) of compound **6bb** (expansion).

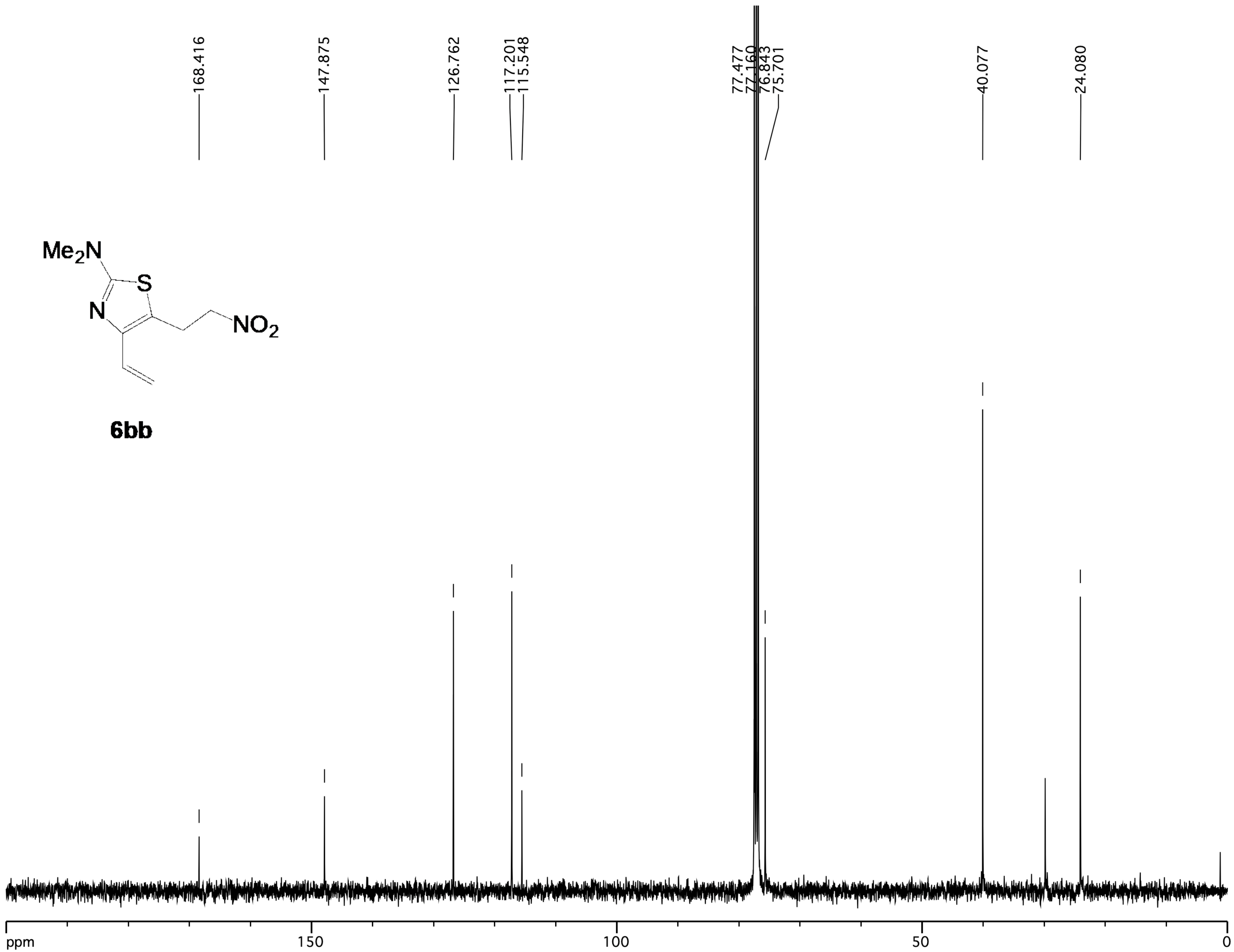

6bb

$^{13}C\{^{1}H\}$ NMR ($CDCl_3$, 100 MHz) of compound **6bb**.

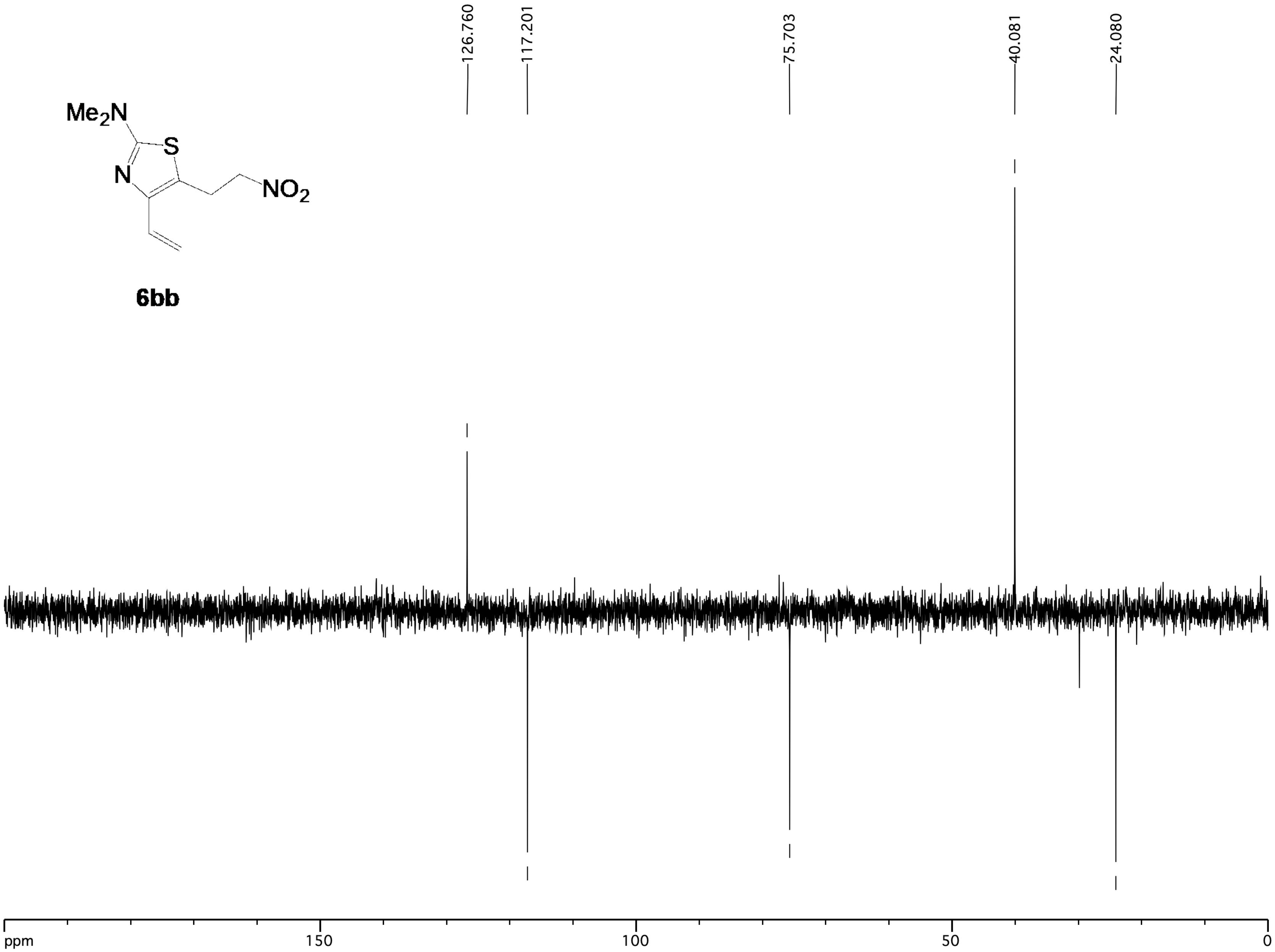

DEPT-135 ($CDCl_3$, 100 MHz) of compound **6bb**.

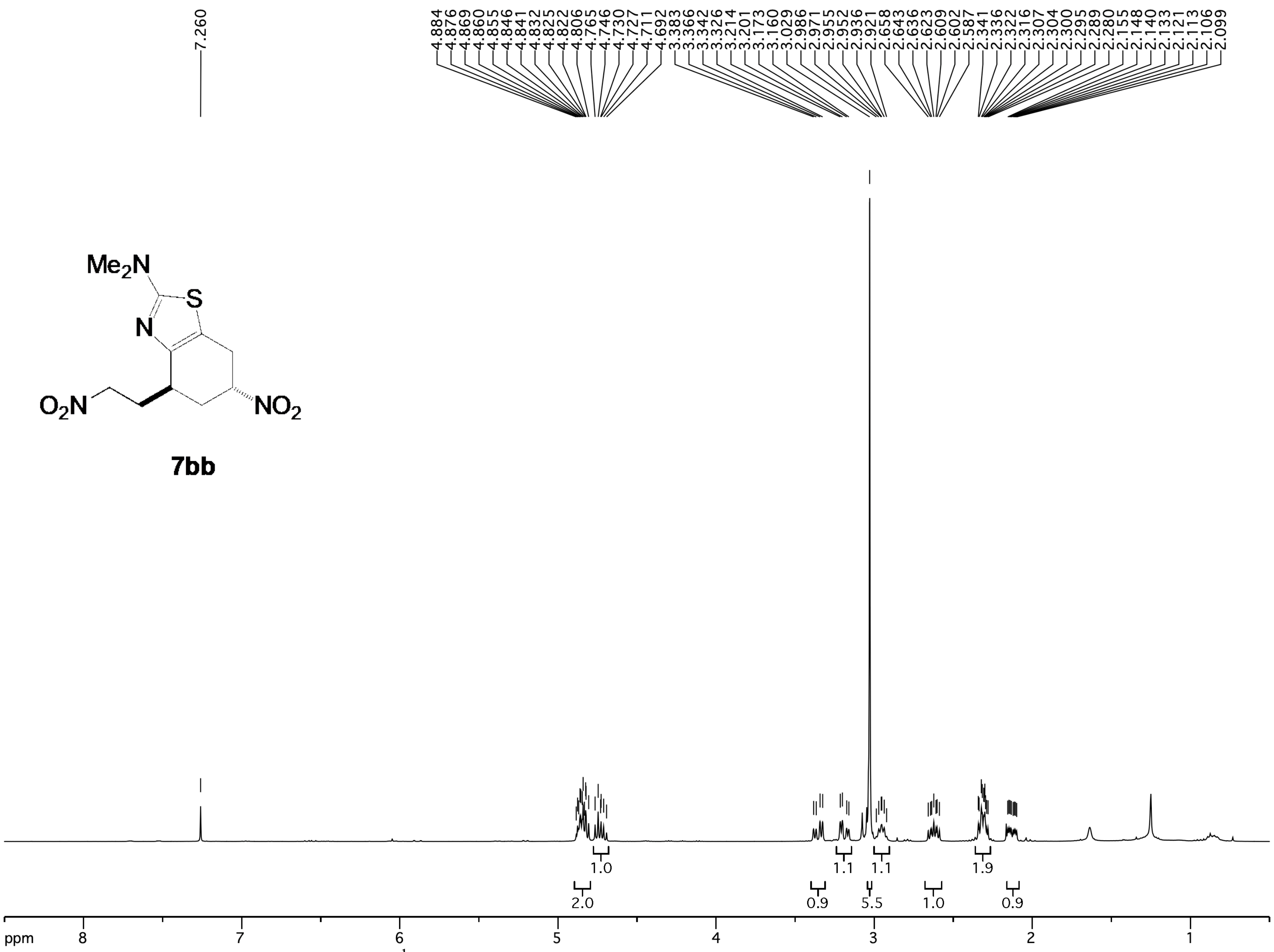

^{1}H NMR ($CDCl_3$, 400 MHz) of compound **7bb**.

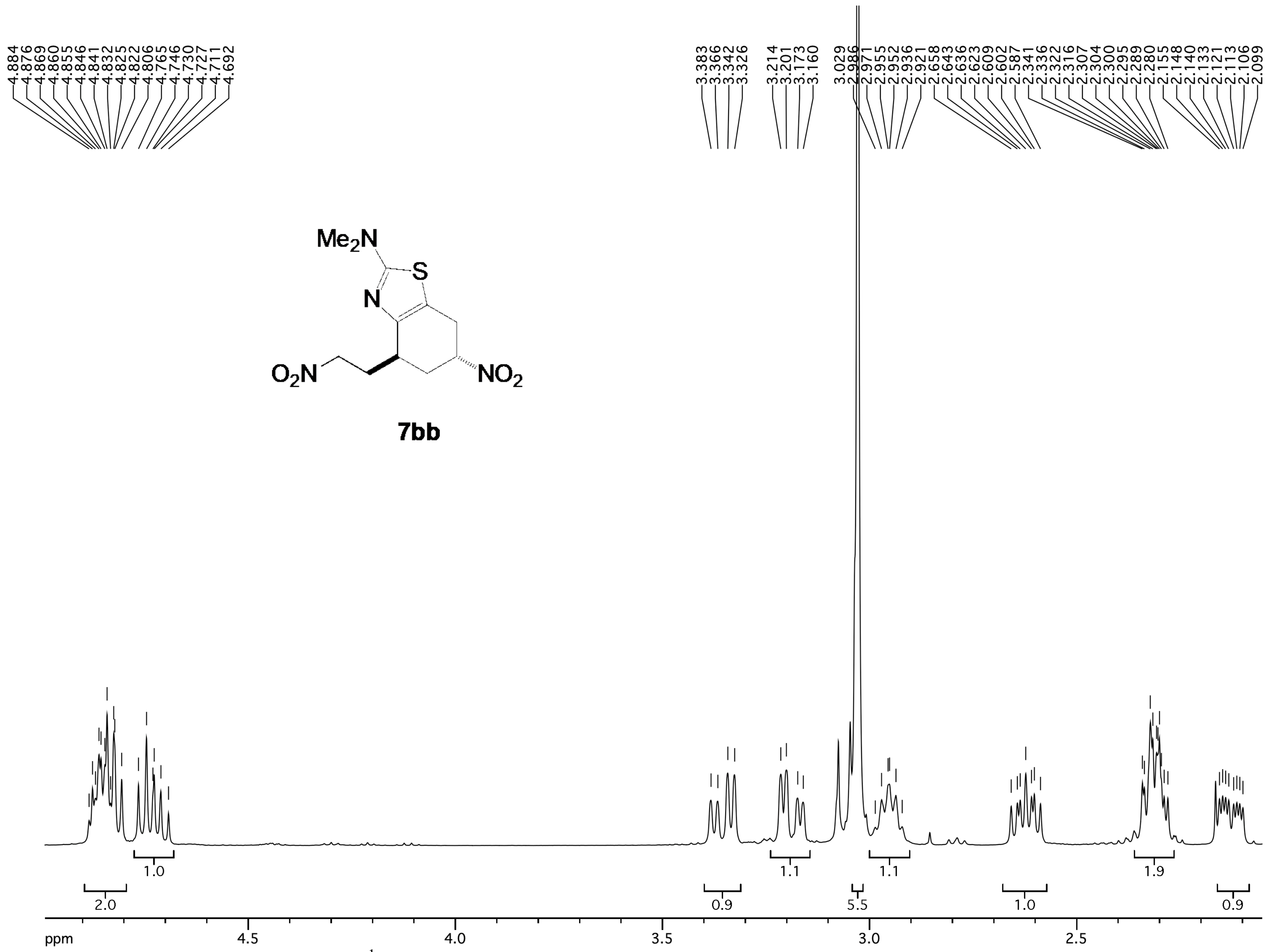

^{1}H NMR ($CDCl_3$, 400 MHz) of compound **7bb** (expansion).

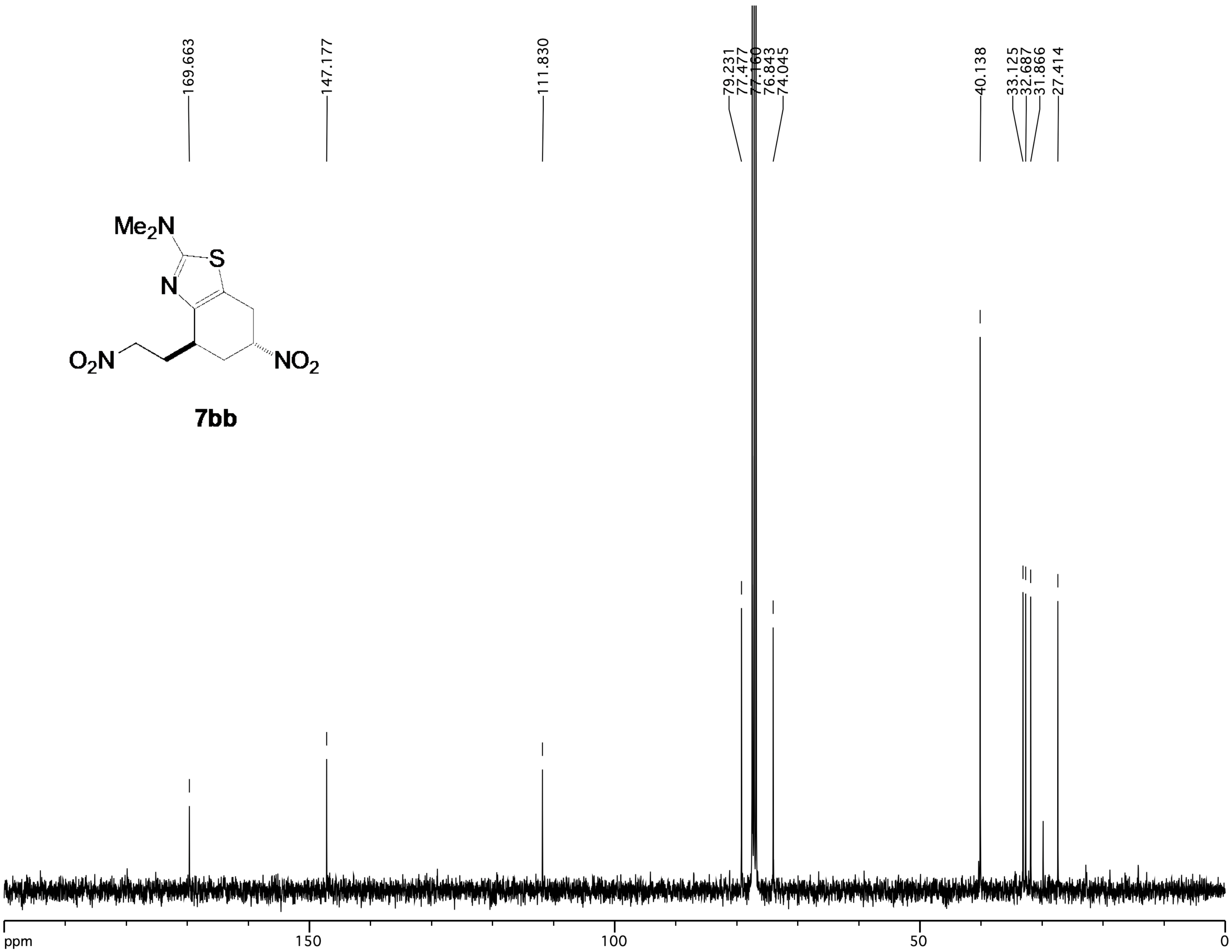

$^{13}C\{^{1}H\}$ NMR ($CDCl_3$, 100 MHz) of compound **7bb**.

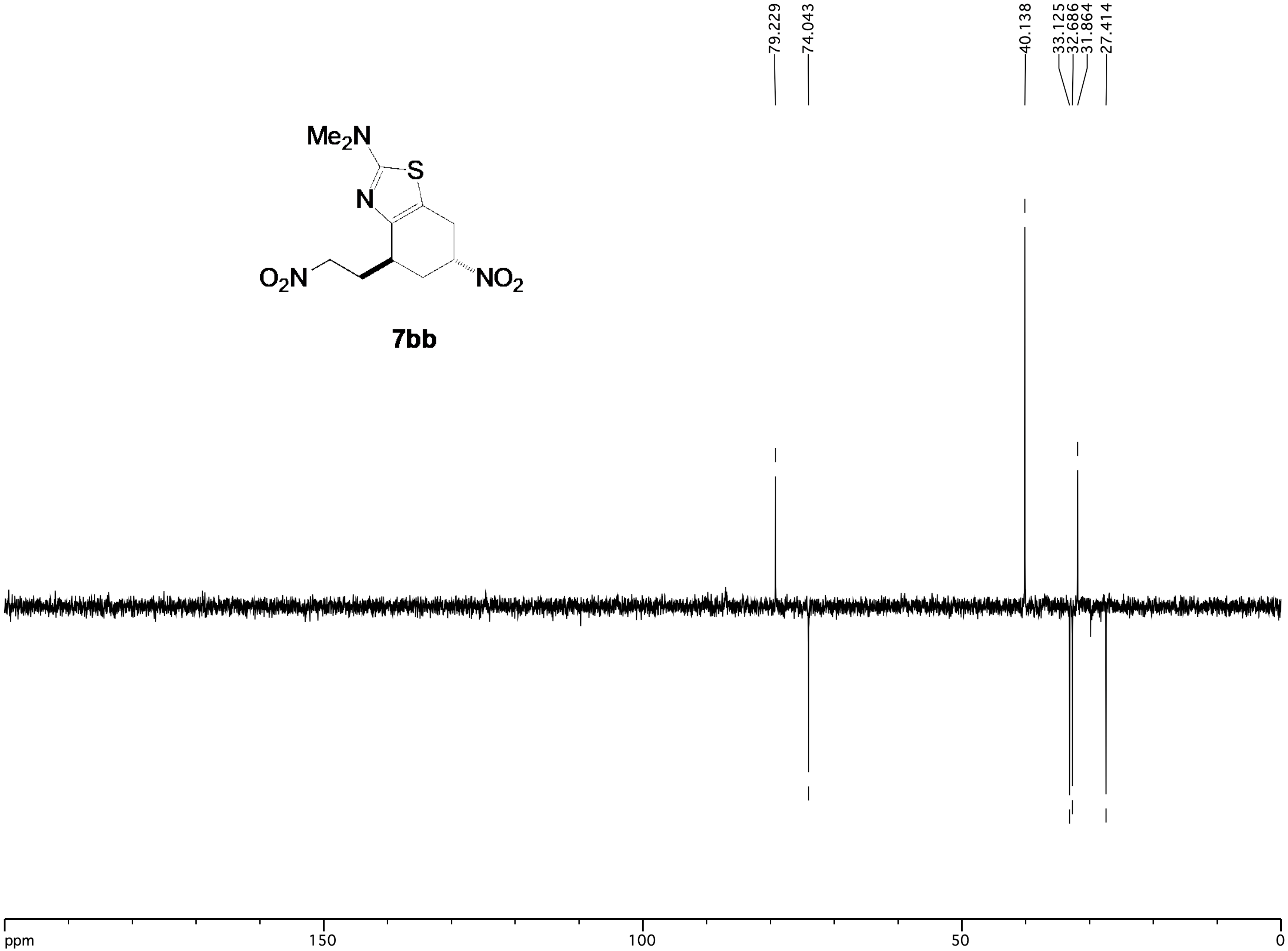

DEPT-135 ($CDCl_3$, 100 MHz) of compound **7bb**.

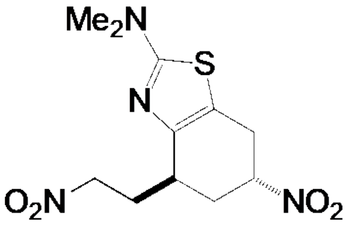

7bb

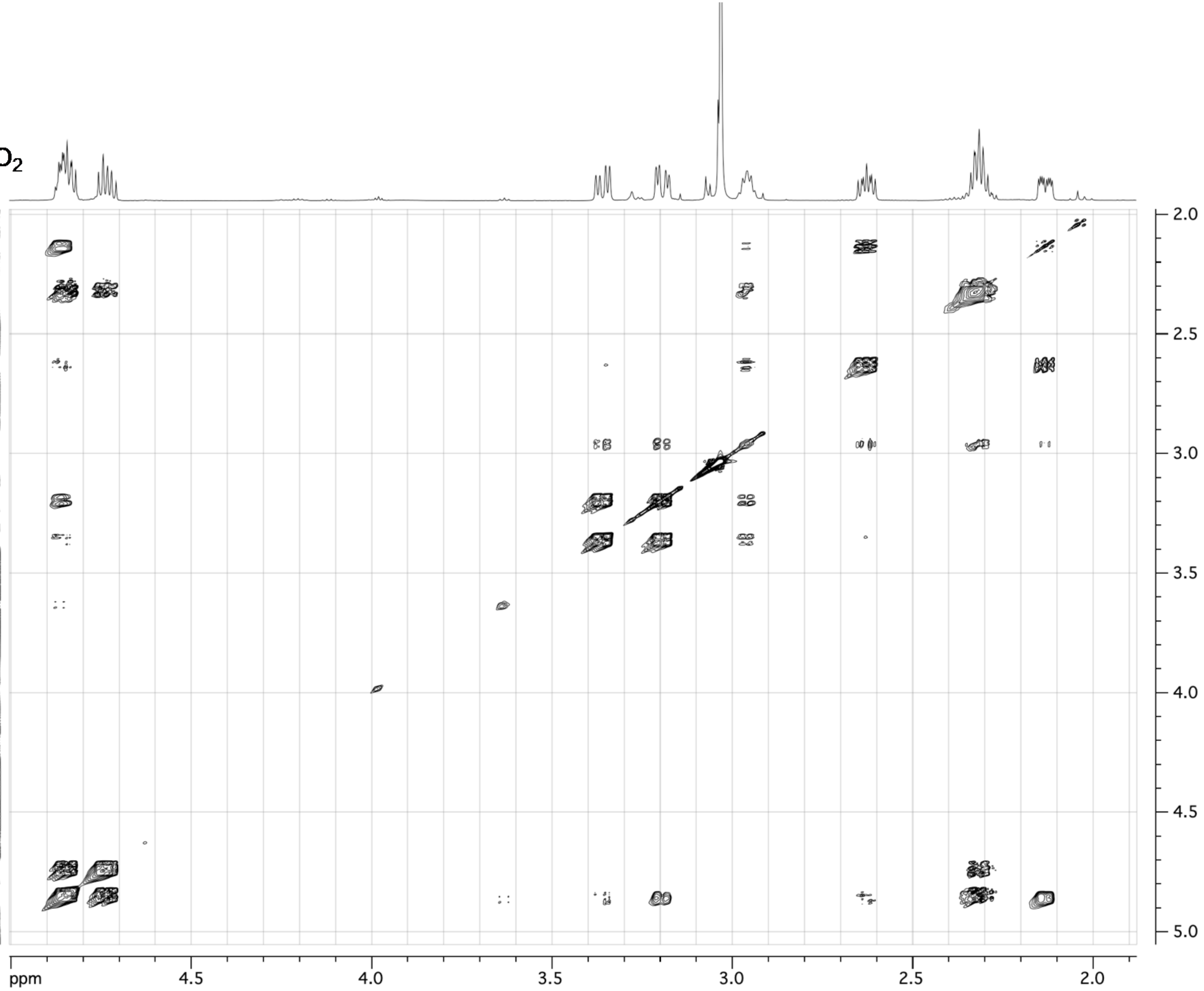

COSY ($CDCl_3$, 600 MHz) of compound **7bb**.

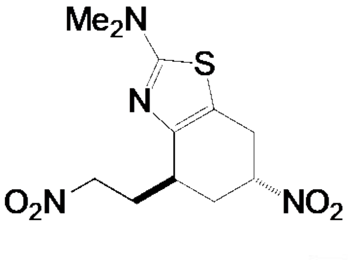

7bb

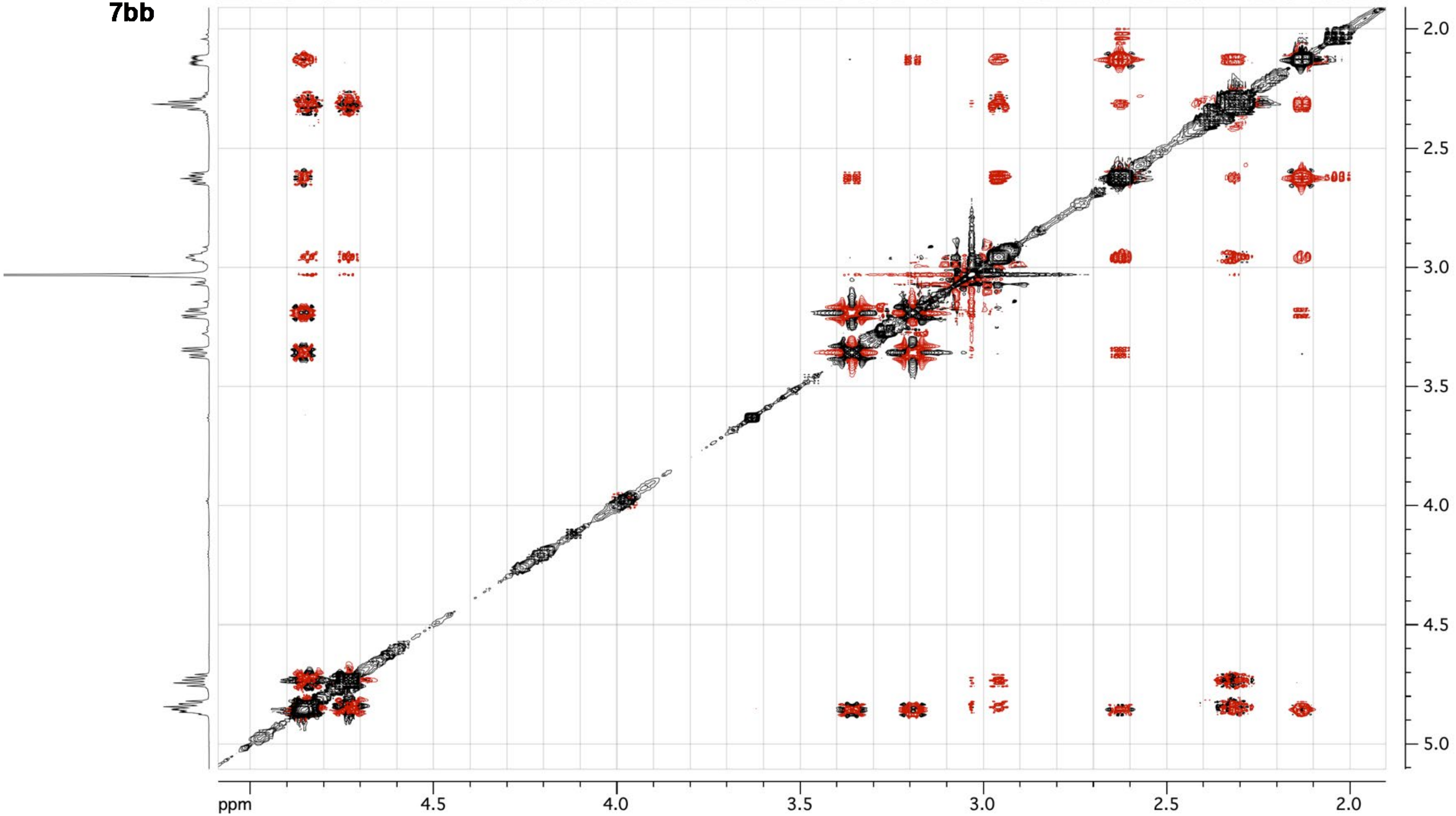

NOESY ($CDCl_3$, 600 MHz) of compound **7bb**.

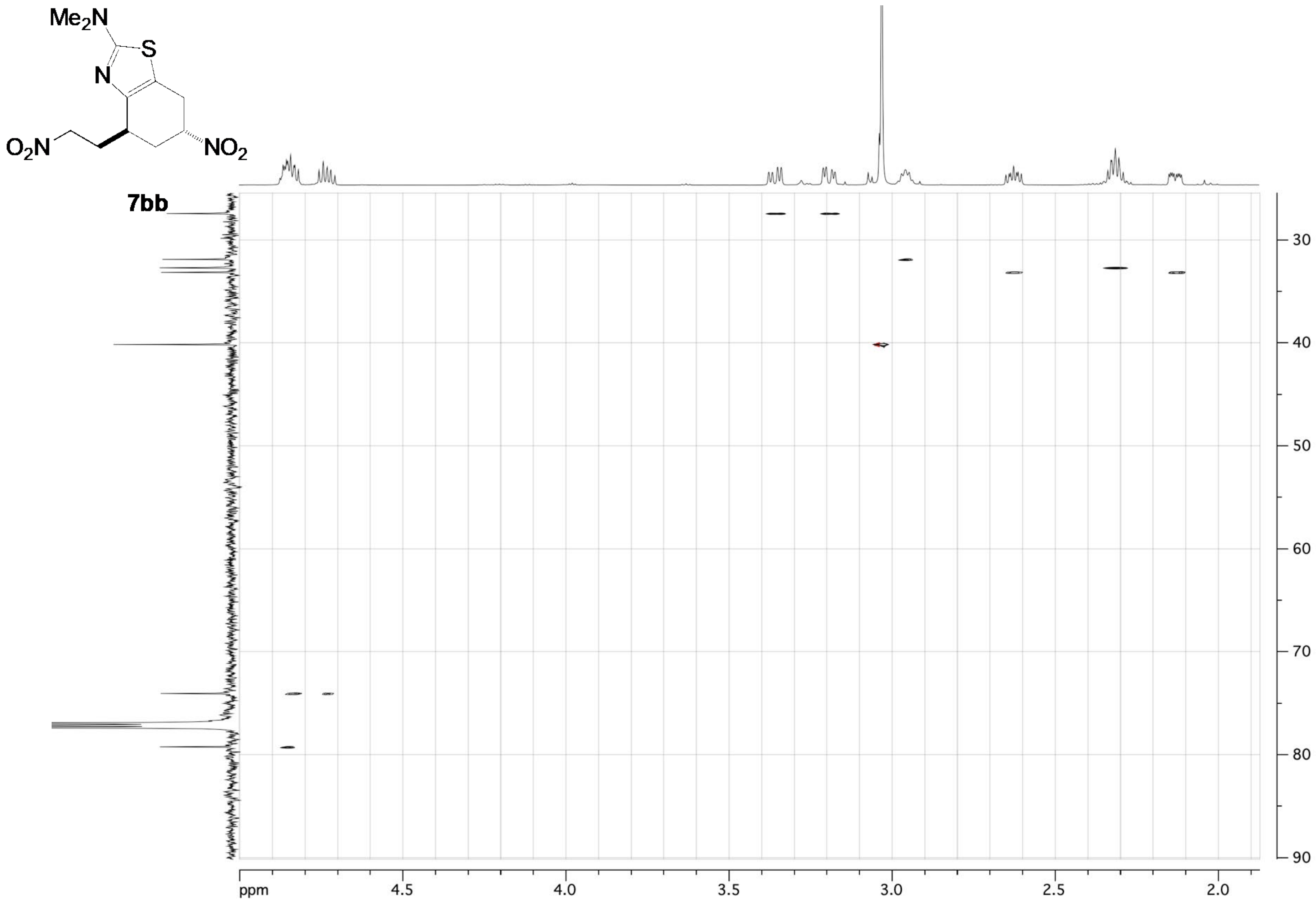

HSQC (CDCl3, 600 MHz) of compound **7bb**.

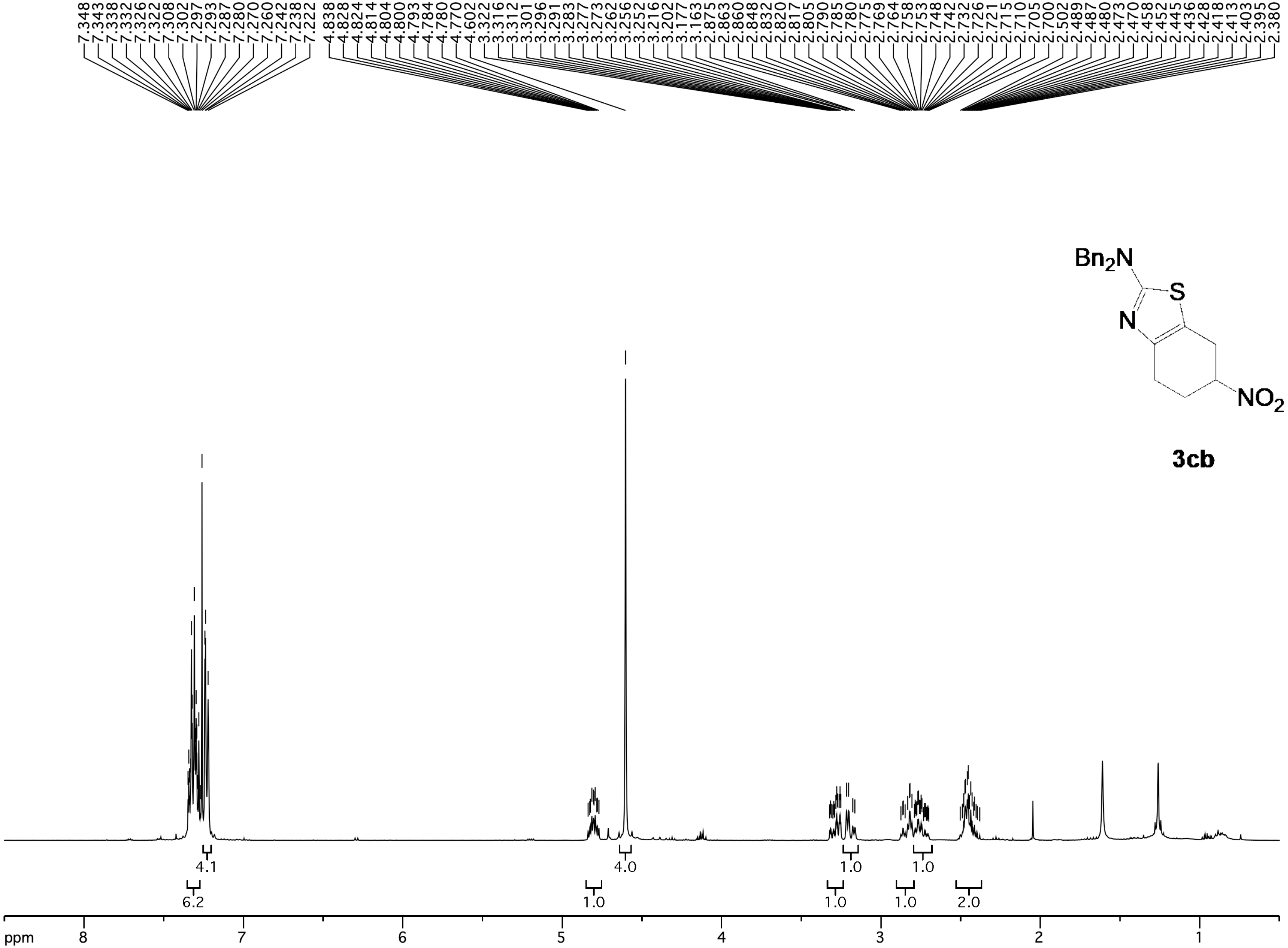

^{1}H NMR (CDCl$_3$, 400 MHz) of compound **3cb**.

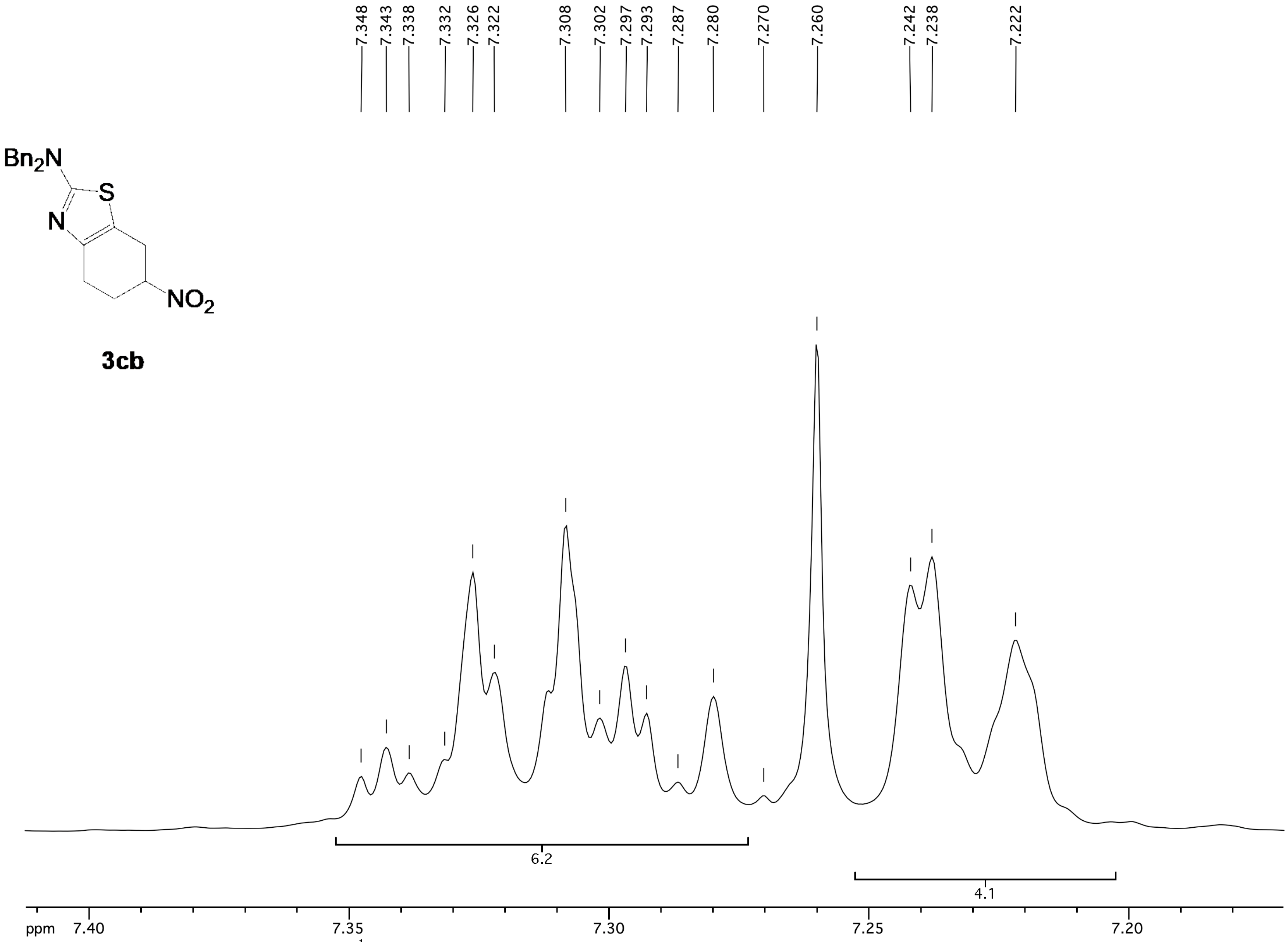

^{1}H NMR ($CDCl_3$, 400 MHz) of compound **3cb** (expansion).

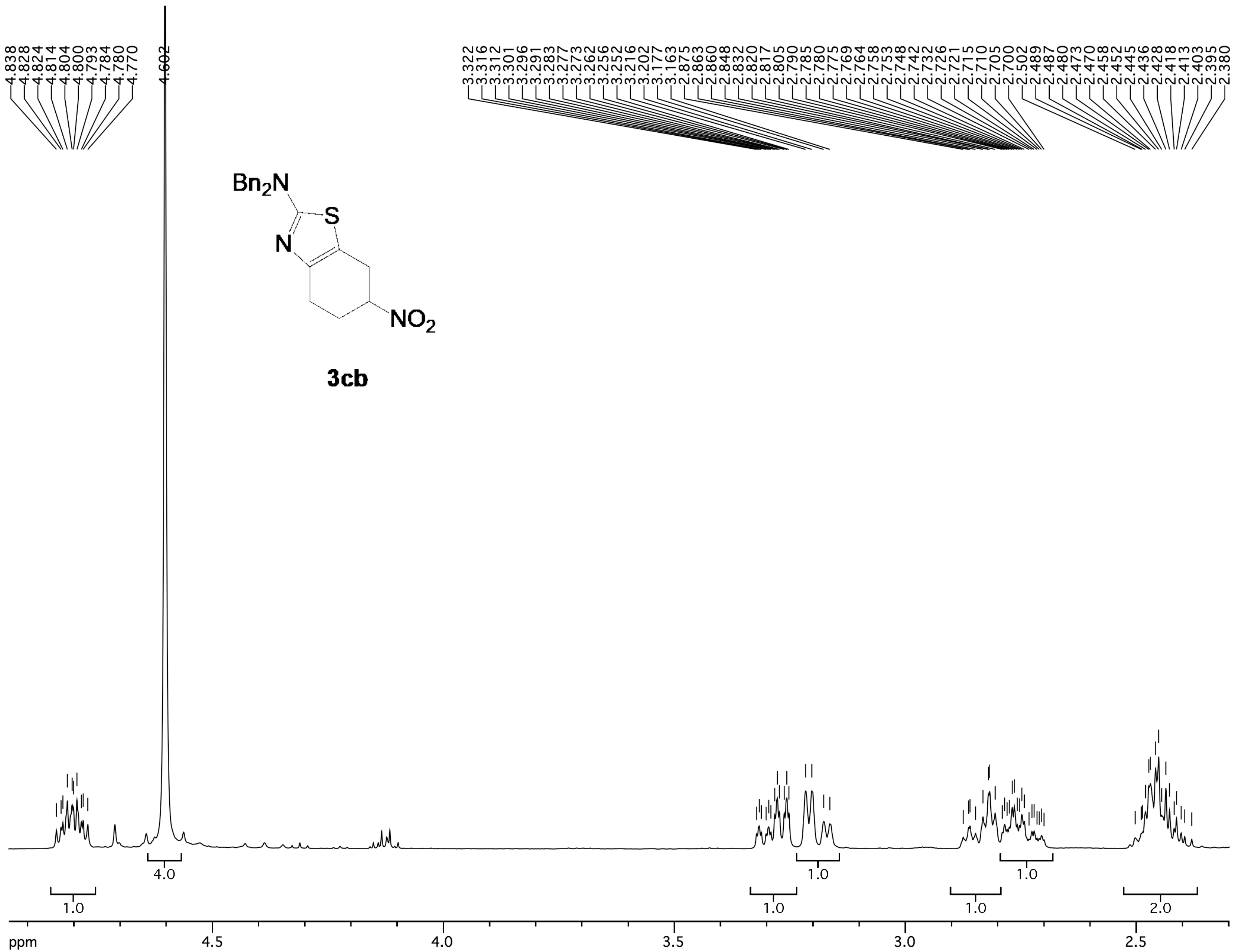

^{1}H NMR (CDCl$_3$, 400 MHz) of compound **3cb** (expansion).

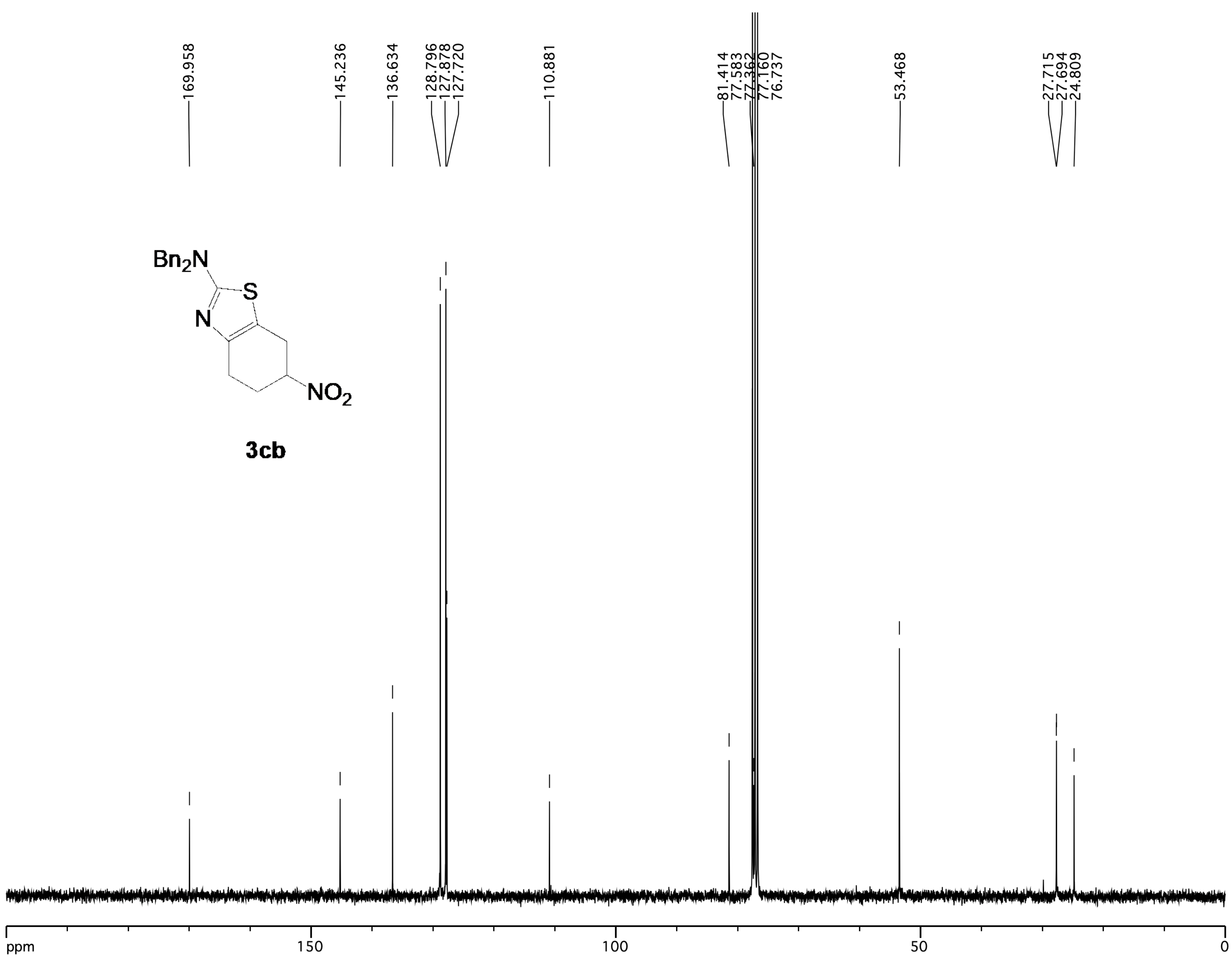

$^{13}C\{^{1}H\}$ NMR ($CDCl_3$, 75 MHz) of compound **3cb**.

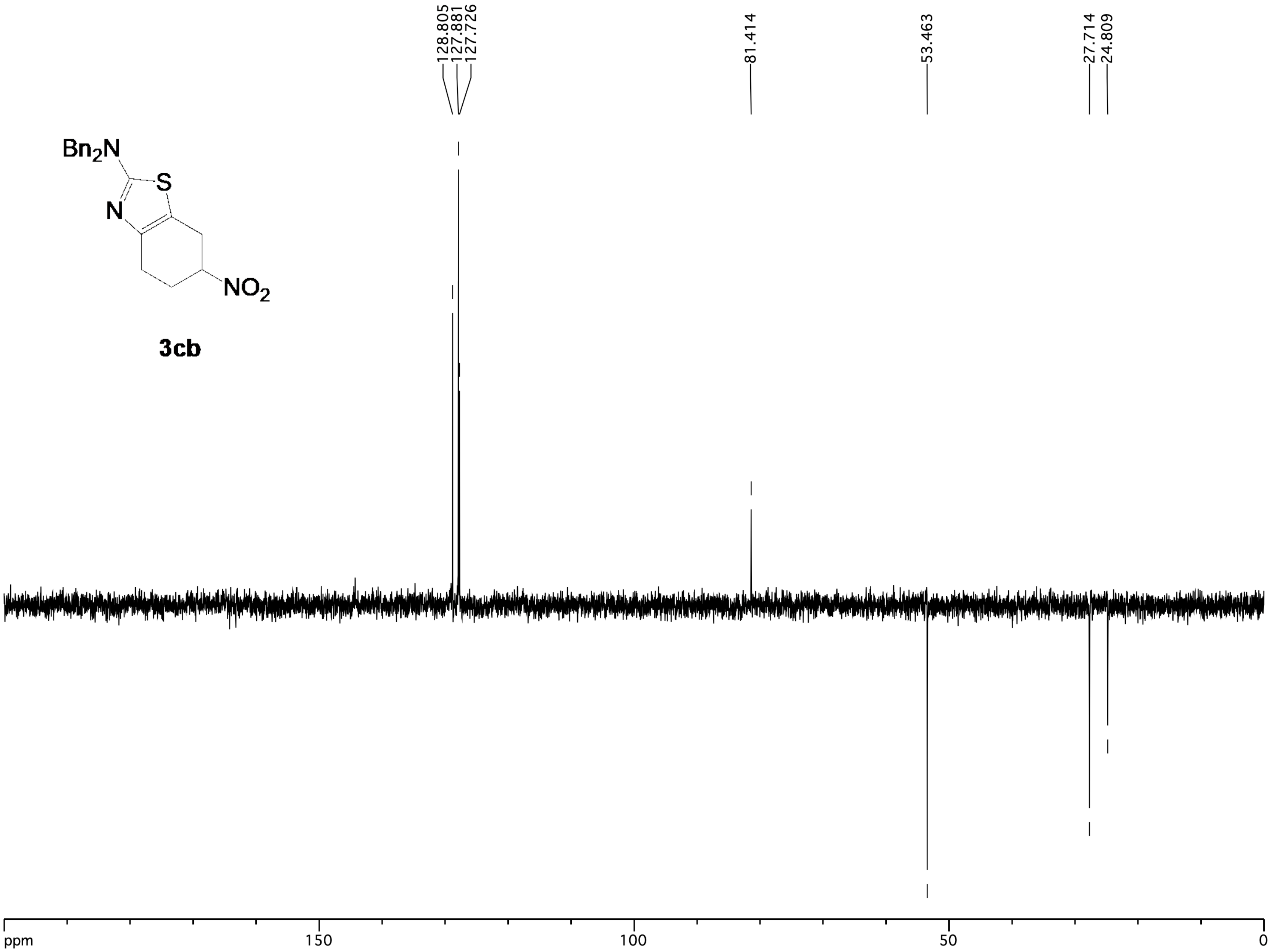

DEPT-135 ($CDCl_3$, 75 MHz) of compound **3cb**.

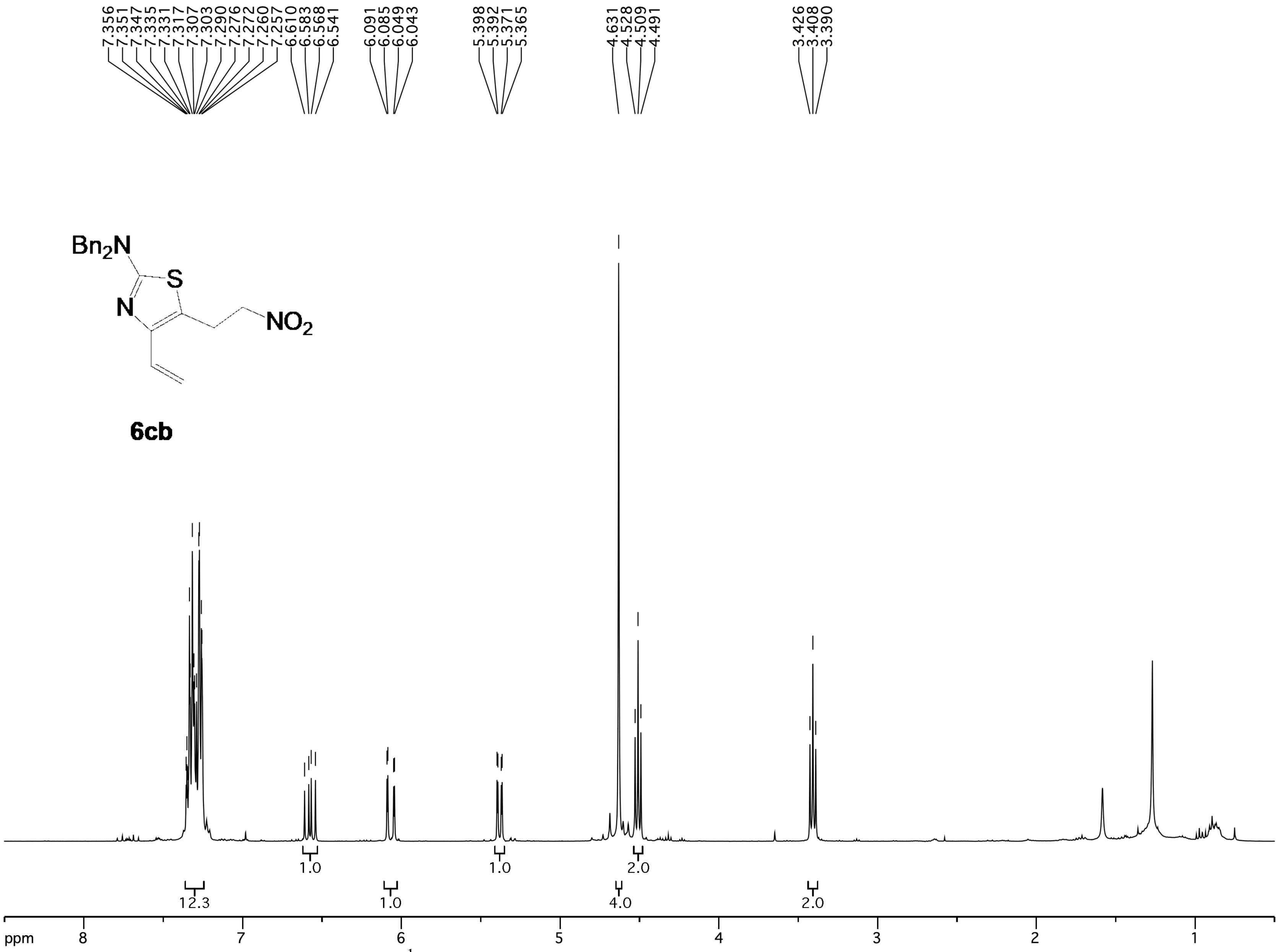

^{1}H NMR (CDCl$_3$, 400 MHz) of compound **6cb**.

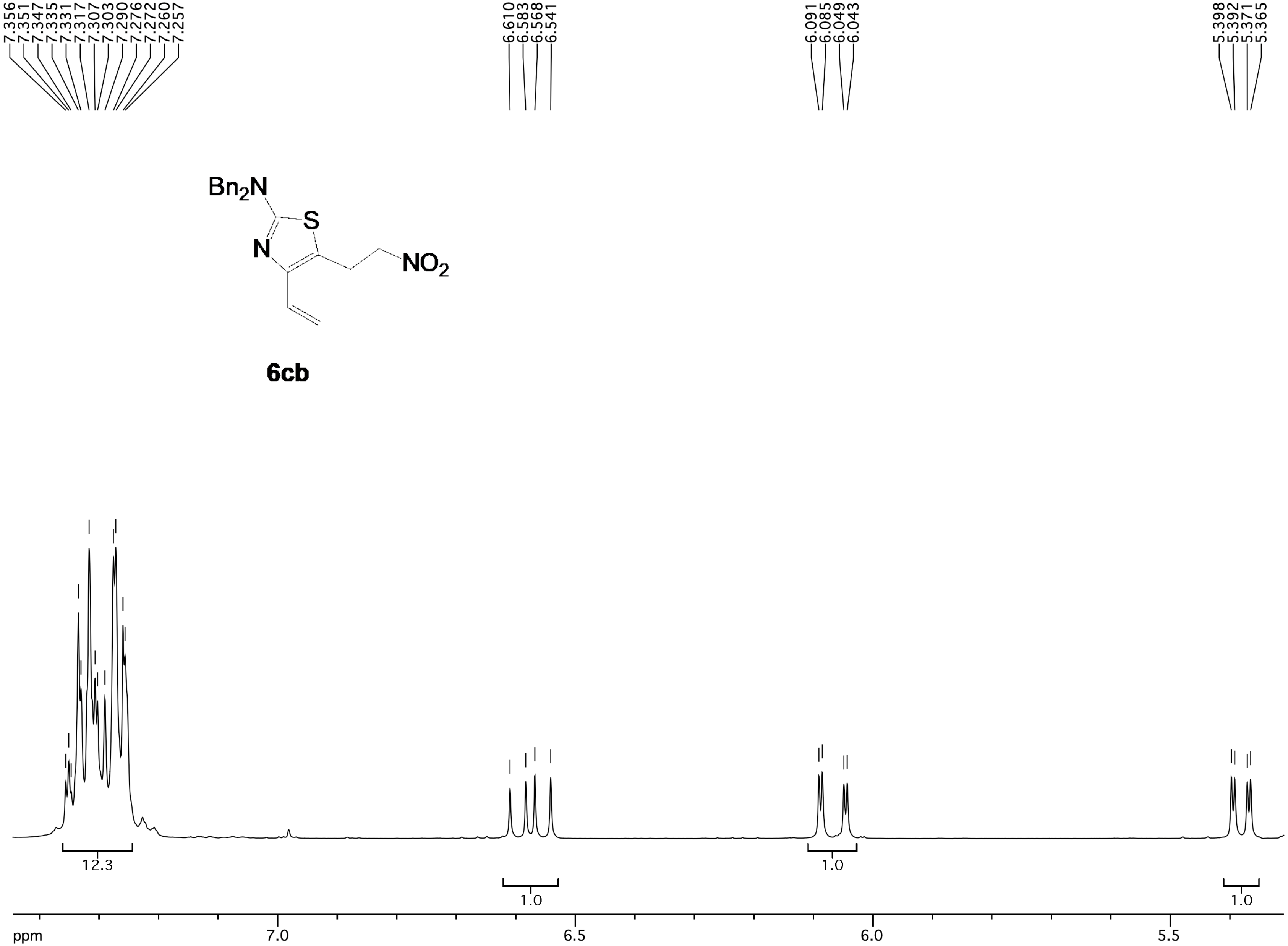

^1H NMR ($CDCl_3$, 400 MHz) of compound **6cb** (expansion).

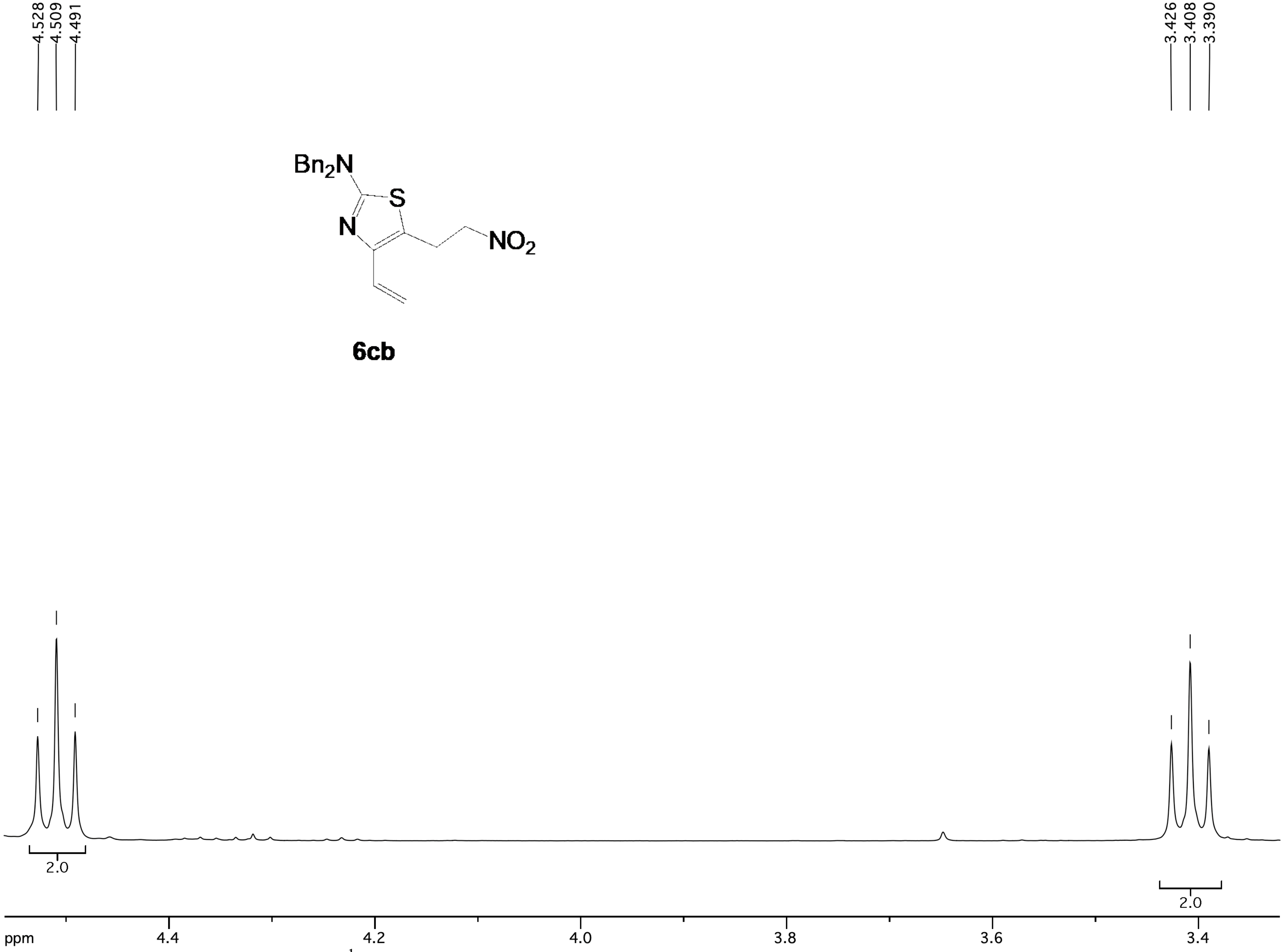

^{1}H NMR ($CDCl_3$, 400 MHz) of compound **6cb** (expansion).

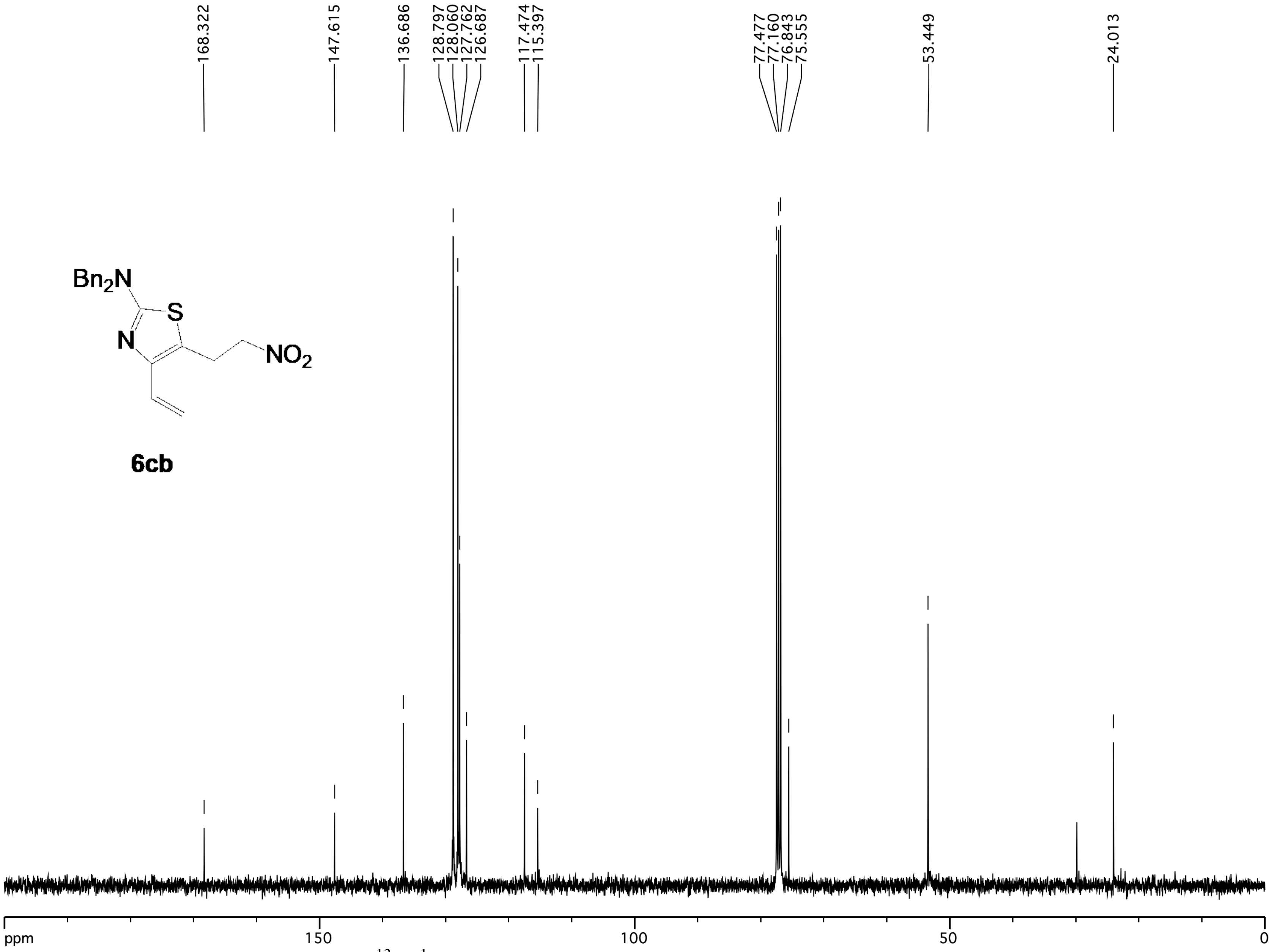

$^{13}C\{^{1}H\}$ NMR ($CDCl_3$, 100 MHz) of compound **6cb**.

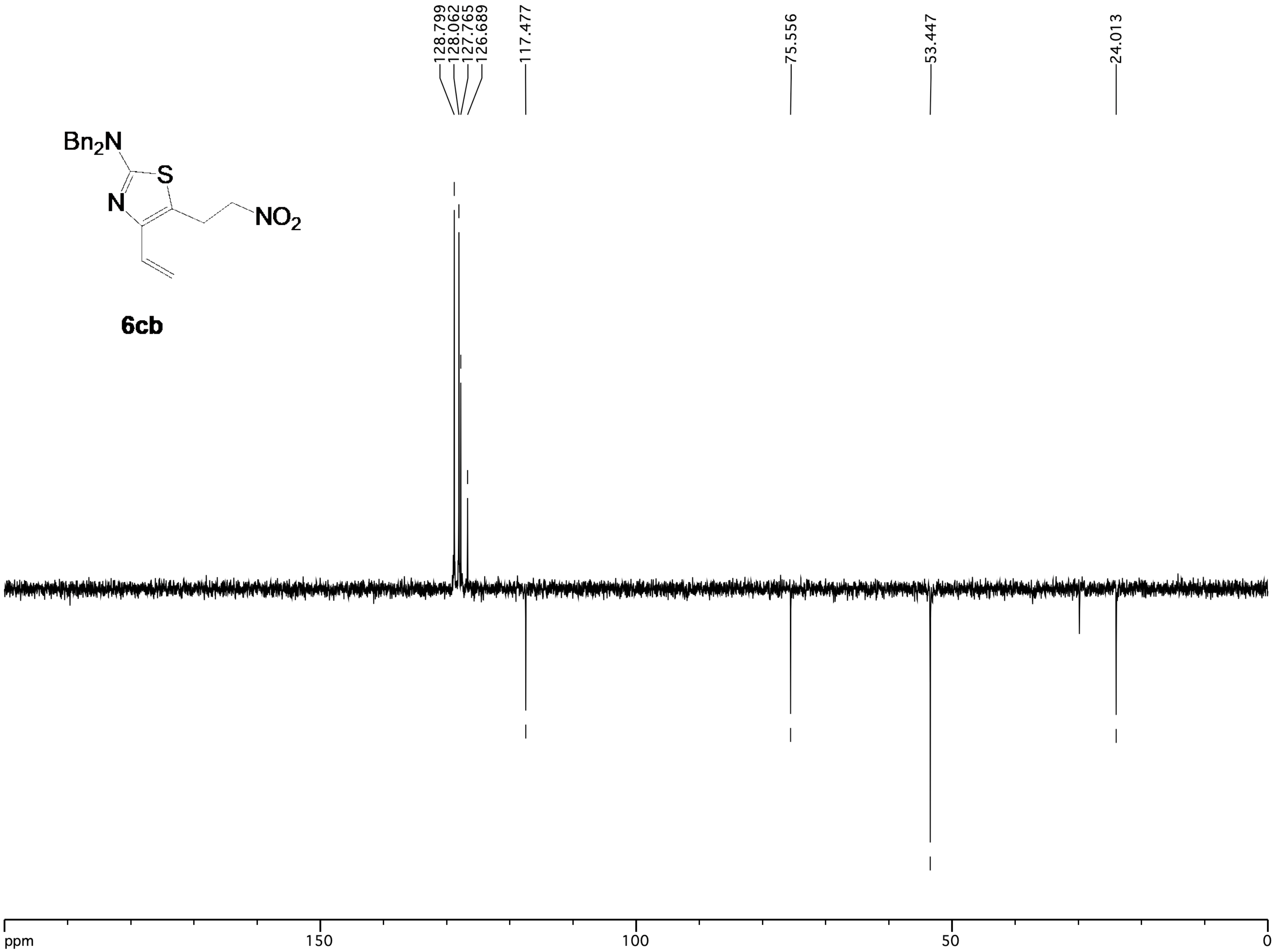

DEPT-135 ($CDCl_3$, 100 MHz) of compound **6cb**.

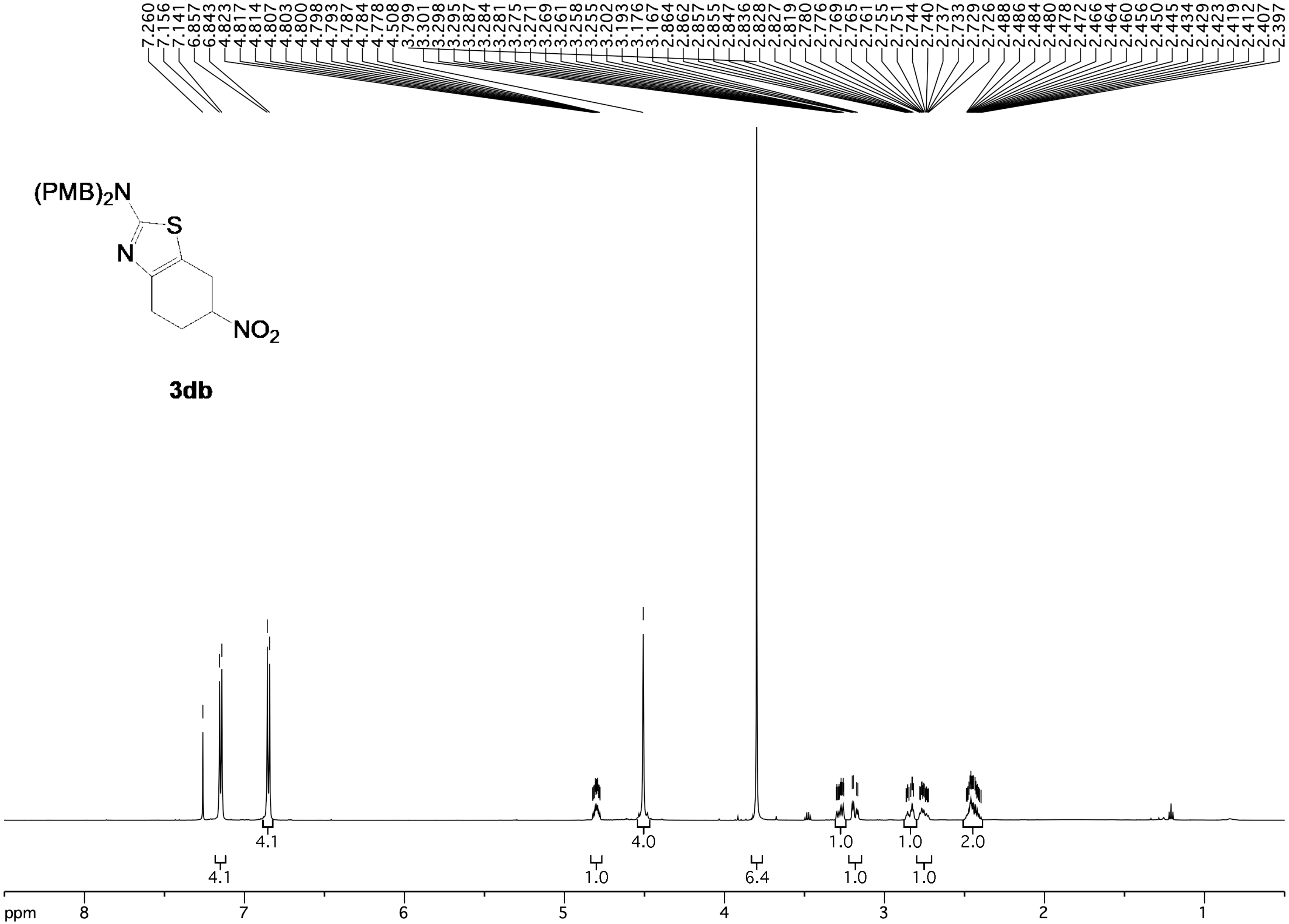

^1H NMR ($CDCl_3$, 600 MHz) of compound **3db**.

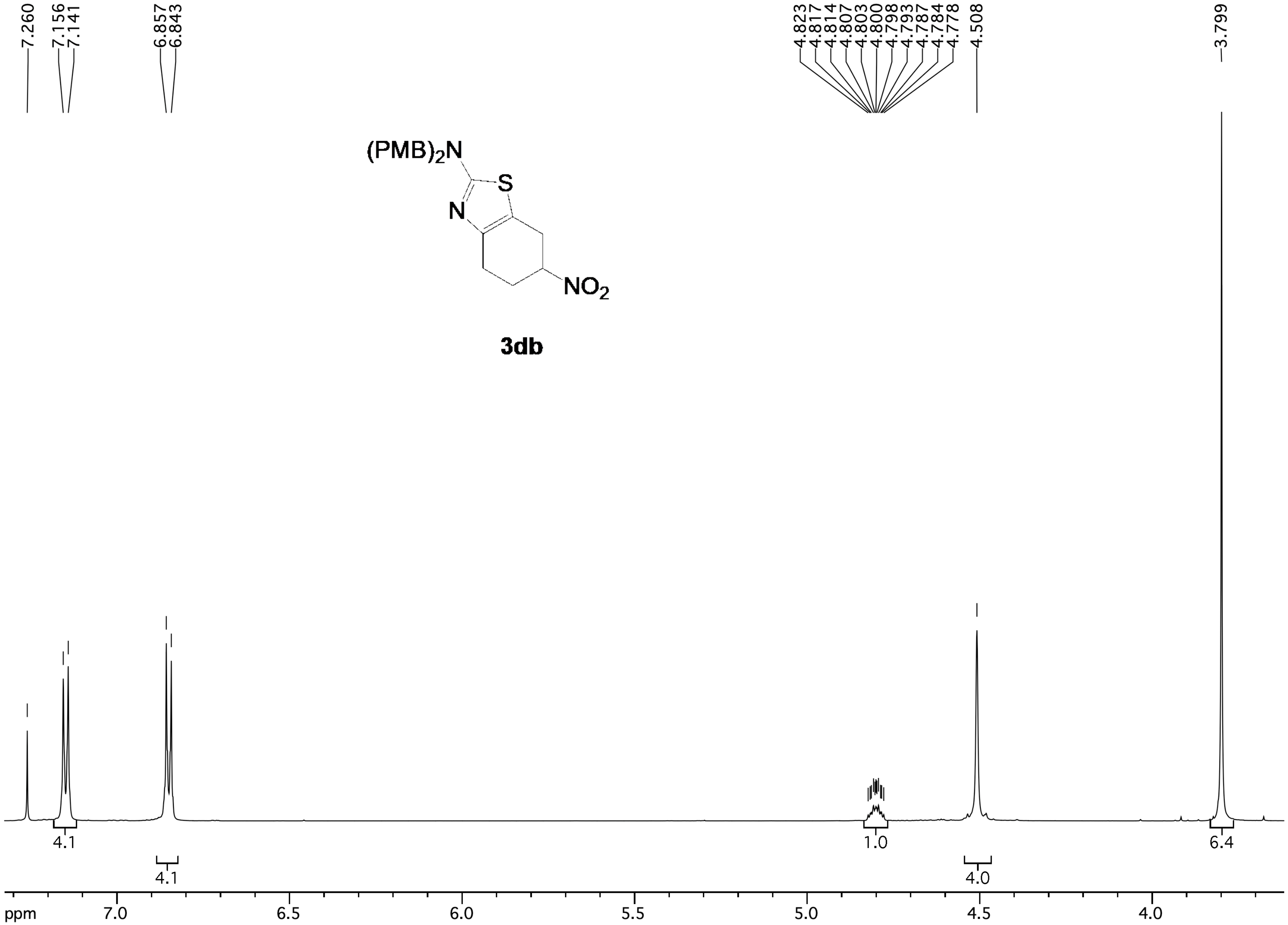

^{1}H NMR (CDCl$_3$, 600 MHz) of compound **3db** (expansion).

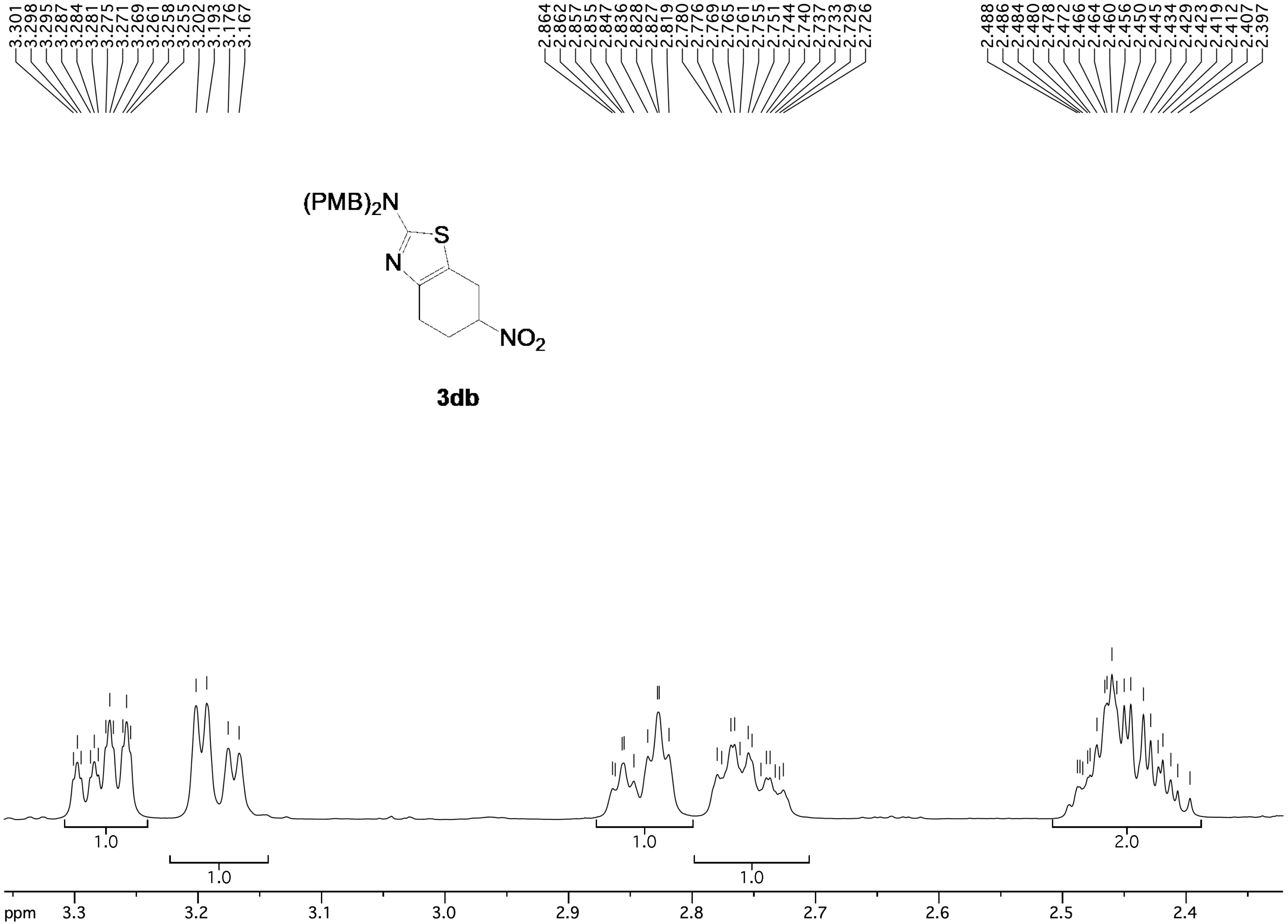

^{1}H NMR ($CDCl_3$, 600 MHz) of compound **3db** (expansion).

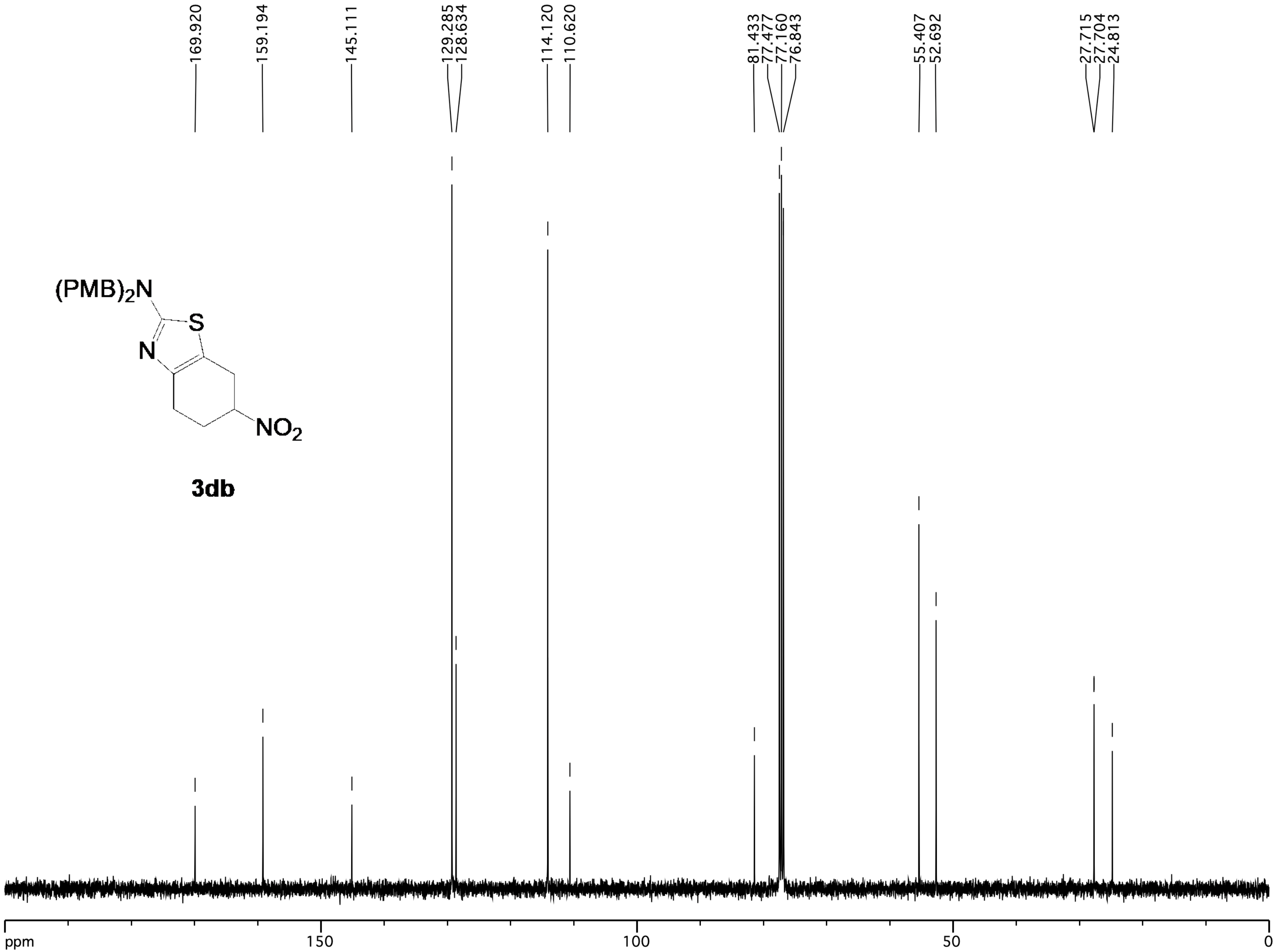

$^{13}C\{^{1}H\}$ NMR ($CDCl_3$, 100 MHz) of compound **3db**.

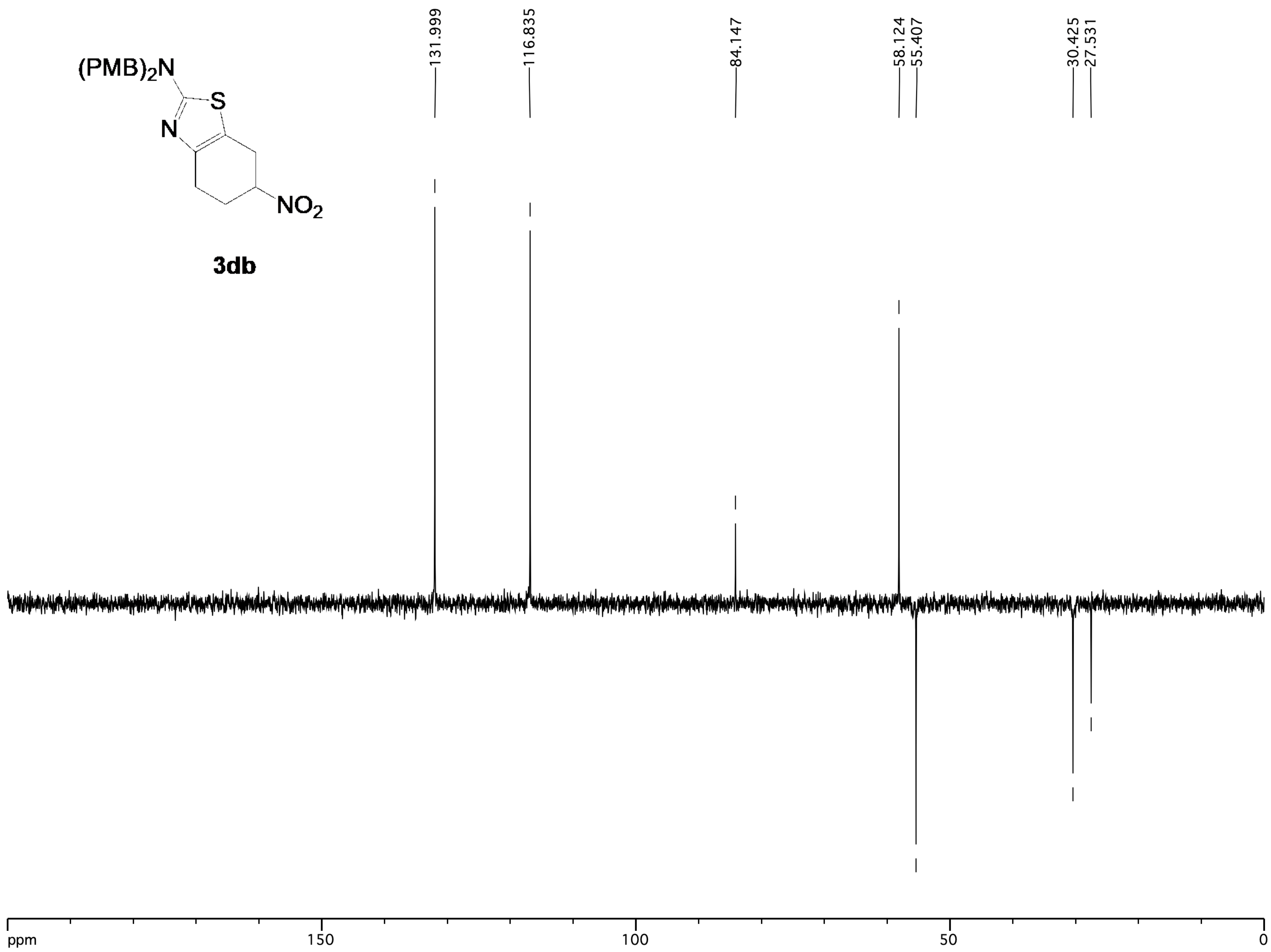

DEPT-135 ($CDCl_3$, 100 MHz) of compound **3db**.

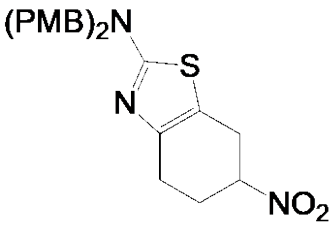

3db

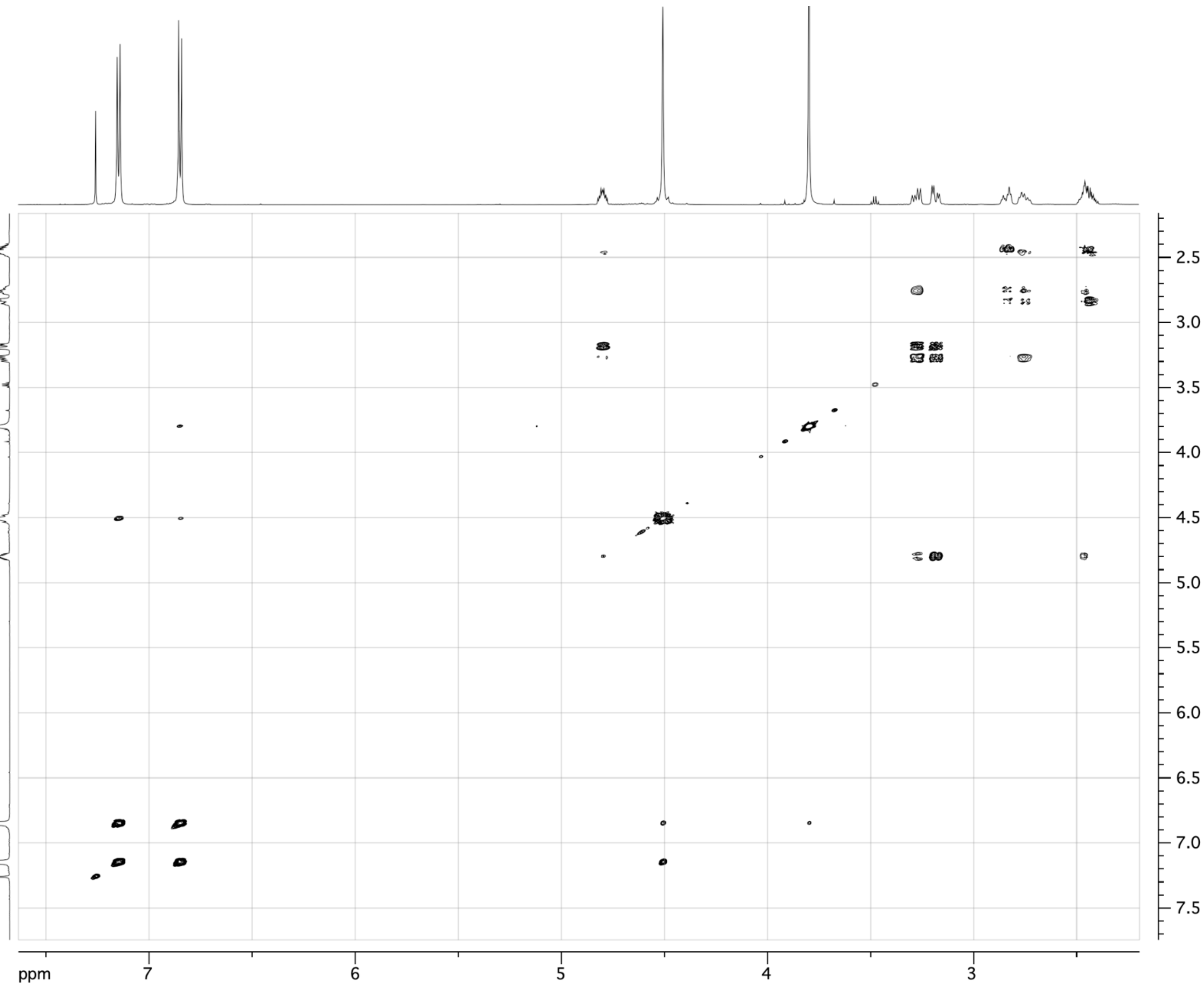

COSY ($CDCl_3$, 600 MHz) of compound **3db**.

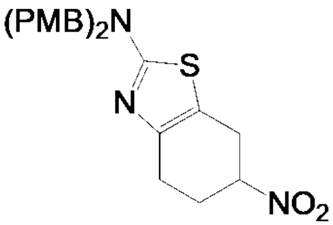

3db

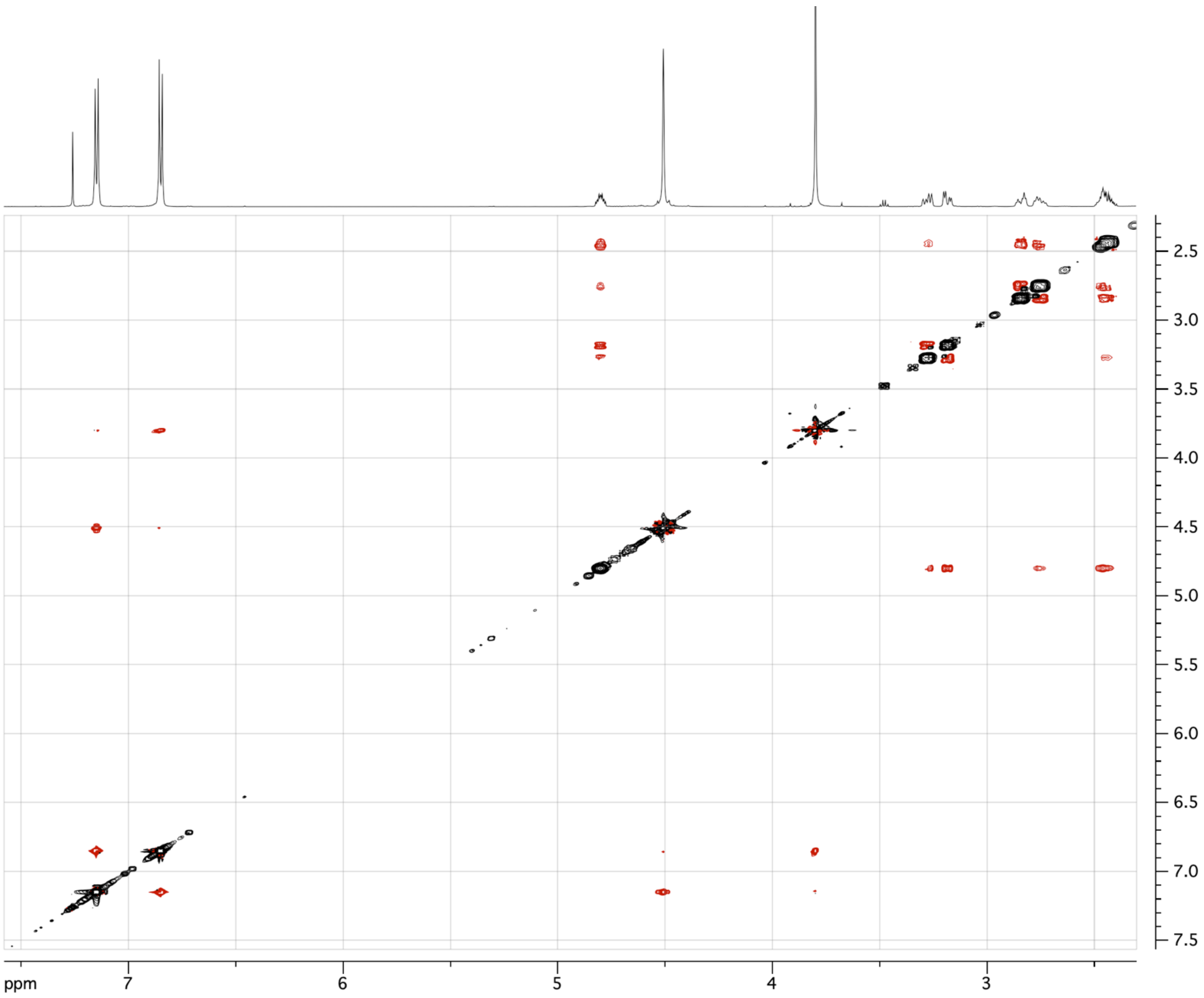

NOESY ($CDCl_3$, 600 MHz) of compound **3db**.

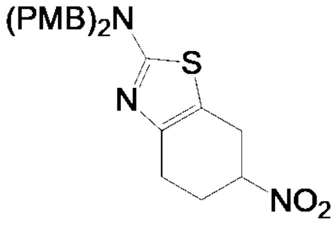

3db

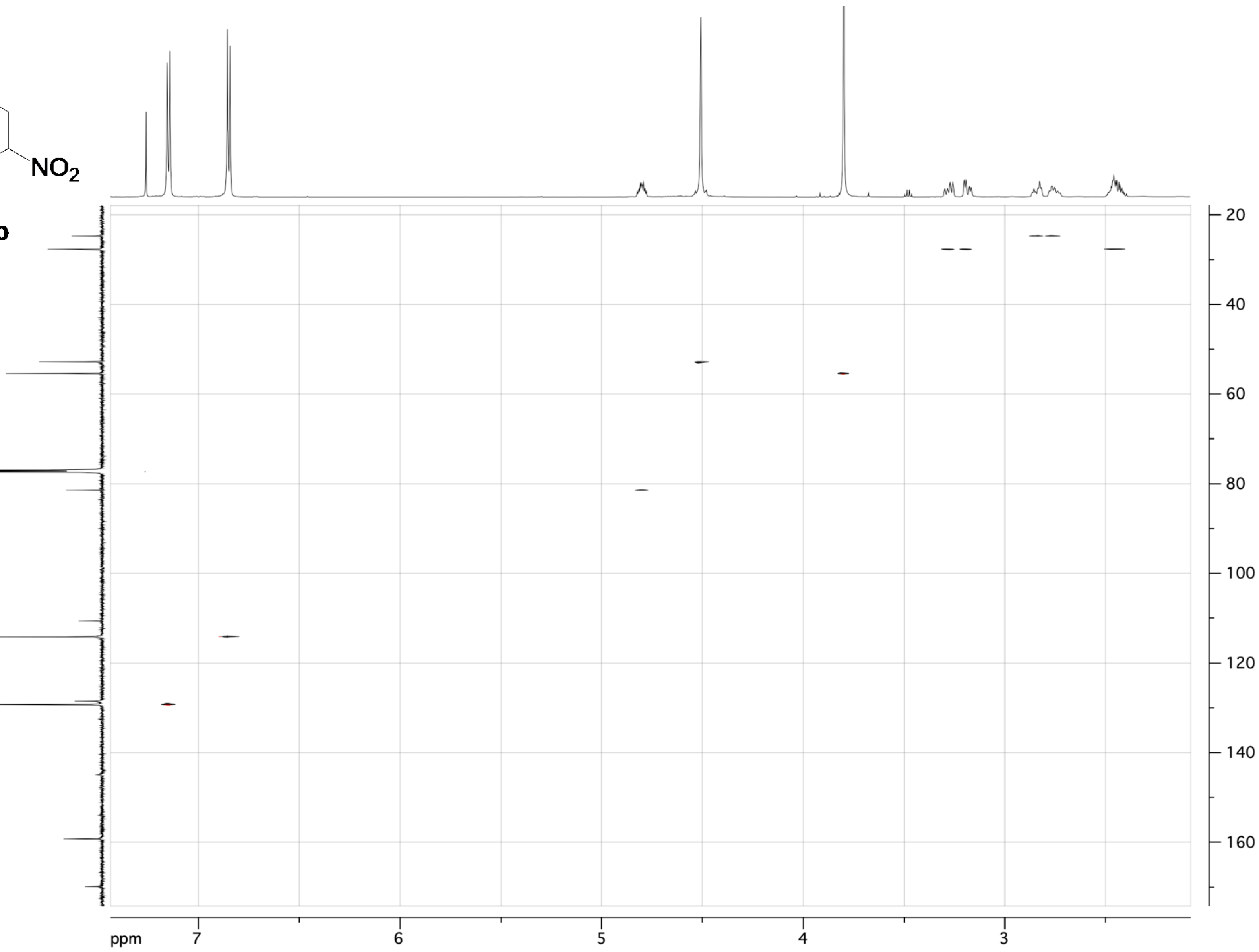

HSQC ($CDCl_3$, 600 MHz) of compound **3db**.

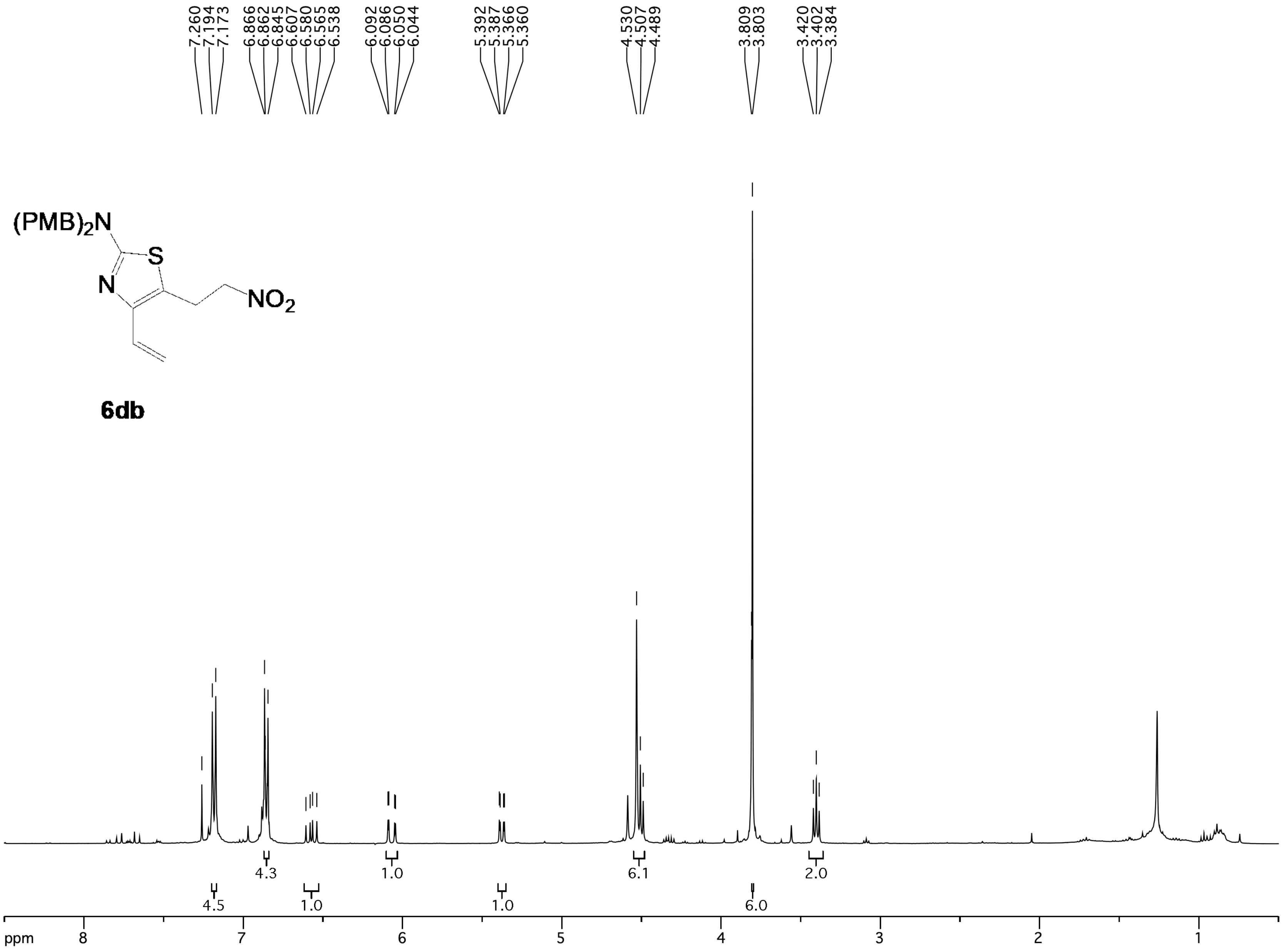

^{1}H NMR (CDCl$_3$, 400 MHz) of compound **6db**.

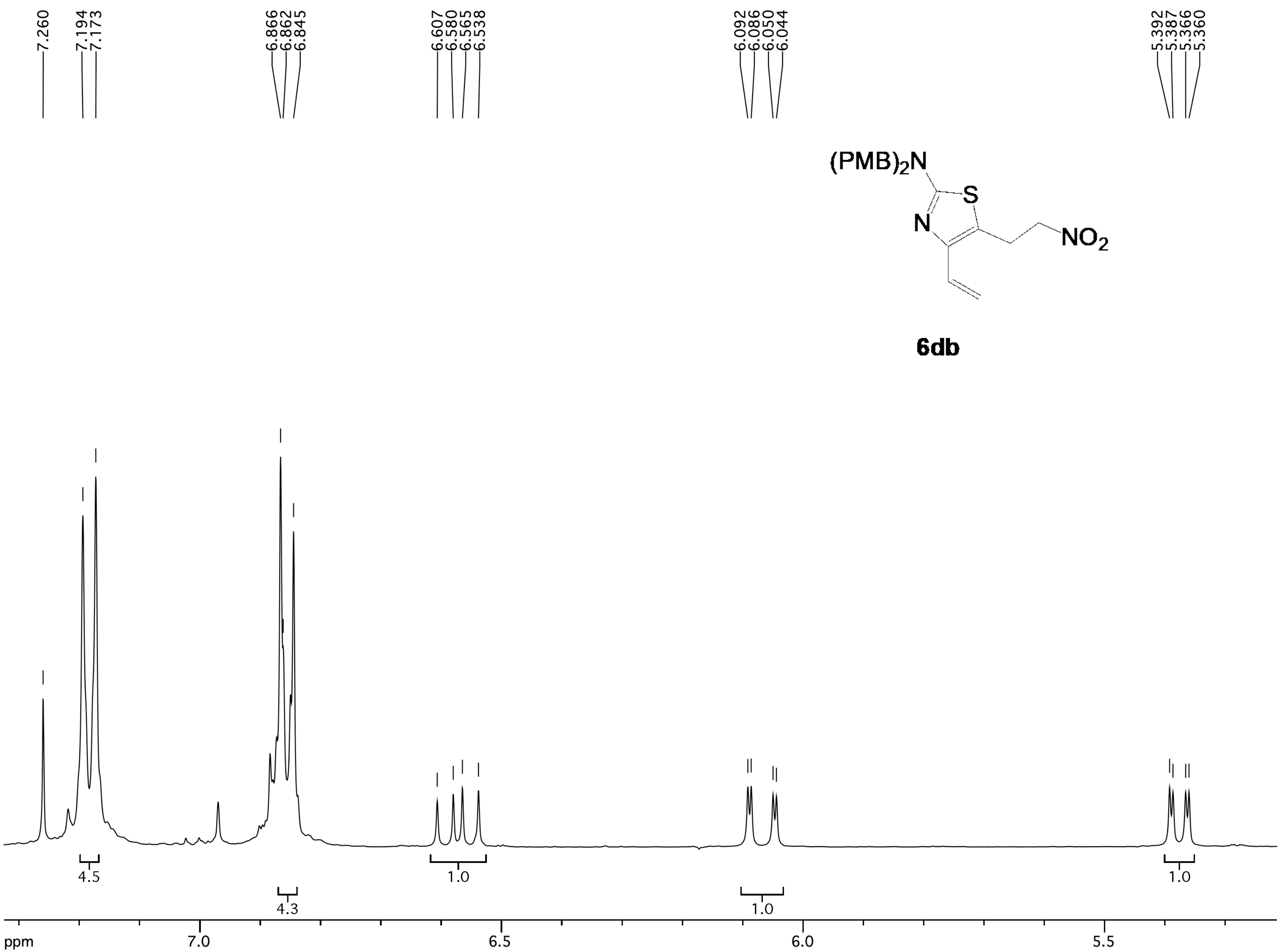

^{1}H NMR ($CDCl_3$, 400 MHz) of compound **6db** (expansion).

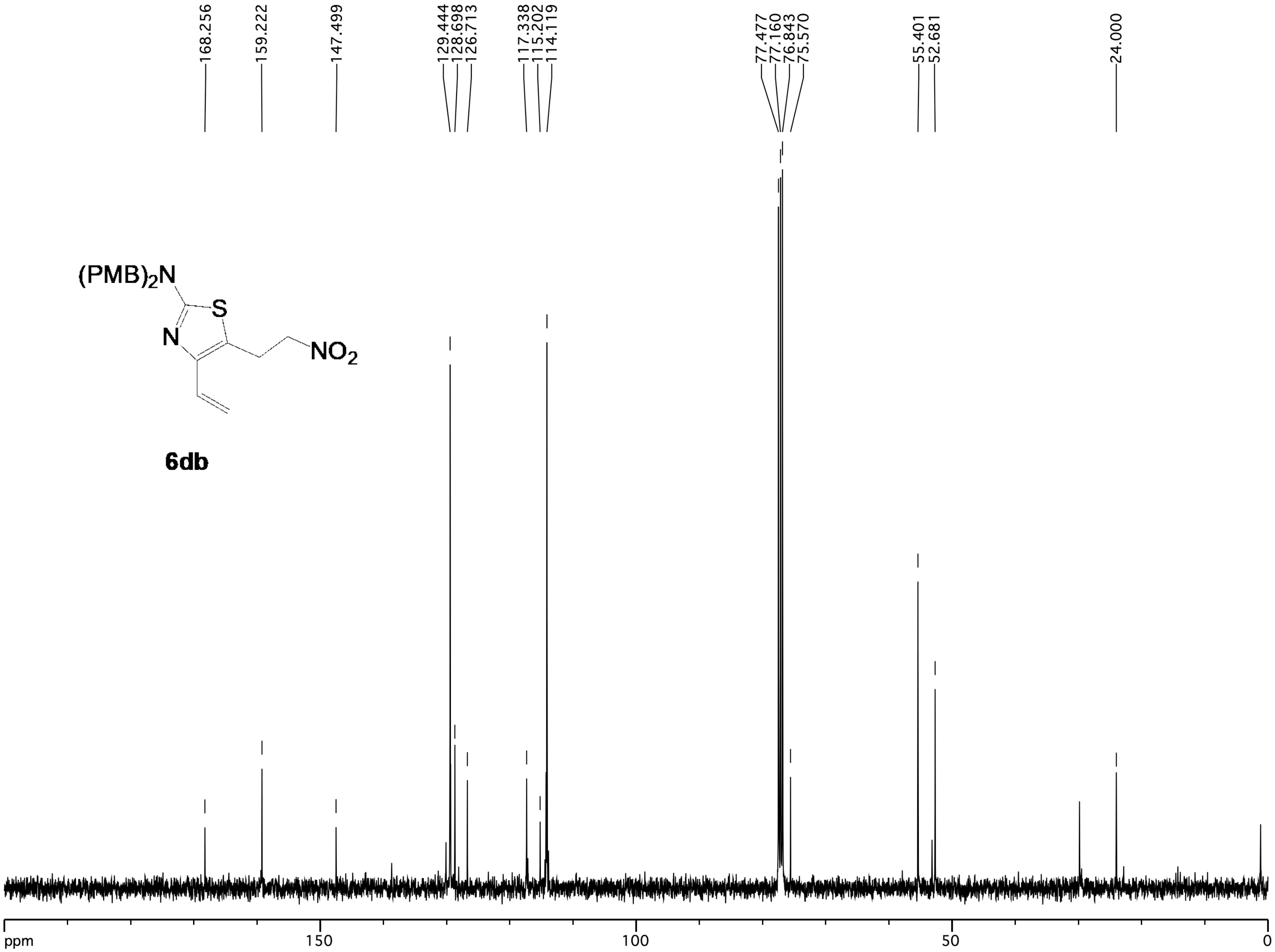

$^{13}C\{^1H\}$ NMR (CDCl₃, 100 MHz) of compound **6db**.

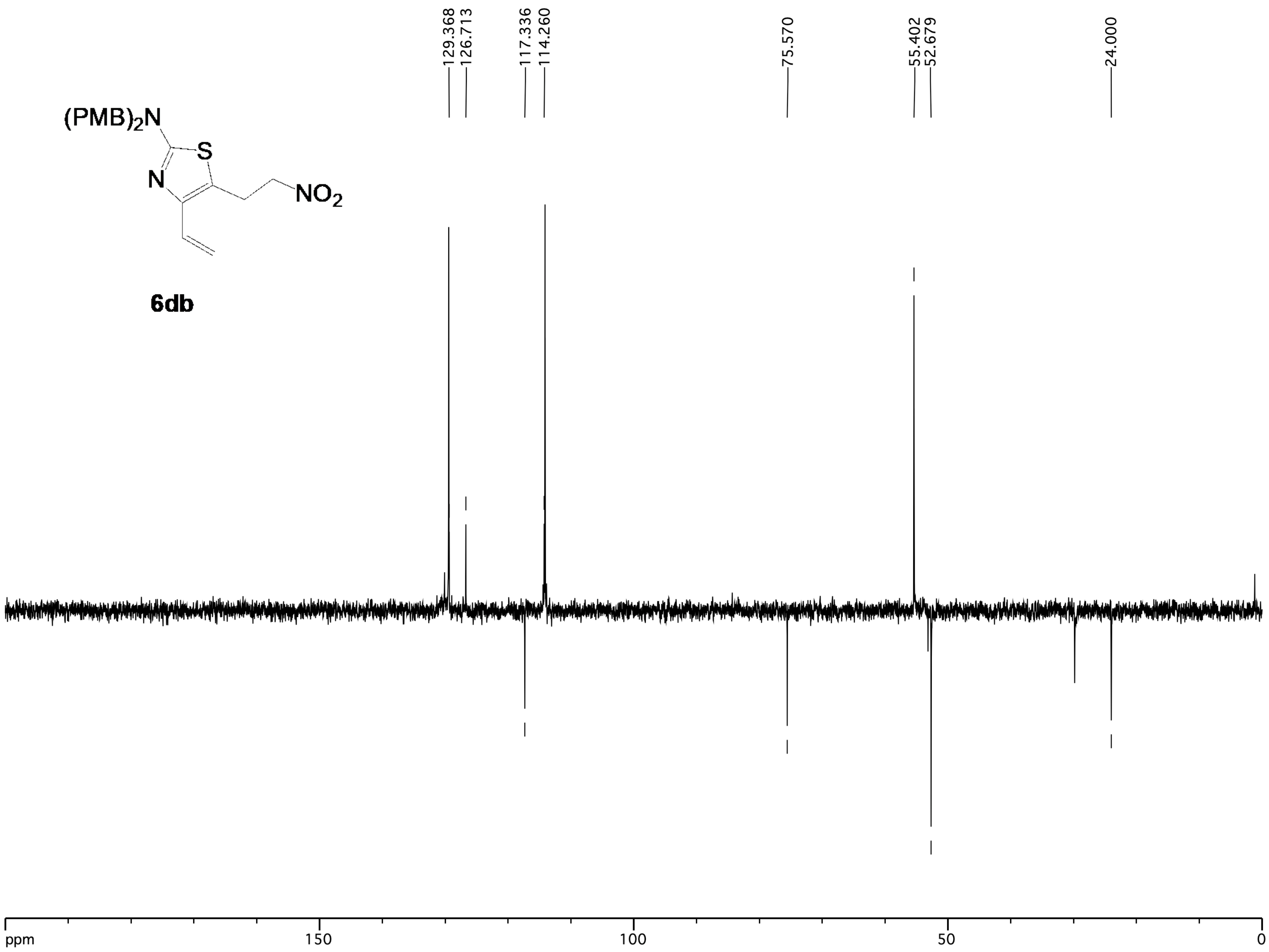

DEPT-135 ($CDCl_3$, 100 MHz) of compound **6db**.

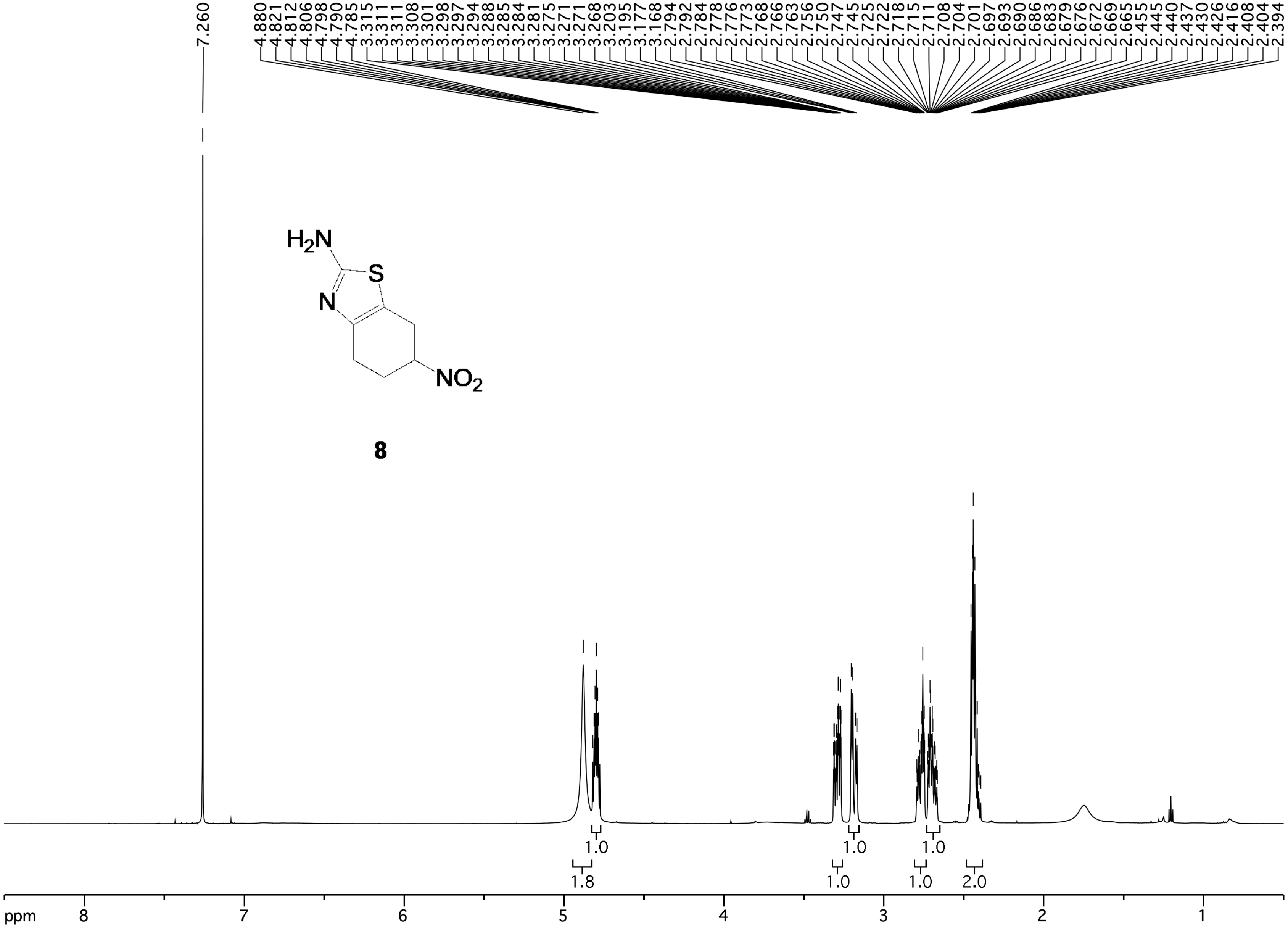

^{1}H NMR (CDCl$_3$, 600 MHz) of compound **8**.

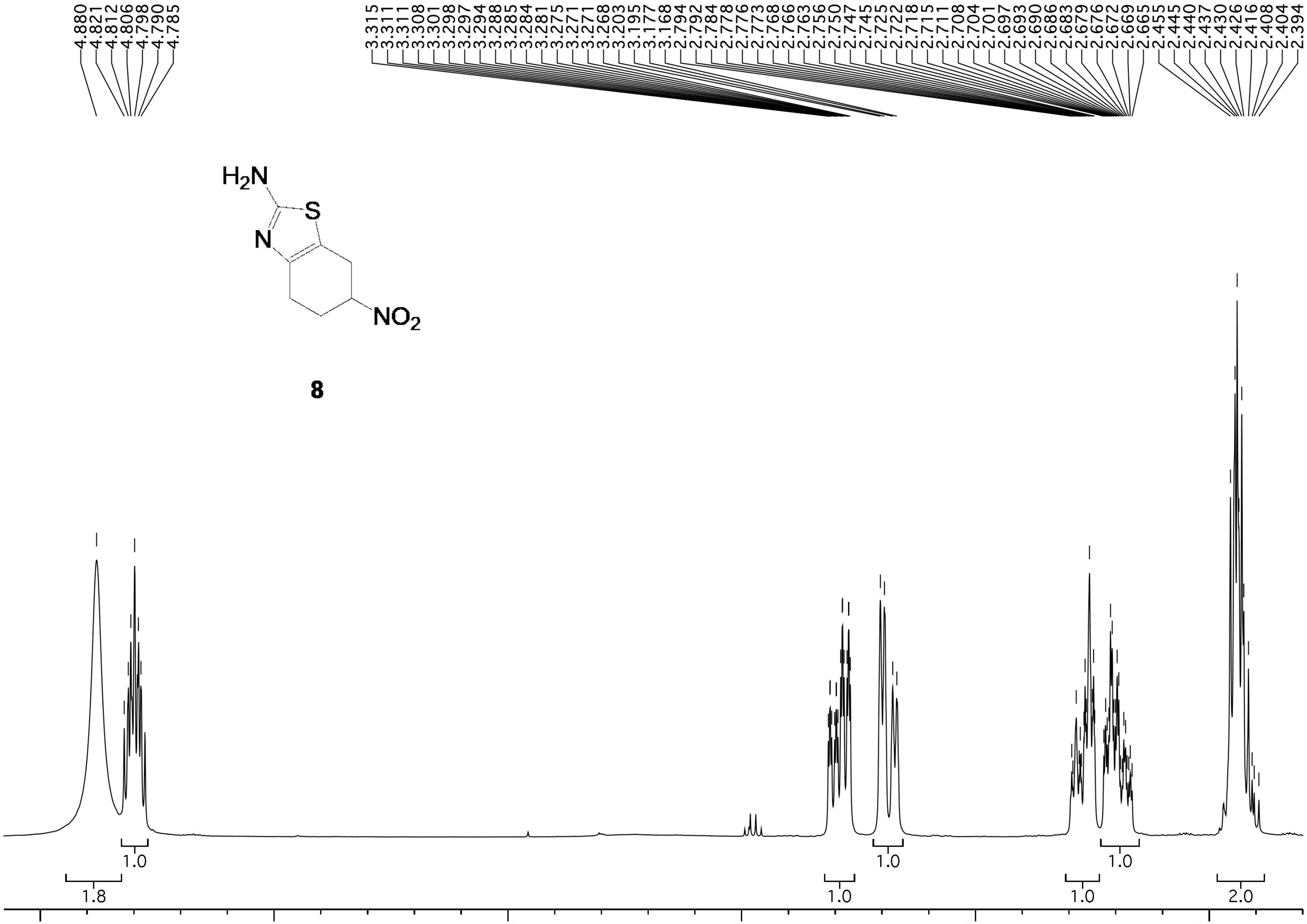

^{1}H NMR ($CDCl_3$, 600 MHz) of compound **8** (expansion).

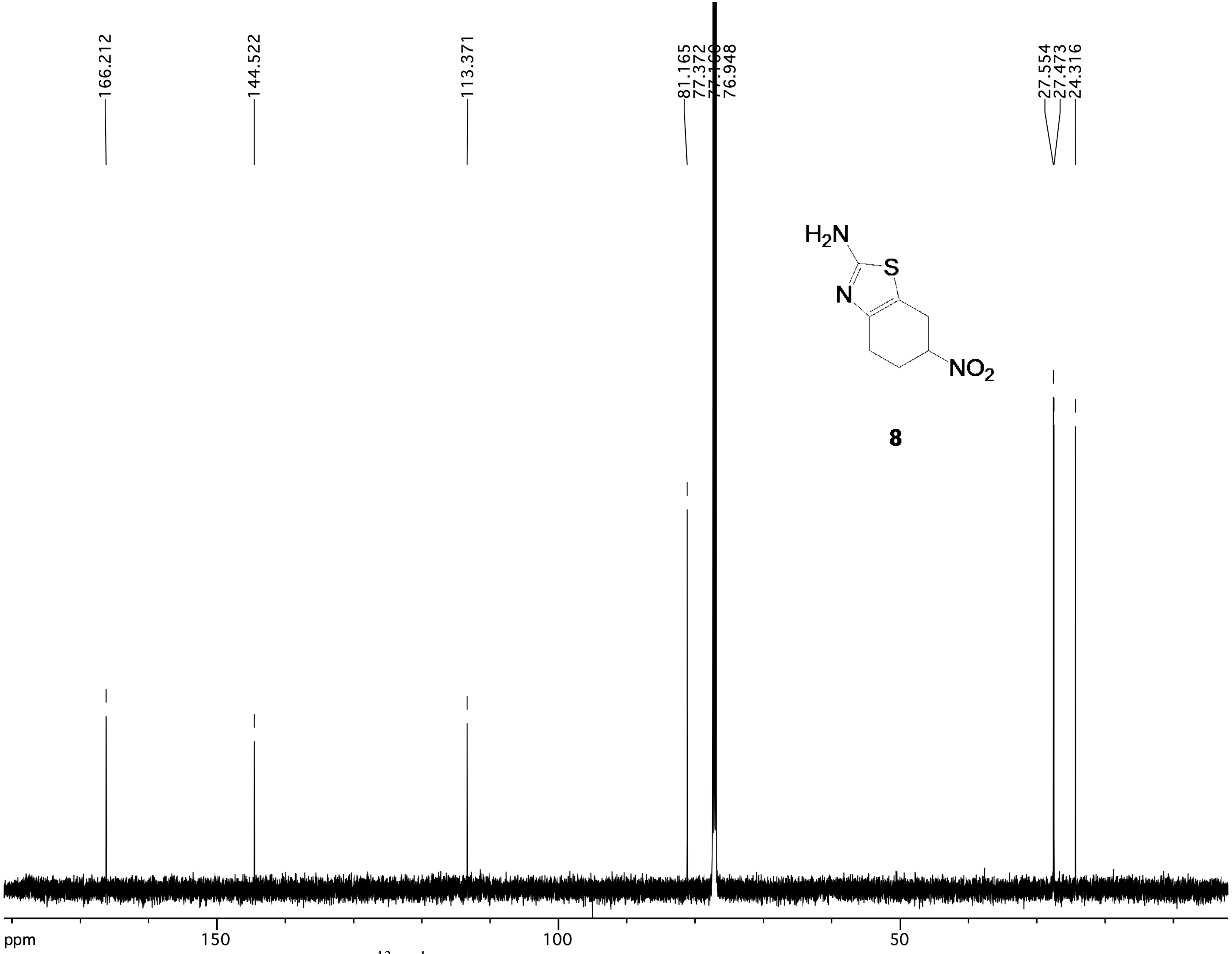

$^{13}C\{^{1}H\}$ NMR ($CDCl_3$, 150 MHz) of compound **8**.

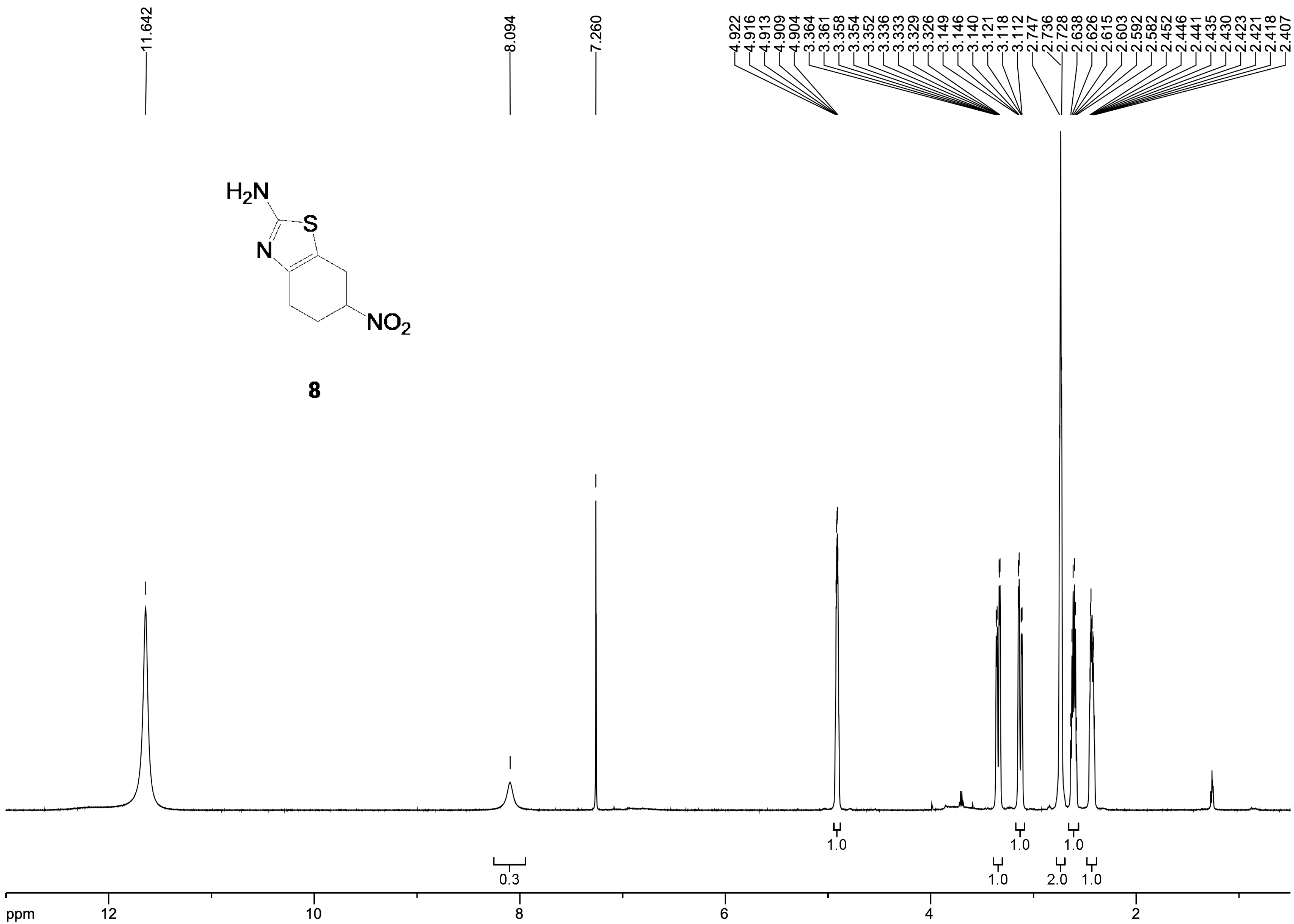

^{1}H NMR ($CDCl_3$/TFA, 600 MHz) of compound **8**.

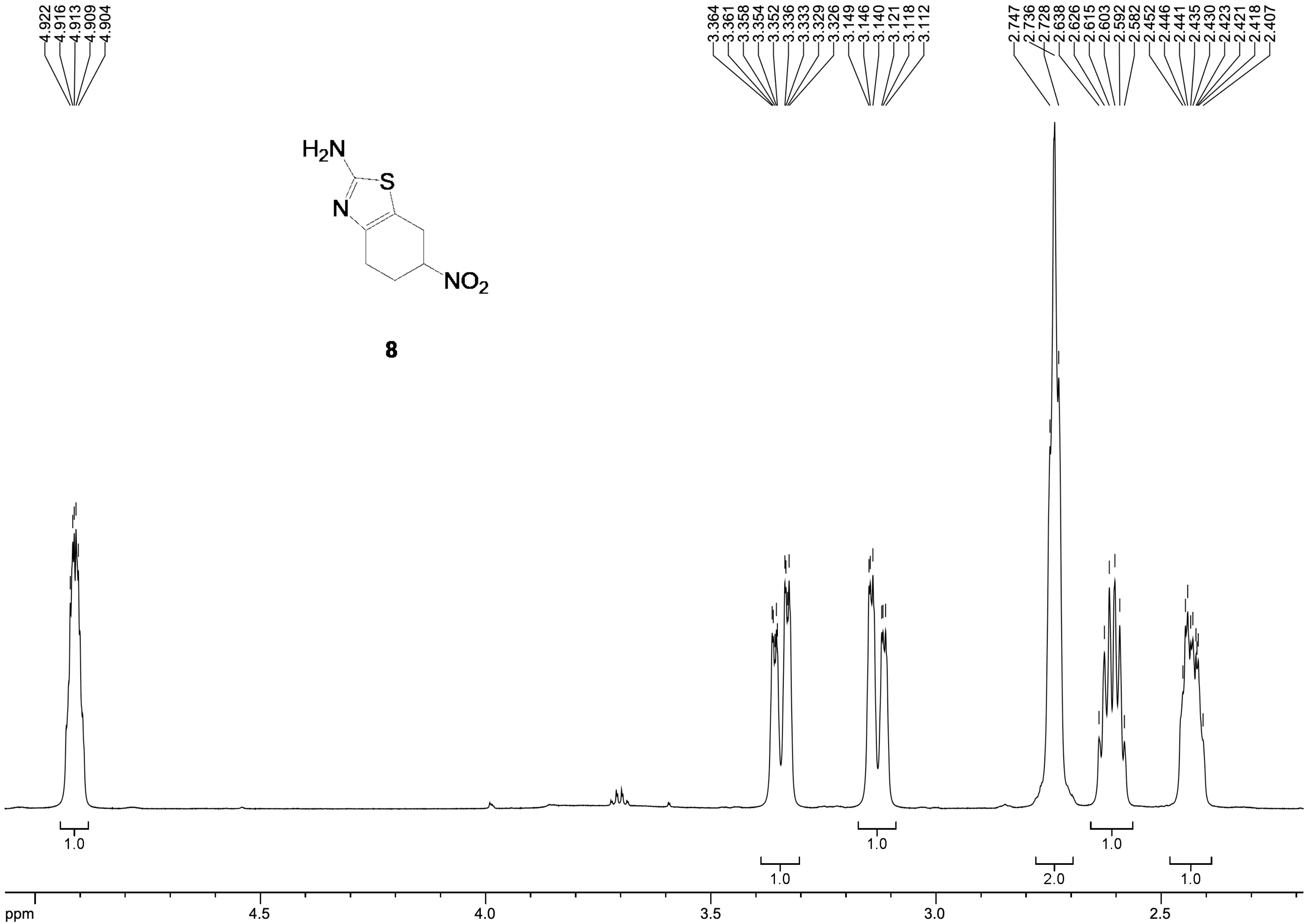

^{1}H NMR (CDCl$_3$/TFA, 600 MHz) of compound **8** (expansion)

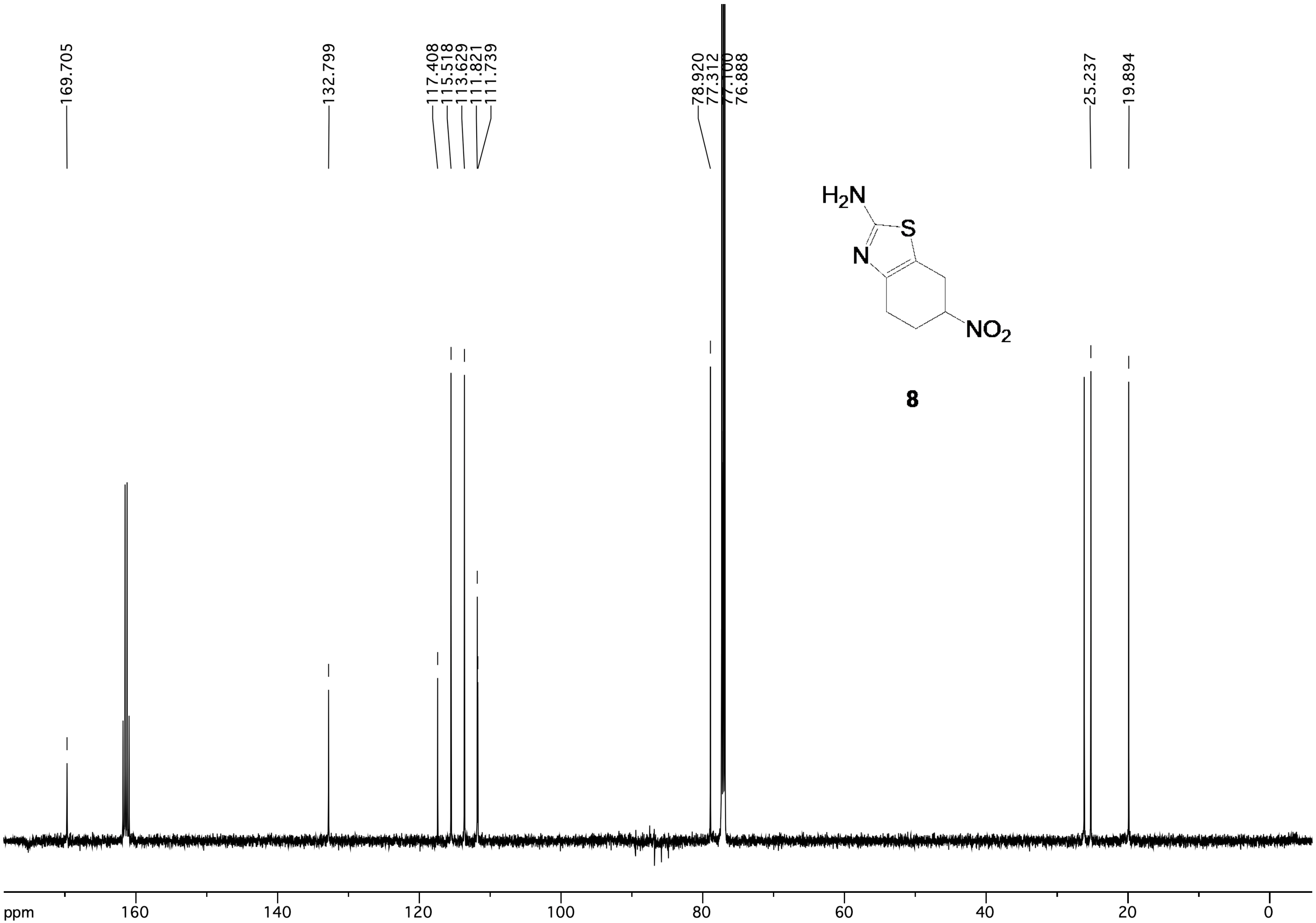

$^{13}C\{^{1}H\}$ NMR ($CDCl_3$/TFA, 150 MHz) of compound **8**.

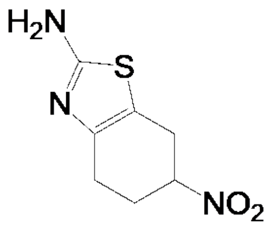

8

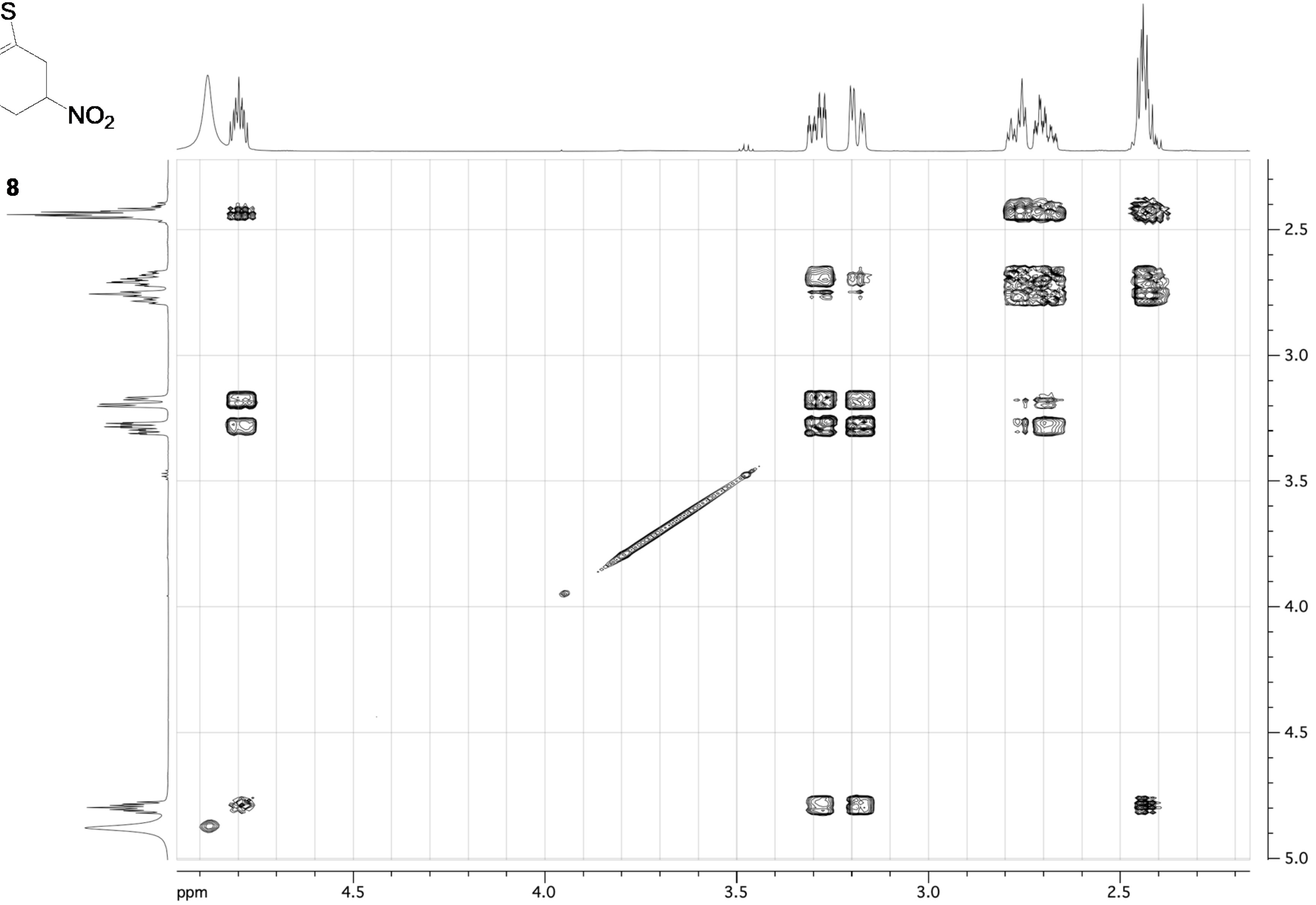

COSY ($CDCl_3$, 600 MHz) of compound **8**.

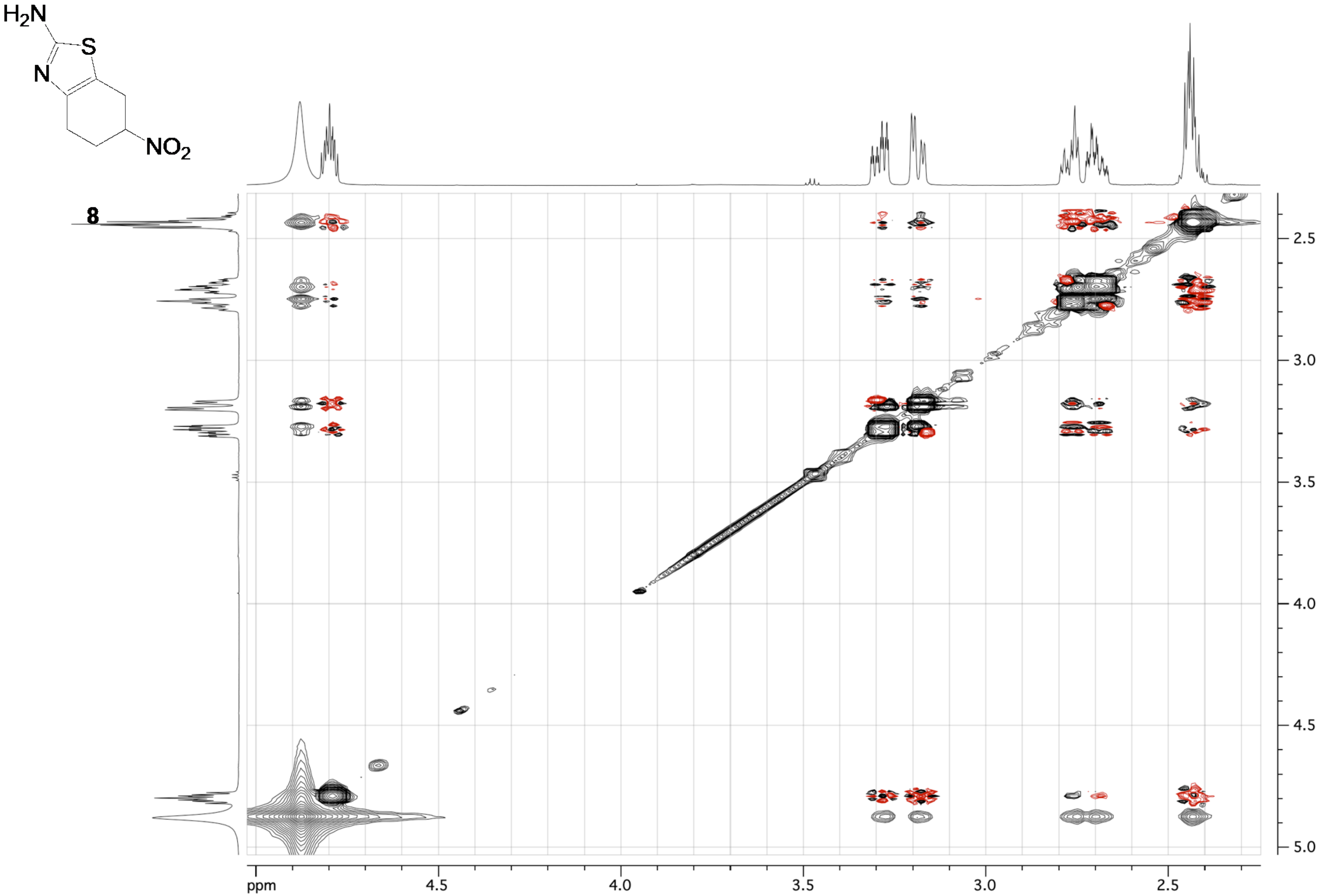

NOESY ($CDCl_3$, 600 MHz) of compound **8**.

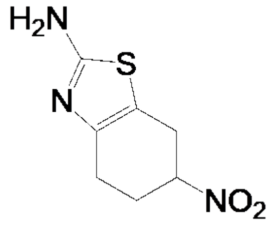

8

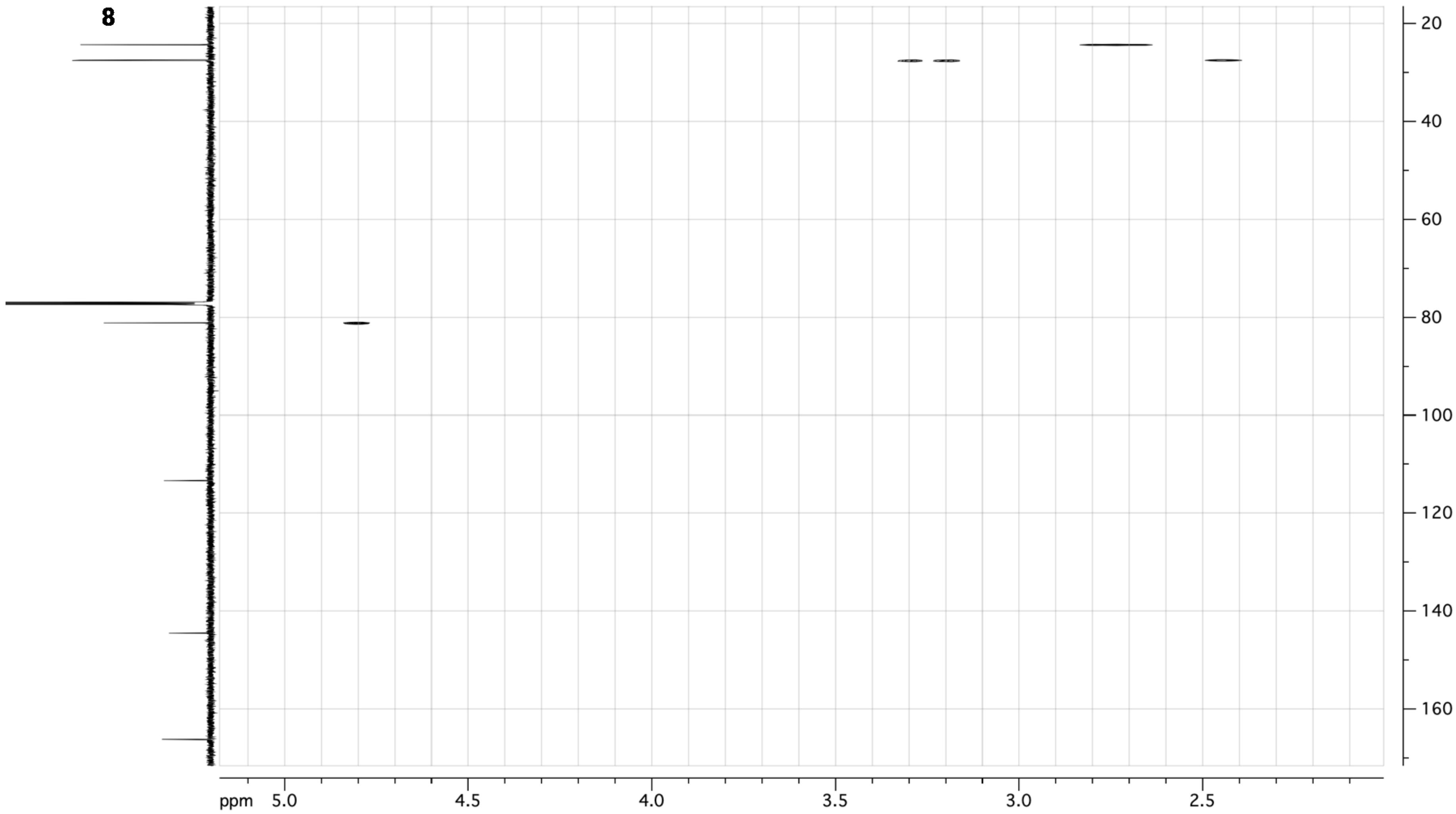

HSQC ($CDCl_3$, 600 MHz) of compound **8**.

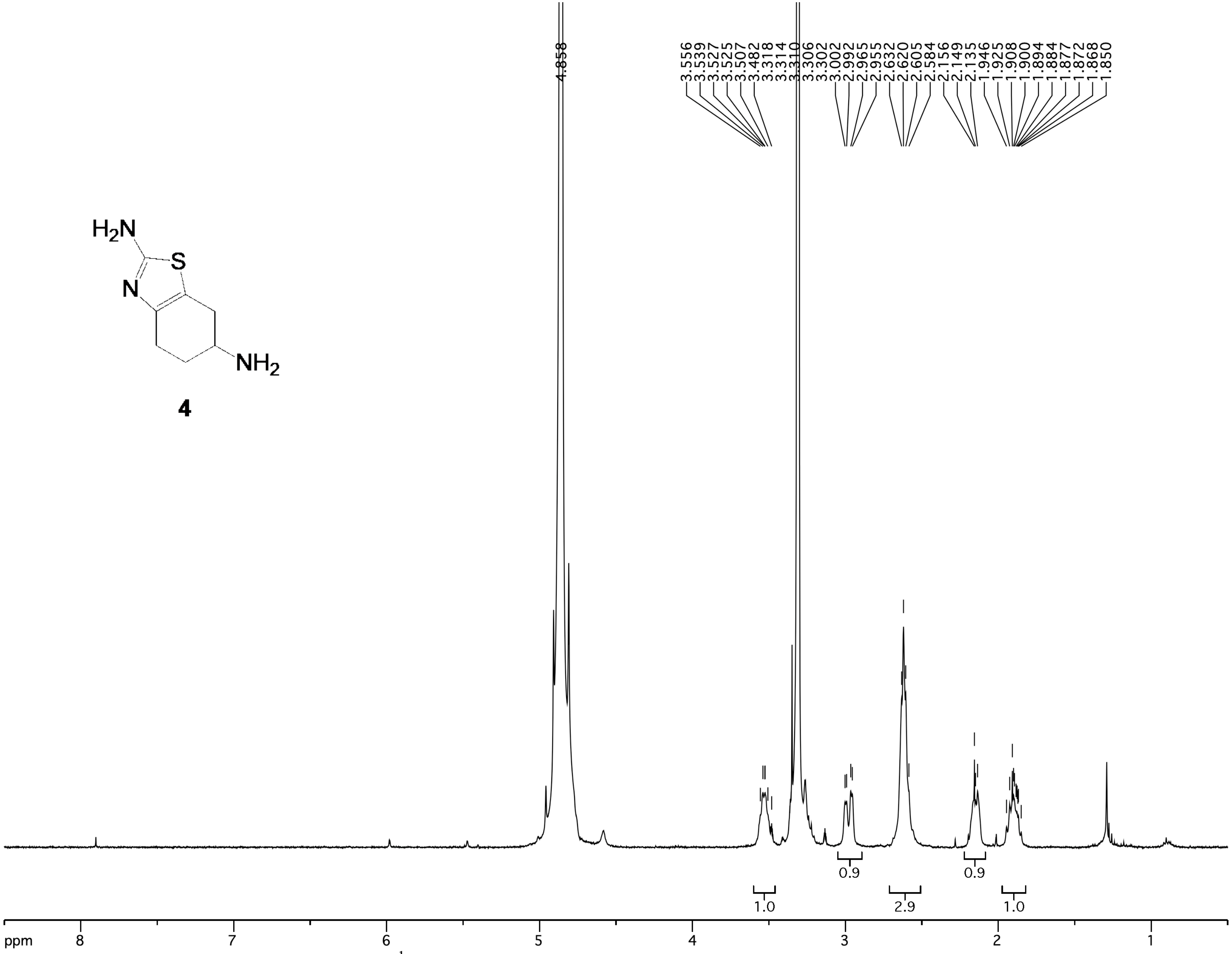

^{1}H NMR (CD_3OD, 400 MHz) of compound **4**.

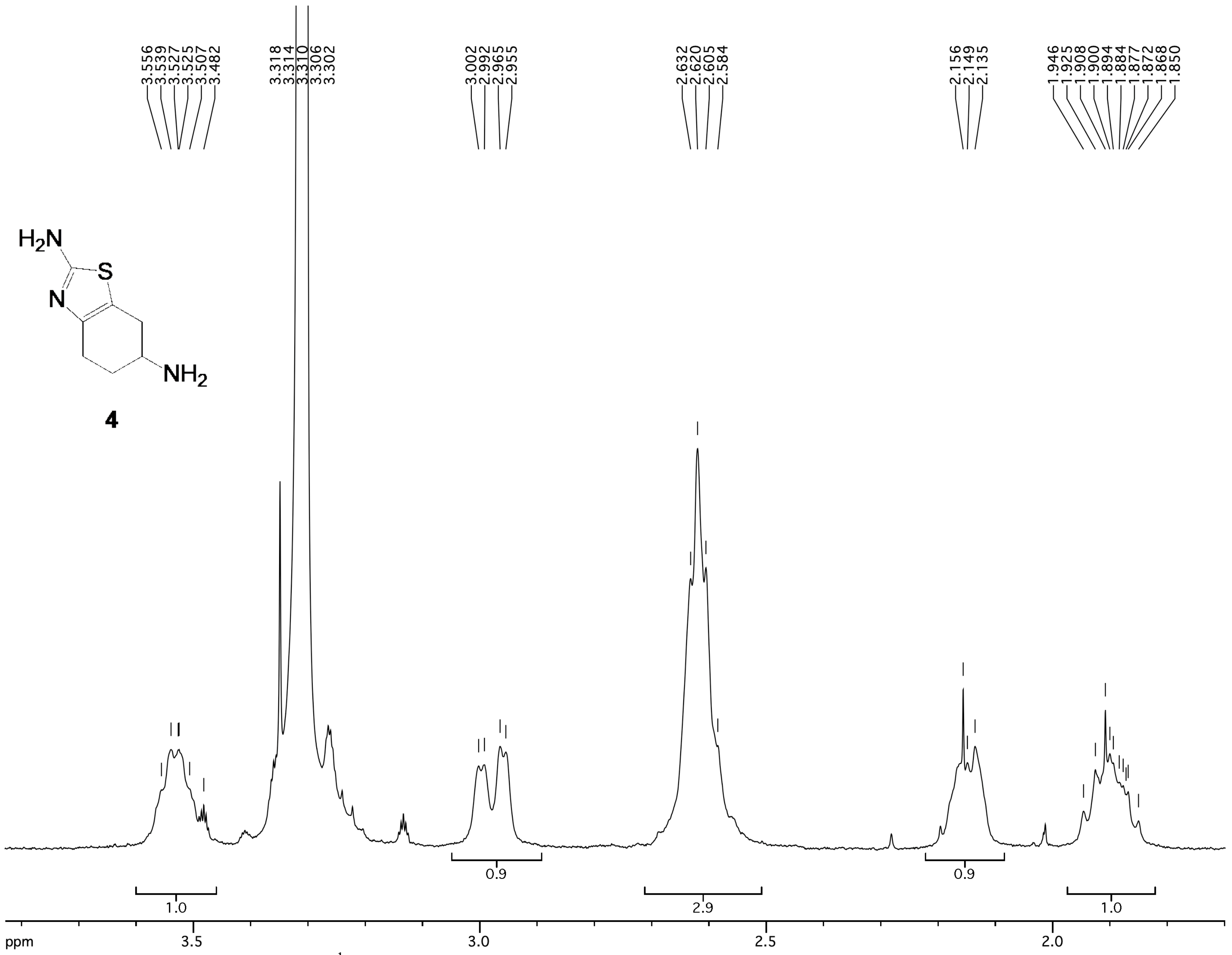

^{1}H NMR (CD_3OD, 400 MHz) of compound **4** (expansion).

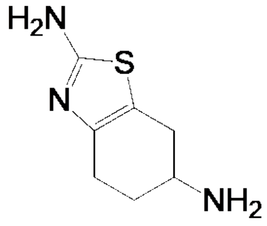

4

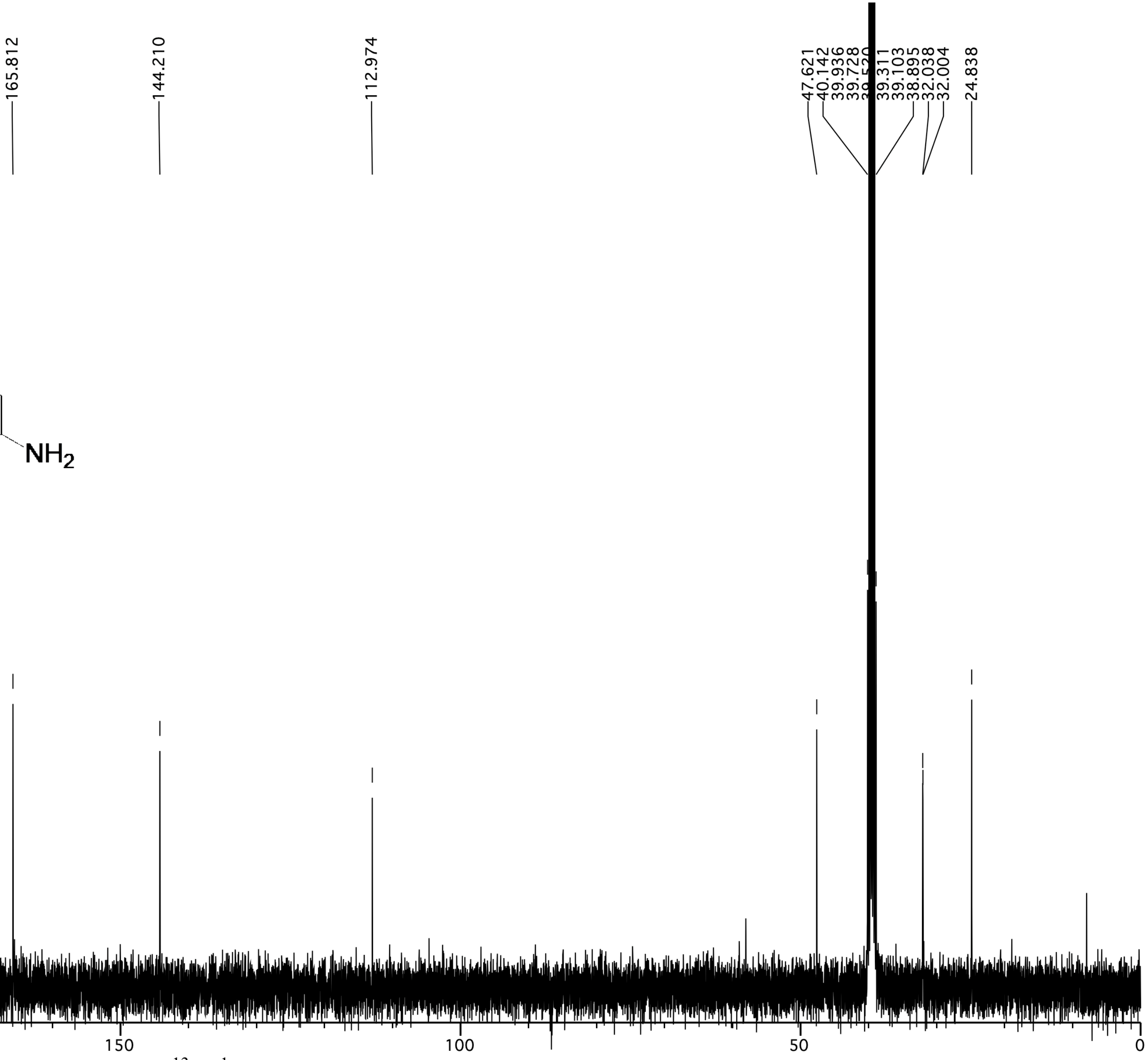

$^{13}C\{^1H\}$ NMR (DMSO-*d*6, 100 MHz) of compound **4**.

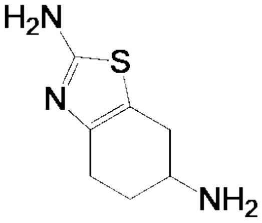

4

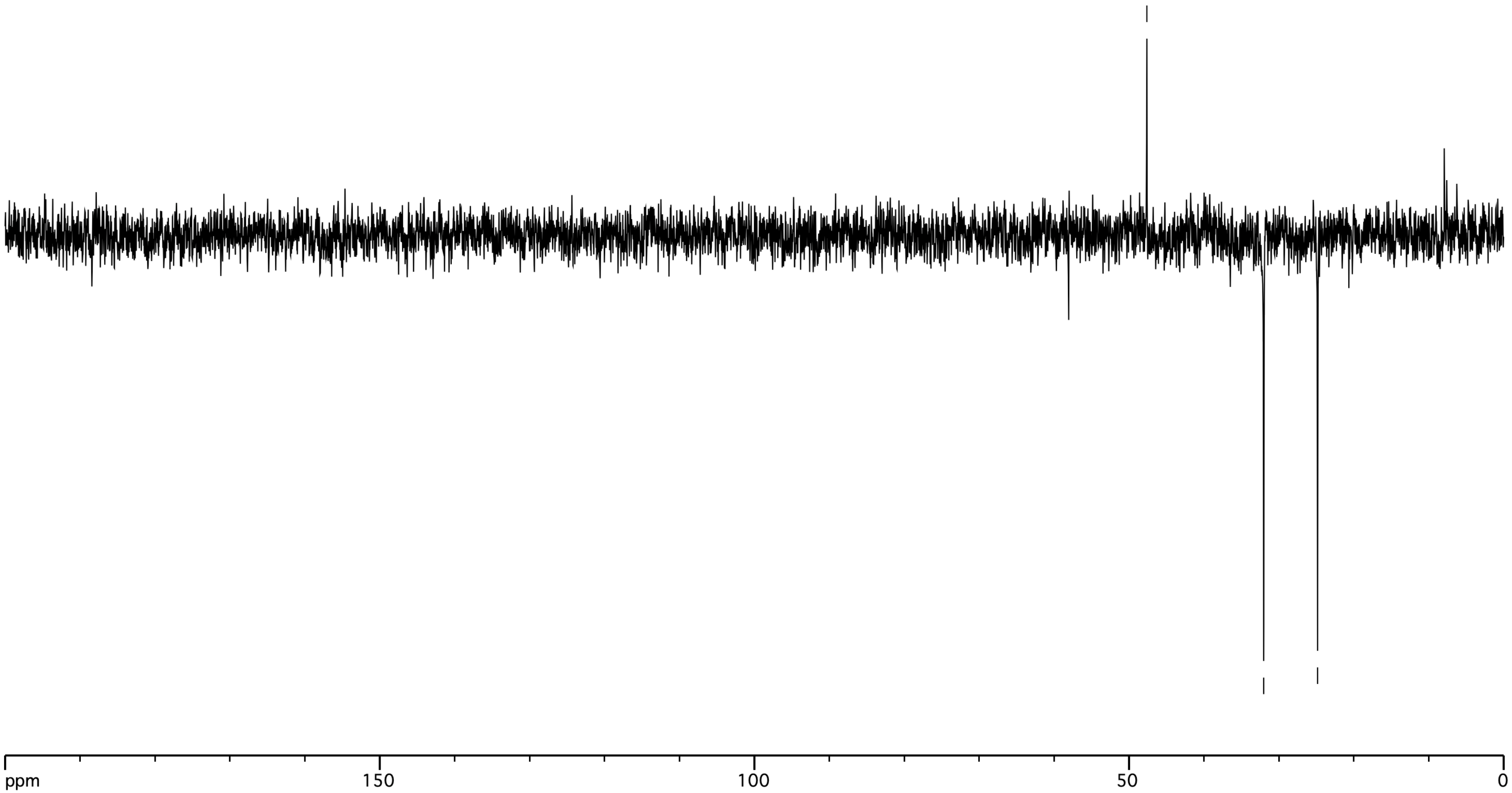

DEPT-135 (DMSO-d_6, 100 MHz) of compound **4**.

1.6. References

1. J. R. Catch, D. F. Elliott, D. H. Hey and E. R. H. Jones, Halogenated Ketones. Part IV. The Application of the Friedel-Crafts Reaction to the Preparation of Halogenated Aliphatic Ketones., *J. Chem. Soc.*, 1948, 278-281.
2. M. Alajarin, J. Cabrera, A. Pastor, P. Sanchez-Andrada and D. Bautista, On the [2+2] Cycloaddition of 2-Aminothiazoles and Dimethyl Acetylenedicarboxylate. Experimental and Computational Evidence of a Thermal Disrotatory Ring Opening of Fused Cyclobutenes, *J. Org. Chem.*, 2006, **71**, 5328-5339.
3. D. Ranganathan, C. B. Rao, S. Ranganathan, A. K. Mehrotra and R. Iyengar, Nitroethylene: a Stable, Clean, and Reactive Agent for Organic Synthesis, *J. Org. Chem.*, 1980, **45**, 1185-1189.
4. H. Hartmann and I. Reuther, Darstellung and Charakterisierung 1,1-disubstituierter Thioharnstoffe, *J. Prakt. Chem. /Chem-Ztg* 1973, **315**, 144-148.
5. A. R. Katritzky, N. Kirichenko, B. V. Rogovoy, J. Kister and H. Tao, Synthesis of Mono- and *N,N*-Disubstituted Thioureas and *N*-Acylthioureas, *Synthesis*, 2004, 1799-1805.
6. R. H. Carroll and G. B. L. Smith, *α',β*-Dichloromethylethyl Ketone (1,4-Dichlorobutanone-2), *J. Am. Chem. Soc.*, 1933, **55**, 370-373.
7. P. Ferraboschi, S. Ciceri, P. Ciuffreda, M. De Mieri, D. Romano and P. Grisenti, Baker's Yeast Catalyzed Preparation of a New Enantiomerically Pure Synthon of (S)-Pramipexole and its Enantiomer (Dexpramipexole), *Tetrahedron: Asymmetry*, 2014, **25**, 1239-1245.
8. G. Abbiati, V. Canevari, D. Facoetti and E. Rossi, Diels–Alder Reactions of 2-Vinylindoles with Open-Chain C=C Dienophiles, *Eur. J. Org. Chem.*, 2007, 517-525.
9. M. Alajarin, J. Cabrera, P. Sanchez-Andrada, R.-A. Orenes and A. Pastor, 4-Alkenyl-2-aminothiazoles: Smart Dienes for Polar [4 + 2] Cycloadditions, *Eur. J. Org. Chem.*, 2013, 474-489.
10. The geometry optimization was carried out with Spartan'16 (EDF2/6-31G*).

2. Computational Part

2.1. Computational Methods

Geometries of the molecules were fully optimized by using the ωB97X-D[1] hybrid functional, which includes long range exchange correction and the semiclassical London-dispersion correction, with the 6-31G+(d,p) basis sets.[2,3] Solvent effects were taken into account with the PCM continuum solvation model[4,5] for toluene in the framework of the self-consistent reaction field (SCRF).[6,7,8] The nature of minimum and transition structures of all found stationary points on the potential energy surface was confirmed by frequency analysis at the same level of theory. The intrinsic reaction coordinate (IRC) paths[9] were traced to check the energy profiles connecting each TS to the two associated minima.[10,11] Thermochemical corrections at 298.15 K and 1 atm were calculated for all stationary points from unscaled vibrational frequencies.[12] All calculations were performed by using

[1] Chai, J.-D.; Head-Gordon, M. Long-Range Corrected Hybrid Density Functionals with Damped Atom–Atom Dispersion Corrections. *Phys. Chem. Chem. Phys.* **2008**, *10* (44), 6615. https://doi.org/10.1039/b810189b.

[2] Ditchfield, R.; Hehre, W. J.; Pople, J. A. Self-Consistent Molecular-Orbital Methods. IX. An Extended Gaussian-Type Basis for Molecular-Orbital Studies of Organic Molecules. *J. Chem. Phys.* **1971**, *54* (2), 724–728. https://doi.org/10.1063/1.1674902.

[3] Clark, T.; Chandrasekhar, J.; Spitznagel, G. W.; Schleyer, P. V. R. Efficient Diffuse Function-Augmented Basis Sets for Anion Calculations. III. The 3-21+G Basis Set for First-Row Elements, Li–F. *J. Comput. Chem.* **1983**, *4* (3), 294–301. https://doi.org/10.1002/jcc.540040303..

[4] Tomasi, J.; Persico, M. Molecular Interactions in Solution: An Overview of Methods Based on Continuous Distributions of the Solvent. *Chem. Rev.* **1994**, *94* (7), 2027–2094. https://doi.org/10.1021/cr00031a013.

[5] Simkin, Y. B; Sheikhet, I. I. Quantum chemical and statistical theory of solutions – computational approach, Ellis Horwood, London, **1995**.

[6] Cossi, M.; Barone, V.; Cammi, R.; Tomasi, J. Ab initio study of solvated molecules: a new implementation of the polarizable continuum model. *Chem. Phys. Lett.*, **1996**, *255*, 327–335. https://doi.org/10.1016/0009-2614(96)00349-1

[7] Cances, E.; Mennucci, B.; Tomasi, J. A new integral equation formalism for the polarizable continuum model: Theoretical background and applications to isotropic and anisotropic dielectrics. *J. Chem. Phys.*, **1997**, *107*, 3032–3041. https://doi.org/10.1063/1.474659

[8] Barone, V.; M. Cossi, M.; Tomasi, J. Geometry optimization of molecular structures in solution by the polarizable continuum model. *J. Comput. Chem.*, **1998**, *19* (4), 404–417. https://doi.org/10.1002/(SICI)1096-987X(199803)19:4%3C404::AID-JCC3%3E3.0.CO;2-W

[9] Fukui, K. Formulation of the reaction coordinate. *J. Phys. Chem.*, **1970**, *74* (23), 4161–4163 https://doi.org/10.1021/j100717a029

[10] González, C.; Schlegel, H. B. Reaction path following in mass-weighted internal coordinates. *J. Phys. Chem.*, **1990**, *94* (14), 5523–5527 https://doi.org/10.1021/j100377a021

[11] González, C.; Schlegel, H. B. Improved algorithms for reaction path following: Higher-order implicit algorithms. *J. Chem. Phys.*, **1991**, *95*, 5853–5860. https://doi.org/10.1063/1.461606

[12] Hehre, M. J.; Radom, L.; Schleyer, P. v. R.; Pople, J. Ab initio Molecular Orbital Theory, Wiley, New York, **1986**.

the Gaussian 09 software.[13] CDFT reactivity indices[14,15,16] were computed through the equations given in reference 15. The GEDT values were calculated by natural population analysis (NPA)[17,18] using the equation $\mathrm{GEDT}(f) = \sum_{q\in f} q$, were q is the natural charge, obtained with the NBO[19] program, of the atoms belonging to a framework at the transition structures. GaussView[20] and Molden[21] programs were used to visualize molecular geometries of all the systems and to animate the imaginary frequency of transition states. Non-Covalent Interaction (NCI)[22] analysis was carried out with the NCIPLOT4 program[23] and visualized with the VMD[24,25] software.

---

[13] Frisch, M. J.; Trucks, G. W.; Schlegel, H. B.; Scuseria, G. E.; Robb, M. A.; Cheeseman, J. R.; Scalmani, G.; Barone, V.; Petersson, G. A.; Nakatsuji, H.; et al. *Gaussian 09 Version D.01*; Gaussian, Inc.: Wallingford CT, 2009.

[14] Parr R. G.; Yang, W. Density Functional Theory of Atoms and Molecules, Oxford University Press, New York, NY, USA, **1989**.

[15] Domingo, L. R.; Ríos-Gutiérrez, M.; Pérez, P. Applications of the Conceptual Density Functional Theory Indices to Organic Chemistry Reactivity. *Molecules*, **2016**, *21(6)*, 748. https://doi.org/10.3390/molecules21060748

[16] Pérez, P.; Domingo, L. R.; Aizman, A.; Contreras, R. The Electrophilicity Index in Organic Chemistry. In Theoretical Aspects of Chemical Reactivity; Elsevier: New York, NY, USA, **2007**; Volume 19.

[17] Reed, A. E.; Weinstock, R. B.; Weinhold, F. Natural Population Analysis. *J. Chem. Phys.*, 1985, *83*, 735–746. https://doi.org/10.1063/1.449486

[18] Reed, A. E.; Curtiss, L. A.; Weinhold, F. Intermolecular interactions from a natural bond orbital, donor-acceptor viewpoint**.** *Chem. Rev.*, **1988**, *88(6)*, 899–926. https://doi.org/10.1021/cr00088a005

[19] Glendening, E. D.; Reed, A. E.; Carpenter, J. E.; Weinhold, F. NBO Version 3.1, implemented in Gaussian suit of programs.

[20] Frisch, Æ.; Hratchian, H. P.; Dennington II, R. D.; Keith, T. A.; Millam, J.; Nielsen, A. B.; Holder, A. J.; Hiscocks, J. GaussView 5 Reference, Gaussian, Inc., Wallingford, CT, 2009.

[21] Schaftenaar, G., Noordik, J. Molden: a pre- and post-processing program for molecular and electronic structures. *J Comput Aided Mol Des.*, **2000**, *14*, 123–134. https://doi.org/10.1023/A:1008193805436.

[22] Johnson, E. R.; Keinan, S.; Mori-Sánchez, P.; Contreras-García, J. A.; Cohen J.; Yang, W. Revealing Noncovalent Interactions. *J. Am. Chem. Soc.*, **2010**, *132* (18), 6498–6506. https://doi.org/10.1021/ja100936w

[23] Boto, R. A.; Peccati, F.; Laplaza, R.: Quan, C.; Carbone, A.; Piquemal, J. P.; Maday Y.; Contreras-García, J. NCIPLOT4: Fast, Robust, and Quantitative Analysis of Noncovalent Interactions. *J. Chem. Theory Comput.*, **2020**, *16* (7), 4150–4158. https://doi.org/10.1021/acs.jctc.0c00063

[24] Humphrey, W., Dalke, A. and Schulten, K., VMD: Visual Molecular Dynamics, *J. Molec. Graphics*, **1996**, *14* (1), 33-38. https://doi.org/10.1016/0263-7855(96)00018-5

[25] https://www.ks.uiuc.edu/Research/vmd/

2.2. Table S1.

Electronic ($E_{SCF,298}$) and Gibbs free (G_{298}) energies (Hartrees), first frequency (cm^{-1}) computed at the PCM-ωB97X-D/6-31+G** level in toluene of the stationary points associated with the Diels-Alder reaction of 4-alkenyl-2-dimethylamino-1,3-thiazoles **1b,e** with nitroalkenes **2a,b** through the *endo-A, exo-A, endo-B and exo-B* paths.

	$E_{SCF,298}$	G_{298}	Freq	$\Delta E_{SCF,298}$	ΔG_{298}
2-dimethylamino-4-vinyl-1,3-thiazole (**1b**) + (*E*)-β-nitrostyrene (**2a**)					
**1b + 2a**	-1294,3174169	-1294,0892890		0,00	0,00
**TS1ba-*endoA***	-1294,3007032	-1294,0468930	-458,7	10,49	26,60
**INTba-*endoA***	-1294,3307027	-1294,0721480	33,1	-8,34	10,76
**TS2ba-*endoA***	-1294,3097011	-1294,0534620	-165,9	4,84	22,48
**3'ba-*endoA***	-1294,3772414	-1294,1181410	18,7	-37,54	-18,10
**3ba-*endoA***	-1294,3980289	-1294,1383300	24,0	-50,58	-30,77
**TS1ba-exoA**	-1294,3062506	-1294,0515000	-434,5	7,01	23,71
**INTba-*exoA***	-1294,3115395	-1294,0556170	29,0	3,69	21,13
**TS2ba-*exoA***	-1294,3114810	-1294,054562	-110,4	3,72	21,79
**3'ba-*exoA***	-1294,3774942	-1294,1183910	21,5	-37,70	-18,26
**3ba-*exoA***	-1294,3993563	-1294,1387710	35,8	-51,42	-31,05
**TS1ba-*endoB***	-1294,2969257	-1294,0415940	-475,0	12,86	29,93
**3'ba-*endoB***	-1294,3726955	-1294,1129870	18,4	-34,69	-14,87
**3ba-*endoB***	-1294,3896288	-1294,1293580	23,9	-45,31	-25,14
**TS1ba-*exoB***	-1294,2981971	-1294,0441400	-486,0	12,06	28,33
**3'ba-*exoB***	-1294,3756557	-1294,1164470	25,6	-36,55	-17,04
**3ba-*exoB***	-1294,3967791	-1294,1356390	25,5	-49,80	-29,09
2-dimethylamino-4-vinyl-1,3-thiazole (**1b**) + nitroethylene (**2b**)					
**1b + 2b**	-1063,3220074	-1063,1681030		0,00	0,00
**TS1ba-*endoA***	-1063,3091538	-1063,1304320	-458,1	8,07	23,64
**INTbb-*endoA***	-1063,3421621	-1063,1595630	41,5	-12,65	5,36
**TS2bb-*endoA***	-1063,3211190	-1063,1402150	-157,7	0,56	17,50
**3'bb-*endoA***	-1063,3884977	-1063,2040690	21,9	-41,72	-22,57
**3bb-*endoA***	-1063,4089932	-1063,2233820	29,3	-54,58	-34,69
**TS1bb-*exoA***	-1063,3099967	-1063,1322990	-464,2	7,54	22,47
**3'bb-*exoA***	-1063,3920002	-1063,2076040	32,7	-43,92	-24,79
**3bb-*exoA***	-1063,4098672	-1063,2258430	28,2	-55,13	-36,23
**TS1bb-*endoB***	-1063,3066246	-1063,1272100	-461,5	9,65	25,66
**3'bb-*endoB***	-1063,3847564	-1063,1999750	45,9	-39,38	-20,00
**3bb-*endoB***	-1063,4074593	-1063,2231350	23,9	-53,62	-34,53
**TS1bb-*exoB***	-1063,3056391	-1063,1274650	-476,5	10,27	25,50
**3'bb-*exoB***	-1063,3876133	-1063,2045950	24,1	-41,17	-22,90
**3bb-*exoB***	-1063,4096485	-1063,2249510	34,4	-55,00	-35,67

(*E*)-2-dimethylamino-4-styryl-1,3-thiazole (**1e**) + (*E*)-*β*-nitrostyrene (**2a**)					
**1e + 2a**	-1525,3113660	-1525,0095570		0,00	0,00
**TS1ea-*endoA***	-1525,2988722	-1524,9701940	-453,6	7,84	24,70
**3'ea-*endoA***	-1525,3637124	-1525,0276930	23,2	-32,85	-11,38
**3ea-*endoA***	-1525,3839177	-1525,0479290	21,6	-45,53	-24,08
**TS1ea-*exoA***	-1525,3059744	-1524,9772330	-454,4	3,38	20,28
**INTea-*exoA***	-1525,3138487	-1524,9834480	15,1	-1,56	16,38[a]
**3'ea-*exoA***	-1525,3662534	-1525,0304850	23,6	-34,44	-13,13
**3ea-*exoA***	-1525,3872011	-1525,0522260	26,0	-47,59	-26,78
**TS1ea-*endoB***	-1525,2932209	-1524,9624150	-547,6	11,39	29,58
**3'ea-*endoB***	-1525,3628591	-1525,0276590	23,2	-32,31	-11,36
**3ea-*endoB***	-1525,3849310	-1525,0500180	24,3	-46,16	-25,39
**TS1ea-*exoB***	-1525,2952986	-1524,9636070	-549,8	10,08	28,83
**3'ea-*exoB***	-1525,3640042	-1525,0296510	16,9	-33,03	-12,61
**3ea-*exoB***	-1525,3858485	-1525,0502090	22,1	-46,74	-25,51
(*E*)-2-dimethylamino-4-styryl-1,3-thiazole (**1e**) + nitroethylene (**2b**)					
**1e + 2b**	-1294,3159565	-1294,0883710		0,00	0,00
**TS1eb-*endoA***	-1294,3070537	-1294,0533370	-449,8	5,59	21,98
**3'eb-*endoA***	-1294,3747568	-1294,1139680	32,8	-36,90	-16,06
**3eb-*endoA***	-1294,3940596	-1294,1339670	27,6	-49,01	-28,61
**TS1eb-*exoA***	-1294,3086373	-1294,0553510	-455,6	4,59	20,72
**3'eb-*exoA***	-1294,3787327	-1294,1199540	26,7	-39,39	-19,82
**3eb-*exoA***	-1294,3978160	-1294,1381240	28,7	-51,37	-31,22
**TS1eb-*endoB***	-1294,2987537	-1294,0450520	-473,5	10,79	27,18
**3'eb-*endoB***	-1294,3691899	-1294,1102930	25,6	-33,40	-13,76
**3eb-*endoB***	-1294,3952925	-1294,1362360	23,0	-49,78	-30,04
**TS1eb-*exoB***	-1294,2978390	-1294,0450770	-489,8	11,37	27,17
**3'eb-*exoB***	-1294,3756159	-1294,1171610	20,6	-37,44	-18,07
**3eb-*exoB***	-1294,3976304	-1294,1385680	28,1	-51,25	-31,50

[a] We have not been able to fully optimize **TS2ea-*exoA***

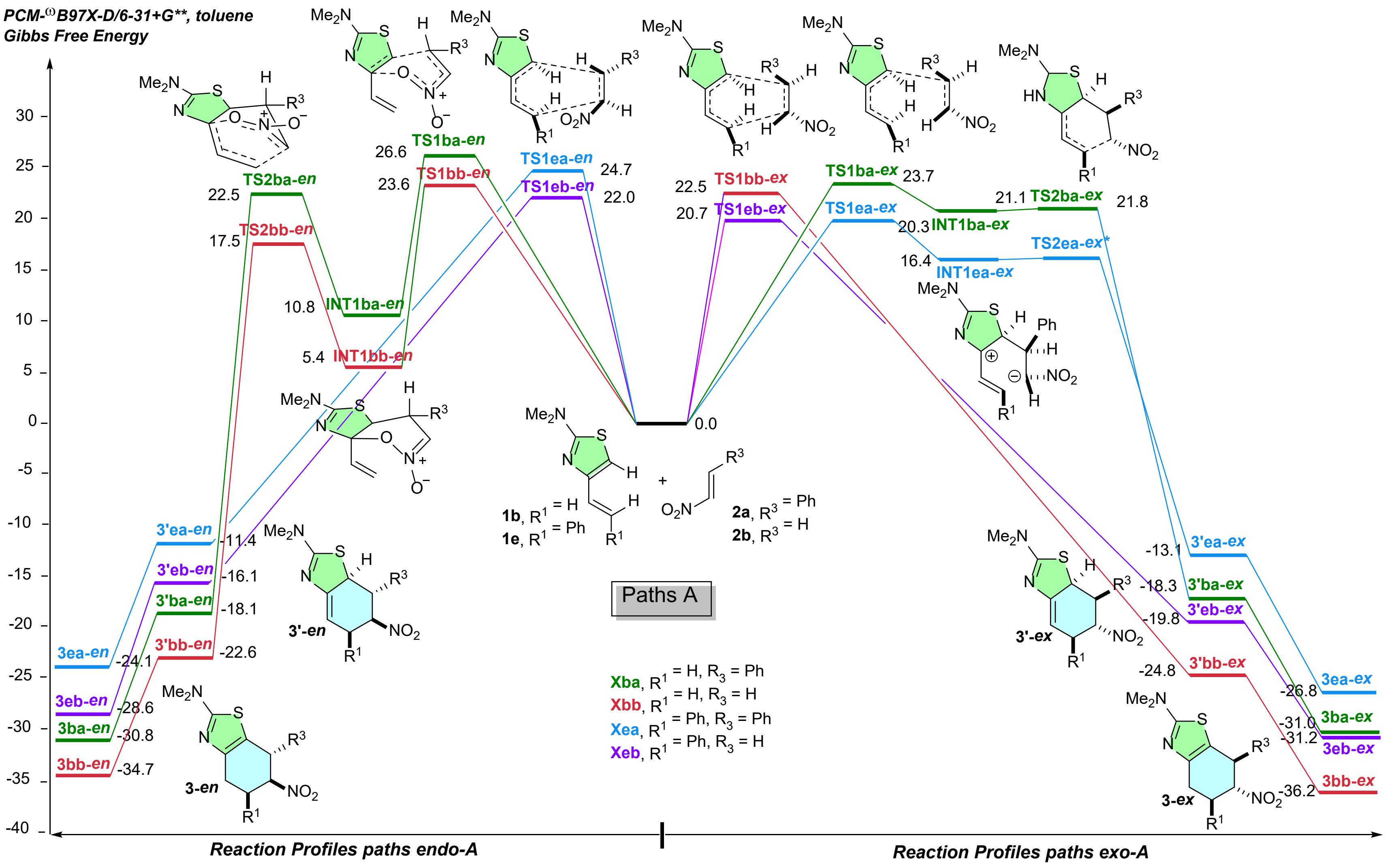

2.3. Figure S1.

Reaction profiles computed at the PCM/ωB97X-D/6-31+G** in toluene corresponding to the Diels-Alder reaction of 4-alkenyl-2-dimethylamino-1,3-thiazoles **1b,e** with nitroalkenes **2a,b** through the *endo-A and exo-A* paths (ΔG in kcal·mol^{-1}). The suffixes *en* and *ex* refer to *endo-A* and to *exo-A* mechanistic paths respectively.

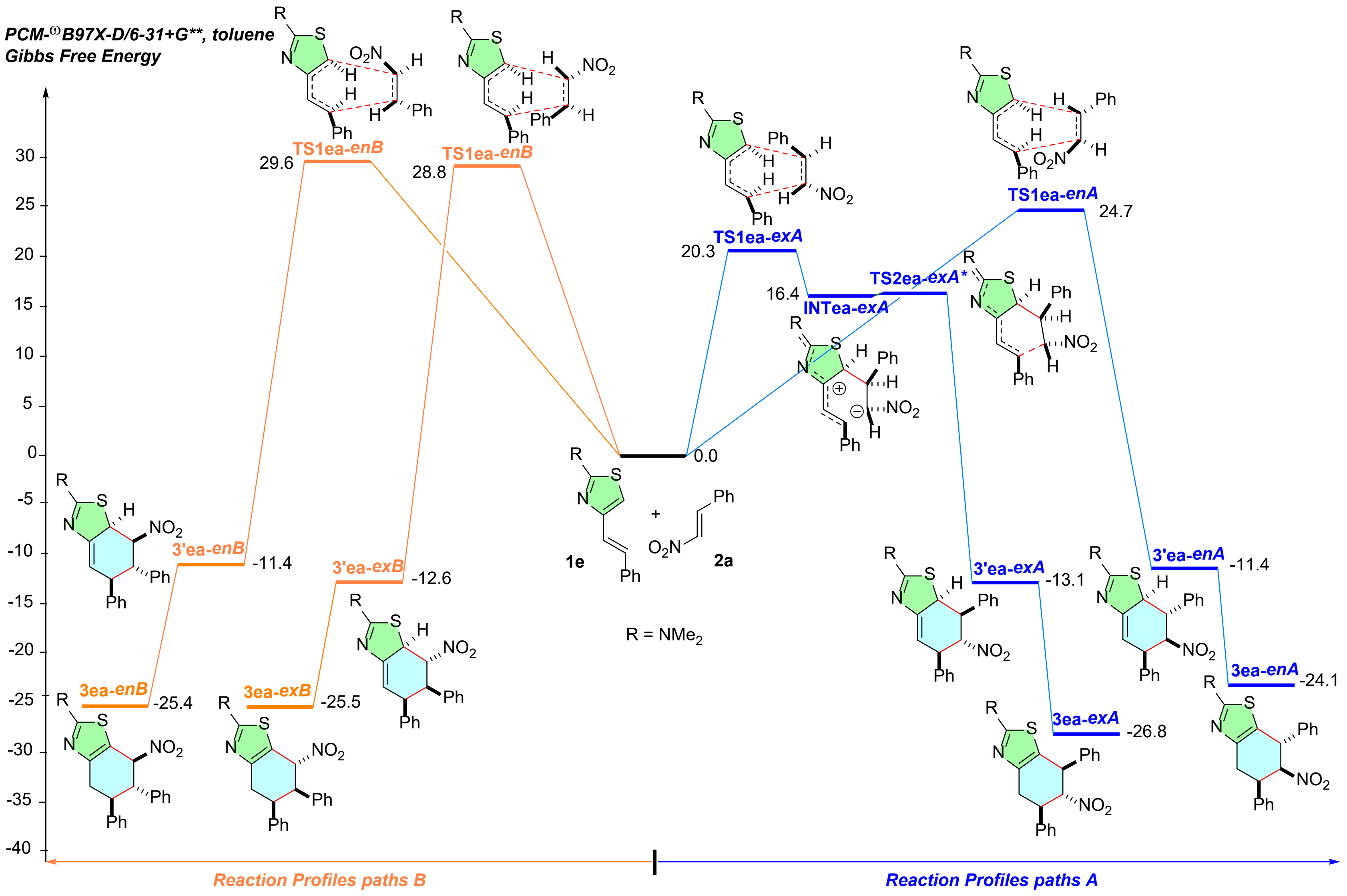

2.4. Figure S2.

Reaction profiles computed at the PCM/ωB97X-D/6-31+G** in toluene corresponding to the Diels-Alder reaction of 2-dimethylamino-4-styryl-1,3-thiazole (1e) with (*E*)-*β*-nitrostyrene (**2a**) through the *endo-A, exo-A, endo-B and exo-B* paths (Δ*G* in kcal·mol^{-1}). The suffixes *en* and *ex* refer to *endo* and to *exo* mechanistic paths respectively.

2.5. Table S2.

NCI isosurfaces of 0.3 associated with the density overlap and blue (hydrogen bonds)-green (van der Waals interactions)-red (steric crowding) color scale computed at the PCM/ωB97X-D/6-31+G** in toluene corresponding to the Diels-Alder reaction of 2-dimethylamino-4-styryl-1,3-thiazole (**1e**) with (*E*)-β-nitrostyrene (**2a**) through the *endo-A, exo-A, endo-B and exo-B* paths.

	TS1-*endoA*	TS1-*exoA*	TS1-*endoB*	TS1-*exoB*
**1b + 2b**	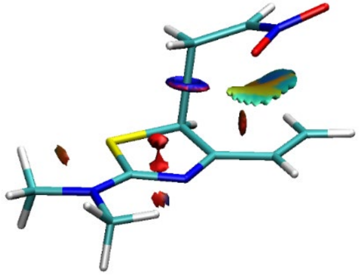	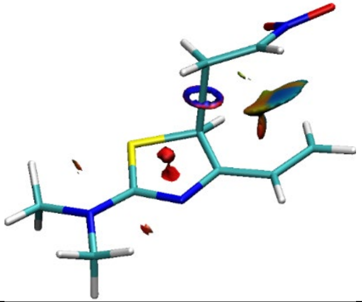	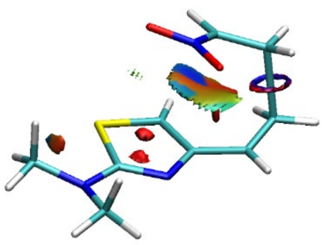	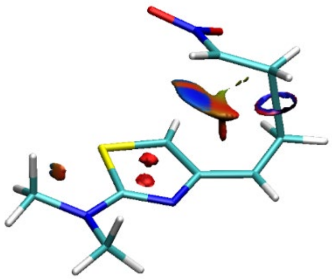
**1b + 2b**	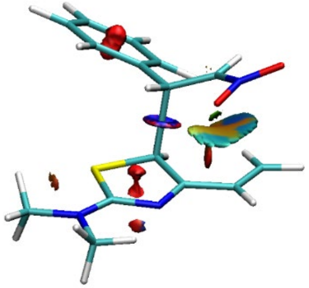	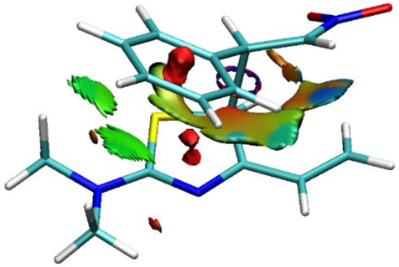	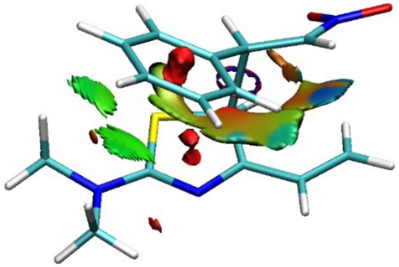	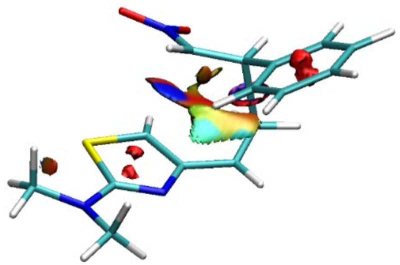
**1e + 2a**	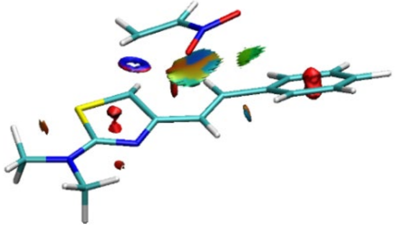	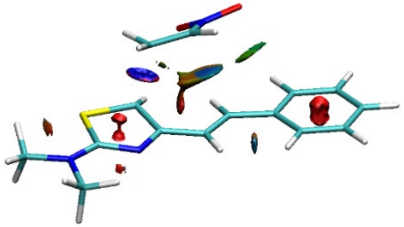	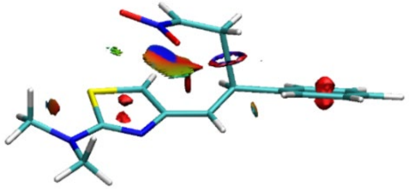	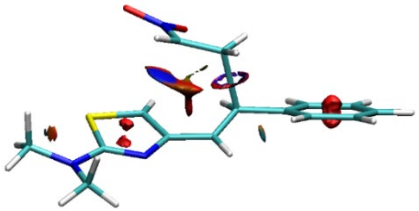

**1e + 2a**	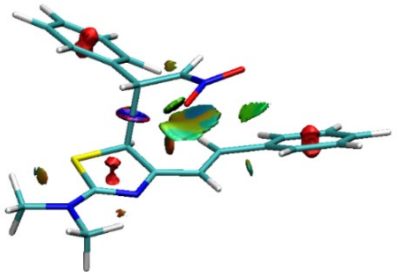	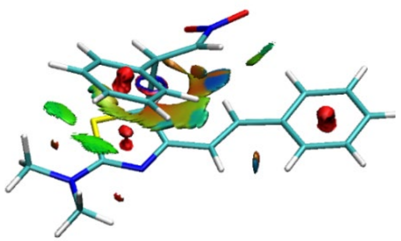	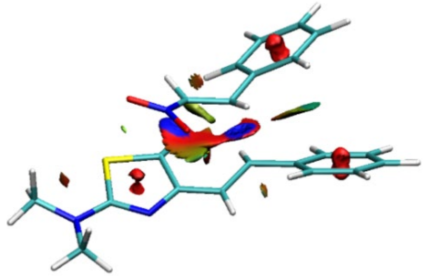	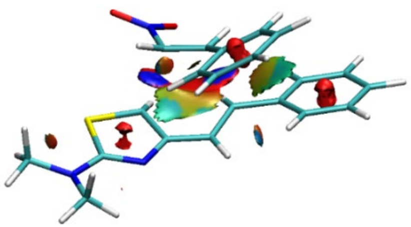

2.6. Table S3.

PCM/ωB97X-D/6-31+G** (toluene) optimized geometries and Cartesian coordinates of the stationary points associated with the Diels-Alder reaction of 4-alkenyl-2-dimethylamino-1,3-thiazoles **1b,e** with nitroalkenes **2a,b** through the *endo-A*, *exo-A, endo-B and exo-B* paths.

Series 1b + 2b

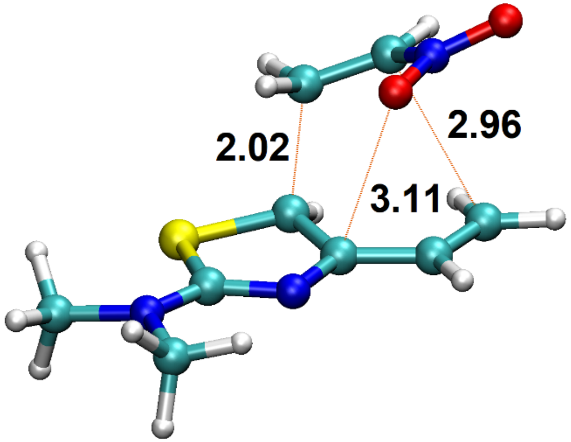

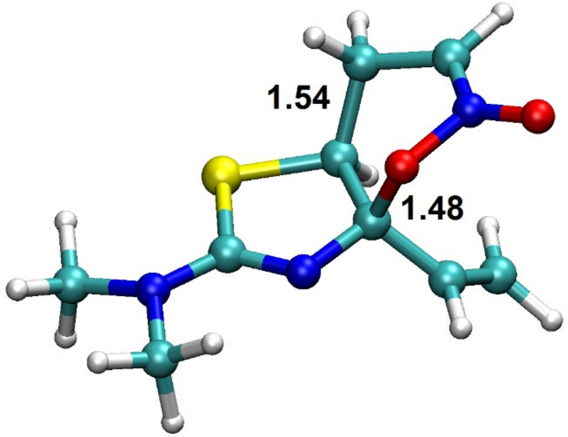

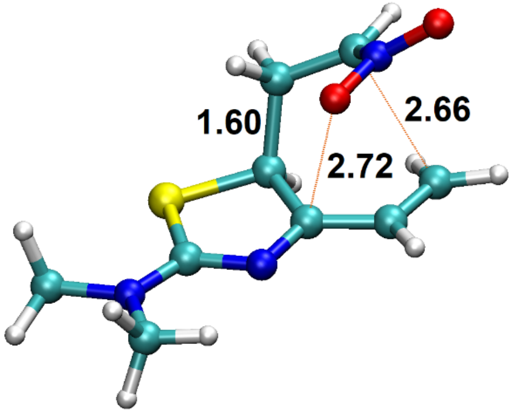

TS1bb-*endoA*
C,-0.4567680266,1.071170811,0.9085379956
C,-0.3238983023,-0.4207452984,0.9758812612
H,-0.8037852681,1.4697572119,1.8639529619
C,-1.4357590239,-1.2164995518,1.3615662695
C,-2.5871714582,-0.6452937637,1.7972479388
H,-1.3731985574,-2.2798712539,1.1617859266
H,-2.6414556498,0.3767645174,2.1483296819
N,0.8510574676,-0.9489694801,0.6482760376
S,1.2399972662,1.6580699211,0.6227720953
C,1.7627133953,-0.008782411,0.3939701293
O,-3.8616335022,-0.7988745016,-1.3792431195
N,2.9982426307,-0.2941159904,0.0124300406

INTbb-*endoA*
C,0.2622562729,0.7658218819,-0.9787957429
C,0.6703659272,-0.2578907696,0.1294113633
H,0.7284688563,0.5015480345,-1.9267945697
C,1.7996855218,-1.1976972021,-0.2223614289
C,2.6144459823,-1.13368902,-1.272172075
H,1.9321461868,-1.9754464229,0.5249427329
H,2.5390379994,-0.371500048,-2.0419278406
N,-0.4407041326,-1.0009233944,0.6123284157
S,-1.536241795,0.5824028392,-1.2008459998
C,-1.5685927994,-0.6492146145,0.0954590071
O,3.2246488304,1.1628515709,1.7349614584
N,-2.7553486939,-1.1621018384,0.4819881582

TS2bb-*endoA*
C,-0.4567680266,1.071170811,0.9085379956
C,-0.3238983023,-0.4207452984,0.9758812612
H,-0.8037852681,1.4697572119,1.8639529619
C,-1.4357590239,-1.2164995518,1.3615662695
C,-2.5871714582,-0.6452937637,1.7972479388
H,-1.3731985574,-2.2798712539,1.1617859266
H,-2.6414556498,0.3767645174,2.1483296819
N,0.8510574676,-0.9489694801,0.6482760376
S,1.2399972662,1.6580699211,0.6227720953
C,1.7627133953,-0.008782411,0.3939701293
O,-3.8616335022,-0.7988745016,-1.3792431195
N,2.9982426307,-0.2941159904,0.0124300406

C,3.9706427875,0.7535801452,-0.270607397
H,4.9692251527,0.3201018837,-0.2137855289
H,3.8169658287,1.1677804741,-1.272289874
H,3.904727439,1.5541838955,0.4698700413
C,3.4025875579,-1.6694055631,-0.272662615
H,3.7105656534,-1.7412284976,-1.3193652226
H,4.2413894802,-1.9420326156,0.3729120674
H,2.5639544227,-2.3369155321,-0.0921475341
O,-1.659483318,-0.7796122322,-1.3618600276
N,-2.7724323042,-0.2441961199,-1.0722141509
C,-2.740122865,0.9020584217,-0.3564717208
H,-3.6956464366,1.3565366858,-0.1438152301
C,-1.4421843874,1.5935239682,-0.237674024
H,-1.6040769558,2.655351299,-0.0362242661
H,-0.882552876,1.4900644421,-1.1711924935
H,-3.4795601507,-1.2454628652,1.9377457569

C,-3.9653654063,-0.9904555588,-0.3033295301
H,-4.0635984143,-1.7642838066,-1.074976326
H,-4.8276917874,-1.0475448742,0.3641417599
H,-3.9829239326,-0.0084091631,-0.7790140419
C,-2.7494981723,-2.2508211203,1.4496331133
H,-3.7168180894,-2.2733570045,1.9556484883
H,-2.5760998029,-3.2164687098,0.9586257286
H,-1.9586843337,-2.0832631844,2.1792344435
O,1.1213038465,0.5094327603,1.317883699
N,2.221225949,1.3355684738,1.0256465485
C,2.0150177353,2.1816042114,0.062257607
H,2.8430319096,2.8248621532,-0.1987533999
C,0.6680795839,2.1960727375,-0.5799472064
H,0.6794413392,2.8513854901,-1.4523631587
H,-0.0723625887,2.5920136767,0.1255172094
H,3.4095820079,-1.861993098,-1.3923394133

C,3.9706427875,0.7535801452,-0.270607397
H,4.9692251527,0.3201018837,-0.2137855289
H,3.8169658287,1.1677804741,-1.272289874
H,3.904727439,1.5541838955,0.4698700413
C,3.4025875579,-1.6694055631,-0.272662615
H,3.7105656534,-1.7412284976,-1.3193652226
H,4.2413894802,-1.9420326156,0.3729120674
H,2.5639544227,-2.3369155321,-0.0921475341
O,-1.659483318,-0.7796122322,-1.3618600276
N,-2.7724323042,-0.2441961199,-1.0722141509
C,-2.740122865,0.9020584217,-0.3564717208
H,-3.6956464366,1.3565366858,-0.1438152301
C,-1.4421843874,1.5935239682,-0.237674024
H,-1.6040769558,2.655351299,-0.0362242661
H,-0.882552876,1.4900644421,-1.1711924935
H,-3.4795601507,-1.2454628652,1.9377457569

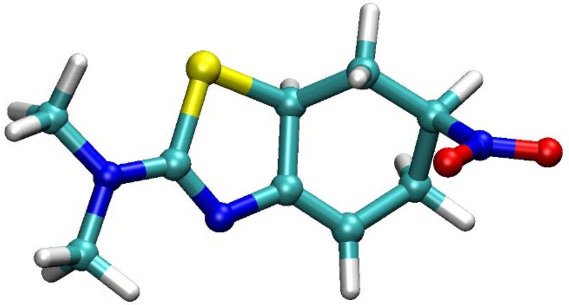

3’bb-*endoA*
C,-1.3286238903,0.9437594491,-0.996238108
C,-1.0756037307,-0.4696597244,-0.4880052312
H,-1.1527596281,0.9956076799,-2.0767234108
C,0.1964785615,-0.8884391447,-0.4436722892
C,1.2563873719,0.0678324648,-0.9107812628
H,0.4591259539,-1.8636222582,-0.0508753045
H,1.2185738869,0.2003995445,-2.0010597586
N,-2.1940412875,-1.1682759003,-0.0631667401

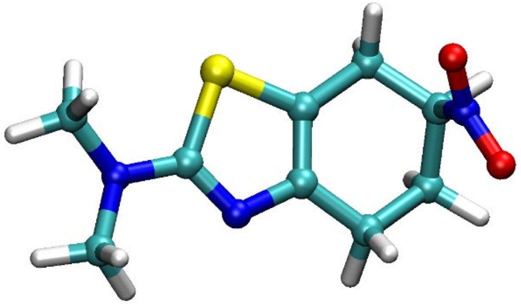

3bb-*endoA*
C,-0.2540360176,-0.5270665593,-0.6370827328
C,-0.032276736,0.7884326959,-0.388257692
H,-0.7806374354,2.686325306,-1.0073788238
C,-1.1217427601,1.8182221577,-0.4349644908
C,-2.3976881623,1.251266674,-1.069767557
H,-1.3251797143,2.176701666,0.5806065405
H,-2.2763254503,1.2024726988,-2.1576799304
N,1.2574588776,1.149056256,-0.0536190084

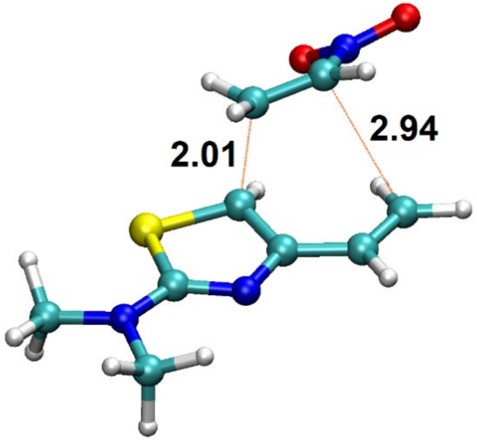

TS1bb-*exoA*
C,0.1875886181,-1.1960901731,0.5528830439
C,0.1563144114,0.1310455144,1.0439358909
H,0.7080647714,-2.0044438311,1.0556018586
C,1.2698924594,0.7595937041,1.7073614345
C,2.3817723212,0.0992655317,2.0766447051
H,1.1920035671,1.8342645326,1.8449964561
H,2.4653656459,-0.9808028152,2.0221729436
N,-0.9429291347,0.865426834,0.7378696621

S,-3.1033421715,1.23298033,-0.713012586
C,-3.2649744527,-0.4411516647,-0.0841619279
O,2.8745087573,1.6452127908,1.1381626252
N,-4.4689424397,-0.8605921197,0.361109255
C,-5.6913702052,-0.1382598859,0.052730456
H,-5.5310835602,0.939714414,0.1146908875
H,-6.0715663546,-0.3876824138,-0.9461062503
H,-6.448603594,-0.4021313509,0.7932474441
C,-4.613753422,-2.2582251211,0.7432465003
H,-3.6928634229,-2.6050613461,1.2091634719
H,-5.4379431094,-2.3429509807,1.4545772047
H,-4.8261030245,-2.8883818362,-0.1297064879
O,0.9602001277,1.2174618438,2.0421554796
N,1.6658774613,1.4608586998,1.0785560689
C,1.0581857285,1.4844473382,-0.3114864324
H,1.6964581681,2.1902215047,-0.8418115033
C,-0.4082861241,1.9455382237,-0.2748281117
H,-0.4929304772,2.9355838876,-0.7290606406
H,-0.7332177811,2.0226321433,0.7642940131
H,2.2638428086,-0.285155307,-0.6802184808

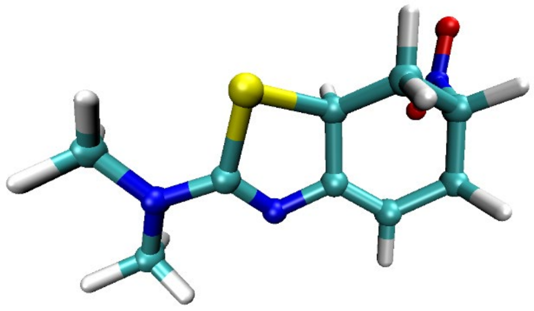

3’bb-*exoA*
C,-1.2060782118,1.0694335014,-0.0644715474
C,-1.0137498056,-0.3911217237,-0.4484131185
H,-1.088307089,1.7343305907,-0.9595304734
C,0.2203424173,-0.9019969152,-0.5737547769
C,1.4399037956,-0.0580334295,-0.3667646678
H,0.3911858921,-1.9441190048,-0.8371959793
H,1.8318209805,0.2206856761,-1.3750080805

S,1.2374064761,-1.4152435455,-0.453917905
C,2.0438276103,0.1062323082,-0.0658428979
O,-3.458885564,0.7730158218,1.3881325694
N,3.3871596088,0.1519573793,0.1614532629
C,4.1032289921,-1.0756492156,0.4532054505
H,3.8711222108,-1.4686922662,1.4533108261
H,3.8661307949,-1.8409795791,-0.2911135825
H,5.1751375901,-0.8803933974,0.3925798759
C,3.9268074079,1.3831161011,0.717435501
H,3.4737024115,2.2381604852,0.2168194998
H,3.7270351067,1.4655575833,1.7946298389
H,5.0059046104,1.3986671944,0.5509619106
O,-2.8171969249,-1.2842870065,1.4586456628
N,-2.9972985361,-0.2311701062,0.8695320752
C,-2.6997672006,-0.1798049997,-0.6281295058
H,-3.6418388788,-0.5249061485,-1.0654447732
C,-1.5732768491,-1.1476449465,-0.9853340491
H,-1.6463796157,-1.3467094192,-2.0614938075
H,-1.7237444405,-2.100060852,-0.4733480872
H,-3.2498664114,1.9006937142,-0.8656101703

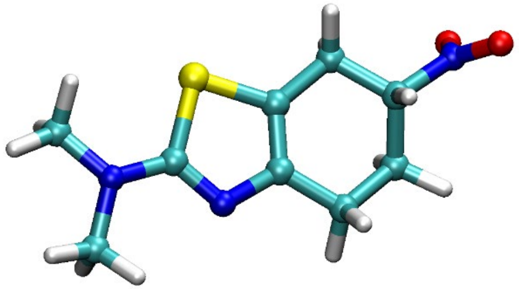

3bb-*exoA*
C,0.1106934829,-0.4585576517,-0.0331678444
C,-0.2302657945,0.8538350743,0.0220914678
H,0.4906244593,2.6885925003,0.8328202079
C,0.7898098197,1.9521713087,0.0812386281
C,2.1768398705,1.397144413,0.4238997859
H,0.8149755378,2.4789356846,-0.8807654765
H,2.2299330378,1.1366651643,1.4858569852

S,-1.4232832621,-1.571033984,-0.0666301816
C,-1.8601347855,0.1214221618,0.1404436453
O,4.7435463571,-1.756762661,0.0653416163
N,-3.0349432995,0.5950655917,-0.2953198509
C,-4.0516234347,-0.3102873536,-0.8059058988
H,-4.4972685975,-0.9103735129,-0.0032048683
H,-4.8367262613,0.2783065321,-1.2801740822
H,-3.6271142919,-0.9786272252,-1.5605151416
C,-3.4247252995,1.9641818564,0.0202876855
H,-4.0084291178,2.3653418232,-0.8103720511
H,-4.0276737962,1.9957196109,0.9347831045
H,-2.531351869,2.5687203793,0.1640166249
O,2.964138081,-3.0109221901,0.1695378028
N,3.5302417119,-1.9384651019,-0.1273689032
C,2.7687917793,-0.897309474,-0.6579239281
H,3.2929110721,0.0411762648,-0.7413092246
C,1.4467314217,-1.128061105,-1.0147527997
H,1.1833688465,-2.1441197469,-1.2894836466
H,0.9562665465,-0.3480119494,-1.589934128
H,3.2252033191,0.6270421764,2.5082411706

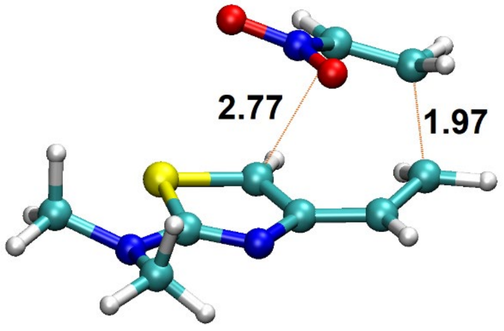

TS1bb-*endoB*
C,-0.785787721,1.0191703797,-0.7553219439
C,-1.0641346375,-0.2449689095,-1.2596874434
H,0.1181308637,1.5986854711,-0.8588920069
C,-0.0683591345,-1.1525206733,-1.6842825538
C,1.2888961704,-0.8570523455,-1.6204122387
H,-0.3963786401,-2.1661900123,-1.8930474485
H,1.6055400565,0.1728162592,-1.7581358724

N,-2.21406245,-1.1465794683,-0.5637402329
S,-2.9355393365,1.1630907861,0.5213847293
C,-3.2632838457,-0.5300015401,-0.0760147266
O,1.1120230321,2.6378968706,-1.3906629933
N,-4.524211306,-1.0559416767,0.1603014252
C,-5.6543509849,-0.0929098156,0.2209444765
H,-5.5273908322,0.5806186894,1.0895787613
H,-5.763123401,0.5198360715,-0.6895971685
H,-6.5999648967,-0.6382991162,0.3815671944
C,-4.8469991002,-2.3389383914,-0.5219515204
H,-3.9797897847,-3.0261197897,-0.4570992344
H,-5.6929123366,-2.818978138,-0.0044330773
H,-5.0945597225,-2.2141385405,-1.5879929241
O,2.4685344986,3.1754695062,0.2073207794
N,1.6380988393,2.4493620788,-0.3100091753
C,1.2335846792,1.2059612719,0.499932605
H,1.9308794075,1.1640852093,1.3807900532
C,-0.2007992153,1.4279917554,1.0193190462
H,-0.3398248775,2.4686744335,1.3760952896
H,-0.3523973616,0.7872194458,1.91785517
H,2.2372899158,-0.6777998971,0.0974510666

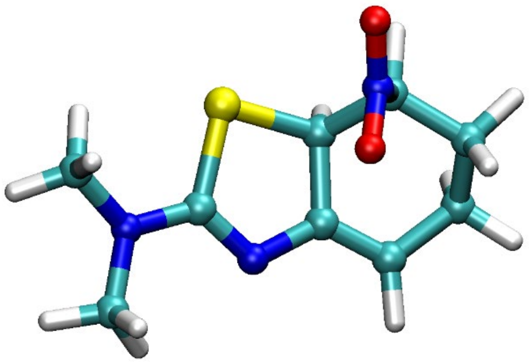

3’bb-*endoB*
C,0.6627377571,0.2382660892,-0.8525249559
C,1.1807658128,1.3842115423,0.0092750325
H,0.3276855287,0.6289763335,-1.8195597274
C,0.2885068681,2.2841936412,0.4385349886
C,-1.1580362085,2.0474568978,0.1007080262

N,-1.5785168125,1.1476893307,0.0152966267
S,-1.3355945685,-1.4350063789,-0.0906332839
C,-2.2867461011,0.0524055704,-0.0578036612
O,4.6553965127,-0.1708042051,-1.1176900646
N,-3.6466724351,0.0179619972,-0.1353289619
C,-4.3474244132,-1.2272536095,0.1166223012
H,-3.9106679338,-2.0389898544,-0.471561862
H,-4.3291812129,-1.5078629916,1.1795137693
H,-5.3860620293,-1.1143266528,-0.1983515211
C,-4.3639206434,1.249982278,0.1550990164
H,-3.8455957928,2.0909895554,-0.3037810447
H,-5.3693694431,1.1747867565,-0.2641774389
H,-4.4369133223,1.4313291372,1.2362331688
O,4.1865144971,-0.707328897,0.9239612619
N,3.8783352587,-0.289143233,-0.1817915887
C,2.4513734607,0.1367584483,-0.4029373842
H,2.3973271189,0.3580930785,-1.4699338956
C,1.5052719928,-1.0098787198,-0.0349399392
H,1.7780026415,-1.4049782516,0.9498090935
H,1.6125817388,-1.821926961,-0.7616014716
H,2.9445380732,2.1494411091,0.2225291259

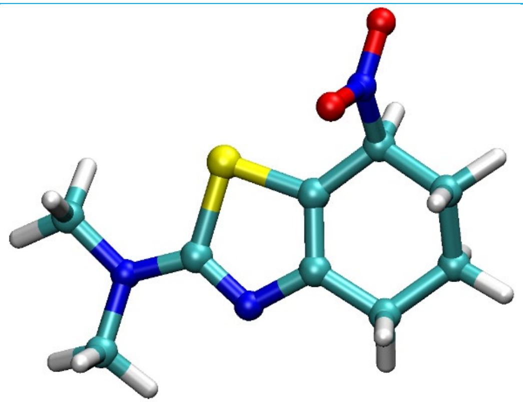

3bb-*endoB*
C,-0.8834761597,0.0450795249,-0.1231762833
C,-1.3479507556,-1.1819038157,0.2368097476
C,-0.4364757409,-2.3259994038,0.5673691651
C,0.9593909422,-2.07441648,-0.0102899983
H,-0.3812056912,-2.4433548638,1.6571474191

N,-2.3810509041,-0.6536886887,-1.1275232864
S,-2.2035450776,1.7305536757,-0.0984270093
C,-3.0978256397,0.256168556,-0.5399585721
N,-4.4255083627,0.1486361027,-0.2773332283
C,-5.0459608911,1.0540925091,0.6750163648
H,-4.7783626471,2.0899417167,0.4502871675
H,-6.1296742698,0.9696311456,0.5812515714
H,-4.7570829777,0.8248675504,1.709470969
C,-5.0168180361,-1.1769990935,-0.4050806944
H,-4.7619454138,-1.8121259101,0.4531549291
H,-6.1014264035,-1.0709667068,-0.4689159212
H,-4.647591168,-1.6533725722,-1.3122859557
C,1.7748891489,-0.9221682277,0.2886705814
C,0.9509268407,-0.1289300254,1.0795331218
H,1.1507718059,0.8845356446,1.391636153
H,1.6778623839,-1.9952605776,0.4060315446
N,-0.2077292108,-0.6857850782,1.640871931
O,-0.8745847509,0.002765729,2.4314118458
O,-0.5307051669,-1.8412898315,1.3191237416
H,1.9832268679,-1.5888080528,-2.0233678225
H,2.7884402449,-0.5575402448,0.1488715267

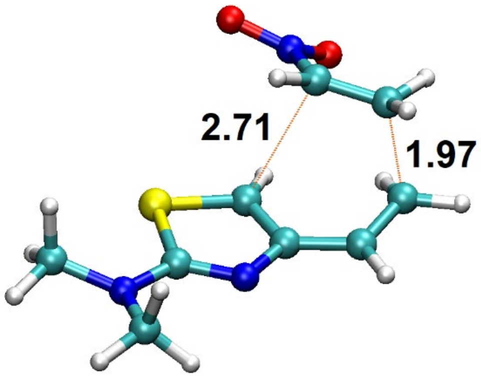

TS1bb-*exoB*
C,1.2034836571,1.2996641831,0.7697644983
C,0.7938310077,0.0929292846,1.320880651
H,0.8333684325,2.2912569894,0.9866523621
C,-0.4481138065,-0.0782141933,1.9692184126
C,-1.4110714355,0.9217675048,2.0110529043

H,0.5925360415,3.1216179074,1.0565893193
H,-1.3611017666,2.2758584426,-0.9562258842
N,2.5407018565,1.3572321478,0.2765984455
S,2.1006199458,-0.8328697515,-1.1520195217
C,3.1247078597,0.3202165261,-0.2251670475
N,4.4495863586,0.0729431541,-0.1231246423
C,5.0001453071,-1.2398141229,-0.41765822
H,4.8779216068,-1.9336224346,0.4235284043
H,4.52471422,-1.6677913965,-1.3024585546
H,6.0641515821,-1.1316375977,-0.635707369
C,5.2328570175,0.9348760951,0.7514679273
H,6.2861281486,0.8398925581,0.4809595791
H,4.9159421757,1.9691438667,0.6232451036
H,5.1036682705,0.6586207036,1.8054052251
C,-1.5507869031,0.5827500619,0.3893980626
C,-0.5856853171,-0.4442351108,-0.2467223926
H,-1.0784001382,-1.0489763039,-1.0061267806
H,-1.5431173534,0.4413991001,1.4724974714
N,-0.1688913536,-1.4599248538,0.7903656399
O,-0.451441672,-2.6286117507,0.5698227273
O,0.4288317904,-1.0653269289,1.7767554564
H,-1.8077076397,2.7030379885,0.6854746684
H,-2.5647718951,0.3817398454,0.0352591781

H,0.9317074953,-2.1917884542,-1.1002919534
N,-2.7120440278,-1.3434597842,0.2472318715
S,-2.2268842509,1.0938013121,-0.5144348727
C,-3.3116062693,-0.2413585378,-0.1313832672
N,-4.6558245513,-0.1031366746,-0.2558462579
C,-5.2321961471,1.2148447039,-0.4448871833
H,-5.1781321085,1.8248767407,0.4672700733
H,-4.7236098309,1.7427124368,-1.2569588189
H,-6.2794080793,1.1014472715,-0.7291187022
C,-5.5101560826,-1.1327162752,0.316351397
H,-6.4575626175,-1.1462999255,-0.2269195829
H,-5.0228841406,-2.1014473738,0.2178871236
H,-5.7080848298,-0.9439870722,1.379805389
C,1.4588047493,-0.673839175,0.3480978892
C,0.5507694521,0.4145604487,-0.253109246
H,0.8350730853,0.645287973,-1.2823766601
H,1.4572826002,-0.5597335274,1.4375225037
N,0.8639259121,1.7120337459,0.4887082389
O,1.5363480644,2.5405071104,-0.1083160746
O,0.4589070015,1.839408989,1.6309940792
H,-0.8643526528,-3.2506690404,0.1696475125
H,1.6671015313,-2.8142341847,0.3744959101
H,2.4806153723,-0.5163681386,-0.007122909

H,-0.7086218815,-1.0925621459,2.2594024666
H,-1.1059727781,1.9656419518,2.0413667551
N,1.6052898458,-0.9961290677,1.0320748446
S,2.6755164531,1.0954829124,-0.097551148
C,2.6313386517,-0.634054273,0.3169727765
N,3.6236438056,-1.465525372,-0.0876447245
C,4.5468314173,-1.0460395235,-1.1275510537
H,4.0779489247,-1.0369369318,-2.1207339282
H,4.9413219654,-0.0492512357,-0.912019355
H,5.3916932832,-1.7358487305,-1.1438083314
C,3.4212965239,-2.8946878842,0.0998852952
H,2.9854889038,-3.0749648657,1.0820529143
H,2.7511900812,-3.3125584907,-0.6629738501
H,4.3897414093,-3.3943442931,0.0397771117
C,-2.0555344052,1.0571077762,0.1574515525
H,-2.7972392577,1.7993874494,0.4381632772
C,-1.0548630063,1.4612759609,-0.721863268
H,-0.5493163593,0.8261244425,-1.4325477401
N,-0.7310201855,2.8210990587,-0.8456014661
O,0.0064641748,3.1708860903,-1.7776186156
O,-1.160254785,3.6212678976,0.0052755449
H,-2.3284584114,0.7107465385,2.5533126173
H,-2.4438098449,0.0569389068,0.0004328063

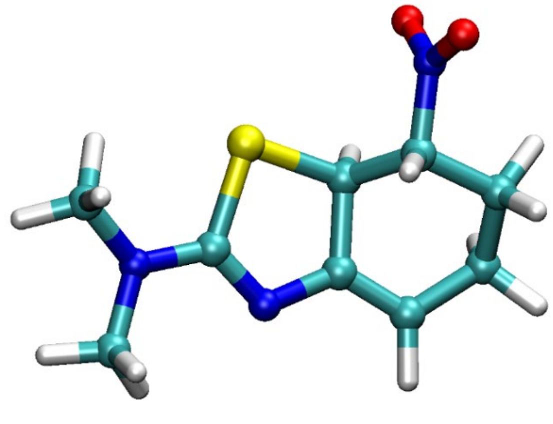

3’bb-*exoB*
C,0.8786134817,0.7305377851,0.8521999048
C,1.1027014863,-0.7498755374,1.1571951416

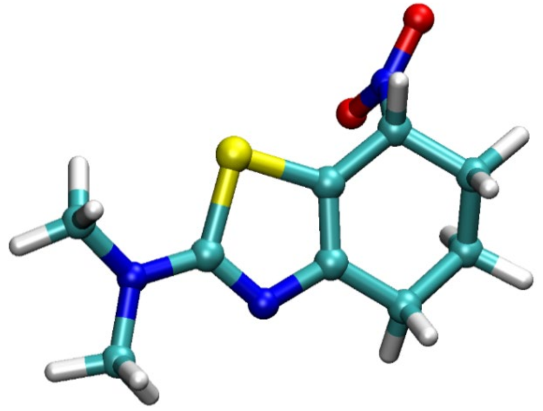

3bb-*exoB*
C,0.5799745488,0.6060381927,-0.1063835351
C,0.7718296426,-0.1226346691,1.0270198666

H,0.8739916356,1.3333363106,1.7650099167
C,0.1190482921,-1.4123841222,1.7787436978
C,-1.1182945549,-0.6484451056,2.1671146883
H,0.2186180767,-2.4680601695,2.0060165165
H,-0.9122273145,-0.0385932791,3.05780063
N,2.2957881053,-1.2800136275,0.6884570442
S,2.2998620194,1.228418378,-0.1670333206
C,2.9920130061,-0.4191525056,0.0194160085
N,4.2038494906,-0.683823363,-0.5157671433
C,4.8089103578,0.1989517508,-1.4994383032
H,4.4142722618,0.0196599436,-2.5076385066
H,4.6428284775,1.2446530286,-1.2335280866
H,5.8869733676,0.028615728,-1.5083331176
C,4.672850203,-2.0623262202,-0.4868553109
H,5.7550981617,-2.0664245726,-0.6289985736
H,4.4313121817,-2.5094321473,0.4765357754
H,4.2024979945,-2.660408375,-1.2774927941
C,-1.6243627774,0.2833408585,1.0389105632
H,-2.1800846341,1.1142308493,1.4828672096
C,-0.476729956,0.839081963,0.1575376495
H,-0.4386813034,0.3254063584,-0.8032656537
N,-0.8143754053,2.263737262,-0.1994531222
O,-0.3933185987,3.1608821705,0.5150090169
O,-1.5315275295,2.4305331956,-1.1733766986
H,-1.9243826659,-1.3308921241,2.4458685949
H,-2.3142467298,-0.2551188026,0.3864861332
C,-0.2819266097,-0.2808769288,2.0817028988
C,-1.4059221686,0.7454027501,1.9047042671
H,-0.6799454391,-1.3025860197,2.0225216983
H,-1.0702247048,1.7216361619,2.2711534113
N,1.9822371186,-0.7585904852,1.1502437363
S,2.0083935881,0.5225095122,-1.1120238327
C,2.7489149735,-0.4971373189,0.1188526835
N,4.0240217444,-0.9418129786,-0.0116916688
C,4.6962405766,-0.8436802371,-1.2935128802
H,4.3073521599,-1.5663267897,-2.0243875199
H,4.5929387351,0.1653384627,-1.7026882171
H,5.7610214576,-1.0307469542,-1.1473367637
C,4.4833348793,-1.9608907922,0.9203098126
H,5.574458488,-1.9821118358,0.9035920115
H,4.1435990874,-1.7147215445,1.9257860423
H,4.0977623486,-2.9541984255,0.6551542549
C,-1.8275979764,0.8683434797,0.4389042511
H,-2.6656271943,1.5608673565,0.3209798559
C,-0.6654861072,1.3202364154,-0.4632731102
H,-0.9409844801,1.2162277536,-1.5140980023
N,-0.5248007795,2.8432346297,-0.3068965009
O,0.2411738046,3.2870386866,0.5292553831
O,-1.2421901654,3.5257817739,-1.0251915757
H,0.180549193,-0.1838468953,3.0685299116
H,-2.2721171393,0.4582107241,2.507309436
H,-2.1571339719,-0.1051915945,0.0597673756

Series 1b + 2a

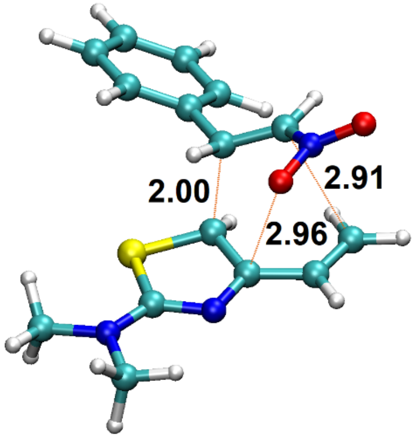

TS1ba-*endoA*
C,-0.1650320604,0.0046385993,0.9840552512
C,-1.2130998578,0.9738989749,0.9939693322
H,0.572728527,-0.0155550105,1.777235485
C,-0.9980886371,2.3420537536,1.3863235479
C,0.1682956088,2.7887581197,1.8863778192
H,-1.8087866345,3.0308341345,1.1721473264
H,0.9798062028,2.1234163465,2.1586295822
N,-2.4152075288,0.5767675476,0.5283765941
S,-0.9085248804,-1.5592351795,0.6157252224
C,-2.4139384014,-0.7182268357,0.2506217108
O,1.0462220636,3.9065681341,-1.3045122088
N,-3.4554607314,-1.365005425,-0.2808578837
C,-3.424966619,-2.8072477444,-0.4611814272
H,-2.4941547394,-3.115708151,-0.9458599927
H,-3.5259266243,-3.3326448838,0.4958195151
H,-4.254042408,-3.0942186208,-1.1080701818
C,-4.7183103706,-0.6740984085,-0.5125279747
H,-5.0742203941,-0.9044025139,-1.5198087033
H,-5.4667699911,-0.9986204955,0.2180643617
H,-4.5630581666,0.3982342431,-0.4180114922
O,-0.5008632329,2.3803776703,-1.5151967034
N,0.635778216,2.7407265462,-1.1463343157
C,1.4596302816,1.813128899,-0.5274483204

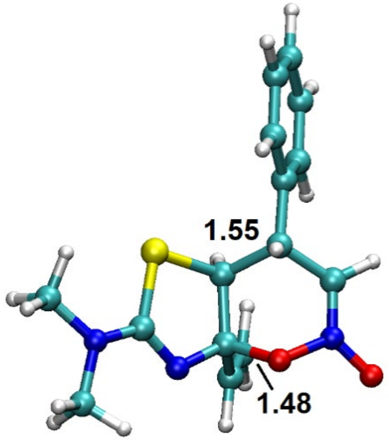

INTba-*endoA*
C,0.8734039262,0.0259910918,-0.1059392059
C,-0.0115029919,1.1002341879,0.6011833318
H,0.290006508,-0.5356185491,-0.8345638957
C,-1.4936921696,1.0264765554,0.3202767528
C,-2.1576669757,0.0521037829,-0.2956954841
H,-2.0276148608,1.8850107125,0.7186295698
H,-1.6797012521,-0.8338574724,-0.7025065486
N,0.4579705859,2.4216151309,0.3666803621
S,2.148530482,0.940220051,-1.0290687317
C,1.5225188176,2.4749027681,-0.3593558699
O,-1.1002929011,-0.3942863671,3.3772059089
N,2.1781917677,3.6226667728,-0.6345775842
C,3.1659922333,3.705809835,-1.6971628511
H,3.7628223545,2.7930633399,-1.7435556195
H,2.698472364,3.876839889,-2.6749962787
H,3.8452621324,4.5329100226,-1.4818530827
C,1.5819138884,4.8762137242,-0.1934514755
H,1.1022051516,4.7290929282,0.7727883269
H,2.3708443936,5.6251807274,-0.099633008
H,0.8299031911,5.2317684365,-0.9089619885
O,0.1420180237,0.933955834,2.0675451714
N,-0.2297095369,-0.3517007296,2.4944107686
C,0.4233095825,-1.3161914603,1.9207976873

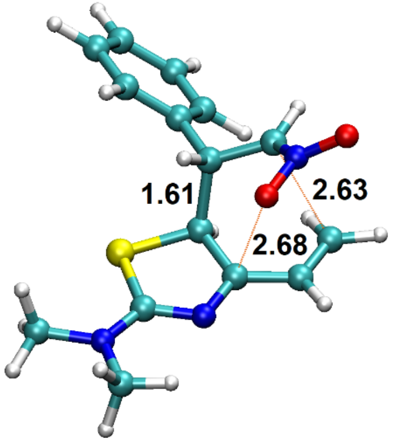

TS2ba-*endoA*
C,-0.0229040693,0.1110421065,0.8622394672
C,-1.1773481274,1.0631581951,0.9247911935
H,0.5351363914,0.1159221648,1.8008854657
C,-0.9510827402,2.4268125344,1.248713764
C,0.2830230799,2.8594953239,1.614972206
H,-1.756583303,3.1237608164,1.0486957902
H,1.056032055,2.1922861251,1.9725804026
N,-2.3874721653,0.5801974514,0.6578277249
S,-0.7945673742,-1.5218051536,0.6625405542
C,-2.3574940108,-0.7373600602,0.4518402831
O,0.8922341827,3.7326165525,-1.6393356774
N,-3.4335038049,-1.4378661961,0.1260137121
C,-3.362089362,-2.8763263733,-0.0960016073
H,-2.9423871586,-3.0987315818,-1.0825093054
H,-2.7539212583,-3.3565595299,0.6745541057
H,-4.3704865554,-3.2858487452,-0.0374636017
C,-4.7110812406,-0.7825661605,-0.1455383905
H,-5.0224017903,-1.018216776,-1.1667271401
H,-5.4639275146,-1.1475937637,0.5579502926
H,-4.5951139119,0.2928782428,-0.0379350737
O,-0.6008622014,2.1240674931,-1.4687936352
N,0.56206328,2.5806793039,-1.2530861149
C,1.4018386451,1.8107372327,-0.5231540223

H,2.4084877938,2.2045633426,-0.1947051415
C,1.0802155372,0.4623401266,-0.5124836218
H,0.3497681055,0.1991906557,-1.274413869
C,2.1498168099,-0.5544234331,-0.293565886
C,2.1849912179,-1.696777393,-1.0980784679
C,3.1219636218,-0.4002653906,0.7018971067
C,3.1762188299,-2.659919758,-0.9214762228
H,1.4336830085,-1.8281971848,-1.8714204881
C,4.1110994313,-1.3619776483,0.8808998341
H,3.1059514192,0.4759942694,1.3452599336
C,4.1417179712,-2.4956656426,0.0685305424
H,3.1927126611,-3.5382183757,-1.5591785965
H,4.8592706839,-1.227416714,1.6557812991
H,4.9132733062,-3.2460319354,0.2086122315
H,0.3192939809,3.8442813808,2.0829098019

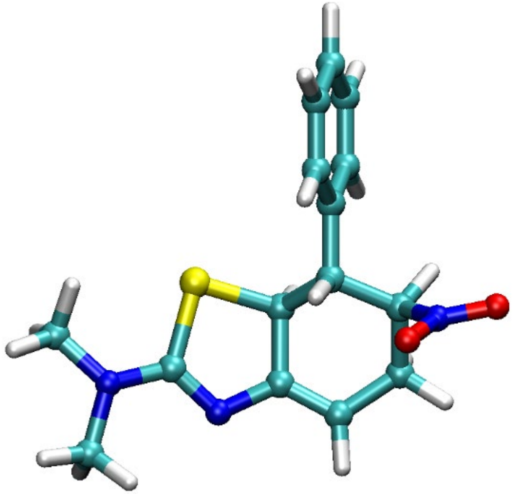

3’ba-*endoA*
C,-1.1251378249,-0.1520433009,-0.746468957
C,-0.6679231671,-1.5686734372,-0.4212993754
H,-0.9344193058,0.0934384548,-1.7976731687
C,0.650202499,-1.7932679777,-0.3436648875
C,1.5806843681,-0.657692751,-0.6575406627
H,1.0418979902,-2.7636397565,-0.0622141618
H,1.6264484785,-0.4858970525,-1.7415103722
N,-1.6771792261,-2.4805039478,-0.1545437669

H,0.1706497454,-2.3217895194,2.2265131359
C,1.4826506266,-0.9593142768,0.9225754767
H,2.276345407,-0.4145105217,1.4503311255
C,2.0852883204,-2.1761477893,0.2542555301
C,3.4715720243,-2.3171814715,0.1732409049
C,1.2709149665,-3.157184255,-0.3209881267
C,4.0365910772,-3.4157389433,-0.4723902157
H,4.1134341462,-1.5602227653,0.6152582755
C,1.8323263704,-4.2573482369,-0.9629385358
H,0.1885449867,-3.065520657,-0.2639001958
C,3.2184751535,-4.3886517062,-1.0410480664
H,5.1164135698,-3.5110049459,-0.5283232082
H,1.1880609719,-5.0134422061,-1.4004727132
H,3.6566030384,-5.246986502,-1.5403592536
H,-3.2348126186,0.1125752548,-0.4105625187

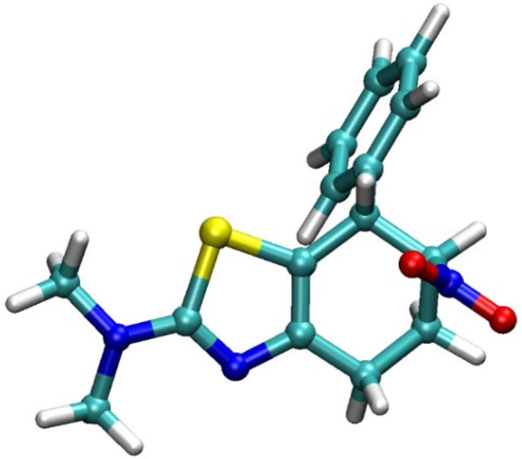

3ba-*endoA*
C,-1.2711359046,0.0425278112,0.455628883
C,-1.0521401071,-1.0758668071,-0.2805153199
H,0.3186795295,-1.8582180236,-1.7146290306
C,0.3215392995,-1.5321377023,-0.6704639163
C,1.3475812702,-0.4126512275,-0.4768849071
H,0.5946258055,-2.4117912563,-0.0732915848
H,1.2359434011,0.3389230315,-1.2650704729
N,-2.162529667,-1.8061258534,-0.6501183007

H,2.4047572085,2.188454825,-0.3917358698
C,1.0224405332,0.3901895763,-0.334203313
H,0.4744136857,0.0734434032,-1.226599621
C,2.2269196695,-0.5056031644,-0.1339581353
C,2.4506257503,-1.5758093342,-1.0021204798
C,3.1258865204,-0.2934659586,0.9167218126
C,3.5459880766,-2.4195328433,-0.8259953734
H,1.7616676517,-1.7489028226,-1.8241619805
C,4.2223105034,-1.1326575931,1.0949926024
H,2.9764642625,0.5400841608,1.5996223583
C,4.434476373,-2.2007506064,0.2236072357
H,3.7052714315,-3.2454650931,-1.5121922566
H,4.9136692856,-0.9510904376,1.9120308991
H,5.2885414581,-2.8560782612,0.3621744044
H,0.490682544,3.9203749467,1.7026383236

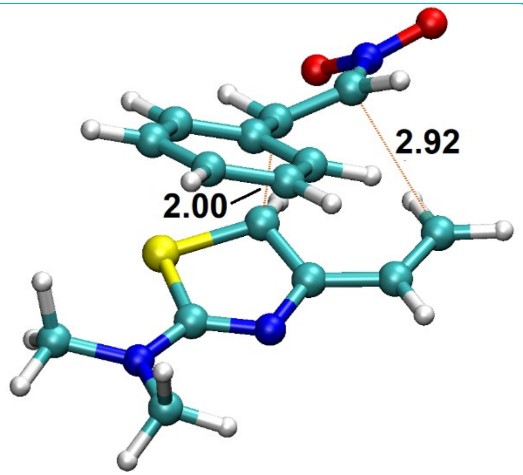

TS1ba-*exoA*
C,-0.6020267127,0.5926312552,-1.3714151802
C,-0.3644770228,-0.7603095181,-0.984831515
H,0.1021216709,1.1558569591,-1.9787362781
C,0.9341700035,-1.2524599746,-0.609867793
C,2.069890379,-0.5535343264,-0.7929505402
H,0.9478331075,-2.2135640956,-0.1038698626
H,2.1079222427,0.360520886,-1.374181648
N,-1.4510030238,-1.5389609558,-0.8105266852

S,-2.9289882489,-0.1708627472,-0.502117504
C,-2.846347928,-1.9231839885,-0.1290731665
O,2.9329295689,1.4620649956,1.2275565178
N,-3.9818362194,-2.5800824846,0.1916186669
C,-5.2935894077,-2.0137280162,-0.0726883773
H,-5.2988087032,-0.9404652352,0.1264877835
H,-5.6123160772,-2.1866229223,-1.1085205033
H,-6.0178970999,-2.4769955834,0.5999785577
C,-3.9088661216,-4.0228046639,0.3747639598
H,-2.9795724429,-4.2759785262,0.883373018
H,-4.757189447,-4.3424845758,0.9832444005
H,-3.9363232461,-4.5489362085,-0.587704894
O,1.3946494075,0.374961615,2.2862030212
N,1.8648119796,0.8681738715,1.2738875109
C,1.1332839166,0.6955194234,-0.0394513314
H,1.5256567992,1.5121363646,-0.6444994827
C,-0.3870058147,0.8388502102,0.1797266452
H,-0.5947823077,0.5032501789,1.1991181936
C,-0.8510237575,2.273476129,0.0373699852
C,-0.7627467489,2.9470956021,-1.1859457595
C,-1.3834991249,2.9472247532,1.1377133318
C,-1.1982741119,4.2633907604,-1.3052326639
H,-0.350263836,2.4439705153,-2.0575692683
C,-1.8183637912,4.266784099,1.0229499014
H,-1.458885154,2.4334559332,2.0920544177
C,-1.7272448304,4.9278679006,-0.1988869084
H,-1.1239577737,4.7722828015,-2.2611992691
H,-2.2283414445,4.7753404721,1.8897731903
H,-2.0647138243,5.9554024662,-0.2907810071
H,2.6062145181,-0.8747211011,-0.3491023839

S,-2.9856174544,0.2365910818,0.7258250103
C,-3.2503162488,-1.2530924465,-0.1808519962
O,2.5129430023,-1.1941894978,1.9780490948
N,-4.5010657823,-1.7672680013,-0.3375706036
C,-5.6565344419,-0.9325445481,-0.0682192054
H,-5.5675644902,-0.4652252932,0.9166293374
H,-5.7889725746,-0.1456728915,-0.8244242112
H,-6.5488949624,-1.5601660957,-0.0556719048
C,-4.6620005037,-2.8515237915,-1.2938153649
H,-3.8488621003,-3.5664996207,-1.1725741849
H,-5.6130222579,-3.3504457609,-1.0975343168
H,-4.6530220532,-2.4858554565,-2.3297787009
O,0.587637713,-0.7898538774,2.8656809382
N,1.4263682792,-0.6341156219,1.9952618255
C,1.1550712895,0.3304203319,0.847026582
H,1.9392445024,1.081591069,0.9704063443
C,-0.2305177445,0.991075699,0.9735722864
H,-0.4052769979,1.1804335561,2.0372445943
C,-0.2048169143,2.3430200024,0.2630606569
C,-0.6304531947,2.489961303,-1.058065139
C,0.2908825942,3.4612225688,0.9405305065
C,-0.5565537931,3.7288143533,-1.6926271195
H,-1.0332076205,1.6316165484,-1.5884567871
C,0.367189815,4.6999006156,0.3093676824
H,0.6176016346,3.3614910534,1.9733200506
C,-0.0561622253,4.8363978112,-1.01216497
H,-0.8955865737,3.8282669961,-2.7191365159
H,0.7529598118,5.5586733031,0.8498614299
H,-0.0011979083,5.8016816335,-1.5056118639
H,2.3656959029,-0.8003737462,-0.5358206658

S,-2.2946926997,0.6889057069,-1.8851699705
C,-2.553232856,-0.909401065,-1.1994801859
O,2.9780967541,2.7735926741,0.1495311344
N,-3.7777319684,-1.4271198815,-1.0805732205
C,-4.9501318257,-0.6114308812,-1.353938995
H,-5.0971266392,0.1423537943,-0.5708482576
H,-4.8574380098,-0.1120693872,-2.3222983844
H,-5.8249156943,-1.2602825488,-1.3918151862
C,-3.9720541625,-2.67599577,-0.3519827473
H,-3.0513886933,-3.2548909122,-0.3736806234
H,-4.240394338,-2.4690985987,0.6900332491
H,-4.773483599,-3.2432325179,-0.8293621304
O,1.3526354861,3.0212534162,-1.291218384
N,1.8001973568,2.5683714444,-0.2075710126
C,0.9803515284,1.7917850022,0.580479179
H,1.4573621453,1.3660826496,1.4486484563
C,-0.3872807847,1.6655793682,0.2480627295
H,-0.7883839261,2.5401442686,-0.260342592
C,-1.3113124619,1.0739828435,1.2552310044
C,-2.6153620775,1.5683084012,1.3680681736
C,-0.9290114221,0.0006093955,2.0692743628
C,-3.5168506981,1.0058523415,2.2690927407
H,-2.9242752068,2.4035756137,0.746508134
C,-1.8271617831,-0.5628369536,2.9692255754
H,0.0728755528,-0.4096998182,1.9862802962
C,-3.1256899653,-0.0644778215,3.0700099662
H,-4.522008459,1.408291339,2.348739916
H,-1.5144347841,-1.3965872169,3.5898022442
H,-3.8252595269,-0.5048101821,3.7738220371
H,3.0178155438,-0.9274016738,-0.4213336569

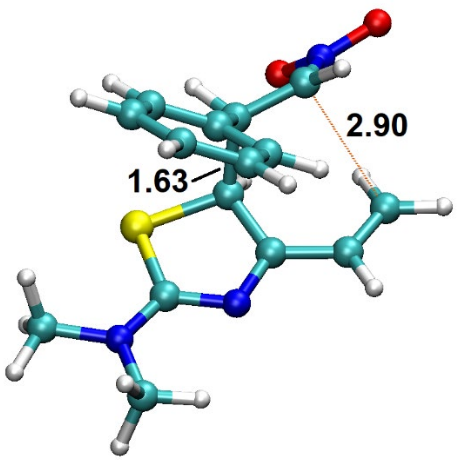

INTba-*exoA*
C,0.5067881178,0.3149388518,-1.0925017965
C,0.7495486519,-1.1275394929,-0.8420030541
H,1.1726036554,0.7686275159,-1.8338087515
C,2.0533603623,-1.6310774236,-0.5217476682
C,3.1764934631,-0.9115316574,-0.7174428384
H,2.0886785725,-2.6224295272,-0.0800168762
H,3.1902342365,0.0251524295,-1.2620620449
N,-0.2997826532,-1.9326794928,-0.8155255527
S,-1.2095935446,0.3784787291,-1.6761556894
C,-1.4217884473,-1.2843410381,-1.1851343847
O,4.1193744813,2.3832519395,0.1269150701
N,-2.6058626866,-1.8679647193,-1.1980448164
C,-3.8007767801,-1.1116753028,-1.5568186553
H,-4.0233513083,-0.3586630517,-0.7944570236
H,-3.6704885808,-0.6218543528,-2.5259542284
H,-4.6388627697,-1.8030600414,-1.6304146744
C,-2.7825240668,-3.25083232,-0.75836427
H,-1.8105414745,-3.6926627248,-0.5555552975
H,-3.3889510571,-3.2650010256,0.1512788246
H,-3.2909471149,-3.8141186923,-1.5441610392
O,2.5013183974,2.4673271661,-1.3666503402
N,2.9297465119,2.1279015277,-0.211982194
C,2.1131237847,1.4411552388,0.6038003792
H,2.5225982361,1.1604398035,1.5613302078
C,0.6977137827,1.2549073399,0.2266900209

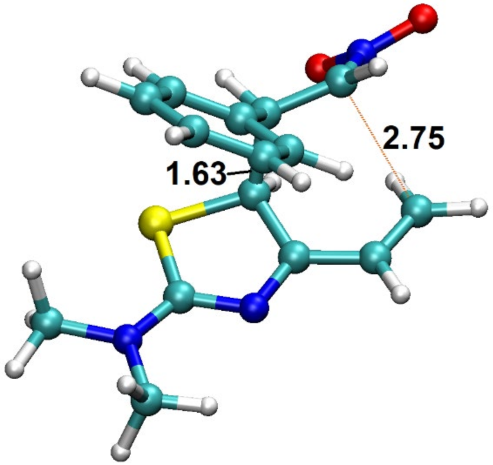

TS2ba-*exoA*
C,0.6107287895,-1.077019798,-0.4370101006
C,0.1451225865,-1.3063233411,0.9530429212
H,1.3811116309,-1.7727158502,-0.7825335486
C,1.0727209702,-1.3655250932,2.0327747604
C,2.4027359456,-1.5039426353,1.8193154617
H,0.6723169727,-1.2172134295,3.0310799702
H,2.8133938121,-1.8234412667,0.8691611403
N,-1.1650463097,-1.3053117313,1.1711311197
S,-0.8849334424,-1.2572952207,-1.4432173542
C,-1.8496370052,-1.2467804609,0.0182579311
O,4.831260557,0.0494322042,-0.133162385
N,-3.1706151491,-1.2020776854,-0.0272687871
C,-3.8663978935,-1.081097109,-1.3031835035
H,-3.6912647643,-0.0964755919,-1.7481457233
H,-3.5367845394,-1.8584324781,-1.9982831034
H,-4.9338724434,-1.2077988442,-1.128286789
C,-3.9739921733,-1.1578677392,1.1923112356
H,-3.3272053133,-1.2897074316,2.0557909948
H,-4.4802252223,-0.1907769516,1.2583019161
H,-4.7184800163,-1.9567994301,1.1607249984
O,3.4284244189,-1.039900272,-1.4312514162
N,3.6601212868,-0.1499526536,-0.5514790703
C,2.6159517857,0.5243994518,-0.0241798936
H,2.8629388788,1.2757695035,0.7093412619
C,1.2853560761,0.3968119484,-0.6515674333

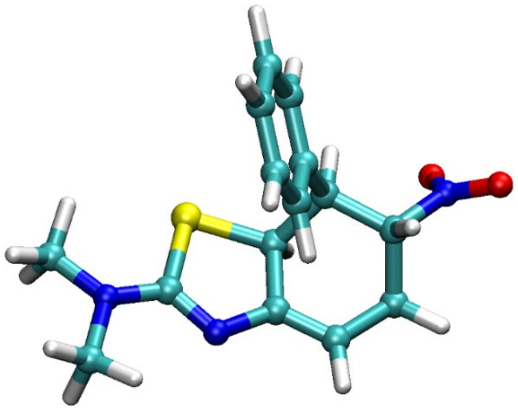

3’ba-*exoA*
C,-0.8328168279,-0.3742821866,-1.3013601266
C,-0.5879390035,-1.4192310023,-0.2208703341
H,-0.3916584846,-0.7022369736,-2.248085129
C,0.6838744853,-1.6103173873,0.1590678281
C,1.7330991266,-0.7709208061,-0.5137979009
H,0.9478462956,-2.3016090421,0.9507897929
H,1.8715016792,-1.0668427886,-1.5630463931
N,-1.7163952999,-2.0128102451,0.3132226109
S,-2.6354409733,-0.3361952689,-1.5188384887
C,-2.8090705422,-1.5379639064,-0.2019538829
O,2.0956370114,1.4255350962,-2.5696171424
N,-4.0447472293,-1.8860012005,0.2091989577
C,-5.2346540016,-1.5256205803,-0.5417660035
H,-5.1300188033,-0.5285099636,-0.9735295512
H,-5.4427619424,-2.2446807971,-1.3438632136
H,-6.0866644533,-1.503401632,0.1405980444
C,-4.1831801125,-2.9536691516,1.1888092474
H,-3.3179614099,-2.9519964266,1.8494682353
H,-5.0895885039,-2.7788137296,1.7727710465
H,-4.2537007826,-3.9338306267,0.7010957241
O,3.1752686072,2.0658353956,-0.8087252319
N,2.2563959441,1.4781223554,-1.3584890753
C,1.2970243502,0.7159966305,-0.480290965
H,1.4760329199,1.1181216209,0.5153127848
C,-0.1689235877,0.9817375636,-0.9225788886

H,0.3087385394,2.2095831546,-0.1449532716
C,-0.1623819148,0.7724332235,1.3742130934
C,-1.3780192465,1.4040525777,1.6513482118
C,0.2165208716,-0.3195493871,2.1623308045
C,-2.2002405876,0.9535222967,2.6821525743
H,-1.6808019828,2.2624021078,1.0580057566
C,-0.603162521,-0.7754830364,3.1912584228
H,1.1624460931,-0.8192868565,1.9734908653
C,-1.81706091,-0.1422147894,3.4524341458
H,-3.1366486635,1.4636915314,2.8864281071
H,-0.2914459198,-1.6253424079,3.7903792745
H,-2.4547351561,-0.4947275529,4.2570469953
H,4.1396706285,-1.2990865406,-0.402767287

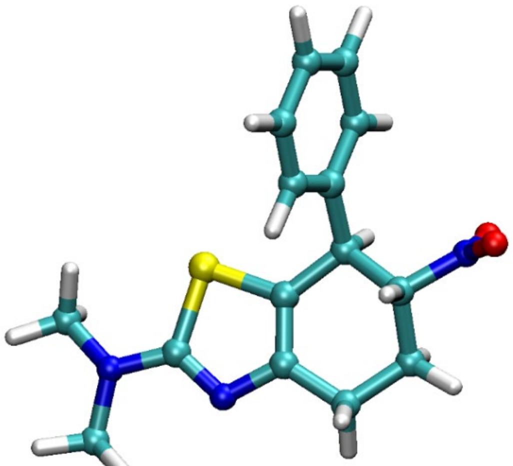

3ba-*exoA*
C,-1.2342591565,-0.2064034492,-0.3158509208
C,-0.9265198816,-1.4749843872,0.0575660939
H,0.5908769122,-2.9452007167,-0.2050045134
C,0.4864606802,-1.9475216895,0.2309977012
C,1.4663405772,-0.980559101,-0.43918218
H,0.7149967943,-2.0465143591,1.2995072024
H,1.4185572109,-1.0863857041,-1.5277814275
N,-1.9824416058,-2.3377627792,0.2597856641
S,-2.9688897508,-0.0426748854,-0.4505867397
C,-3.1194765005,-1.7278258008,0.0457773936

H,1.4147653359,0.4056462967,-1.7392381247
C,0.3337745548,1.5015718029,-0.2475545944
C,-0.3982699894,2.1841780147,-1.2231186842
C,0.1441041052,1.8499269607,1.093908226
C,-1.3040406783,3.1824357399,-0.8705655387
H,-0.2536553507,1.9352671728,-2.2707863438
C,-0.7625678243,2.8442894888,1.4513820048
H,0.7092091467,1.3405554323,1.8691814316
C,-1.4928741222,3.5119605339,0.4698388327
H,-1.8570117458,3.7056466437,-1.6447258061
H,-0.8965938836,3.0979220273,2.4983194554
H,-2.1973156416,4.2896719949,0.7477904942
H,3.104985655,-1.4327482032,2.6431950437

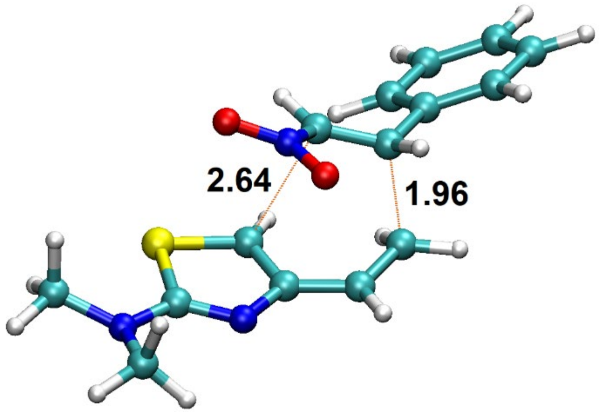

TS1ba-*endoB*
C,-1.6643660383,0.7155578655,-1.1521815087
C,-1.593724557,-0.6434713742,-0.8427392451
H,-0.8952383804,1.3377774379,-1.5817342607
C,-0.3765830212,-1.3018136237,-0.5927568803
C,0.8468889424,-0.6332700533,-0.6813938016
H,-0.4347232244,-2.2941875414,-0.1577923688
H,0.9566545975,0.1255324378,-1.4513368933
N,-2.8097390335,-1.2449994105,-0.5649153666
S,-3.2968271342,1.2536681941,-1.1255439868
C,-3.7877452764,-0.3969245691,-0.6639033735

H,-0.1366080586,1.5825634728,-1.8343897871
C,-0.9416700853,1.7649242798,0.1232630831
C,-1.5544368069,2.9675200425,-0.2346495507
C,-1.0806243504,1.3079198667,1.4387615195
C,-2.295835547,3.6974060185,0.6924572853
H,-1.4549112924,3.3365792424,-1.2521439296
C,-1.8252405183,2.0318373709,2.3655939924
H,-0.6118732631,0.3766531578,1.7427270978
C,-2.4364120763,3.229071354,1.9961435859
H,-2.7636939074,4.6300659171,0.3929047938
H,-1.9277930864,1.6587680225,3.3799287174
H,-3.0147585228,3.7933390414,2.7210925265
H,2.7020491031,-0.8658999833,-0.0193447295

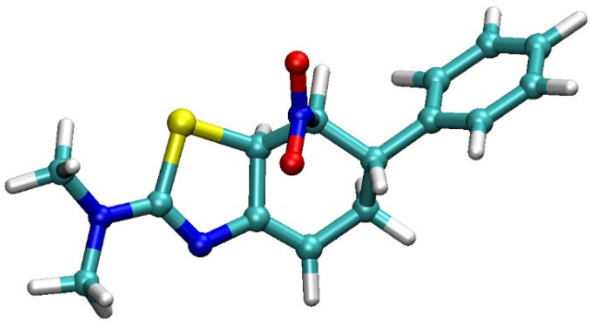

3'ba-*endoB*
C,1.5078438846,-0.5531432528,-0.931859725
C,1.5771226147,0.9418241293,-0.6431685515
H,1.1994328505,-0.7078038443,-1.9715028837
C,0.4227089694,1.6184060542,-0.6386272065
C,-0.8447053704,0.8314154296,-0.8243572947
H,0.3974148862,2.6832340931,-0.4358250016
H,-0.9719743286,0.5108389414,-1.8691139769
N,2.8452727928,1.4399195039,-0.3920906236
S,3.211945579,-1.1613117101,-0.7597417539
C,3.7511661437,0.5184064049,-0.406538984

O,2.270211939,1.5338214779,-1.7900972185
N,-4.34118823,-2.3058679667,0.2149729444
C,-5.5194810493,-1.6712889755,-0.3455587934
H,-5.5474494496,-0.611590057,-0.0777817414
H,-5.5620976226,-1.76302386,-1.440194901
H,-6.407622259,-2.1405949584,0.0809910679
C,-4.3768210741,-3.7521843888,0.3701303704
H,-3.5775833598,-4.0663615566,1.0402612794
H,-5.3399678059,-4.0346553048,0.7997298964
H,-4.2469734403,-4.266242529,-0.5922826688
O,2.9146820708,1.9016244062,0.2410414264
N,2.1784466148,1.3795869511,-0.5810133883
C,1.1034513485,0.4591269031,-0.0673850841
H,1.1280725014,0.5963214322,1.0152352979
C,-0.2610953987,0.8956297901,-0.6378070635
H,-0.1479302741,0.971697043,-1.7261553314
C,-0.6592074925,2.2633426482,-0.1079525163
C,-0.5603798699,3.384998465,-0.9320621715
C,-1.0823569011,2.4280862259,1.2147809799
C,-0.8790108623,4.652525151,-0.4462217145
H,-0.2278777844,3.2670194232,-1.9598224381
C,-1.4015594732,3.6914574435,1.7017624318
H,-1.1726582631,1.559680692,1.8628421513
C,-1.300267687,4.8080620598,0.8713570442
H,-0.7958375007,5.5156993539,-1.0990217502
H,-1.7315350685,3.805890493,2.7295487101
H,-1.5509335172,5.7934132238,1.2515247921
H,2.4904293993,-1.1978591738,-0.122710305

N,-5.094617653,-0.6941462248,-0.4594423108
C,-6.0592158682,0.3745710363,-0.2626944536
H,-5.9593111425,1.1327913236,-1.0440339993
H,-7.0630352641,-0.0452448147,-0.3425196392
H,-5.9506560273,0.8540099429,0.7190967043
C,-5.3908733744,-1.9795198386,0.1597127117
H,-5.1923774077,-1.9583887604,1.2388077958
H,-6.4425631118,-2.2146251037,-0.0135292511
H,-4.7695263206,-2.7517231364,-0.2921736934
C,0.9751957061,0.6498845386,0.7990355379
C,-0.2364076966,1.3398935009,0.9737824213
H,-0.4147235397,2.3776634986,0.7373431637
H,1.1519327962,-0.114897153,1.5500479524
N,-1.2381027342,0.7515601814,1.7517631331
O,-2.251627854,1.4174975181,2.0255695951
O,-1.0855687541,-0.4267172605,2.1198681277
C,2.1823169631,1.4307693971,0.4014216099
C,2.1149587761,2.4734933027,-0.5298569971
C,3.4250954657,1.100465944,0.9498755212
C,3.2589302187,3.177314905,-0.8926129367
H,1.1635948879,2.7412548417,-0.982439961
C,4.5716457351,1.8045931608,0.58887181
H,3.4924270125,0.2898063523,1.6701832766
C,4.4925558831,2.8454757204,-0.3333234109
H,3.1886583186,3.9835587544,-1.6158382382
H,5.5270123375,1.5374207058,1.0295459128
H,5.3849045606,3.3932869895,-0.6186231152
H,1.7506235423,-1.2146167552,-0.5214386816

N,5.0664666483,0.7501218776,-0.2051140387
C,5.9906574268,-0.3329313432,0.0898325172
H,5.9432755703,-0.6377547931,1.1426860453
H,5.7834979004,-1.2004962186,-0.5399339497
H,7.0042581204,0.0025332277,-0.1360076566
C,5.4565735526,2.0895564099,0.2148684517
H,6.5324609959,2.199544741,0.069021014
H,4.9313550573,2.8300875936,-0.3877648162
H,5.2144366047,2.264441046,1.2703414764
C,-0.8457607471,-0.4128146862,0.103020773
C,0.4299739891,-1.2773538277,-0.0917372785
H,0.1958228155,-2.238134205,-0.5478299517
H,-0.7962499488,-0.0158797697,1.1204717862
N,0.9863027664,-1.6576505326,1.2585777418
O,1.1071027106,-2.8498817295,1.495101783
O,1.2958218808,-0.7620847866,2.0269905813
C,-2.1084884904,-1.2371595136,-0.0314185233
C,-2.9776925256,-1.3757617608,1.0523022626
C,-2.4422168458,-1.8566945992,-1.2412912873
C,-4.1540764088,-2.1147996073,0.9343955517
H,-2.7302477074,-0.9038680526,1.9996194845
C,-3.6158782767,-2.5943143226,-1.3638365602
H,-1.78087777,-1.7638011628,-2.0999477695
C,-4.4770949371,-2.7251918924,-0.274511075
H,-4.8157905519,-2.2145341832,1.7891299452
H,-3.860221442,-3.0665844382,-2.3103335661
H,-5.3929441728,-3.2998982503,-0.3686995327
H,-1.7226158365,1.4338507602,-0.5794401771

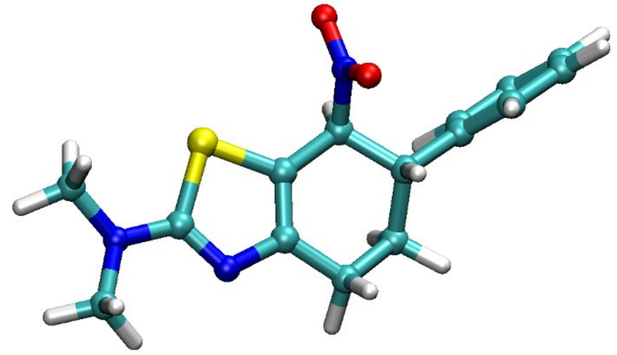

3ba-*endoB*

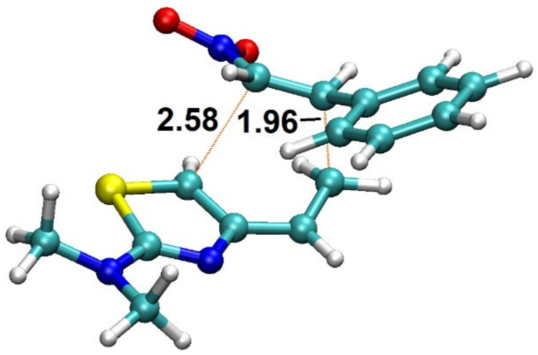

TS1ba-*exoB*

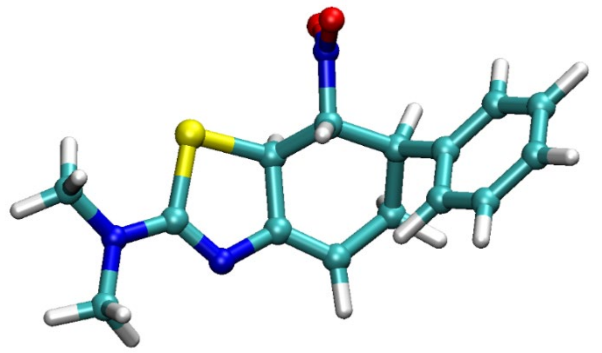

3’ba-*exoB*

C,1.6730799817,-0.4200046568,-0.1931999532
C,1.7311748424,0.9343247836,-0.0888083773
C,0.5023762639,1.7887142312,-0.0095121896
C,-0.7200102362,1.0088164972,-0.4995907048
H,0.3544763655,2.1190996479,1.0266931536
H,-0.6691146231,0.8980567985,-1.5897613423
N,2.9834929269,1.4976757913,-0.0911368337
S,3.2885985431,-1.0733419105,-0.3319979021
C,3.9015619532,0.5744825221,-0.2192315668
N,5.2360839438,0.8306150791,-0.2901236821
C,6.1844982222,-0.2572876047,-0.1538652711
H,6.2213000724,-0.6535366477,0.8710081428
H,5.9337050295,-1.072339985,-0.8393208285
H,7.1766208226,0.1084192818,-0.4239673314
C,5.6870873909,2.1649903592,0.0721986897
H,6.6581830613,2.3463418818,-0.3939738751
H,4.9684122513,2.8986888917,-0.2903954556
H,5.7825967651,2.2783864955,1.1607460448
C,-0.8016157953,-0.3727828968,0.1705318128
C,0.418137057,-1.2248379938,-0.227671288
H,0.2693952047,-1.7201205166,-1.1907877992
H,-0.7230386994,-0.2171383425,1.251892122
N,0.5419147041,-2.4029490132,0.7398792224
O,0.7651619538,-3.4939111667,0.2405344876
O,0.4393811829,-2.1867976623,1.9346467866
C,-2.0965966862,-1.1071121321,-0.1107361902
C,-2.8773699088,-1.5799013016,0.945760763
C,-2.5310526831,-1.3382592275,-1.4202897905
C,-4.0681937582,-2.2619187066,0.7035821511
H,-2.5432485097,-1.419717261,1.9672143408
C,-3.7198837127,-2.0180843176,-1.6662051623
H,-1.9372663147,-0.9852209448,-2.2597739898
C,-4.4935930129,-2.481775751,-0.6033295119
H,-4.6607849447,-2.6244313514,1.5376504315
H,-4.0425857894,-2.188298372,-2.6886868214
H,-5.4205018731,-3.0133839812,-0.7937037465
H,0.6572044705,2.690874758,-0.6087554715
H,-1.6399247715,1.5578823744,-0.2785254036

C,1.7367767762,-1.4171629734,-0.6533210087
C,1.2669169308,-0.2279439484,-1.2114686264
H,1.2499870216,-2.3819821617,-0.6617789257
C,-0.1007005999,0.0323244756,-1.4000489653
C,-1.0838022498,-0.8263632166,-0.9118368315
H,-0.3675728432,1.0343295814,-1.7227516753
H,-0.909589943,-1.9005656401,-0.9042014033
N,2.218470724,0.7723948565,-1.3565923695
S,3.434936218,-1.3164831037,-0.3725445513
C,3.392287095,0.3584421829,-0.976036863
N,4.524737876,1.1014842629,-1.0262073362
C,5.7129701315,0.6718711471,-0.3093252232
H,5.6144909526,0.8072039535,0.7760488131
H,5.9306765137,-0.3788043974,-0.5186102375
H,6.5631586104,1.2580370592,-0.660700498
C,4.3820873208,2.5231011291,-1.3060212579
H,3.6515742826,2.6662809846,-2.1014185625
H,4.0453981423,3.0752787659,-0.4185069614
H,5.3484715517,2.9144890419,-1.628021717
C,-0.9453229666,-0.6782258782,1.0388288955
H,-1.7867063488,-1.3576774988,1.1588647202
C,0.2825921816,-1.2072671185,1.4713859348
H,1.0704798888,-0.6403234924,1.9433476593
N,0.429386546,-2.5951815396,1.6166347844
O,1.4135313167,-3.0221803219,2.2357881098
O,-0.4032797931,-3.3477044143,1.0797529823
C,-1.244676725,0.74530848,1.3633484388
C,-0.2620263918,1.7398265845,1.2933049414
C,-2.5446027122,1.1065424552,1.7281626513
C,-0.5745214907,3.0628192793,1.5898031014
H,0.7490546855,1.4874120056,0.9849887902
C,-2.8581364558,2.4309723822,2.0255183993
H,-3.3178362201,0.3446540967,1.7817905606
C,-1.8730992563,3.4136387405,1.9582940259
H,0.1982415042,3.8226641246,1.5271115344
H,-3.8725959699,2.6937869843,2.3082535863
H,-2.1162573919,4.4463717466,2.1868997411
H,-2.119294802,-0.5485981054,-1.0897977267

C,-1.5798546807,0.399996232,-0.9341885098
C,-1.4538659145,-1.0855571686,-0.6032820522
H,-1.3460256401,0.6066502702,-1.9831348109
C,-0.2237030958,-1.6136948915,-0.5989070377
C,0.9326002066,-0.7247135055,-0.9661853045
H,-0.066838131,-2.6560706931,-0.3441762753
H,0.9476595756,-0.5721782068,-2.0540444408
N,-2.634907544,-1.7214548086,-0.2532427707
S,-3.3263447021,0.8082377159,-0.6320177075
C,-3.6447990122,-0.9130790592,-0.2280850445
N,-4.9090607659,-1.2924922671,0.0554266458
C,-5.9453983255,-0.3195298469,0.3596963637
H,-5.8979549792,0.013433193,1.4041760523
H,-5.8645256323,0.5508974083,-0.2942150759
H,-6.9195196254,-0.777851237,0.1799244692
C,-5.1249934492,-2.6539746057,0.5260069954
H,-6.166117255,-2.9261199133,0.3421052203
H,-4.4693100565,-3.3346967101,-0.0149272078
H,-4.9140231579,-2.7415169433,1.5992409477
C,0.8814305778,0.6805729811,-0.2898863111
H,1.3147770695,1.3911954273,-0.9999954417
C,-0.5854893717,1.1324517893,-0.0312951168
H,-0.8628806816,0.989592999,1.0129204542
N,-0.6618669783,2.622218639,-0.238767211
O,-0.6333572819,3.0389985635,-1.3888275545
O,-0.7271682874,3.3273199359,0.7542087899
C,1.6860580626,0.747342504,0.9933369854
C,2.6626438379,1.729098002,1.1649679263
C,1.462713011,-0.1687316688,2.0280703284
C,3.4054410457,1.7975039452,2.3437448915
H,2.8437828048,2.4498847085,0.3717840046
C,2.2038286499,-0.1049645627,3.2033323812
H,0.7028595442,-0.9375903357,1.9107226755
C,3.1788749829,0.8800078386,3.3654548022
H,4.1597771286,2.5691822719,2.461141967
H,2.0204231565,-0.8240431154,3.99572888
H,3.7553873565,0.9312859174,4.283690884
H,1.8833172682,-1.1968457231,-0.709937552

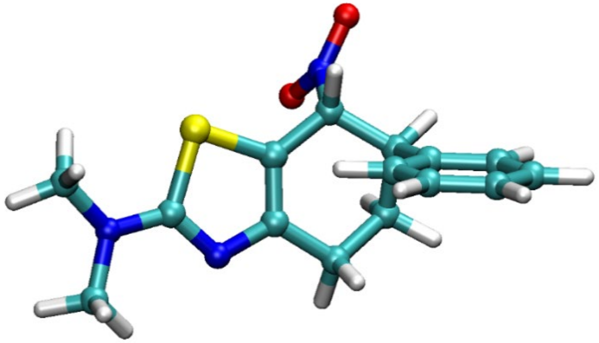

3ba-*exoB*
C,-1.5229540753,0.2958529468,0.5964868061
C,-1.3255656408,0.2950903743,-0.7500929679
C,0.0331823089,0.4283209573,-1.3637164824
C,1.0341758624,0.9853564359,-0.3506305135
H,0.3533707105,-0.5587921655,-1.7220331771
H,0.8403959901,2.0527814787,-0.2005368296
N,-2.4359017782,0.1060993112,-1.5335446545
S,-3.2183502889,0.0463487624,0.9459064443
C,-3.5107475222,-0.0136573456,-0.7908963231
N,-4.7672728425,-0.1454311396,-1.281950636
C,-5.8576235997,-0.5179570614,-0.4010856205
H,-5.8120284295,-1.5768688448,-0.1110816412
H,-5.8494144011,0.0982398082,0.502499494
H,-6.8017052065,-0.3362767105,-0.916779358
C,-4.930553677,-0.4136856738,-2.7025968168
H,-5.9030727872,-0.0340048105,-3.0234010607
H,-4.1437059141,0.0933873959,-3.2586010677
H,-4.8757600114,-1.4894290166,-2.9157897608
C,0.9537948842,0.2715327054,1.0151221435
H,1.6404499716,0.7787077042,1.7007973306
C,-0.4647116448,0.4088922442,1.6235212209
H,-0.5838192032,-0.2981975568,2.4472222552
N,-0.5108714703,1.7670568143,2.3417444746
O,-0.8762596167,2.7508617618,1.7239507202
O,-0.1297759945,1.7764424574,3.5036657951
C,1.4166171189,-1.1751272235,0.9306906547
C,2.786328335,-1.4340913488,1.053817953
C,0.5558058579,-2.251533299,0.6961939055
C,3.2865993145,-2.7278052706,0.9407523099

H,3.4693534964,-0.609841715,1.2450075174
C,1.0534940891,-3.549417647,0.5866612633
H,-0.5116673413,-2.084163593,0.5903039349
C,2.418854375,-3.7931581775,0.7059463226
H,4.3526633186,-2.9050870599,1.043654614
H,0.367748078,-4.3710456457,0.4039741244
H,2.8039110546,-4.8043254746,0.6198883997
H,-0.0272301244,1.0787813944,-2.2408665709
H,2.0534990642,0.8902982872,-0.7360978531

Series 1e + 2b

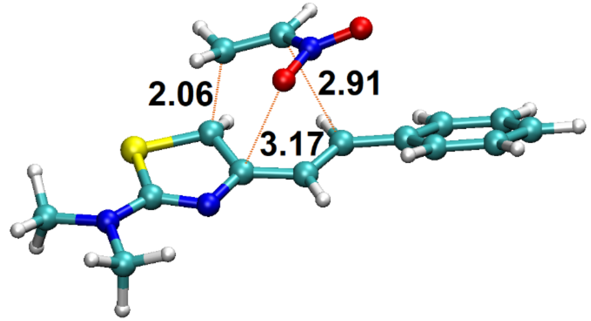

TS1eb-*endoA*
C,-1.1295957607,0.1266803043,-1.4935255715
C,-0.7591116741,-0.7620621195,-0.4529672606
H,-0.579353334,0.1933822672,-2.4235657202
C,0.5980037634,-1.0614825967,-0.1122963643
C,1.6593361012,-0.6260707566,-0.8320568462
H,0.7396271542,-1.60241236,0.8179300179
H,1.4939240269,-0.1776939324,-1.8086043066
N,-1.7728547736,-1.2374213227,0.317947489
S,-2.8874818139,0.0929172003,-1.632595092
C,-2.9462249714,-0.8545626672,-0.1432266334
O,2.239750151,2.0698310428,1.300687017
C,3.0630159299,-0.7833658861,-0.4447858395
C,3.447630345,-0.9466594503,0.8941871086
C,4.7884649107,-1.0992598131,1.2274282235
C,5.7665702428,-1.0839104487,0.233626545
C,5.3980664075,-0.9033662859,-1.0991126193
C,4.0573485695,-0.7460315934,-1.4328515183
H,2.6990332216,-0.9158450494,1.6791022072
H,5.0724805078,-1.2114655881,2.26891185
H,6.8133155482,-1.1977922863,0.4974742295
H,6.1560188476,-0.8801565316,-1.8757986444
H,3.7723633213,-0.6005915526,-2.4717799067
N,-4.1120842926,-1.129380953,0.4608742113
C,-5.3759959096,-0.8275805243,-0.1888534335
H,-5.5891467005,-1.5259253858,-1.0076050205
H,-6.1738673746,-0.9000682005,0.5506171334

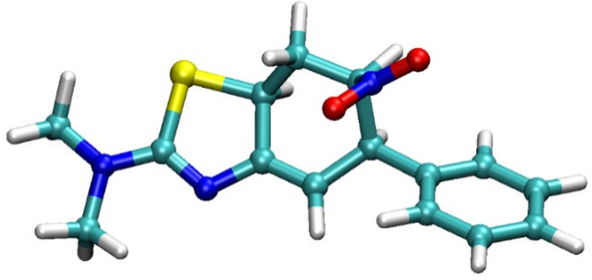

3'eb-*endoA*
C,-1.2883932151,0.8611025097,-0.9756348006
C,-1.0828916148,-0.5276214458,-0.3895786613
H,-1.0649443122,0.8666829437,-2.0487210661
C,0.1765383786,-0.9615139199,-0.2534853075
C,1.2858952547,-0.0715587829,-0.7486752729
H,0.3941095486,-1.9276039025,0.185954789
H,1.1942272637,0.021430175,-1.8416929626
N,-2.2315705044,-1.1914069692,0.0094790965
S,-3.0665219781,1.1942441309,-0.780419182
C,-3.2879110105,-0.4497055596,-0.0915618568
O,2.7616773906,2.2076539477,1.1420001136
C,2.6883681339,-0.5781809576,-0.4748638895
C,3.0153761718,-1.2141177535,0.7260040512
C,4.3186086094,-1.6418451479,0.9688788814
C,5.3132376715,-1.4405302146,0.0147420229
C,4.9973188677,-0.8064115109,-1.1852659703
C,3.6939287265,-0.379702223,-1.424732036
H,2.2517577727,-1.3680653557,1.48255602
H,4.5553284036,-2.1307092696,1.9086657066
H,6.3282073802,-1.7750603621,0.2047426518
H,5.7643431566,-0.6461598156,-1.9365534702
H,3.4540815621,0.1117454034,-2.3647523338
N,-4.5186787945,-0.8295191633,0.3126991412
C,-5.7107153735,-0.086852364,-0.0584249037
H,-5.5164386037,0.9872449129,-0.0491975291
H,-6.079068141,-0.3755417833,-1.0510695089

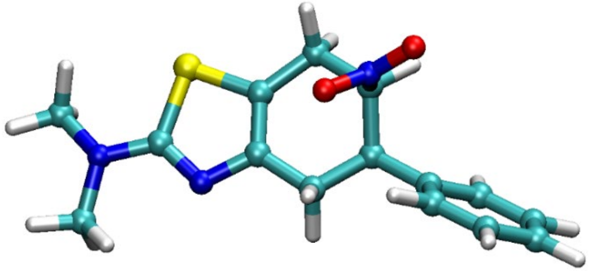

3eb-*endoA*
C,-1.3093499717,0.9563347253,-0.1066535716
C,-1.1312349867,-0.3823762677,-0.0024780788
H,0.2065376118,-1.9334362215,-0.5966984088
C,0.2236025735,-1.0161168147,0.0000251641
C,1.2818745896,-0.0682564763,-0.5851739272
H,0.4744603016,-1.3093602598,1.0243876589
H,1.0519483454,0.0421364267,-1.6528192331
N,-2.267693285,-1.1548265938,0.1275512701
S,-3.0148909613,1.3252544453,-0.0428312891
C,-3.3341423293,-0.40151685,0.1428823526
O,2.5602442082,2.1309874344,1.6503060917
C,2.701548359,-0.5941870241,-0.4849339273
C,3.2229185828,-1.0975194648,0.7115148233
C,4.5393373227,-1.5474035608,0.7789017319
C,5.3554290498,-1.5055802642,-0.3494572518
C,4.8466041502,-1.0079849162,-1.5470051455
C,3.5307829176,-0.5563175503,-1.609903108
H,2.606747562,-1.1354578563,1.6051581831
H,4.9275004652,-1.9311684121,1.7171172226
H,6.3808197437,-1.8571810118,-0.2950687938
H,5.4721941131,-0.9734235673,-2.4335572349
H,3.1401552868,-0.1701750272,-2.5484165368
N,-4.6037068836,-0.8672631626,0.3126277537
C,-5.728392395,-0.0092626088,-0.0081805028
H,-5.6165402538,0.9641226664,0.4776456435
H,-5.8432612014,0.1447468355,-1.0908338145

H,-5.3728049435,0.1941023539,-0.5790918692
C,-4.1272681949,-2.0094458332,1.6225467089
H,-4.9268178364,-1.6917027057,2.2946512955
H,-4.297684291,-3.0499878639,1.3229800167
H,-3.1712085063,-1.9416514441,2.1381473614
O,0.2327895418,1.3296464736,1.71265989
N,1.0785958167,1.8167703472,0.9413563862
C,0.7163406311,2.0916713123,-0.3827944628
H,1.5311384393,2.4401709082,-0.9990438413
C,-0.6072986766,1.9778717847,-0.7711582013
H,-0.8855162582,2.483391943,-1.690289096
H,-1.3602289156,1.9643168137,0.0106690465

H,-6.4910975396,-0.2876532252,0.6782162199
C,-4.7154620194,-2.2062402261,0.7435531978
H,-3.8069920461,-2.5706621122,1.2198297706
H,-5.5403564154,-2.2361338265,1.4588343127
H,-4.9537586039,-2.8570309453,-0.1073265714
O,1.1383298046,1.1044949874,2.0479744241
N,1.7039979242,1.5983479268,1.0847127523
C,1.0901679495,1.4145241889,-0.2802440866
H,1.6994356919,2.0564639026,-0.9148775871
C,-0.3824741178,1.8660078848,-0.2462796058
H,-0.4723451012,2.8615367496,-0.687183576
H,-0.7160172713,1.9222581726,0.7920440265

H,-6.6401488978,-0.4669173882,0.3788609692
C,-4.8051611242,-2.3031377739,0.1960906648
H,-4.0163453687,-2.8265159075,0.7355006189
H,-5.7715943541,-2.5567215699,0.636181086
H,-4.788631457,-2.6324331503,-0.8521727591
O,0.8696166109,0.9084353158,2.2160234316
N,1.5597515385,1.4811608188,1.3854062937
C,1.1641153117,1.3851819231,-0.0668447897
H,1.9097347337,2.0030178815,-0.566038798
C,-0.236232948,1.9864294327,-0.2672002181
H,-0.2567855449,2.4149557078,-1.2765895473
H,-0.3737663551,2.8244055467,0.4250824566

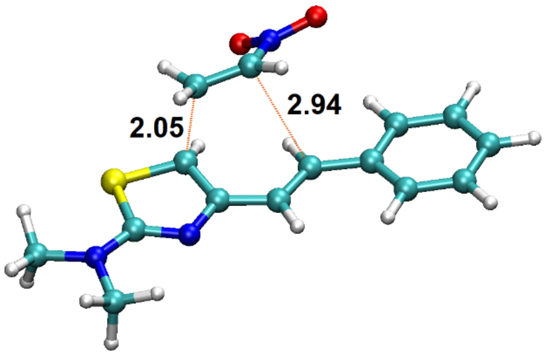

TS1eb-*exoA*
C,-1.3246628586,-0.0459604035,-1.3601260618
C,-0.9492051127,-0.6360129855,-0.1314645292
H,-0.788803433,-0.1989837562,-2.2901603458
C,0.4050998432,-0.8788508219,0.2638479373
C,1.460570355,-0.6949010767,-0.5658585644
H,0.5425304914,-1.1639427853,1.3023471159
H,1.2677855831,-0.4843394087,-1.6147100821
N,-1.9554221431,-0.8518126763,0.7627441077
S,-3.0852402848,-0.0614718573,-1.4520654863
C,-3.1324465567,-0.5836347328,0.2311081824
O,2.6147370786,2.2918965063,-2.1718632624
C,2.8781528888,-0.8113483791,-0.2234808747
C,3.333174757,-1.1290491165,1.0672351032
C,4.6927478934,-1.2153699104,1.3339850337
C,5.6254758775,-0.9807382624,0.320807375

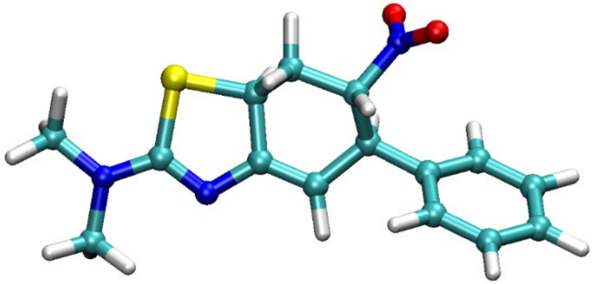

3’eb-*exoA*
C,-1.3245546164,0.848220191,-1.0093549312
C,-1.097842854,-0.5379019275,-0.4240221409
H,-1.114317327,0.8569031048,-2.0840052074
C,0.1680069288,-0.9644956651,-0.3156425485
C,1.2671804971,-0.0758489943,-0.8445899476
H,0.4081613372,-1.9310265755,0.1123992956
H,1.1669361704,-0.0005878934,-1.9362928551
N,-2.2333025333,-1.2116241934,-0.0056039126
S,-3.1012726814,1.1675998986,-0.7824685986
C,-3.2981126218,-0.4787658451,-0.0963866484
O,1.5861058578,2.4158863779,-2.3270973856
C,2.6647660788,-0.5695804434,-0.53578575
C,3.0175470442,-0.9559391568,0.7613622704
C,4.31030788,-1.3843987501,1.0468133189
C,5.271473092,-1.4306219203,0.0369121674

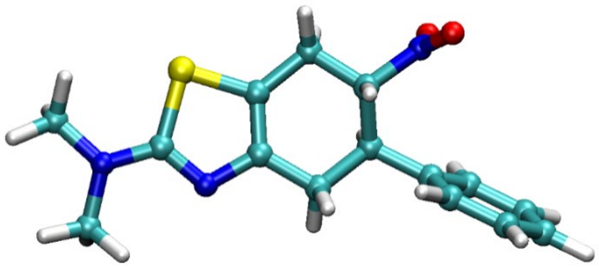

3eb-*exoA*
C,-1.3566898025,0.8272469499,0.3350493379
C,-1.2157930232,-0.4907494352,0.0505053524
H,0.094614061,-1.7955290503,-1.0144586708
C,0.1214666236,-1.1393344281,-0.1391815464
C,1.2417162654,-0.099976177,-0.3313120737
H,0.3353852507,-1.7835248228,0.7219656821
H,1.1177638574,0.3536323872,-1.3210749122
N,-2.3736059305,-1.234329627,-0.0492831326
S,-3.05251173,1.2134580561,0.4897879618
C,-3.4202750224,-0.4848976245,0.1740422448
O,2.4020479501,2.5489760099,-0.4686549703
C,2.6209793677,-0.7259315693,-0.2636981453
C,3.050967742,-1.4129913494,0.8767562846
C,4.3219492091,-1.9771644177,0.9305996324
C,5.1855609772,-1.8616172733,-0.1577587957

C,5.1882922559,-0.6607275153,-0.9612804446
C,3.8252413512,-0.5788295937,-1.2312883031
H,2.6250820504,-1.3092936214,1.8697099123
H,5.0291213199,-1.4623326499,2.3359717543
H,6.6877304588,-1.0436600532,0.5350692191
H,5.9052245597,-0.4680447803,-1.7525950299
H,3.4889601503,-0.3147963719,-2.2294723223
N,-4.2937034476,-0.6728208154,0.8992371972
C,-5.5622858164,-0.5246290442,0.2060908596
H,-6.3458341742,-0.3512458603,0.9443697152
H,-5.5344118916,0.3381714021,-0.4645980767
H,-5.8138343061,-1.4225542275,-0.3718508037
C,-4.316043591,-1.2621390414,2.2314759305
H,-5.0663780637,-0.7458322793,2.8341747107
H,-4.5656913384,-2.3285777158,2.1811521058
H,-3.33730818,-1.1485829397,2.6933499261
O,0.8887996938,1.5880053054,-3.3009351137
N,1.4122840756,1.993354568,-2.2421443597
C,0.6318908393,2.0730423802,-1.0874214448
H,1.1942641162,2.2470853854,-0.1835750968
C,-0.7405111734,1.91335362,-1.1734755843
H,-1.2124904405,2.1432661592,-2.1224540018
H,-1.3184765372,2.1565815955,-0.2870221879

C,4.9305094277,-1.0444330239,-1.256363903
C,3.6338835739,-0.6167435011,-1.5389424005
H,2.2744574449,-0.9240972523,1.5546187501
H,4.568608087,-1.6837323833,2.057895021
H,6.2795255436,-1.7658660721,0.2595830526
H,5.6713308244,-1.0752981387,-2.0490761289
H,3.3736688649,-0.3148573955,-2.5502004733
N,-4.5206558146,-0.8754290872,0.3162505293
C,-5.7222010052,-0.1537706083,-0.0696391568
H,-5.564572772,0.924554845,-0.0024212147
H,-6.0402822332,-0.4066679312,-1.0888973437
H,-6.5237177267,-0.4143472471,0.6236884185
C,-4.6890864263,-2.2601157031,0.7358176912
H,-3.7907882386,-2.5926872931,1.2534390612
H,-5.5451751712,-2.3206460603,1.410912335
H,-4.8624788451,-2.9169763143,-0.1257214585
O,2.8266809907,2.877087935,-0.6161999094
N,1.8909893391,2.3013302274,-1.147154889
C,1.0604060909,1.3719915971,-0.3011203697
H,1.4985585479,1.4560687377,0.6921762864
C,-0.4069023761,1.8528156867,-0.2981385019
H,-0.4855681191,2.8406369315,-0.758133125
H,-0.7338335994,1.9447118734,0.7401338023

C,4.7699415881,-1.1752111838,-1.2956165595
C,3.4968374257,-0.6099970623,-1.3447099994
H,2.3928093375,-1.5093039116,1.7366087491
H,4.6389740242,-2.505593028,1.8242806659
H,6.1765770156,-2.3023429328,-0.1165121101
H,5.4380034738,-1.0731722745,-2.1450666056
H,3.1823562627,-0.0648462266,-2.2304211432
N,-4.7019066285,-0.9453298682,0.2022048251
C,-5.8053538851,-0.0065440928,0.1247455789
H,-5.658534879,0.8162568703,0.8295186111
H,-5.9297603972,0.4085447339,-0.8855074257
H,-6.7248144062,-0.5214576181,0.4082297524
C,-4.9374579542,-2.2887712733,-0.3046567904
H,-4.1635869474,-2.9593544396,0.0674773017
H,-5.9115416431,-2.6295992922,0.051584838
H,-4.9240061919,-2.31579581,-1.402819023
O,2.8932225029,2.166431509,1.6027513809
N,2.2131389262,1.9943541339,0.6035805186
C,1.0586405789,1.0315685383,0.6999150697
H,1.114278528,0.6433087208,1.718162693
C,-0.2418251967,1.8150772085,0.4845211736
H,-0.1409981477,2.4402264048,-0.4101479816
H,-0.4080150721,2.480406376,1.3381939214

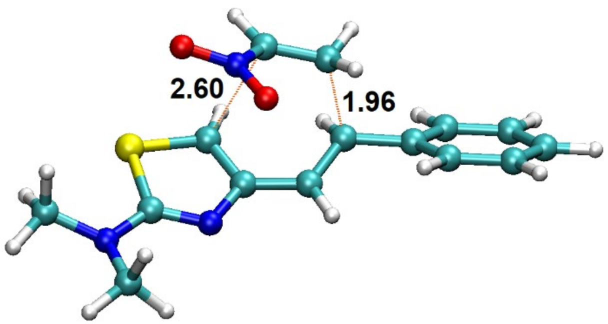

TS1eb-*endoB*
C,-1.1468968666,0.186252614,-1.523579418
C,-0.7601453067,-0.9326062194,-0.7809258864
H,-0.5449946453,0.7736407547,-2.1990521889
C,0.5681737992,-1.1892554759,-0.4167144433

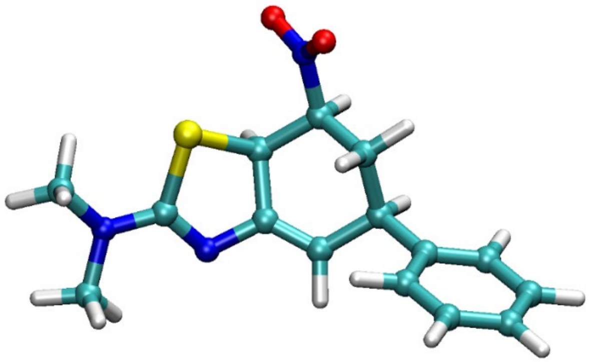

3’eb-*endoB*
C,1.1005124259,-1.123366066,-0.6342589308
C,0.5825483043,0.2855669904,-0.8415938903
H,1.5941793949,-1.4568775709,-1.5515137829
C,-0.7145005814,0.5766547284,-0.9882755415

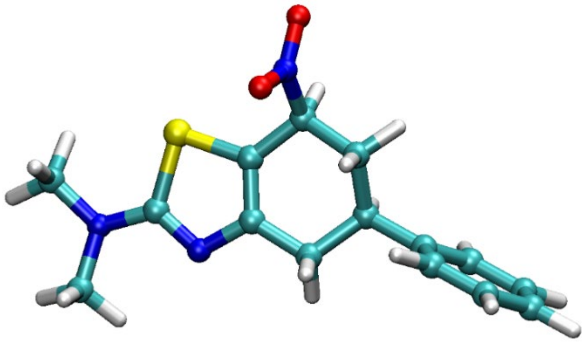

3eb-*endoB*
C,-1.0251850095,0.547718933,0.206405019
C,-0.6806739924,-0.7378685001,-0.0725990928
C,0.7445051942,-1.1890695191,-0.1805374779
C,1.6740227191,0.0112307221,-0.4273445488

C,1.6211690804,-0.3313193034,-0.7684795097
H,0.732957347,-1.9987177085,0.2878554006
H,1.5241997735,0.1629466864,-1.7336117879
N,-1.8053456899,-1.6681290089,-0.2426940117
S,-2.8608265583,0.3055252285,-1.5802130269
C,-2.9537878977,-1.1536924443,-0.5594185965
C,3.0283898964,-0.6946458969,-0.4403401896
C,3.3713244359,-1.2494358256,0.7982564392
C,4.6939343926,-1.5718671609,1.0871990716
C,5.6965296221,-1.3349367593,0.1472228696
C,5.3672510556,-0.76917592,-1.0824464282
C,4.0426811464,-0.4480229523,-1.3704429773
H,2.6024822051,-1.4210773486,1.5472640754
H,4.9426441887,-2.0034150935,2.0516927154
H,6.7283223668,-1.5832958279,0.3744655057
H,6.1408377947,-0.5775613507,-1.8195066959
H,3.7921865778,-0.0078981608,-2.3324277829
N,-4.157497206,-1.6566378829,-0.187139667
C,-5.3467219838,-0.8249954416,-0.2715105784
H,-5.3634976655,-0.0509350316,0.5068436001
H,-5.4142845097,-0.3466502846,-1.2520247329
H,-6.2255482626,-1.4614206438,-0.1576969648
C,-4.149083811,-2.6930354735,0.8369838625
H,-5.1223018501,-3.1871441977,0.8381202301
H,-3.3748473005,-3.4252668704,0.6103247204
H,-3.9530683989,-2.2716527029,1.831162143
C,1.3155417565,1.318753382,0.2436320579
H,2.090819597,1.8930688366,-0.2557347461
C,0.01959477,1.8230614232,0.1259093645
H,-0.290309034,2.6211708369,-0.5309137812
H,1.5930204775,0.9250080498,1.2154376646
N,-0.9437409678,1.4761444587,1.0908939321
O,-2.0415581033,2.0542235254,1.0603675072
O,-0.6693063455,0.5960369997,1.9206978037
C,-1.7933895454,-0.4564168643,-0.7625004843
H,-1.0027247532,1.6056836527,-1.1805087769
H,-2.0297202941,-0.9782960313,-1.7015365272
N,1.6065564945,1.235283535,-0.7868583861
S,2.4640744289,-0.8726664315,0.573016074
C,2.5948532923,0.8031430342,-0.0705389714
C,-3.080912935,0.1702040177,-0.2596498977
C,-3.0683574703,1.0495753627,0.8283510522
C,-4.2514402429,1.6032805034,1.3088566716
C,-5.4689052754,1.2878076973,0.7056357693
C,-5.4920652171,0.4149171996,-0.3790528213
C,-4.3043869937,-0.1390915832,-0.8559254836
H,-2.123065891,1.3054602652,1.3005068643
H,-4.2235304202,2.2838359054,2.1544174396
H,-6.3911954656,1.7223359435,1.0782552975
H,-6.4338186427,0.1668051153,-0.858879123
H,-4.3300049023,-0.8172915968,-1.7053476141
N,3.7023986388,1.5268660413,0.2056545592
C,4.6565286604,1.1068961455,1.2176529953
H,4.3416204075,1.4014943375,2.2268491736
H,4.7937414095,0.0242184834,1.1875340689
H,5.6220792251,1.5695906294,1.0038656639
C,3.7029114425,2.9365734899,-0.159389397
H,4.7371959436,3.2785510796,-0.2313879006
H,3.211486017,3.0639667442,-1.1229289826
H,3.1740831946,3.5417951441,0.5877688855
C,-1.2811026276,-1.5024955185,0.2512502711
H,-2.0369692341,-2.2748492528,0.4129281434
C,-0.0141355902,-2.1319798186,-0.2912559043
H,-0.2454168164,-2.6785779356,-1.210409013
H,-1.0868831705,-1.0146808323,1.2108983753
N,0.5232761815,-3.2222979302,0.6105030362
O,1.4817905518,-3.8518492469,0.185645387
O,-0.0192479444,-3.4222393671,1.6819777005
H,1.0331398355,-1.7123623312,0.7397397935
H,1.4564855021,0.3955420642,-1.4322973172
N,-1.712393971,-1.6174382925,-0.29186591
S,-2.7651398977,0.7135198565,0.175028254
C,-2.8677363047,-1.0051035747,-0.2033213506
C,3.1375699947,-0.3805427888,-0.3938410704
C,3.7377092072,-0.8467721549,0.7815005587
C,5.0842133656,-1.1993354757,0.8037588711
C,5.8555964247,-1.0949264932,-0.3533617909
C,5.2685018741,-0.6373167063,-1.5301448578
C,3.9202582707,-0.2826865227,-1.5465823188
H,3.1516348518,-0.9369596193,1.692514609
H,5.533178343,-1.5542559215,1.7263368938
H,6.9061806634,-1.3671006932,-0.3359183863
H,5.8592825023,-0.5535712827,-2.4371955359
H,3.4693214362,0.0738971938,-2.4692563953
N,-4.0685899557,-1.60457787,-0.4037227502
C,-5.2910135769,-0.9166932319,-0.0314289124
H,-5.427875851,-0.8769246492,1.057971063
H,-5.2929669345,0.1029380621,-0.4268008326
H,-6.1375208234,-1.4441253817,-0.4737295516
C,-4.1068074783,-3.0590748271,-0.4448315555
H,-5.003626757,-3.3698216962,-0.9844766444
H,-3.2250170469,-3.430842541,-0.9647257883
H,-4.1269945619,-3.488063649,0.5657755199
C,1.3698057523,1.1296530652,0.5830071643
H,2.074141048,1.9571352949,0.4621054883
C,-0.0644112568,1.6602425782,0.4260368151
H,-0.1238478556,2.429753883,-0.3469766321
H,1.4807564827,0.7388346198,1.5998382648
N,-0.3954563795,2.4329798057,1.7025875759
O,-0.4590992052,3.6501238641,1.6109383999
O,-0.5534299141,1.8045000086,2.7346136994
H,0.8353754346,-1.9128061293,-0.9960877502

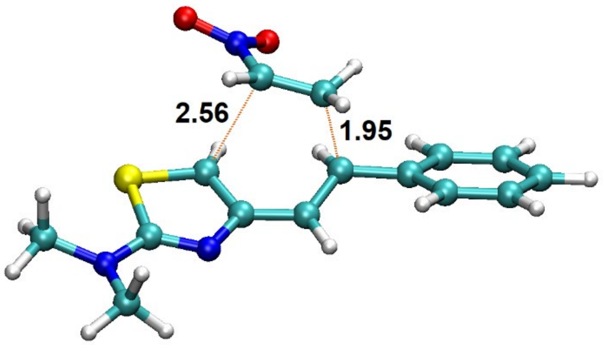

TS1eb-*exoB*
C,-0.9873409766,0.6552355749,-1.2295390822
C,-0.5819858691,-0.5248104282,-0.6029641714
H,-0.4103898641,1.2717010786,-1.904560333
C,0.7614738396,-0.8256170435,-0.3439985264
C,1.7920658479,0.0909299454,-0.5915599415
H,0.9592316731,-1.727725941,0.2284537223
H,1.6631044584,0.7567935578,-1.4448443003
N,-1.6092899333,-1.2945419448,-0.0721435802
S,-2.7067820326,0.7735850965,-1.2260276218
C,-2.7689503316,-0.7611051876,-0.3228411758
C,3.2139836771,-0.2817931685,-0.3522443205
C,3.6009650349,-1.0563846214,0.7474417463
C,4.9404046368,-1.3709228838,0.9570389779
C,5.9165723706,-0.9075946042,0.0755540836
C,5.5433038914,-0.1231863017,-1.0136044208
C,4.2024614362,0.1906141983,-1.2218150471
H,2.8551064819,-1.4098129286,1.4544539826
H,5.2238539008,-1.9718118073,1.8154951581
H,6.9614056195,-1.1491791981,0.2425858217
H,6.2959927764,0.2480726772,-1.7019955922
H,3.9170064138,0.8066812739,-2.0703990854
N,-3.9598091161,-1.2996543014,0.0377883814
C,-5.1710347179,-0.4992981327,-0.0110647005
H,-6.0322701012,-1.1642994163,0.0675742489
H,-5.2166295589,0.2353949987,0.8039639423
H,-5.2483128466,0.0251080779,-0.9669595923
C,-3.9351851621,-2.4269458624,0.9586012406
H,-3.1379162648,-3.1127154557,0.6738567226

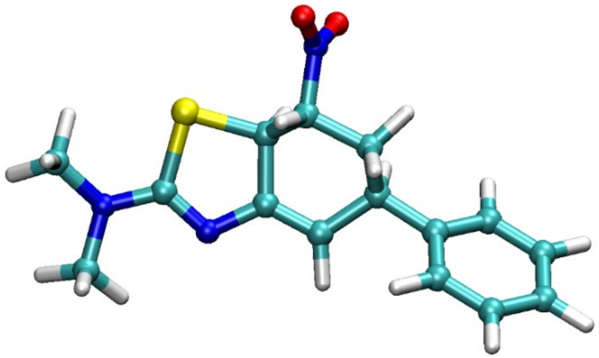

3'eb-*exoB*
C,-0.9017654483,0.5366158165,-0.9209728328
C,-0.5580272582,-0.8906934448,-0.5074870415
H,-0.6223681923,0.7138616297,-1.964681432
C,0.7374089311,-1.2143024113,-0.4092606556
C,1.7598231489,-0.1429073232,-0.7017485164
H,1.0451461591,-2.2174891981,-0.1346451126
H,1.7350349005,0.0896629778,-1.7764893508
N,-1.6433791227,-1.7130104709,-0.2503280194
S,-2.7114270284,0.6542667941,-0.7754079792
C,-2.7735946679,-1.091465911,-0.3521394326
C,3.1797168057,-0.5421470102,-0.3578965068
C,3.4858259782,-1.1018217771,0.8871883973
C,4.7966090542,-1.4386844158,1.2137992851
C,5.8251369084,-1.2191371684,0.2978375661
C,5.5315383744,-0.6613500506,-0.9440661273
C,4.2172748207,-0.3259938563,-1.2666907971
H,2.6909378432,-1.278891544,1.607742478
H,5.0159165229,-1.8734848749,2.1842361619
H,6.8474736023,-1.4818970025,0.5508587195
H,6.3237677659,-0.4883235135,-1.6658944392
H,3.994809606,0.1063666416,-2.2390783225
N,-3.9775018821,-1.6782427379,-0.1850602454
C,-5.1961172788,-0.8994538473,-0.0436434096
H,-5.3438275185,-0.5556903971,0.9877970852
H,-5.1826375589,-0.0343423723,-0.7092516421
H,-6.0449766566,-1.522695851,-0.3311033725
C,-4.0167411672,-3.0503402447,0.3014613738
H,-4.9422599965,-3.5181494568,-0.0409093345

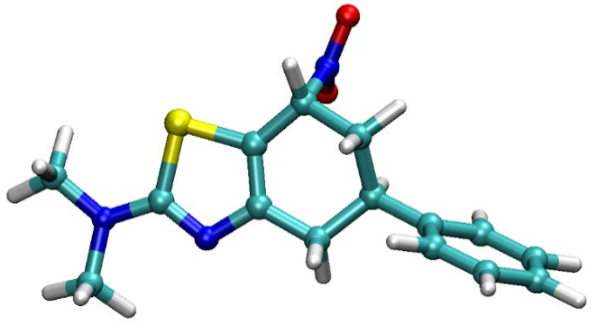

3eb-*exoB*
C,-1.1010185766,0.5580851408,0.4497981669
C,-0.7832811898,-0.693733165,0.0223813339
C,0.6284921256,-1.1605886523,-0.1618687921
C,1.6167460654,0.0177088144,-0.1897568698
H,0.8753226,-1.8537267559,0.6525337103
H,1.4876140161,0.5297825599,-1.1501288103
N,-1.8312688182,-1.5509820268,-0.2013789645
S,-2.8372701745,0.7140825092,0.5848592089
C,-2.9754524463,-0.9513827702,0.0261262869
C,3.0592016803,-0.4394039317,-0.1033328604
C,3.5312921314,-1.136501553,1.014775284
C,4.8608172966,-1.5405918593,1.0963681326
C,5.7449645193,-1.2539103199,0.0568434425
C,5.2874414434,-0.5613739126,-1.0612725897
C,3.9547751248,-0.1579772807,-1.1374432497
H,2.8556058234,-1.3702150288,1.8338700692
H,5.2074428439,-2.0796974976,1.9727723145
H,6.781830953,-1.5689552774,0.1197526469
H,5.9674381303,-0.3317853662,-1.8758957642
H,3.6059851193,0.3839481816,-2.0128298558
N,-4.1870918605,-1.5299374498,-0.1642208725
C,-5.3812816241,-0.9034713156,0.3709875589
H,-5.4491839685,-1.0042478964,1.4630991342
H,-5.40591857,0.1582362754,0.1100151193
H,-6.2565710462,-1.3738162446,-0.079195046
C,-4.2244532353,-2.9657195467,-0.4004780718
H,-5.1837062117,-3.2183477393,-0.8559432744
H,-3.4193572029,-3.2451771238,-1.0789516925

H,-3.7627900945,-2.0981597085,1.9918614476
H,-4.8938444887,-2.9454597555,0.9020428919
C,1.4336539253,1.5293585786,0.6826414787
H,2.3199237451,2.0795208735,0.3798633638
C,0.2016464599,2.153255435,0.4687920383
H,-0.662964056,2.0661109448,1.1087970539
H,1.5333068391,0.9939977881,1.6212744217
N,0.1015835765,3.1903742319,-0.4772621775
O,-0.9084519729,3.9053880183,-0.4646903722
O,1.0048438924,3.3180433518,-1.3216994128

H,-3.1634649707,-3.599756884,-0.0928116519
H,-3.980575505,-3.0823758101,1.3976548864
C,1.3847147249,1.1607125452,0.0527906051
H,1.9897240773,1.9847764444,-0.3347883777
C,-0.1202329966,1.5152946997,-0.0349160966
H,-0.5745306039,1.5670457768,0.9532833185
H,1.6381159073,1.0392466872,1.1085114062
N,-0.2621194618,2.9138141688,-0.5826149624
O,0.0304763626,3.0976341618,-1.7551258701
O,-0.6470600792,3.7840612801,0.1819094755

H,-4.107764471,-3.5306982358,0.5337747917
C,1.2900358514,1.0272463039,0.9246567087
H,2.0218935774,1.8398229108,0.9358239481
C,-0.1253643182,1.613652688,0.7943162418
H,-0.3841959548,2.1679829977,1.6980495356
H,1.3387721885,0.5370634882,1.9030374327
N,-0.0697025157,2.7216798878,-0.2703563707
O,-0.2751700344,2.4346380804,-1.4361964779
O,0.2261647766,3.8385232918,0.1298733679
H,0.704452602,-1.7350428507,-1.0901337133

Series 1e + 2a

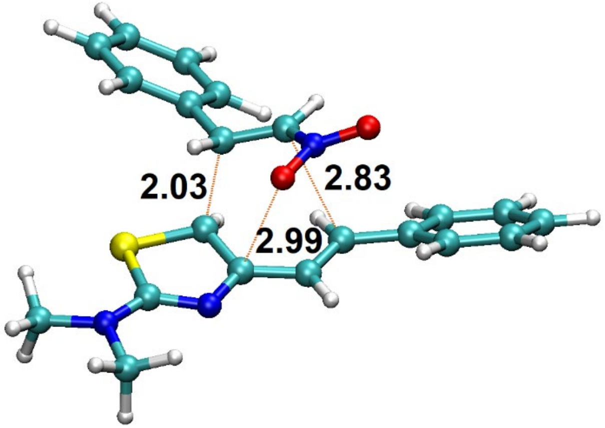

TS1ea-*endoA*
C,-1.5386018939,0.0442723671,-1.2298506886
C,-1.0644402434,-0.6520099788,-0.0784496984
H,-1.0686122302,-0.0926755812,-2.196218779
C,0.3236033605,-0.8353047553,0.1959528809
C,1.2974277371,-0.4630920186,-0.6731601331
H,0.5689113555,-1.2059003913,1.1860299187
H,1.026234866,-0.2077076037,-1.694280907
N,-2.0018208846,-1.0496564983,0.8177982439
S,-3.3015184149,-0.0894757798,-1.2369856925
C,-3.2191358531,-0.7949416868,0.3798706293
O,1.7103967242,2.4430299255,1.1515463772
C,2.7345720761,-0.4726060239,-0.3966379214
C,3.2400538791,-0.4007944168,0.909593389
C,4.6118508202,-0.4176761106,1.1337392156
C,5.4993284565,-0.499750677,0.0611241935
C,5.0089787958,-0.5531272614,-1.2431702522
C,3.6371182136,-0.5314169584,-1.4687663334
H,2.5595827083,-0.2918115248,1.7473132107
H,4.9902950841,-0.3494604403,2.1485185488
H,6.5698183675,-0.5073421726,0.240943425
H,5.6952720527,-0.6083724612,-2.0822464194
H,3.2557183472,-0.5701585961,-2.4860287858

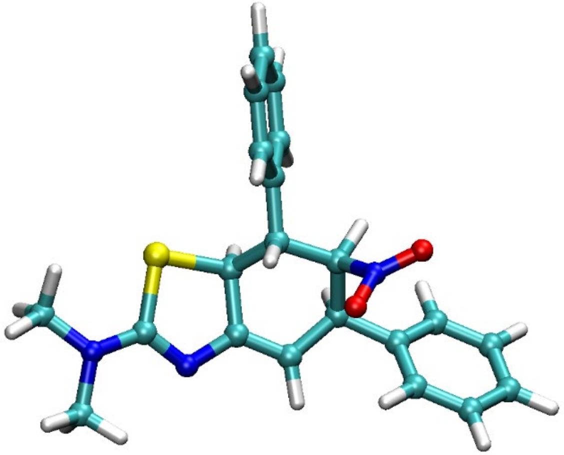

3'ea-*endoA*
C,-0.8357701046,-0.3443345598,-1.2519721633
C,-0.6429884482,-1.3551349104,-0.1312008282
H,-0.49444095,-0.7511209298,-2.2112959825
C,0.6079512453,-1.5904299015,0.2839439198
C,1.7333735031,-0.9042206588,-0.4435505577
H,0.8137949747,-2.2778868247,1.0957455855
H,1.7662482196,-1.2980339327,-1.4707726255
N,-1.8029675659,-1.8939633645,0.4010299253
S,-2.6357667189,-0.101588527,-1.3633556122
C,-2.8681373515,-1.3444997303,-0.091097662
O,2.9717272584,2.0730762431,0.2898928222
C,3.1113544506,-1.1310851745,0.1462563788
C,3.3190316315,-1.1544847628,1.528049051
C,4.5986988149,-1.3333841836,2.0478934629
C,5.6876485681,-1.4922071849,1.1937712094
C,5.4904653162,-1.4700130612,-0.1854253266
C,4.2101761509,-1.2904867191,-0.7019885679
H,2.4789067979,-1.0206378738,2.2028975964
H,4.7436804398,-1.345458769,3.1235450726
H,6.6842341645,-1.631068703,1.6007404598
H,6.3323763759,-1.5956607655,-0.8591061027
H,4.0619187203,-1.2759044375,-1.7792187057

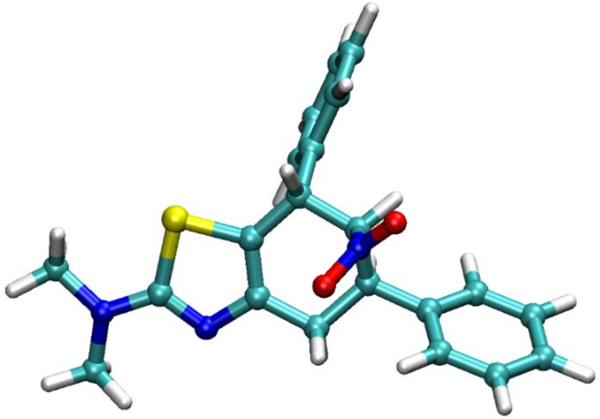

3ea-*endoA*
C,-1.2625000433,-0.3039572548,0.6947545413
C,-0.8309276225,-1.4239628394,0.064282741
H,0.6687030331,-2.060542365,-1.3107178615
C,0.6022286654,-1.6548680401,-0.2961552164
C,1.4126551566,-0.3523992,-0.2324049552
H,1.0115963723,-2.414940329,0.3761542192
H,1.0817983658,0.2690875317,-1.0741077366
N,-1.7796362104,-2.3853188236,-0.2141091898
S,-2.9825244606,-0.4177813856,0.9779270596
C,-2.9535532619,-2.011210208,0.2225397409
O,2.2826592289,0.5912291692,2.9757893971
C,2.9109397594,-0.5374566563,-0.3854553734
C,3.6226931512,-1.4980361182,0.3400755246
C,5.0043199223,-1.612757352,0.2001701601
C,5.6955958833,-0.7711515759,-0.6683078662
C,4.9964342944,0.1895420906,-1.3958985427
C,3.6161992449,0.3029519721,-1.2523120799
H,3.1054981281,-2.1610266851,1.0271001781
H,5.5404669556,-2.3620509431,0.7739868922
H,6.7715885529,-0.8627968123,-0.7775909991
H,5.5235328558,0.850131422,-2.0772143752
H,3.0765899698,1.0555674495,-1.8222788681

N,-4.3271144218,-1.0257847385,1.0986467087
C,-5.6466860039,-0.9188124312,0.5002278761
H,-5.7028726799,-0.0429467756,-0.1506686537
H,-5.9000259493,-1.8152202925,-0.0789506013
H,-6.3823884152,-0.7921963028,1.2954499165
C,-4.2185279054,-1.7140186717,2.379100542
H,-5.0153680537,-1.3589466898,3.0355540631
H,-4.3135503272,-2.7983597123,2.2485850108
H,-3.2519073019,-1.4937905982,2.8280835105
O,-0.3122933508,1.7300200682,1.5573629791
N,0.5718592332,2.1185260441,0.7675519943
C,0.2815894207,2.1745182885,-0.5898579937
H,1.1117064748,2.4779477997,-1.2092282815
C,-1.0392409102,1.9955562571,-1.0205711018
H,-1.7900704213,2.1557911861,-0.2505364435
C,-1.3974783436,2.4939549938,-2.3785208729
C,-2.6087903797,3.16300091,-2.5754712861
C,-0.550440722,2.2955072577,-3.4758264041
C,-2.9619358904,3.6338781575,-3.8388475233
H,-3.2763426927,3.3199195761,-1.7330825581
C,-0.9023372602,2.7632202414,-4.737297834
H,0.3904476931,1.7662653795,-3.3462102079
C,-2.110480765,3.4357294132,-4.9228493437
H,-3.9034445542,4.1571628805,-3.9734322587
H,-0.2331659307,2.6030429324,-5.576760751
H,-2.3864112767,3.8003231214,-5.9071283771

N,-4.1205137168,-1.6334515976,0.3221177929
C,-5.292575816,-1.2133473023,-0.4262526748
H,-5.1426049457,-0.2227987963,-0.8592200932
H,-5.5378599882,-1.9201046865,-1.2291714916
H,-6.1413341047,-1.1493318789,0.2576756334
C,-4.3024012448,-2.7313693228,1.2609690937
H,-3.4599243482,-2.7618299071,1.9498787587
H,-5.2260805718,-2.5657095598,1.8194938635
H,-4.3656568826,-3.6939659798,0.7381147836
O,1.3273162797,1.4025025126,1.5230513906
N,1.9439524698,1.4386012839,0.4685192984
C,1.4349537745,0.6163697744,-0.6888108215
H,2.0497428471,0.9571366622,-1.5209228496
C,-0.0599254774,0.9473422674,-0.9145434153
H,-0.4589473705,1.2855114782,0.0454367986
C,-0.2499321059,2.0470790044,-1.9380816768
C,0.1456719975,1.8705357579,-3.2685333182
C,-0.8383973246,3.2567581503,-1.566597609
C,-0.0447816357,2.88095321,-4.2057896491
H,0.6080962291,0.9368803148,-3.5813786318
C,-1.029446917,4.2723235592,-2.5026677638
H,-1.1518447592,3.4052036104,-0.5369705167
C,-0.6335408792,4.0865350168,-3.8242560178
H,0.2675172955,2.7282633759,-5.2340507201
H,-1.4874612913,5.207200953,-2.1956182659
H,-0.7811069962,4.8753261711,-4.5550467077

N,-4.0799797953,-2.7717813149,0.1386866798
C,-5.3765832238,-2.1544440573,0.3460993183
H,-5.3797683327,-1.5741057108,1.2728832176
H,-5.6630183621,-1.4961741166,-0.4867036461
H,-6.1280266176,-2.9392055948,0.4472464504
C,-4.0268633827,-3.94411071,-0.7212713662
H,-3.0968377274,-4.4840856914,-0.5460027279
H,-4.8705643153,-4.5930456164,-0.4791551238
H,-4.0756464909,-3.6699205745,-1.7840891368
O,1.1151919795,-1.185835565,2.5899099374
N,1.5277599027,-0.077239006,2.284180406
C,1.0723587796,0.5335333341,0.9829472727
H,1.6518334616,1.4543584969,0.92910963
C,-0.4368581082,0.8855785488,1.0782780695
H,-0.6409972812,1.1602361617,2.1208114205
C,-0.6961434687,2.1261691309,0.2249985157
C,-1.1497380715,2.0318135938,-1.0914576682
C,-0.423941804,3.3894690244,0.7583349018
C,-1.3198979108,3.1794175996,-1.8643772409
H,-1.3791491854,1.0557128813,-1.5094976309
C,-0.5935316111,4.5369111381,-0.0107247188
H,-0.0724550928,3.4741811759,1.7841287053
C,-1.0411642764,4.4339041916,-1.3275181064
H,-1.675382615,3.0914390694,-2.886369981
H,-0.3788868604,5.5109720871,0.4173962843
H,-1.175185801,5.3274420575,-1.9288748822

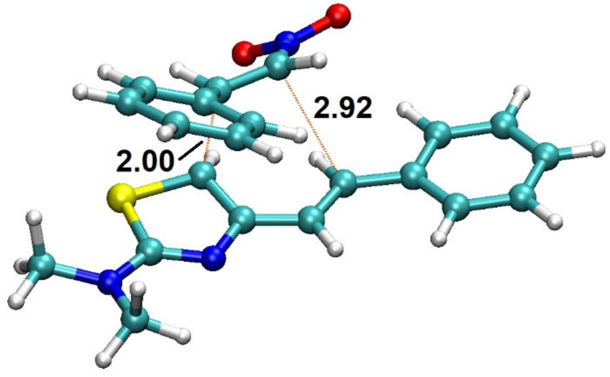

TS1ea-*exoA*

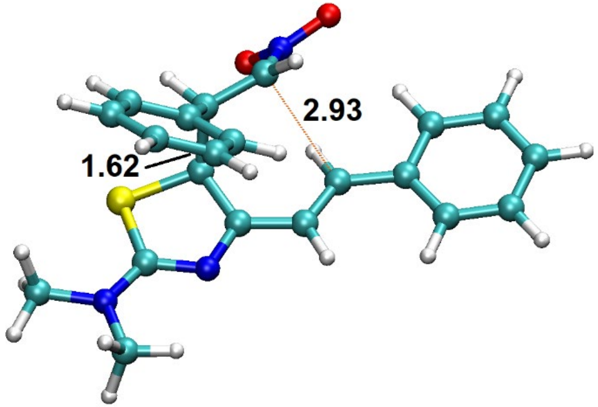

INTea-*exoA*

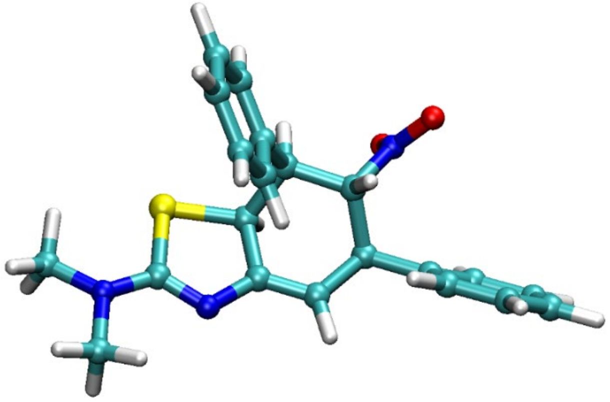

3’ea-*exoA*

C,0.6071864156,-0.6306773296,-1.3700465097
C,0.366357862,0.7274702861,-1.015338598
H,-0.0948298034,-1.2260713409,-1.9465969009
C,-0.9177599403,1.2410935054,-0.6559239778
C,-2.0641610366,0.5301686945,-0.8014922008
H,-0.911762426,2.2239608454,-0.1946745591
H,-2.0310161759,-0.4031612309,-1.3560494057
N,1.4629688563,1.5086618597,-0.8586478828
S,2.3007552978,-0.7528757088,-1.8613625665
C,2.5598983036,0.8693914765,-1.22754983
O,-2.9995217148,-2.7425926051,0.2658622179
C,-3.4002345989,0.9188620015,-0.3512594044
C,-3.667853341,2.1307864926,0.3077184585
C,-4.957778549,2.439598248,0.7175318148
C,-6.0036723924,1.5428550874,0.4855435732
C,-5.7507456704,0.3363590843,-0.1609824761
C,-4.4596465399,0.0284204045,-0.5797759402
H,-2.8689809936,2.8395591939,0.5009263423
H,-5.1512173425,3.3816631863,1.2207529658
H,-7.0101489872,1.7862262574,0.8113111374
H,-6.5552530455,-0.3699804535,-0.3374707596
H,-4.2618436561,-0.9219374653,-1.0660332144
N,3.788688555,1.3903931971,-1.1326440111
C,4.9569998248,0.5621936189,-1.38303945
H,5.1013601449,-0.173867754,-0.582266515
H,4.8617748493,0.040164134,-2.3392993901
H,5.8357932497,1.2047809768,-1.4375451106
C,3.9818030561,2.6563989181,-0.4353976179
H,3.0727218589,3.2498115613,-0.5080076193
H,4.2090634941,2.4814116263,0.6224935563
H,4.8090878903,3.1944452115,-0.9020689199
O,-1.3923795075,-3.0392377105,-1.1852051927
N,-1.8241562196,-2.5568106107,-0.1088258213
C,-0.992394502,-1.7635460929,0.6523842868
H,-1.4692213513,-1.2933677106,1.4978344692
C,0.3734444063,-1.6722329814,0.3244631403
H,0.7685884469,-2.555627078,-0.1718948372
C,1.3041801951,-1.0481239197,1.3002828923
C,2.6012658848,-1.5553212698,1.436283813
C,0.934031376,0.0629356347,2.0689232934
C,3.5064953084,-0.9731464919,2.3206882532

C,0.649112156,-0.6598430213,-1.0932903311
C,0.4094524823,0.7906864051,-0.8453899364
H,-0.0320104414,-1.1125462487,-1.8192873198
C,-0.8735426159,1.3095172971,-0.5369467485
C,-2.0099843677,0.5716981487,-0.6889288329
H,-0.8934970446,2.3233232074,-0.1503943075
H,-1.946103484,-0.3944388615,-1.1795788918
N,1.4749397848,1.5862933582,-0.8244602845
S,2.3510197037,-0.7239930414,-1.7249247481
C,2.5807341623,0.9361467829,-1.2159485037
O,-2.9112701742,-2.7631502684,0.2805102734
C,-3.3608275947,0.9788421611,-0.3267326509
C,-3.6783311259,2.2749516064,0.1171776308
C,-4.9849563247,2.6005719423,0.449849128
C,-5.9926818814,1.6365169708,0.3553008066
C,-5.6887266377,0.3479322476,-0.0756155138
C,-4.3818045602,0.0198294766,-0.4228872313
H,-2.9075409153,3.0353210794,0.1910178086
H,-5.2238944802,3.6047293413,0.7848705709
H,-7.0134761285,1.8951678741,0.619197573
H,-6.4665316448,-0.4055223092,-0.1404214216
H,-4.1338616082,-0.9927359637,-0.7308010644
N,3.7715976072,1.5100224213,-1.2445218525
C,4.955902667,0.7573430906,-1.6411863914
H,5.2527053438,0.05970101,-0.8516089519
H,4.7698268334,0.2049361384,-2.565823263
H,5.7674607531,1.4615453754,-1.8214597757
C,3.9722991184,2.8734143825,-0.759360644
H,3.0174964648,3.2915783873,-0.4516308764
H,4.6565284018,2.8519313406,0.0932543531
H,4.4053391385,3.4822357387,-1.5568280105
O,-1.3844829295,-2.7796179857,-1.3110739217
N,-1.745224756,-2.4733971576,-0.1223507969
C,-0.9005673424,-1.7864078268,0.6564329782
H,-1.2672915322,-1.5271557909,1.6375111621
C,0.5036583096,-1.5858829256,0.2272621426
H,0.8904889257,-2.5438216063,-0.1404127027
C,1.3865494907,-1.088933682,1.3525479321
C,2.6241662989,-1.6925774114,1.5907622772
C,1.0040074317,-0.0110615576,2.1588177992
C,3.4638119464,-1.2311048697,2.6026829052

C,-1.2505018887,0.9137089285,-0.2790378944
C,-1.0919844123,-0.59630272,-0.3645839421
H,-0.9292404566,1.3785302632,-1.2167358992
C,0.1579590931,-1.0742009081,-0.4409632009
C,1.2931821224,-0.0809872547,-0.4479864271
H,0.363035914,-2.1379148289,-0.4802102411
H,1.2599400553,0.515816343,-1.37016889
N,-2.2588261851,-1.3333663743,-0.2868223043
S,-3.0373893047,1.1920194499,-0.1111526284
C,-3.3012839231,-0.5793561709,-0.1136825969
O,1.9166884497,2.7663456325,-0.4308224456
C,2.6623007382,-0.7209323434,-0.3512613805
C,2.9411047848,-1.6581889585,0.6487966106
C,4.2051735005,-2.229492055,0.7531623232
C,5.2115699723,-1.8690715961,-0.1431201479
C,4.9442556761,-0.9346322206,-1.1397096396
C,3.6762480262,-0.3634589803,-1.2406678838
H,2.1617059734,-1.9445130004,1.3512892707
H,4.4072711187,-2.9544152948,1.5354099579
H,6.1976910911,-2.3154012978,-0.0628663293
H,5.7216294584,-0.6473899004,-1.8407642157
H,3.4727189733,0.3668783875,-2.0192240402
N,-4.5457471049,-1.0609835712,0.0761243658
C,-5.7198853371,-0.2079056954,0.0075962285
H,-5.5028082674,0.7797488634,0.4179560613
H,-6.0813713533,-0.0970294036,-1.0223772718
H,-6.5123437032,-0.6514953626,0.6135733628
C,-4.7598103306,-2.4944602752,-0.0652422124
H,-3.914550057,-3.033548495,0.3602546756
H,-5.6719831753,-2.7644243637,0.4702346687
H,-4.8621309233,-2.7794497479,-1.119528055
O,2.9051587796,2.2064323,1.4104693049
N,2.0426998311,2.0588958938,0.5607391337
C,1.073800591,0.9193641201,0.7280937566
H,1.3788952399,0.448307716,1.6610108722
C,-0.3664711388,1.4964101856,0.8597994578
H,-0.3123287063,2.5757866962,0.6953739156
C,-0.9362152742,1.268075951,2.2480550913
C,-1.3887159236,2.3589842348,2.9925268802
C,-1.0435510498,-0.0126213882,2.8023714499
C,-1.9445682315,2.1812204015,4.2579586306

H,2.9005791766,-2.4176740798,0.8478401873
C,1.8369351656,0.6468608837,2.950567229
H,-0.0601760585,0.486846985,1.9636117219
C,3.1266906895,0.1318682956,3.0790495006
H,4.5043422054,-1.3888511016,2.4217618411
H,1.5341922124,1.5088953795,3.5365714279
H,3.8290661271,0.5861638889,3.7710855889

H,2.9318850261,-2.5361081805,0.9790908607
C,1.8401581312,0.4544174907,3.17021361
H,0.0429166658,0.46919584,1.9976857358
C,3.0748076752,-0.1526438316,3.3942472923
H,4.4175703162,-1.7199230477,2.776320092
H,1.5235156364,1.2895723312,3.7873128889
H,3.7242821186,0.205987142,4.1868721523

H,-1.3092378225,3.3606783948,2.5781266
C,-1.603904696,-0.1938865039,4.0633705436
H,-0.695316331,-0.877105192,2.2451832197
C,-2.0575358778,0.9019360029,4.7958731453
H,-2.2889473415,3.0430434812,4.8209225392
H,-1.6858817725,-1.1956066218,4.4738180381
H,-2.4916709313,0.7597775989,5.7805150622

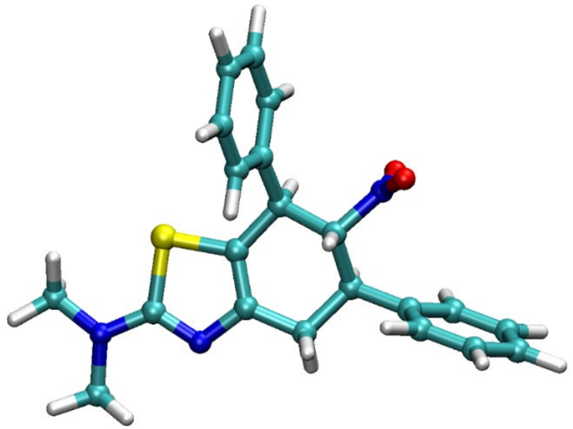

3ea-*exoA*
C,-1.2420333796,-0.2494762778,-0.1226634626
C,-0.8173463496,-1.5040441453,0.1699259949
H,0.8399595749,-2.8011512086,-0.1757928429
C,0.6315110496,-1.8516989029,0.3264710128
C,1.5399340712,-0.7617724255,-0.2691988054
H,0.8592194581,-2.0067266647,1.3880915367
H,1.4362000021,-0.7909718192,-1.3598163045
N,-1.7870474876,-2.4732386378,0.3128125766
S,-2.9843876031,-0.2379377269,-0.2524890312
C,-2.9763804144,-1.9582676337,0.1357224063
O,2.1329669146,1.8306234152,-1.4748664245
C,2.999447039,-0.9824929017,0.0764092974
C,3.4362948893,-0.9860146051,1.4055102494
C,4.7794432743,-1.1813258042,1.7118387571
C,5.7085443295,-1.3784923386,0.6911615149
C,5.2847316233,-1.37531515,-0.6351261957
C,3.9387632716,-1.1755856239,-0.937857604
H,2.7267085539,-0.8261395812,2.2132133621

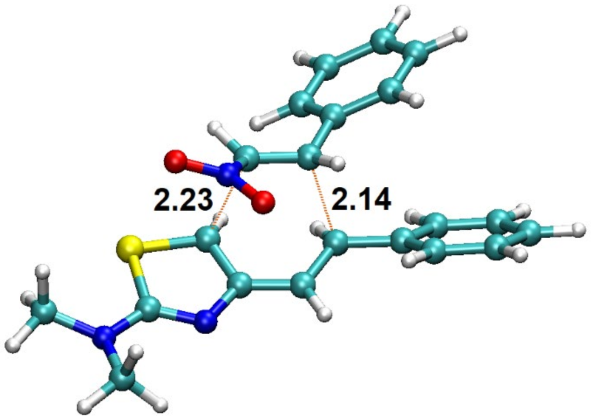

TS1ea-*endoB*
C,1.5919480642,-0.8514295154,-1.0182889639
C,1.6163299231,0.5627844779,-1.0803639534
H,0.8461187035,-1.4740939715,-1.4930297268
C,0.4420632368,1.310588358,-1.0287542548
C,-0.8103818133,0.690747645,-1.0194794175
H,0.5362765262,2.3763661243,-0.8449127324
H,-0.9104965835,-0.2267890632,-1.5942826966
N,2.8677908396,1.1358573061,-0.9426969497
S,3.2239811964,-1.4541189314,-0.9665431636
C,3.7981733176,0.2312144167,-0.8701209737
C,-2.0637244035,1.4664873999,-0.9119262676
C,-2.1595808646,2.5932793249,-0.084918484
C,-3.3587860042,3.2902413272,0.0246999617
C,-4.4847356782,2.8668822282,-0.682378853
C,-4.4035081119,1.7391380191,-1.49614709
C,-3.2032435955,1.0424333733,-1.6041151406
H,-1.2954289221,2.9163957583,0.4895784917
H,-3.4168818412,4.1616018735,0.6694857171

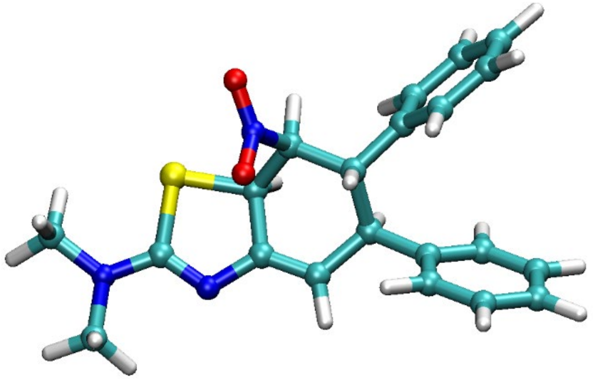

3’ea-*endoB*
C,-0.9103511259,0.7393716136,-0.7234795872
C,-0.5700526944,-0.7247737511,-0.4893086919
H,-0.5934454314,1.0335527269,-1.7302724396
C,0.7235555324,-1.0505293244,-0.3990538877
C,1.7490001349,0.0505250539,-0.4872473707
H,1.0330044939,-2.0780821196,-0.2430438306
H,1.7918662425,0.4346717911,-1.5180127826
N,-1.6554689328,-1.5773897968,-0.3792790497
S,-2.7246985718,0.8335904885,-0.6597731705
C,-2.7850195242,-0.9514098314,-0.4408643052
C,3.1454674887,-0.4029868078,-0.1137653307
C,3.3685716853,-1.1150422288,1.0685847247
C,4.6572472085,-1.486907679,1.4427364917
C,5.7446382127,-1.1498599569,0.6373544904
C,5.5319542987,-0.4410095303,-0.5426257523
C,4.2404473393,-0.0705781578,-0.9124427969
H,2.5271341715,-1.380293433,1.704548359
H,4.812319813,-2.0390536432,2.3646572941

H,5.101596105,-1.1742590696,2.7484014453
H,6.7571397396,-1.5273405266,0.9285272357
H,6.0014413312,-1.523126479,-1.4367599009
H,3.6155033151,-1.1626983639,-1.9752361887
N,-4.1386188982,-2.65560612,0.2653066251
C,-5.3713681254,-2.0987241224,-0.259627038
H,-5.4921077679,-1.0619304238,0.0660439264
H,-5.4101654259,-2.1334357091,-1.3577021635
H,-6.2121848759,-2.6684669307,0.1393197366
C,-4.0422264846,-4.105738152,0.3372118014
H,-3.2236678836,-4.3846442635,0.999877405
H,-4.9791239533,-4.4977650782,0.7377082139
H,-3.8581709144,-4.5487077959,-0.6510104169
O,2.5511007119,2.3467273593,0.5837730917
N,1.9828968189,1.6871326482,-0.2696655566
C,1.0286427749,0.6169460677,0.1905322856
H,1.0399297903,0.6950209326,1.2793403033
C,-0.3732750567,0.9532078277,-0.3623660144
H,-0.2616629756,1.1111527067,-1.4424794109
C,-0.905005727,2.2385233273,0.2499045005
C,-0.959186291,3.4064330832,-0.5109015574
C,-1.3075160491,2.2811415172,1.5884131639
C,-1.4090401886,4.5997106774,0.0536151796
H,-0.6448303994,3.3844862804,-1.5509539402
C,-1.7548850274,3.4698421149,2.1543531202
H,-1.2780776021,1.3744831513,2.187971759
C,-1.8068889117,4.6340564475,1.3868313322
H,-1.4460648831,5.5003678947,-0.5510358165
H,-2.0637851696,3.4897678849,3.194760167
H,-2.1552548335,5.5621113356,1.8289622043
H,-5.4209493639,3.4088241907,-0.5927272533
H,-5.2771191149,1.3954246293,-2.0412449036
H,-3.1490833316,0.1532236305,-2.2262736324
N,5.1189311144,0.4956635512,-0.7364972105
C,6.0396950493,-0.5614563103,-0.3534416439
H,5.885182333,-1.4484321884,-0.973780889
H,7.0593271112,-0.2145641714,-0.5268402309
H,5.9312479116,-0.8397014581,0.7028931069
C,5.4962487277,1.8650598765,-0.4126946392
H,5.3078541604,2.092384019,0.64412137
H,6.5585098805,1.993662388,-0.627419298
H,4.9188829795,2.5567856025,-1.0250878046
C,-0.8101588991,-0.5169359057,0.7471566468
C,0.4619286666,-1.0929861634,0.8938777974
H,0.6319683995,-2.1603993299,0.9113935907
H,-0.994598275,0.3609541109,1.3563423659
N,1.4090301637,-0.3951298358,1.7112382104
O,2.3817728393,-1.030346774,2.1270457136
O,1.2335856097,0.8042359417,1.9321675097
C,-1.9797706262,-1.3780736348,0.4789836475
C,-1.8891195296,-2.5349441296,-0.30769605
C,-3.2338328778,-1.0099199618,0.9814237831
C,-3.0169140433,-3.3054648791,-0.5732650465
H,-0.9325413999,-2.8411001572,-0.7234431862
C,-4.3612385534,-1.7831990373,0.7217112082
H,-3.3234011759,-0.1041443504,1.5742967435
C,-4.2581204854,-2.933554044,-0.0575705681
H,-2.9269046924,-4.1975007748,-1.1853527313
H,-5.3229722325,-1.4817060228,1.1245624547
H,-5.137738955,-3.5347524825,-0.264474904
H,6.7501257965,-1.4377250077,0.9282489731
H,6.3712536052,-0.1723380659,-1.1767830351
H,4.0828589833,0.4931848682,-1.8281391618
N,-3.9881648181,-1.5608570114,-0.3835800616
C,-5.2145750675,-0.8179526424,-0.1461598459
H,-5.395915732,-0.6659914999,0.9250552231
H,-5.1812561077,0.1542788132,-0.6410088922
H,-6.0515910417,-1.3755042513,-0.5713683807
C,-4.0113161251,-2.9839334813,-0.0724003583
H,-4.9795910323,-3.3899214802,-0.3706838149
H,-3.2191635002,-3.4910544298,-0.6219251001
H,-3.8604176184,-3.1587532358,0.9998805206
C,1.3330746757,1.2511103569,0.4347490095
C,-0.1434873554,1.6831858939,0.2262870165
H,-0.2112677562,2.7061731789,-0.1397509248
H,1.40659419,0.8660506683,1.4552562337
N,-0.8398093494,1.7466863261,1.5651291371
O,-1.3403149387,2.8155241931,1.8791518262
O,-0.87630559,0.7350624785,2.2458722076
C,2.289832901,2.414307526,0.2839382448
C,3.1914851753,2.7224373158,1.3030560198
C,2.3368443831,3.1556217742,-0.9009380684
C,4.1234068748,3.746303424,1.1443293619
H,3.1743158758,2.1464880326,2.2241271257
C,3.2650306322,4.1796424441,-1.0641478771
H,1.6480889285,2.9303323235,-1.7124906978
C,4.1638035012,4.4769503127,-0.0403940464
H,4.8198398612,3.9688001976,1.946475883
H,3.2889811062,4.7434802191,-1.9915066166
H,4.8916943017,5.2720758251,-0.1668531753

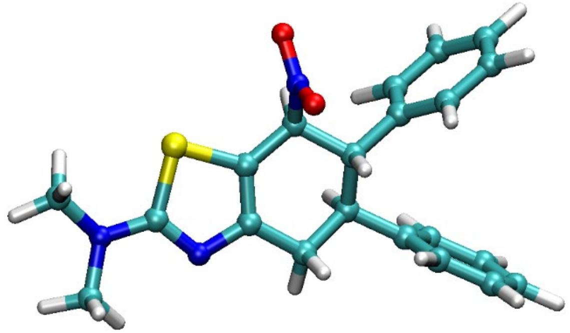

3ea-*endoB*

C,1.6393344611,-0.4579384574,0.0159848417
C,1.6750535762,0.8947223489,-0.1029512689
C,0.4346082165,1.7319496942,-0.1094606634
C,-0.7996984058,0.888575417,-0.4680804317
H,0.3062542157,2.1962138791,0.8759445975
H,-0.7035317868,0.5889186446,-1.5197473734
N,2.9140167107,1.4673547958,-0.253707183
S,3.2604403675,-1.1063997303,-0.087796228
C,3.8470939924,0.546439823,-0.2727244469
C,-2.0845833378,1.6803283408,-0.3258691981
C,-2.4611633539,2.2281728617,0.9048210191
C,-3.6558450179,2.929201459,1.0386139518
C,-4.4972570157,3.0950234225,-0.0607670695
C,-4.1324397009,2.5551599123,-1.2911871175
C,-2.9349559133,1.8530420219,-1.418816457
H,-1.8230286262,2.1005420667,1.7756187651
H,-3.9313961962,3.3425780713,2.003979265
H,-5.4316567418,3.6375693379,0.0428311903
H,-4.7815897935,2.675281758,-2.1530932196
H,-2.6637105916,1.4252135411,-2.3804574991
N,5.1678824437,0.800666016,-0.4443373549
C,6.143371457,-0.2484291609,-0.2140281743
H,6.25708744,-0.4850333268,0.8527162649
H,5.858856908,-1.1582909678,-0.7506086014
H,7.107345358,0.0783403939,-0.6064010224
C,5.6163509335,2.181438664,-0.3427008788
H,6.5727006268,2.2777713124,-0.860398078
H,4.8839525073,2.8360418731,-0.8133883397
H,5.740466558,2.4897138901,0.7036588321

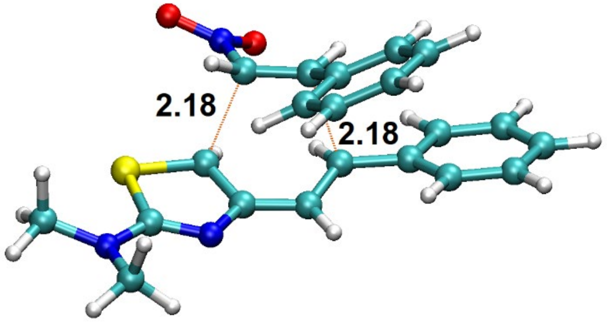

TS1ea-*exoB*

C,-1.1360169869,0.5742232862,-0.697342972
C,-0.8528457922,-0.7922985802,-0.4553409092
H,-0.6173418812,1.1630817623,-1.4449128842
C,0.4564361847,-1.2693774771,-0.4254096518
C,1.5417825098,-0.4099810853,-0.5828992795
H,0.599855046,-2.2882168504,-0.0799699085
H,1.4112821536,0.4750189309,-1.2014675326
N,-1.9220415694,-1.5507811904,-0.0085567967
S,-2.8571577554,0.835293103,-0.5323679906
C,-3.0232973008,-0.8558708209,-0.0001033226
C,2.944162204,-0.8488255799,-0.462167535
C,3.3188378681,-1.9787398964,0.2771143369
C,4.6575505801,-2.3411658101,0.3880903648
C,5.647743851,-1.5732320516,-0.2225031561
C,5.2895859465,-0.4349647595,-0.9440794008
C,3.9504032448,-0.0765463925,-1.058784363
H,2.565205835,-2.569734806,0.7880968818
H,4.9293755947,-3.2206386343,0.9636075438
H,6.6915312109,-1.8572994265,-0.1332734259
H,6.054090017,0.1724884079,-1.4183223301
H,3.6744280641,0.8133745372,-1.6189547219
N,-4.2314464639,-1.3415431938,0.3701752253
C,-5.3469771864,-0.4420663616,0.6076155081
H,-5.2512891535,0.0908929355,1.5628969841
H,-5.4303894561,0.2889332587,-0.2005099446
H,-6.2692808146,-1.024647082,0.6194751349
C,-4.2719659274,-2.6457229531,1.0157163461
H,-3.5482781913,-3.3076679482,0.5421665619
H,-4.0324869735,-2.5700854924,2.0847745141

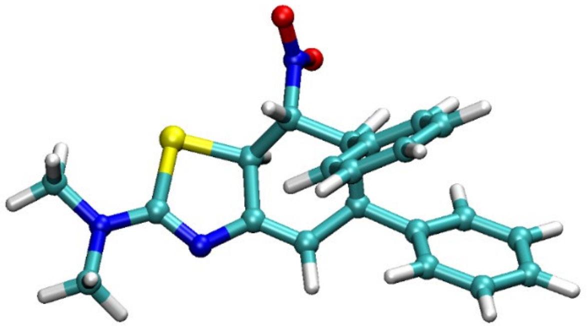

3'ea-*exoB*

C,-1.085711802,0.654856221,-0.4462035964
C,-0.8202879598,-0.8465975241,-0.423535461
H,-0.7358190715,1.0954648496,-1.3854427714
C,0.4554341014,-1.2472262993,-0.3627838829
C,1.5135432535,-0.1778211307,-0.3053313716
H,0.7094960158,-2.3007554707,-0.3490399108
H,1.4875797026,0.3889012539,-1.2478576691
N,-1.9474437511,-1.6512737305,-0.419573092
S,-2.8947302727,0.8227444967,-0.3590723584
C,-3.0459736144,-0.9672685514,-0.3931105727
C,2.9336014161,-0.6755446949,-0.1381745944
C,3.2444356296,-1.7870199694,0.6498217363
C,4.5672947271,-2.1808303301,0.8336117215
C,5.6032281455,-1.4644516363,0.2369218381
C,5.3054135762,-0.3525672879,-0.5481248052
C,3.9798538394,0.0340412808,-0.7332688124
H,2.4497289058,-2.3379610053,1.1435439007
H,4.7886299177,-3.0459595052,1.4510199633
H,6.6344712821,-1.7707141999,0.3826505898
H,6.1040601738,0.2114208117,-1.0201876184
H,3.7543793499,0.902747756,-1.347733733
N,-4.2786228447,-1.5157900539,-0.412828171
C,-5.464260412,-0.7333251472,-0.1046779028
H,-5.6081750368,-0.6171595488,0.9767808101
H,-5.4039759346,0.2552686373,-0.5645001017
H,-6.3360671871,-1.2383521437,-0.5240848883
C,-4.3910390409,-2.9632008311,-0.2972719752
H,-5.3336130753,-3.2787792946,-0.7489386861
H,-3.5612774985,-3.433870236,-0.8222655055

C,-0.8445088045,-0.400068891,0.3955045797
C,0.4011874637,-1.272554048,0.1405007274
H,0.258623967,-1.9395683783,-0.7125456036
H,-0.7930307026,-0.0873384055,1.4439746824
N,0.5443196165,-2.254234345,1.3077817681
O,0.6901903937,-3.4318806067,1.0197499011
O,0.5267574679,-1.8137907698,2.4442445453
C,-2.1121671575,-1.2054051596,0.1929617934
C,-2.994704615,-1.4088266838,1.2536997402
C,-2.4319132469,-1.7401097031,-1.0590705966
C,-4.1740392036,-2.1293096238,1.0711820853
H,-2.7600230844,-0.9971701672,2.2314444363
C,-3.6075562165,-2.4596486068,-1.24635521
H,-1.7627759386,-1.5881669613,-1.9027903025
C,-4.4839169326,-2.6558702582,-0.1796560019
H,-4.849891278,-2.277747914,1.9074494049
H,-3.8403559043,-2.8651242832,-2.2260670291
H,-5.4023247403,-3.2161028403,-0.3238238208
H,0.5507035052,2.5498263536,-0.8266553216
H,-5.2738917959,-3.0638997525,0.9016117831
C,1.2290881086,0.9074543598,1.1244372363
H,2.1145543004,1.4572991531,0.8216027905
C,-0.000098505,1.5469783335,0.891358224
H,-0.814528258,1.5168181708,1.6031384817
N,0.0464367573,2.8132666197,0.2105285971
O,-0.8553776208,3.6173986225,0.4373073683
O,0.9464226544,3.0110233417,-0.6089572621
C,1.3832251107,-0.003951274,2.2683547489
C,0.3129920972,-0.7618831683,2.7667107271
C,2.6465618049,-0.1612516163,2.8539538707
C,0.503690472,-1.6441863609,3.8247173203
H,-0.6715147689,-0.6851107902,2.3142598858
C,2.8347104585,-1.0399952377,3.9167416061
H,3.4874050811,0.4062511172,2.4655778225
C,1.7645038938,-1.78614354,4.4053562875
H,-0.3344239137,-2.2269055641,4.1941753052
H,3.8216748986,-1.1470902245,4.3558734849
H,1.9092499573,-2.4756894196,5.2310527555
H,-4.3705818458,-3.2818519166,0.7524928621
C,1.16203779,0.8779602324,0.8109402819
H,1.7813528376,1.7534867718,0.5953505031
C,-0.3293147352,1.3158315559,0.7159607526
H,-0.8529196328,1.133421626,1.6527586668
N,-0.3764682229,2.8143785374,0.556270135
O,0.0306698988,3.2857643207,-0.4964355452
O,-0.8049203841,3.4698775098,1.491664164
C,1.514457251,0.3949384772,2.2036773277
C,2.6693401851,0.8771306879,2.8231523701
C,0.7515983291,-0.5714090004,2.8679599328
C,3.0609993385,0.4039522758,4.0730496451
H,3.2788640718,1.6187658347,2.3139403879
C,1.1402210163,-1.0469672817,4.1175998226
H,-0.1440161438,-0.9738485597,2.4034016607
C,2.2974155695,-0.5616029987,4.7242786743
H,3.9633240631,0.7908605782,4.5360179207
H,0.5368228278,-1.7981489258,4.6179298728
H,2.5988677607,-0.9318139308,5.6991015154

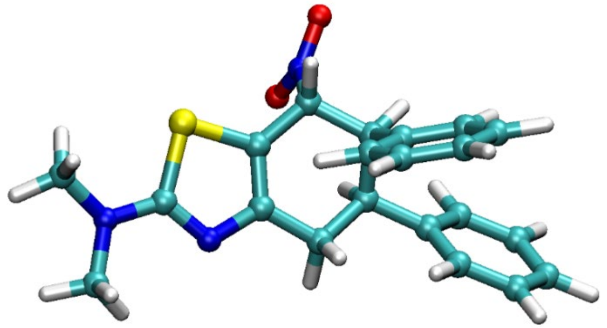

3ea-*exoB*
C,-1.5194450227,0.5057655515,-0.1798380805
C,-1.3126715788,-0.8368024684,-0.2528658308
C,0.0431926459,-1.4335407079,-0.4590052764
C,1.0145993984,-0.3945494328,-1.0280317166
H,0.391880623,-1.8287885094,0.5029480907
H,0.6603046295,-0.1550628682,-2.0371906902
N,-2.4091964697,-1.6393591151,-0.0641115803
S,-3.2070124438,0.8271425703,0.1455541833
C,-3.4845765455,-0.9127583248,0.1288471304

C,2.440246685,-0.8892847498,-1.1712464781
C,3.0641730105,-1.6883302402,-0.2084401261
C,4.3902582187,-2.0868769806,-0.362900594
C,5.1189380127,-1.6877813999,-1.4814172119
C,4.5101483229,-0.8878548654,-2.4464024412
C,3.1826774458,-0.4962342874,-2.2893710907
H,2.5242766067,-1.9931752409,0.6826276715
H,4.8555303592,-2.7061748167,0.3978701153
H,6.151770634,-1.9997673173,-1.6015480937
H,5.0653043378,-0.5724977665,-3.3245705841
H,2.7126786018,0.1237972629,-3.0492766731
N,-4.7299502973,-1.4242562036,0.2835231784
C,-5.8055906319,-0.5750590663,0.7607123683
H,-5.7010323719,-0.3341145531,1.8276256595
H,-5.8395138932,0.3563163879,0.1880267891
H,-6.7549469642,-1.0899561444,0.60840932
C,-4.8543706115,-2.86039074,0.4851001488
H,-5.8841053222,-3.1535020383,0.2728323246
H,-4.1831452546,-3.3848056287,-0.194121643
H,-4.6032813624,-3.1453950347,1.5152407352
C,0.9577370984,0.9478113728,-0.2357080039
H,1.6084068003,1.6537961115,-0.7623359212
C,-0.4730886687,1.5457657852,-0.2797849414
H,-0.5648522139,2.3248188361,0.479508387
N,-0.5864637995,2.3410581969,-1.5899686825
O,-0.9418825976,1.763457512,-2.6022219785
O,-0.2636837052,3.5189557632,-1.5376366575
C,1.4915562653,0.8433157706,1.1822656667
C,2.8301176981,1.175101726,1.4158827357
C,0.7218888099,0.3888963387,2.2585253134
C,3.3922110237,1.0447575619,2.6822799558
H,3.4446407184,1.5247169829,0.5907595039
C,1.2806919092,0.2618355091,3.5290968205
H,-0.3229496005,0.1325520092,2.1127714375
C,2.6182359299,0.5838704986,3.745323955
H,4.4356846004,1.3009174146,2.8363745859
H,0.6650330289,-0.0925032175,4.3501282914
H,3.0536555237,0.4795320778,4.7341855472
H,-0.0294874229,-2.2866621719,-1.1400395294

Reactants

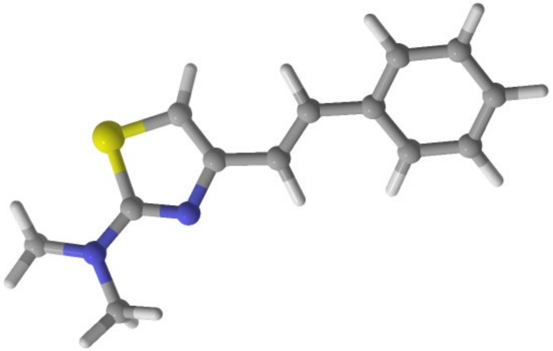

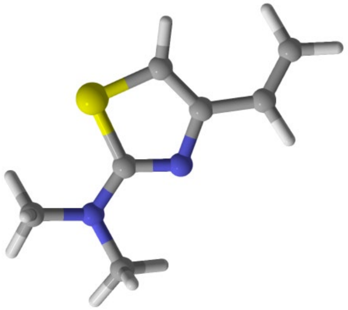

1e

C,-1.1721533197,-1.526931503,0.4701478399
C,-0.7463992638,-0.2582097522,0.2053602913
H,-0.5897947423,-2.3977949105,0.7324530535
C,0.6407103243,0.2112389513,0.167997206
C,1.7157594467,-0.5796449229,0.0328060698
H,0.7488004034,1.2906094563,0.2390647994
H,1.5604489366,-1.6494494938,-0.1027723508
N,-1.7465473421,0.6689732948,-0.0348791402
S,-2.9029492688,-1.6073146039,0.4008070651
C,-2.9259295266,0.1101833855,0.0085474628
C,3.123265243,-0.1517216535,0.0170705493
C,3.5382032228,1.1375802304,0.3860152254
C,4.8817865563,1.4925641343,0.3421218472
C,5.8445982861,0.5672154549,-0.0633440847
C,5.4494450632,-0.719393783,-0.4217862624
C,4.103386316,-1.072799286,-0.3792185433
H,2.8093813256,1.8685224923,0.7218756828
H,5.1809032192,2.4947716069,0.6337497003
H,6.893208321,0.8460838782,-0.0926211646
H,6.1887289679,-1.4502966701,-0.7344738759
H,3.8034316586,-2.0784346531,-0.6625733852
N,-4.0946536845,0.76175887,-0.2504286346
C,-5.351230135,0.1495610654,0.1369456449
H,-5.415562325,-0.8707390038,-0.252485442
H,-5.4867521503,0.1225188879,1.2276957873
H,-6.1700994301,0.720076449,-0.303853902
C,-4.0448401159,2.2141964484,-0.3127210147

1b

C,1.1369529472,1.1184200992,0.0367143285
C,1.1187979926,-0.2442976259,0.0162515378
H,1.9831367076,1.7853477126,0.1106290682
C,2.2813368211,-1.1421915965,0.0596965106
C,3.5551589557,-0.7864241766,-0.127610784
H,2.0335634703,-2.182908689,0.2544976125
H,3.8441961359,0.2371274098,-0.3509533967
N,-0.1373240529,-0.8237568943,-0.0407808227
S,-0.4791559759,1.7422046284,-0.0329329217
H,4.3515384798,-1.5204636119,-0.0676607599
C,-1.0732838646,0.0852003345,-0.096495661
N,-2.4033457137,-0.1868810947,-0.226429353
C,-2.8283134534,-1.5591339271,0.0033379104
H,-2.8763480426,-1.7943859687,1.0756461945
H,-3.8173809406,-1.6964882257,-0.4384367256
H,-2.1244396608,-2.2422566123,-0.4705082648
C,-3.3747942529,0.856185415,0.0417707343
H,-3.4616308057,1.0814097023,1.1145416603
H,-3.1100446617,1.7731662713,-0.4919450571
H,-4.3487667953,0.5308612798,-0.3278940405

H,-3.1766304757,2.5233380466,-0.8941911352
H,-4.9523701548,2.5748532683,-0.8007117652
H,-3.9700393561,2.6636573153,0.6870784759

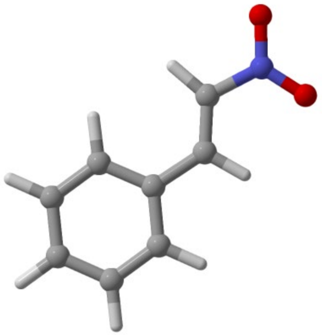

2a

O,2.4518135622,1.5558204647,1.3832989155
O,0.5022791094,1.8509322005,2.2824831823
N,1.2488282513,1.791447516,1.3118147434
C,0.7196953698,2.0059889806,-0.0170969329
H,1.4853951967,1.9245512483,-0.7746820568
C,-0.579058477,2.2688360267,-0.1900309147
H,-1.195619116,2.3102038096,0.704763458
C,-1.2528270107,2.5066679372,-1.4669105113
C,-2.6299373668,2.7686662962,-1.4480214426
C,-0.5846590426,2.4840217085,-2.7017504617
C,-3.3253879445,3.0025709769,-2.6301840898
H,-3.1563925831,2.7884461483,-0.4980470937
C,-1.279648466,2.7175512965,-3.8805441243
H,0.4809806814,2.2837610492,-2.7462550388
C,-2.651317854,2.9773911533,-3.8483505701
H,-4.3910900675,3.2039982992,-2.6001939743
H,-0.7532452853,2.6976155412,-4.8291494661
H,-3.1902529573,3.1593493471,-4.7725886221

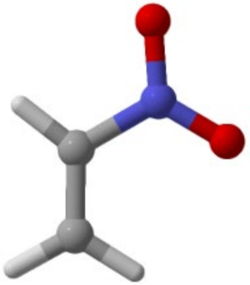

2b

O,3.8127058176,-0.9529866344,-0.0004057803
O,3.2885516772,1.1495651252,0.000347955
N,2.9982076281,-0.0379096793,-0.0000410776
C,1.5916087692,-0.4322111168,-0.0001159089
H,1.4719830372,-1.5065622756,-0.0004397967
C,0.6315421852,0.4804478637,0.0001836545
H,0.8622305341,1.5398174207,0.0004965266
H,-0.4054103685,0.1667578564,0.0001164173

2.7. IRC Energy Plots Corresponding to the Movies

Correspondence between the names of the energy plots and the videos of the IRC (Intrinsic Reaction Coordinate) calculations:

Name of IRC Energy plot	Name of IRC Movie	Movie
ircforward_TS1ba_endoA_plot	ircforward_TS1ba_endoA.mp4	S1
ircforward_TS1ba_endoB_plot	ircforward_TS1ba_endoB.mp4	S2
ircforward_TS1ba_exoA_plot	ircforward_TS1ba_exoA.mp4	S3
ircforward_TS1ba_exoB_plot	ircforward_TS1ba_exoB.mp4	S4
ircforward_TS1bb_exoA_plot	ircforward_TS1bb_exoA.mp4	S5
ircforward_TS1bb_exoB_plot	ircforward_TS1bb_exoB.mp4	S6
ircforward_TS1ea_endoA_plot	ircforward_TS1ea_endoA.mp4	S7
ircforward_TS1ea_exoA_plot	ircforward_TS1ea_exoA.mp4	S8
ircforward_TS1eb_endoA_plot	ircforward_TS1eb_endoA.mp4	S9
ircforward_TS1eb_exoA_plot	ircforward_TS1eb_exoA.mp4	S10
ircforward_TS2bb_endoA_plot	ircforward_TS2bb_endoA.mp4	S11
ircforward_TS1bb_endoA_plot	ircfoward_TS1bb_endoA.mp4	S12

1. ircforward_TS1ba_endoA_plot

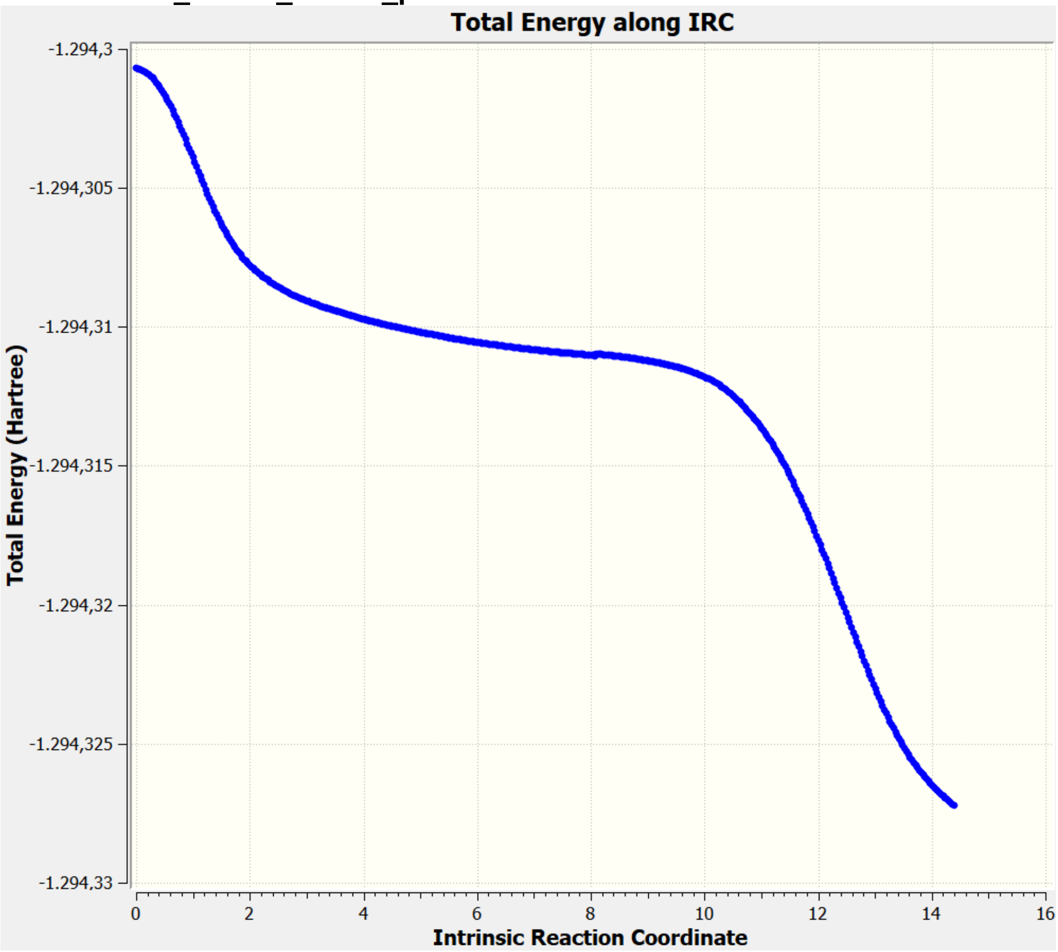

2. ircforward_TS1ba_endoB_plot

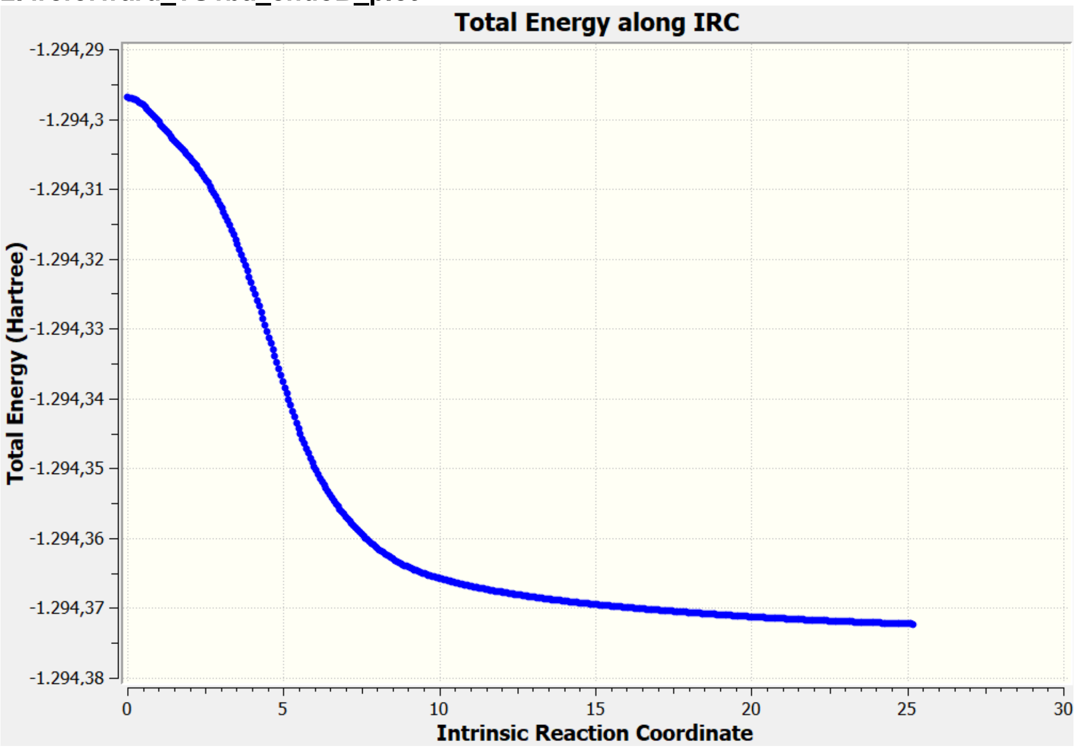

3. ircforward_TS1ba_exoA_plot

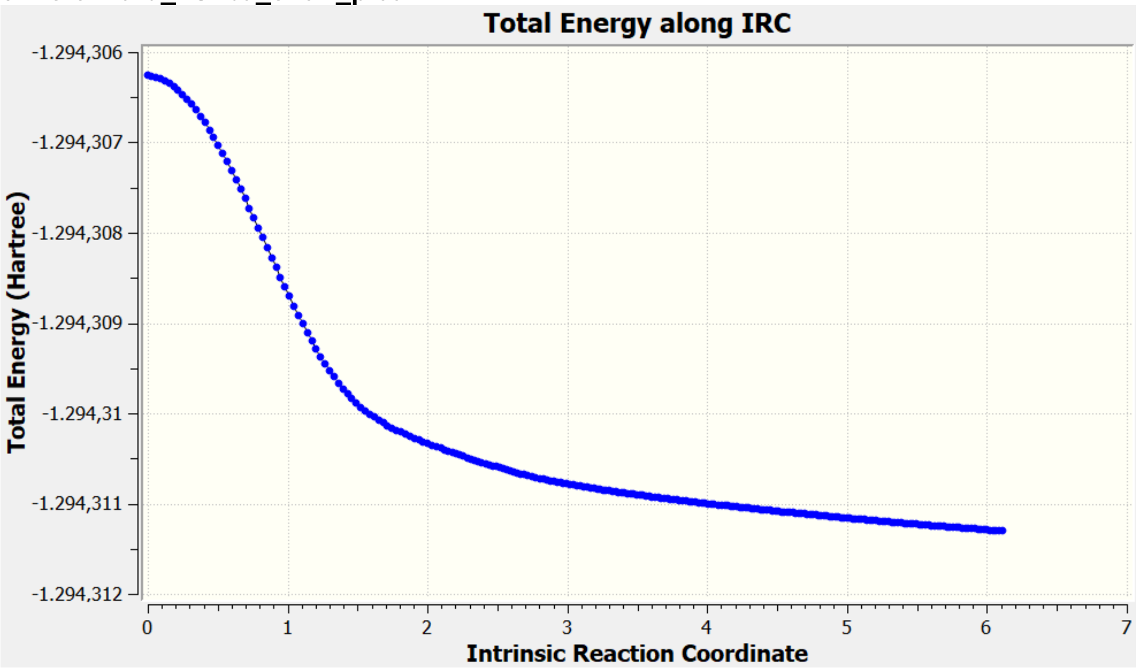

4. ircforward_TS1ba_exoB_plot

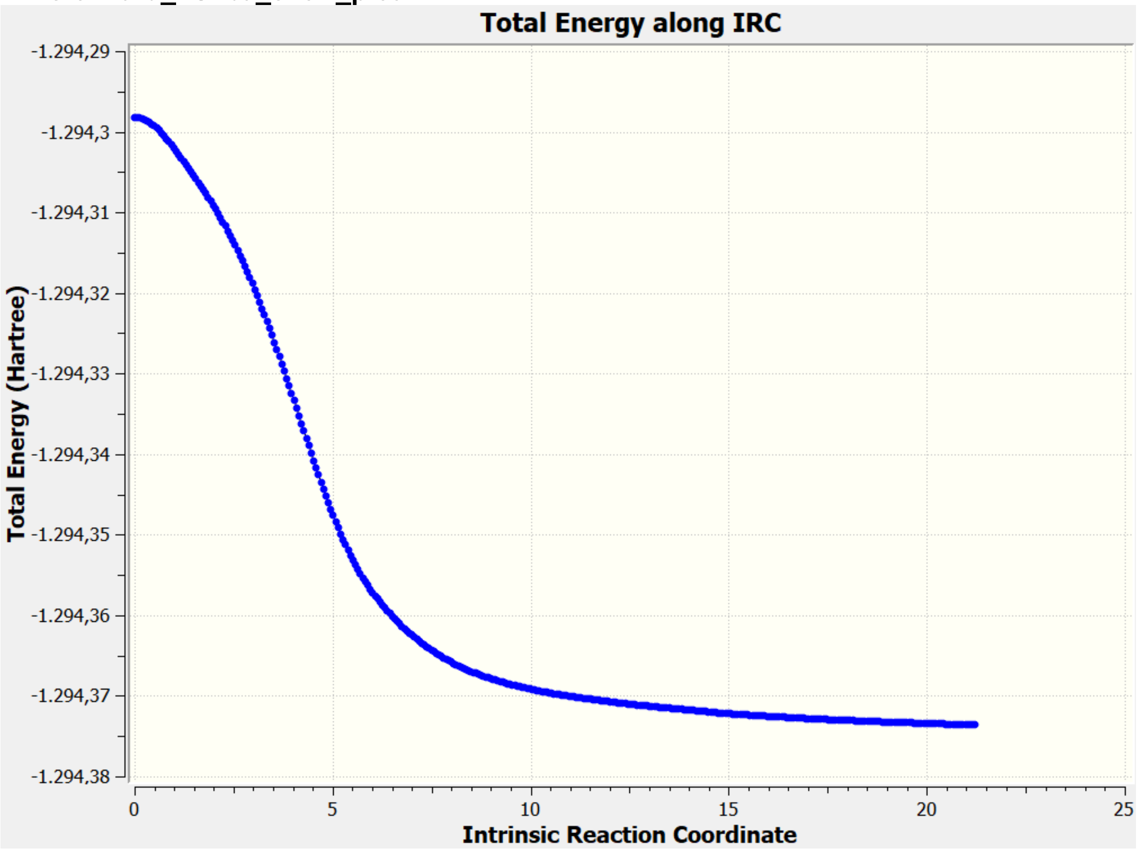

5. ircforward_TS1bb_exoA_plot

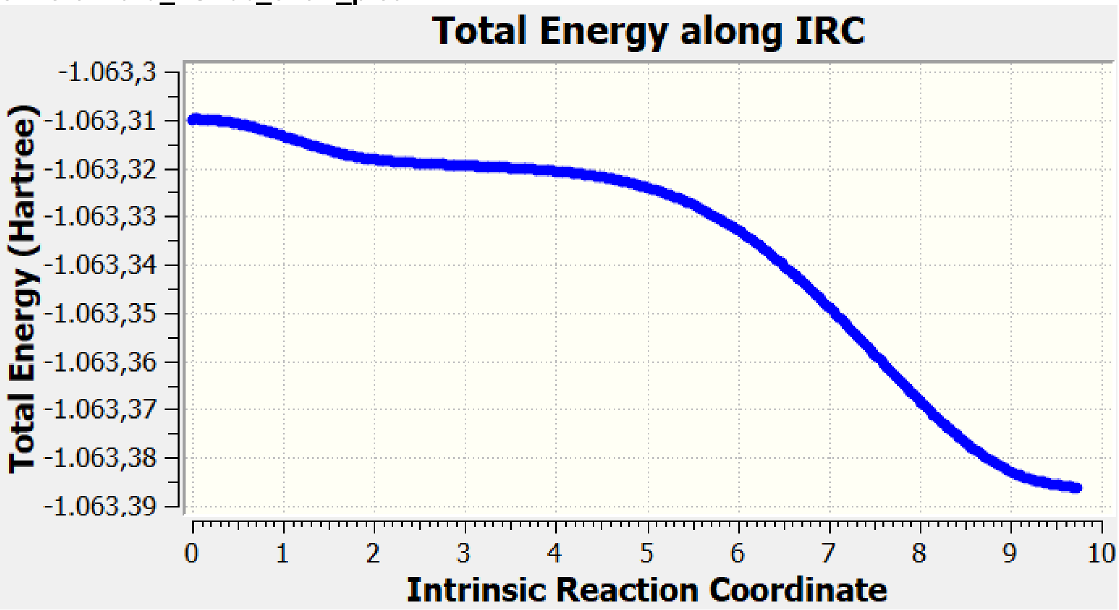

6. ircforward_TS1bb_exoB_plot

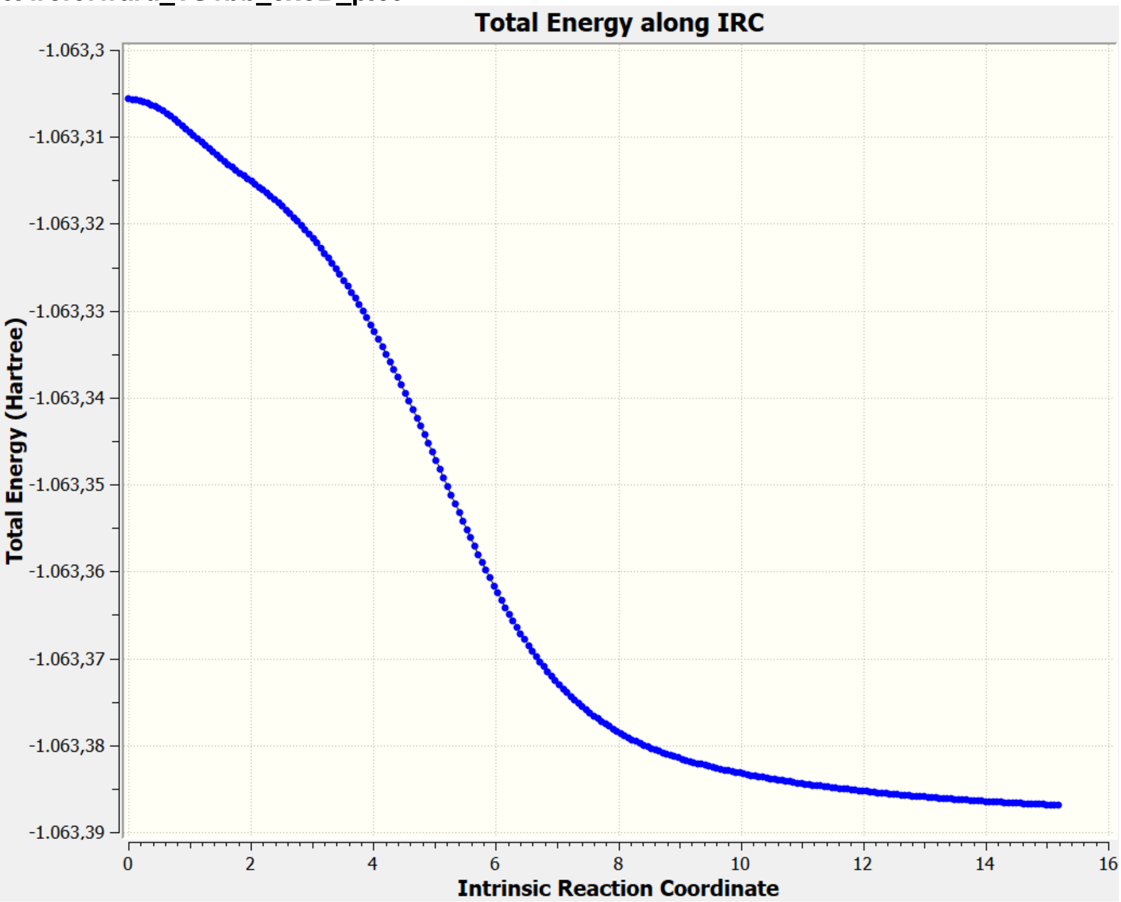

7. ircforward_TS1ea_endoA_plot

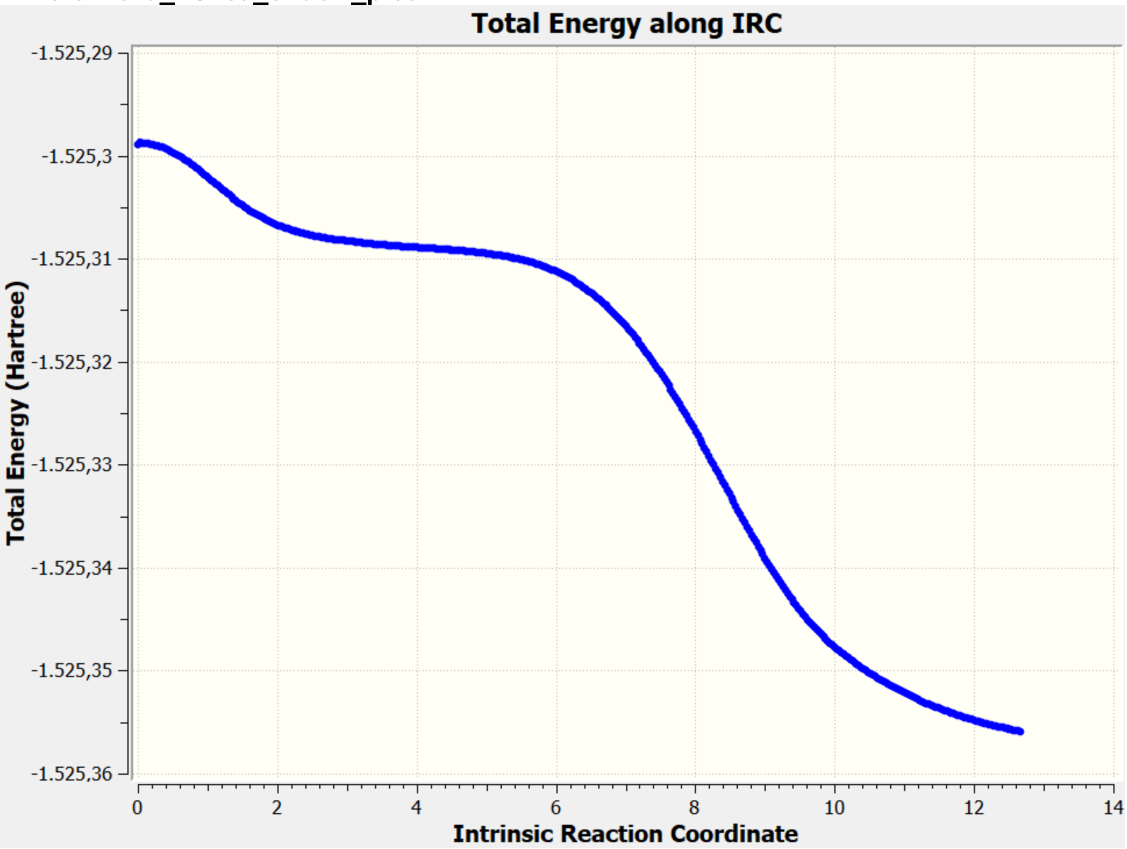

8. ircforward_TS1ea_exoA_plot

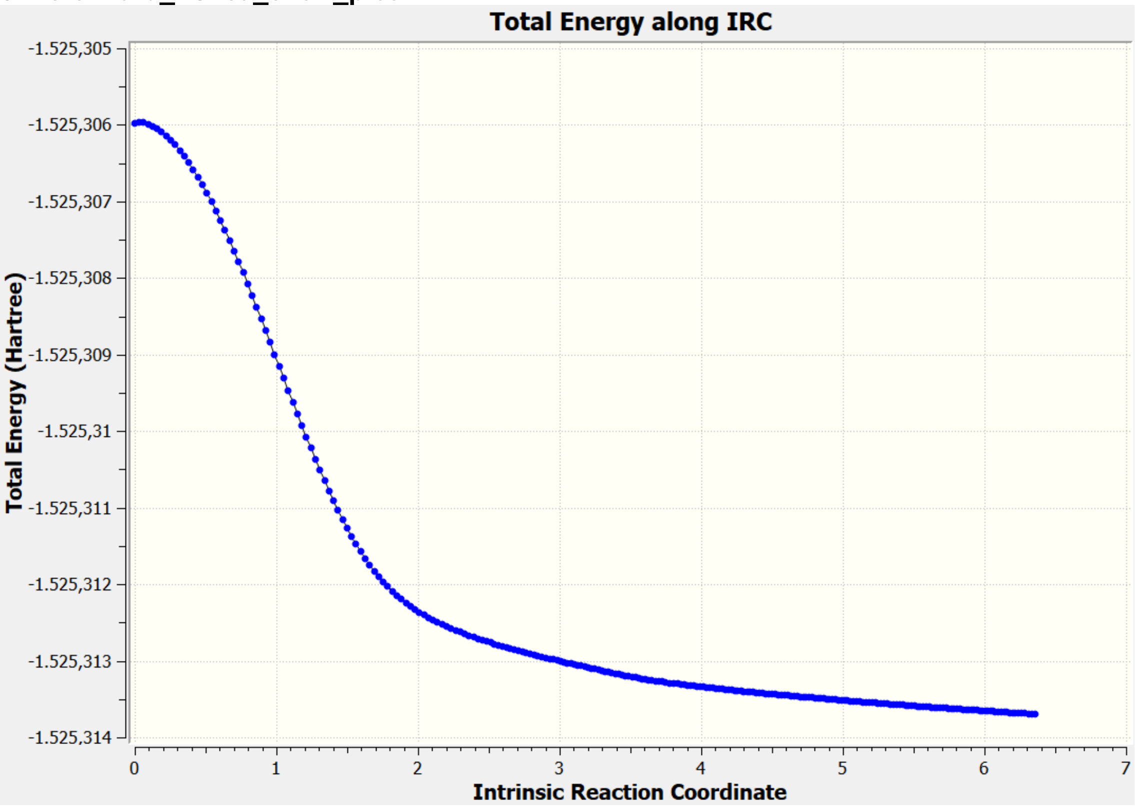

9. ircforward_TS1eb_endoA_plot

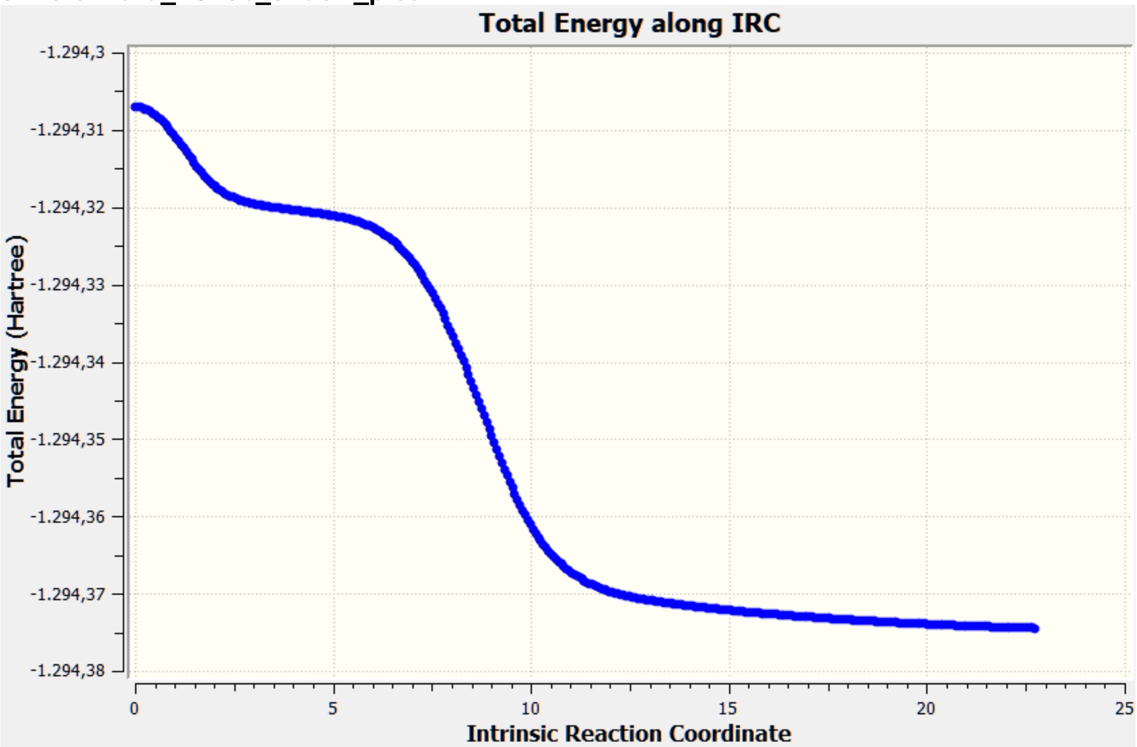

10. ircforward_TS1eb_exoA_plot

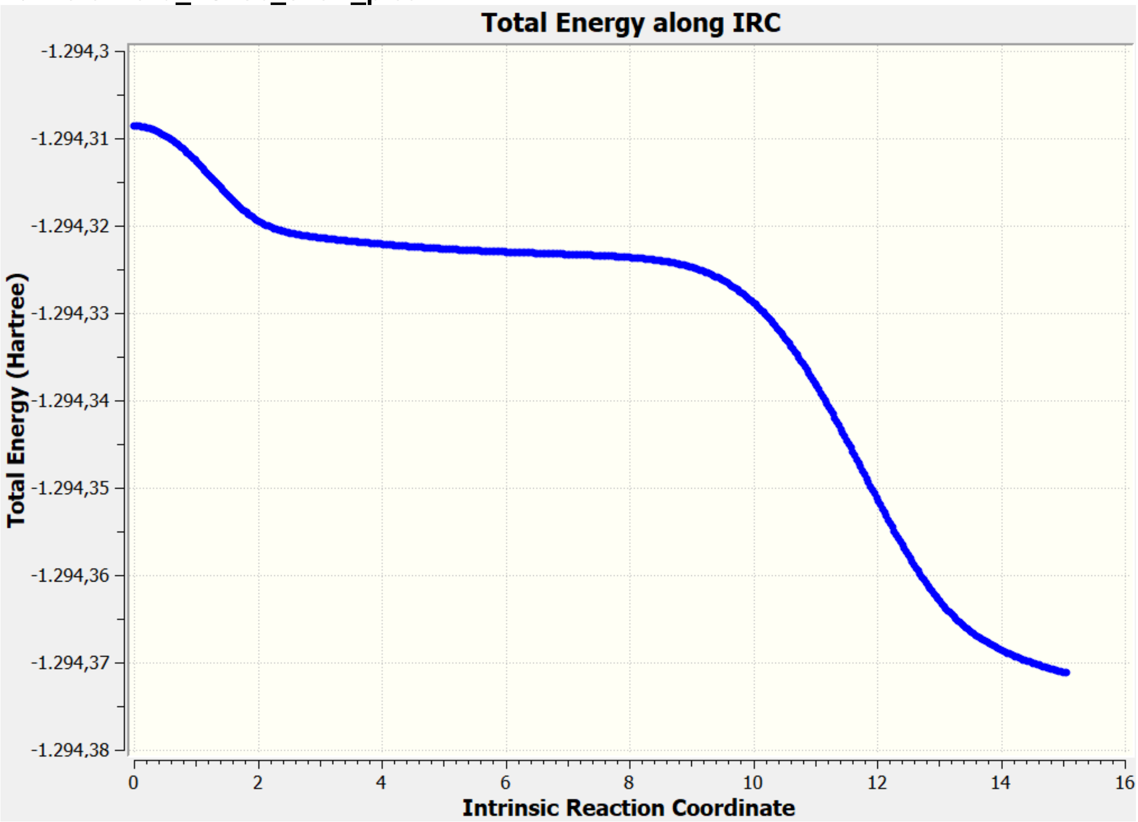

11. ircforward_TS2bb_endoA_plot

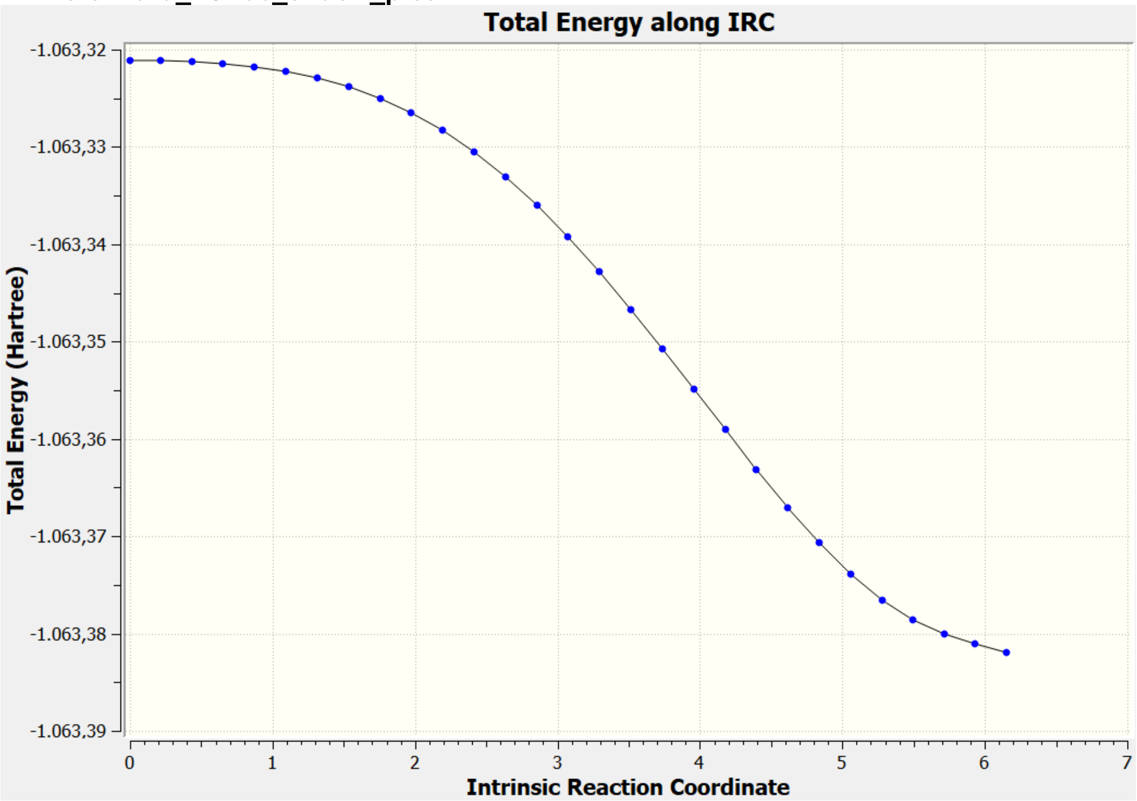

12. ircfoward_TS1bb_endoA_plot

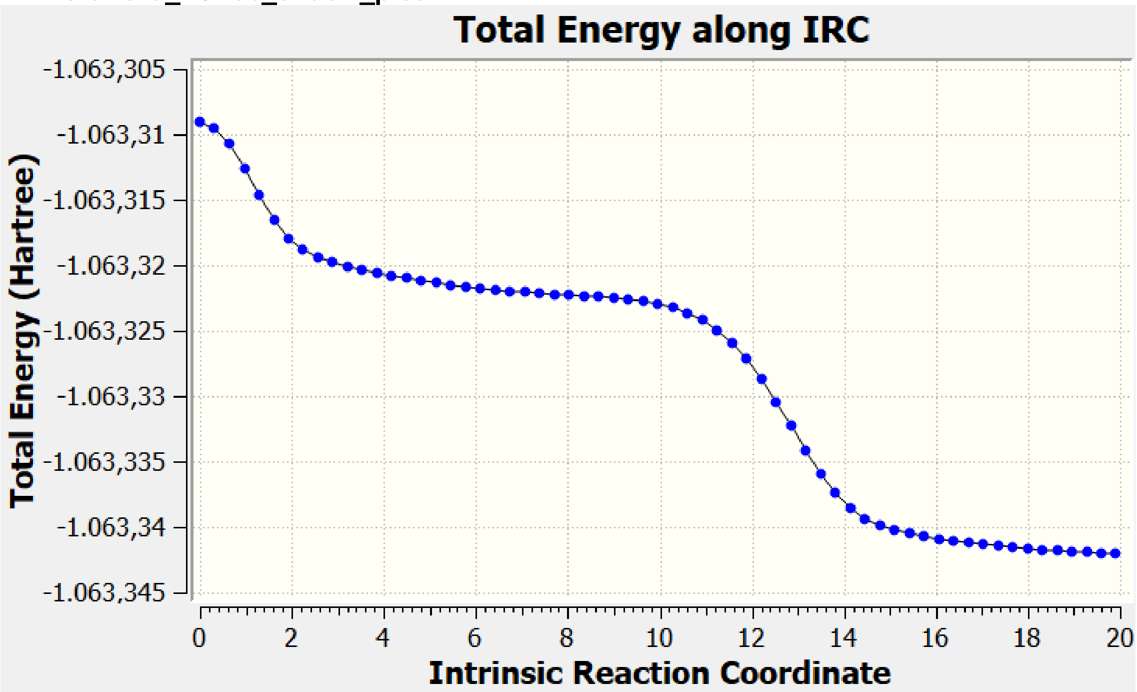

Supplement: Supplementary file 1 — jo4c00843_si_001.pdf [file jo4c00843_si_001.pdf]
